# Meta-proteomic analysis of the Shandrin mammoth by EVA technology and high-resolution mass spectrometry: what is its gut microbiota telling us?

**DOI:** 10.1007/s00726-021-03061-0

**Published:** 2021-08-28

**Authors:** Annamaria Cucina, Vincenzo Cunsolo, Antonella Di Francesco, Rosaria Saletti, Gleb Zilberstein, Svetlana Zilberstein, Alexei Tikhonov, Andrey G. Bublichenko, Pier Giorgio Righetti, Salvatore Foti

**Affiliations:** 1 Laboratory of Organic Mass Spectrometry, Department of Chemical Sciences, University of Catania, Viale A. Doria 6, 95125 Catania, Italy; 2 Spectrophon Ltd, Oppenheimer 7, 7670107 Rehovot, Israel; 3 Zoological Institute, Russian Academy of Sciences, Universitetskaya Nab.1, Saint-Petersburg, 199034 Russia; 4 Department of Chemistry, Materials and Chemical Engineering ‘‘Giulio Natta’’, Politecnico di Milano, Via Mancinelli 7, 20131 Milan, Italy

**Keywords:** Paleoproteomics, Meta-proteomics, Mammoth microbiota, Shotgun proteomics, High-resolution mass spectrometry, Orbitrap fusion tribrid, Deamidation

## Abstract

During the last decade, paleoproteomics allowed us to open a direct window into the biological past, improving our understanding of the phylogenetic relationships of extant and extinct species, past human diseases, and reconstruction of the human diet. In particular, meta-proteomic studies, mainly carried out on ancient human dental calculus, provided insights into past oral microbial communities and ancient diets. On the contrary, very few investigations regard the analysis of ancient gut microbiota, which may enable a greater understanding of how microorganisms and their hosts have co-evolved and spread under the influence of changing diet practices and habitat. In this respect, this paper reports the results of the first-ever meta-proteomic analysis carried out on a gut tissue sample some 40,000 years old. Proteins were extracted by applying EVA (ethylene–vinyl acetate) films to the surface of the gut sample of a woolly mammoth (*Mammuthus primigenus*), discovered in 1972 close to the Shandrin River (Yakutia, Russia), and then investigated via a shotgun MS-based approach. Proteomic and peptidomic analysis allowed in-depth exploration of its meta-proteome composition. The results were validated through the level of deamidation and other diagenetic chemical modifications of the sample peptides, which were used to discriminate the “original” endogenous peptides from contaminant ones. Overall, the results of the meta-proteomic analysis here reported agreeing with the previous paleobotanical studies and with the reconstructed habitat of the Shandrin mammoth and provided insight into its diet. The data have been deposited to the ProteomeXchange with identifier < PXD025518 > .

**Supplementary Information:**

The online version contains supplementary material available at 10.1007/s00726-021-03061-0.

## Introduction

The significant technological development, in terms of performance and sensitivity, of the mass spectrometers in the past 2 decades has expanded the application of MS-based approaches to the study of ancient proteins. Paleoproteomics, analogously to paleogenomics (i.e. the genome-scale analysis of ancient DNA), allowed to open a direct window into the biological past, improving our understanding of evolutionary history (Cappellini et al. 2018). In particular, characterization of the proteinaceous material extracted from fossils, bone, dental calculus (mineralized plaque), preserved food remains, potsherds, and ceramic vessels, provided new insights into the phylogenetic relationships of extant and extinct species (Cappellini et al. 2014; Cleland et al. 2016; Welker et al. 2015), the characterization of past human diseases (Hendy et al. 2016; D’Amato et al. 2018a), and the reconstruction of the human diet (Warinner et al. 2014a, b, c; Shevchenko et al. 2018; Greco et al. 2018; Tanasi et al. 2021). In particular, dental calculus has become recognized as one of the richest sources of ancient biomolecules, preserving molecular evidence of oral bacteria, the human host, as well as consumed foodstuffs (Hardy et al. 2009; Warinner et al. 2014a, b, 2015). In this respect, shotgun meta-proteomics (i.e. characterization of proteins expressed from multiple organisms in a sample) applied to dental calculus is emerging as a powerful tool for disease and dietary characterization of ancient populations. On the contrary, very few investigations regard the analysis of the ancient gut microbiota (i.e. the community of microorganisms inhabiting the digestive tract) from human or animal remains. At the end of the twentieth century, the intestinal contents of a 12,000-year-old mastodon were investigated (Rhodes et al. 1998). Authors cultivated 295 bacteria, 41% of which were *Enterobacteriaceae*, and identified 38 individual taxa by fatty acid-methyl ester (FAME) profiles and biochemical characteristics. However, it was never established if these microorganisms were direct descendants of the original intestinal microbiota. The gut microbiota, in many animal taxa, is highly influenced by host phylogeny, diet, and habitat (Muegge et al. 2011; Amato et al. 2013). Therefore, characterization of the ancient gut microbiota may enable a greater understanding of how microorganisms and their human or animal hosts have co-evolved and spread under the influence of changing diet practices and habitats. Overall, paleoproteomic research highlighted that proteins are more resistant to degradation than DNA due to their chemical and physical properties, and therefore they permit recovery information much further back in time than previously thought possible. On the other hand, it is also evident that diagenesis, which includes a complex network of reactions such as chemical modifications of amino acids, chemical degradation, and molecular breakdown, deeply affects the protein sequences. As a result, short and altered peptide fragments are usually recovered from ancient materials, providing an important challenge for protein identification (Cleland et al. 2020). Another major issue is represented by contamination of exogenous proteins, and even more in microorganisms’ studies, as they potentially provide false insights into protein composition, taxonomy assignment, and may also affect the detection and identification of the ancient proteins. Contamination can be introduced at nearly any stage of burial, excavation, storage, and analysis, but several concrete measures can be taken to reduce contamination from modern proteins also in the laboratory environment. The use of protected specimens, such as the internal surface of a gastrointestinal tract, could limit the risk of contamination from the external environment of the burial site (Drancourt and Raoul 2005). In any case, in compliance with protection guidance for archaeological samples, some precautions should be adopted to minimize the effects of contamination from modern proteins, but also authentication and validation criteria are needed to discriminate ancient endogenous proteins from contaminants (Hendy et al. 2018). In this respect, ancient proteins have been studied for their patterns of degradation and diagenetic chemical modifications (DCMs), and how these patterns can be used as markers for endogenous, ancient proteins as opposed to potential modern contaminants (Hill et al. 2015; Cleland et al. 2015; Cappellini et al. 2012). Last but not least, another important challenge is related to the inestimable value of archaeological samples, which requires minimally destructive or non-destructive sampling techniques. Although the advent of high-performance mass spectrometers allows the identification and quantification of low amounts of substances from small samples, most of the actual protocols and strategies are invasive and usually require micro-sampling. Therefore, in recent times, several strategies have been developed to mitigate artifact damage, including minimally invasive or non-destructive sampling approaches (McGrath et al. 2019; Fiddyment et al. 2015; Manfredi et al. 2016; Cicatiello et al. 2018; Ntasi et al.^2021^). One of the more promising non-invasive techniques is known under the acronym EVA (Barberis et al. 2018; Manfredi et al. 2017). It is based on films of ethylene–vinyl acetate studded with strong cation and anion exchangers as well as with C_8_ and C_18_ resins, which can extract proteins and small molecules from the surface of several types of supports. Although proteomic results obtained using this extraction technique appear to be promising, up-to-date EVA films have been applied mostly to documents and clothing items barely 100–150 years old (Righetti et al. 2020, 2021). In the present paper, we report for the first time the use of EVA technology on a tissue sample some 40,000 years’ old. In particular, EVA films were applied to a gut tissue sample of a woolly mammoth (*Mammuthus primigenus*), discovered in 1972 close to the Shandrin River (Yakutia, Russia), and, therefore, named the “Shandrin mammoth”. A meta-proteomic approach allowed us to get insight into the gut microbiota composition, which may be reasonably related to the last meal of the mammoth, its diet and habitat.

## Materials and methods

### Chemicals

The chemicals employed during the analysis were of the highest purity commercially available and used without further purification. Formic acid (FA), ammonium bicarbonate (AMBIC), dithiothreitol (DTT), iodoacetamide (IAA) were purchased from Aldrich (St. Louis, Missouri, USA), ammonia from Carlo Erba (Milan, Italy); sequencing Grade Modified Porcine Trypsin from Promega (Madison, WI, USA); water and acetonitrile (ACN) (OPTIMA^®^ LC/MS grade) for LC/MS analyses from Fisher Scientific (Milan, Italy). All the chemicals listed above were exclusively employed for the present study.

### The Shandrin mammoth

The Shandrin mammoth, a 60–70 years old woolly mammoth (*Mammuthus primigenus*), was discovered in 1972 by Kuzmin and Struchkov close to the Shandrin River, a tributary of the Indigirka River (Yakutia, Russia). Based on radiocarbon, its age was estimated as 41,750 ± 880 years (Kuzmin et al. 2003).

Mammoth was dried after excavation and exhibited in the Zoological Museum of the Russian Academy of Sciences in St. Petersburg without any chemical treatments. The collection access number of the intestine sample of the Shandrin mammoth is ZIN # 86,580.

### Synthesis and characterization of the EVA Film

A special plastic-like film based on ethylene–vinyl acetate (EVA) as a binder of ground AG 501 Bio-Rad mix-bed cation/anion exchange resins was prepared. A mixture was made comprising 60% 1–10 μm size ground beads and 30% EVA (the melting temperature was 75 °C). This mixture of melted EVA and Bio-Rad resins was poured in a "Brabender" mixer W30 and extruded via a "Brabender" extruder KE19 (both from Brabender GmbH, Duisburg, Germany) in the form of a thin film. The thickness of the film was 150–200 μm.

### Protein sampling by EVA diskette

Protein sampling by EVA diskettes was carried out at the Zoological Museum of the Russian Academy of Sciences in St. Petersburg. For sampling large sample surfaces, it is necessary to find regions with a higher concentration of proteins. For this search, the fluorescence of phenylalanine, tyrosine, and tryptophan under UV illumination was studied.

**Fig. 1:**
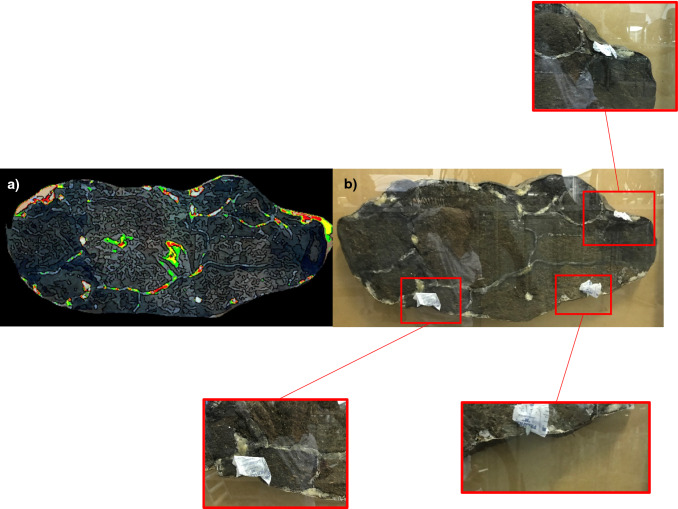
**a** Mapping of fluorescence of phenylalanine, tyrosine and tryptophan under flash UV illumination; **b** regions of sampling by EVA diskettes

UV LED for illumination and a digital camera with a special optical filter for fluorescence detection were used. The fluorescence level at each point was displayed in pseudo-colors on the instrument interface (green, yellow, and red—in order of increasing fluorescence intensity) (see Fig. 1a). This made it possible to quickly identify regions for sampling on paleontological samples. This portable system was made in SpringStyle Tech Design Ltd for quick examination of protein traces' presence on paleontological and archaeological samples.

A SpringStyle sensor for formaldehyde (FA) residuals detection to check the gut before sampling was applied. The selection criterion for the Museum's exhibits was the absence of formaldehyde processing of paleontological samples. Most of the samples from the paleontological collection in the 70s of the twentieth century were treated with formaldehyde. The specimen of the mammoth guts is one of the few exhibits that did not undergo this formaldehyde treatment.

The EVA diskette was gently humidified with ultrapure water and then placed on sample gut cavities in three regions for 60 min. To prevent drying of the EVA films, they were covered with parafilm^®^ from the outside (Fig. 1b).

### Protein extraction protocol

Protein extraction and sample handling were performed in a laboratory “clean room” dedicated to ancient protein analysis and using dedicated chemicals, lab glassware, and equipment. Surfaces and equipment were washed with 50% 2-propanol before use. Non-latex gloves were used. A section of the EVA diskette (5 mm × 5 mm) was cut with a scalpel and the proteins trapped in its film were eluted sequentially with 200 μL of volatile buffers (formate 5 mM at pH 3, followed by ammonia 0.5 mM at pH 10) and finally with volatile solvents (acetonitrile, OPTIMA® LC/MS grade, > 99.9%) to collect positively and negatively charged as well as hydrophobic proteins. The dried eluate was suspended in 50 mM AMBIC and the proteins were quantified by a fluorimetric assay using the Qubit Protein Assay kit with the Qubit 1.0 Fluorometer (ThermoFisher Scientific, Milan, Italy) (Saletti et al. 2018).

Then, about 7 μg protein extract was reduced, for 3 h at room temperature, by 5.4 μg of DTT (concentration of the stock solution: 10 mM) and alkylated, for 1 h in the dark at room temperature, by 11 μg of IAA (concentration of the stock solution: 45 mM). The sample was finally digested by 0.14 μg of porcine trypsin (overnight, 37 °C). The resulting peptide mixture solutions were dried under vacuum (Concentrator Plus, Eppendorf), re-dissolved in 50 µL of 5% aqueous FA, filtered by ultracentrifugation (750 µL, 0.2 µm Nonsterile Micro-Centrifugal Filters, Sepachrom, Rho, Milan), and analyzed by UHPLC/high-resolution nanoESI–MS/MS. An empty diskette of EVA film was used as a control sample. It was processed and analyzed by proteomics in the same way as the EVA diskettes placed in contact with the surface of the mammoth gut sample. Only a meagre number of background peptides was identified by database search in the EVA control sample (see Supplementary Material).

### Mass spectrometry analysis

Mass spectrometry data were acquired via a Thermo Fisher Scientific Orbitrap Fusion Tribrid^®^ (Q-OT-qIT) mass spectrometer (Thermo Fisher Scientific, Bremen, Germany). Liquid chromatography was carried out using a Thermo Scientific Dionex UltiMate 3000 RSLC-nano system (Sunnyvale, CA). One microliter of peptide mixture was loaded onto an Acclaim ^®^Nano Trap C18 Column (100 µm i.d. × 2 cm, 5 µm particle size, 100 Å). After washing the trapping column with solvent A (H_2_O + 0.1% FA) for 3 min at a flow rate of 7 μL/min, the peptides were eluted from the trapping column onto a PepMap^®^ RSLC C18 EASY-Spray column (75 µm i.d. × 50 cm, 2 µm particle size, 100 Å) and separated by elution at a flow rate of 0.25 µL/min at 40 °C by a linear gradient of solvent B (ACN + 0.1% FA) in A, 5% for 3 min, followed by 5% to 65% in 85 min, 65% to 95% in 5 min, finishing by holding 95% B 5 min, 95% to 5% in 10 min and re-equilibrating at 5% B for 15 min. The eluting peptide cations were converted to gas-phase ions by electrospray ionization using a source voltage of 1.75 kV and introduced into the mass spectrometer through a heated ion transfer tube (275 °C). Survey scans of peptide precursors from 200 to 1600 *m*/*z* were performed at 120 K resolution (@ 200 m/z). Tandem MS was performed by isolation at 1.6 Th with the quadrupole, HCD fragmentation with a normalized collision energy of 35, and rapid scan MS analysis in the ion trap (low-resolution MS/MS analysis). Only those precursors with charge state 2 ÷ 4 and intensity above the threshold of 1 × 10^3^ were sampled for MS^2^. The dynamic exclusion duration was set to 60 s with a 10 ppm tolerance around the selected precursor and its isotopes. Monoisotopic precursor selection was turned on. The instrument was run in full speed mode with 3 s cycles, meaning it would continuously perform MS^2^ events until the list of non-excluded precursors diminished to zero or 3 s, whichever is shorter. MS/MS spectral quality was enhanced by enabling the parallelizable time option (i.e., using all parallelizable time during full scan detection for MS/MS precursor injection and detection). Mass spectrometer calibration was performed using the Pierce^®^ LTQ Velos ESI Positive Ion Calibration Solution (Thermo Fisher Scientific). MS data acquisition was carried out by utilizing the Xcalibur v. 3.0.63 software (Thermo Fisher Scientific). To avoid cross-contamination with other biological samples, all solvents were prepared freshly, and ancient samples were not processed or analyzed in one batch with modern references. Also, to avoid carryover during nLC-MS/MS runs, from three to five blank runs were performed before each analysis using the same gradient program. Spectra acquired in the last blank run were searched by PEAKS software against the SwissProt database without species origin restrictions and using the same parameters of the archaeological samples.

### Database search

nLC–MS/MS data were processed by MaxQuant (MQ) software 1.6.17.0 (https://www.maxquant.org/). The raw data were analyzed and searched against three different databases separately i) a proteins database, referred to in the text as *Proboscidea* database (30,304 entries, February 2021) including Swiss-Prot and TrEMBL sequences of *Loxodonta africana*, *Elephas maximus*, *Mammut americanum,* and *Mammuthus primigenius;* (ii) a *Viridiplantae* database (Swiss-Prot; 40,656 entries, February 2021); and (iii) a *Bacteria* and *Nematoda* database including 334,868 and 5099 Swiss-Prot entries from bacteria and nematode, respectively (February 2021). Moreover, the common Repository of Adventitious Proteins (c-RAP; https://www.thegpm.org/crap/) contaminant database was enabled in the database search.

The first step of database search was carried out using the following parameters: (a) tryptic peptides with a maximum of three missed cleavage sites; (b) cysteine carbamidomethylation as a fixed modification; (c) oxidation of methionine, the transformation of N-terminal glutamine and N-terminal glutamic acid residue to pyroglutamic acid form, and the deamidation of asparagine and glutamine as variable modifications. The match type was “match from and to”. The decoy mode was “revert”. PSM, Protein, and Site decoy fraction FDR were set at 0.01 as the threshold for peptide and protein identifications. The minimum score for modified and unmodified peptides was set at 40. All the other parameters were set as default. In the data analysis, only peptides with intensity over the Max Quant threshold were considered.

Then, based on previous observation of ancient proteome degradation (Cappellini et al. 2012), the unassigned spectra were examined by an additional search function of Max Quant, named “dependent peptide search” (Tyanova et al. 2016), which allows the identification of potential DCMs related to aging and photo-oxidation damages (Pattison et al. 2012).

In the light of the results obtained by the first step of database search, each database was investigated to identify additional chemical modifications and improve peptides identifications. All the parameters were the same as the previous step; in particular, the following chemical modifications were investigated, as variable parameters: (i) oxidation, di-oxidation, formation of kynurenine, and formation of oxo-lactone, for tryptophan residues;( ii) oxidation, di-oxidation, iodination and di-iodination, and formation of dopaquinone, for tyrosine residues; (iii) acetylation of lysine; (iv) di-oxidation of methionine; (v) tri-oxidation of cysteine.

Finally, to be sure of the species assigned by the software to each protein identified, all the identified peptides underwent BLASTp (Basic Local Alignment Search Tool for protein) searches through the NCBI database (http://blast.ncbi.nlm.nih.gov/Blast.cgi) to validate species identifications and to rule out conserved peptides between species. A protein was considered identified if a minimum of two peptides were matched. The mass spectrometry proteomics data have been deposited to the ProteomeXchange Consortium (http://proteomecentral.proteomexchange.org) via the PRIDE partner repository (Perez-Riverol et al. 2019) with the dataset identifier < PXD025518 > .

### Calculation of the level of deamidation and other chemical modifications

An estimation of the percentage of deamidation for each sample was calculated with the aid of a freely available command-line script for Python 2.x (https://github.com/dblyon/deamidation), which uses the MaxQuant “evidence.txt” file (Mackie et al. 2018). The calculations were done separately for potentially original peptides and potential contaminants peptides as previously reported (Mackie et al. 2018; Tanasi et al. 2021). Analogously, estimation of the percentage of the other chemical modifications investigated was obtained applying the same model of the deamidation script, separately for potentially original and potentially contaminant peptides (See Supplementary Material).

### Meta-proteomics analysis

The meta-proteomics analyses were performed by consulting the open-source web application Unipept (Unipept 4.3; http://unipept.ugent.be) (Mesuere et al 2015), using the peptide matches with an ion score greater than 40, assigned by Max Quant to all the peptides matched, and with intensity over the Max Quant threshold.

The tool for meta-proteomics analysis is realized for tryptic peptides, obtained with a shotgun approach, from environmental samples. It can calculate the Lowest Common Ancestors (LCA) of a group of peptides, giving an insight into the biodiversity of the sample, and integrating complementary functional analysis (Mesuere et al 2018). Thanks to its algorithm, it shows the most specific taxonomic level for each peptide. Ubiquitarian peptides are generically assigned to “organism”.

## Results

As reported in the “Introduction”, the sequence and chemical structure of ancient proteins is altered by a series of complex diagenetic reactions. Diagenesis, including a complex network of reactions such as chemical modifications of amino acids, chemical degradation, and molecular breakdown driven by environmental factors, results in protein modifications beyond those produced in vivo and introduces a new challenge for protein identification and authentication (Cleland et al. 2020). Some substrates (e.g. bone, dental calculus, and eggshell) may harbor a better potential for preserving endogenous proteins than other specimens. On the contrary, for proteins present in tissues such as the gut, the microbial attacks, and diagenetic effects may be more extensive because of the poorer screen effect in comparison with the hydroxyapatite cage that protects bone proteins. Consequently, identification of proteins may result more challenging, or even fail, compared to other ancient samples. Taking into account this aspect and the very high level of degradation of our gut sample (a 40,000 years’ old tissue), the results obtained by the investigation of *Proboscidea*, *Viridiplantae,* and *Bacteria/Nematoda* databases were analyzed at both protein and peptide levels (i.e. not considering also the proteins from which these peptides come). In particular, all the “original peptides” identified by Max Quant were used to perform a meta-proteomics analysis by Unipept search engine, which uses the UniProt database, a version of the NCBI taxonomy, and an LCA algorithm (see Material and Methods section), to achieve a global vision about the taxonomic distribution of all peptides.

### Protein identification results

#### Proteins related to Mammoth

By searching the *Proboscidea* database and c-RAP as background database a total of 743 peptides were characterized (Supplementary Tables S1 and S2). 227 peptides were related to contaminant proteins of the c-RAP database, whereas 516 peptides were from proteins of *Proboscidea*. Among the c-RAP entries, some proteins (i.e. human keratins and albumin) were identified by peptide trait sequences shared with the homologs of *Loxodonta africana* (i.e., the African savanna elephant), and any *Loxodonta*-specific peptides from these proteins were recognized (Table S2). As a consequence, these proteins might be also related to the mammoth gut, and considered as potentially endogenous components of the sample. However, since it was not possible to establish the origin of these proteins, as a precaution, they were still considered contaminants.

Among the 516 peptides from *Proboscidea* entries, 83 were related to 21 proteins identified with at least 2 peptides, whereas 433 peptides came from other 433 different proteins (Table S1).

The twenty-one proteins identified with at least two peptides were further analyzed to validate species identifications. In particular, all the 83 peptides which allowed the identification of these proteins were subjected to a sequence search by BLASTp. By this search, the identified proteins were classified into three groups: (i) proteins specifically belonging to mammoth; these proteins were identified by at least a peptide (peptide marker) related only with the *Proboscidea* species; (ii) proteins related to many species, but not to human; these proteins were identified only with unspecific peptides, i.e. peptides related to more species, but not coming from *Homo sapiens*, and finally iii) proteins related to many species comprising human. These three groups include 3, 2, and 16 proteins, respectively, and are reported in Table 1. The last group also comprises three proteins (i.e. heat shock proteins, actin, and histone 4) that were identified with conserved peptides between *Proboscidea* and *Viridiplantae* or *Bacteria*.

**Table 1:** Classification of the proteins identified by searching *Proboscidea* database and after peptide BLAST search. More details are reported in the Supplementary Material (Table S1)

Proteins	Taxonomy^a^	Razor + unique peptides	Chemical modifications
Hemoglobin subunit beta	*Mammoth*	6	Deamidation (NQ), Oxidation (W), Dioxidation (W), Dioxidation (Y)
Hemoglobin subunit alpha	*Mammoth*	2	Deamidation (NQ)
Integrin subunit alpha E	*Mammoth*	2	Deamidation (NQ), Acetyl (K), Trioxidation (C)
Gamma-tubulin complex component	*Euteleostomi—not human*	2	Deamidation (NQ), Dioxidation (W)
Chromosome 12 open reading frame 60	*Not specific, but not human*	2	Oxidation (M), Glu- > pyro-Glu, Dioxidation (Y)
Desmoplakin	*Not specific (possibly human)*	17	Deamidation (NQ), Oxolactone, Gln- > pyro-Glu
Junction plakoglobin	*Not specific (possibly human)*	10	Oxidation (M), Deamidation (NQ)
Annexin	*Not specific (possibly human)*	6	Gln- > pyro-Glu
Peroxiredoxin 1	*Not specific (possibly human)*	2	Gln- > pyro-Glu
Plakophilin 1	*Not specific (possibly human)*	3	–
Desmoglein 1	*Not specific (possibly human)*	3	Gln- > pyro-Glu, Oxidation (M)
Transglutaminase 1	*Not specific (possibly human)*	2	–
Arginase	*Not specific (possibly human)*	3	Oxidation (M)
DNA helicase	*Not specific (possibly human)*	2	Oxidation (Y), Dioxidation (Y)
Catalase	*Not specific (possibly human)*	2	–
HECT-type E3 ubiquitin transferase	*Not specific (possibly human)*	2	Dioxidation (Y)
60S ribosomal protein L40	*Not specific (possibly human)*	2	–
Glyceraldehyde-3-phosphate dehydrogenase	*Not specific (possibly human)*	2	Gln- > pyro-Glu, Oxidation (M)
Heat Shock Protein^b^	*Not specific (possibly human)*	5^b^	Oxidation (M)
Actin^b^	*Not specific (possibly human)*	4^b^	
Histone H4^c^	*Not specific (possibly human)*	4^c^	Deamidation (NQ), Iodination(Y) Peptides in common with bacteria/nematoda search

More details are reported in the Supplementary Material (Table S1)

^a^Taxonomy classification after the BLASTp search

^b^Conservative peptides between *Proboscidea* and *Viridiplantae*. See the Supplementary Material

^c^Conservative peptides among *Proboscidea, Viridiplantae,* and *Bacteria.* See the Supplementary Material

#### Proteins related to *Viridiplantae*, *Bacteria*, and *Nematoda*

MS data were also used to investigate separately the *Viridiplantae,* and the *Bacteria/Nematoda* databases. By searching the *Viridiplantae* database, 426 potentially “original peptides” (i.e. not included in the c-RAP database) were identified. 42 out of 430 peptides allowed the identification of 17 potentially “original proteins” from *Viridiplantae* with at least two peptides (Supplementary Table S3). These proteins were further analyzed to validate species assignment as above reported. In this way, we obtained the unequivocal species identification for seven proteins (Table 2). In particular, these proteins belong to *Prunus*, *Arabidopsis thaliana, Tetradesmus obliquus*, *Chaetosphaeridium globosum* (freshwater green algae), and *Huperzia lucidula,* a clubmoss. The identification of proteins from the model plant *Arabidopsis thaliana* could be related to the predominant presence of entries from this species in the currently available protein databases, and a misinterpretation cannot be excluded. On the other hand, it should be noted that genetic studies suggested that this species was involved in the Pleistocene dynamics, and was preserved in the periglacial refugial areas (Beck et al. 2008; Yin et al. 2010). Similarly, the identification of proteins related to *Tetradesmus obliquus* and *Chaetosphaeridium globosum*, two unicellular species of green algae from the group *Chlorophyta*, cannot exclude, in principle, the presence of closely related species not represented in the current database. However, unicellular algae, and in particular the group of *Chlorophyta*, have been previously reported as the predominant *Archeoplastida* in Late Pleistocene deposits (Shmakova et al. 2020). Moreover, six proteins are related to the *Poaceae*, *Pentapetaleae*, *Magnoliopsida*, *Mesangiospermae,* and *Euphyllophyta*, and a histone H4 was unspecific for *Viridiplantae* (Table 2). Finally, two proteins were identified with peptides shared with *Proboscidea*.

**Table 2:** Classification of the proteins identified by searching *Viridiplantae* database and after peptide BLAST search

Proteins	Taxonomy^a^	Razor + unique peptides	Chemical modifications
Prunin 1 Pru	*Prunus*	3	–
Probable disease resistance protein RPP1	*Arabidopsis thaliana*	2	Dioxidation (W)
Protein tesmin/TSO1-like CXC 4	*Arabidopsis thaliana*	2	–
Probable cysteine protease RD19B	*Arabidopsis thaliana*	2	Ox (M), Deamidation (NQ), Acetyl (K)
Uncharacterized membrane protein ycf78	*Tetradesmus obliquus*	2	Deamidation (NQ)
DNA-directed RNA polymerase subunit beta	*Chaetosphaeridium globosum*	2	Ox (Y)
Protein TIC 214	*Huperizia lucidula*	2	Ox(M), Deamidation (NQ), Acetyl(K)
Kinesin-like protein KIN-8B	*Poaceae*	2	Deamidation (NQ), ox(M)
DNA-directed RNA polymerase subunit alpha	*Pentapetaleae*	2	Iodination, Ox (Y)
Acyl-CoA-binding domain-containing protein 4	*Magnoliopsida*	2	Deamidation (NQ), Dioxidation (Y)
Glyceraldehyde-3-phosphate dehydrogenase	*Mesangiospermae*	3	–
Transcription factor HY5	*Magnoliopsida*	2	Deamidation (NQ), DQ formation
ATP-synthase subunit beta, chloroplastic	*Euphyllophyta*	2	Ox(M), Deamidation (NQ)
Histone H4^c^	*Not specific*	5^c^	Ox(M)
Heat Shock Protein^b^	*Not specific (possibly human)*	5^b^	Oxidation (M)
Actin^b^	*Not specific (possibly human)*	4^b^	

More details are reported in the Supplementary Material (Table S4)

^a^Taxonomy classification after the BLASTp search

^b^Conservative peptides between *Proboscidea* and *Viridiplantae*. See the Supplementary Material

^c^Conservative peptides among *Proboscidea, Bacteria,* and *Viridiplantae*. See the Supplementary Material

By the same approach, a database including only *Bacteria* and *Nematode* entries was also investigated, and 430 “original peptides” were characterized. However, only two proteins were identified with at least two peptides, the actin and the histone 4. However, these proteins were identified with peptides shared by the homologs from the *Proboscidea.* (Supplementary Table S4).

### Unipept analysis results

#### Peptides related to Mammoth

**Fig. 2:**
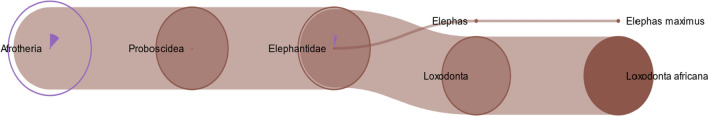
Tree view of the identified peptides belonging to *Afrotheria*. The percentage of peptides is calculated considering the 100% as the total number of peptides of the previous node

Unipept analysis of the peptides characterized by searching the *Proboscidea* database allowed the classification of 369 sequences that were specific for the domain of *Eukaryota*. 109 out of 369 sequences were specific for the superorder of *Afrotheria*, and finally, 95 out of 109 were specific for the family of *Elephantidae* (Fig. 2). *Loxodonta* represented 95% of *Elephantidae* peptides (90 peptides), whereas only one sequence was attributed to *Elephas maximus*. However, it should be noted that 85% of the sequence in the *Proboscidea* database are from *Loxodonta*, thus this result has not to be intended as evidence of a closer phylogenetic relationship with the woolly mammoth.

#### Peptides related to *Viridiplantae*

**Fig. 3:**
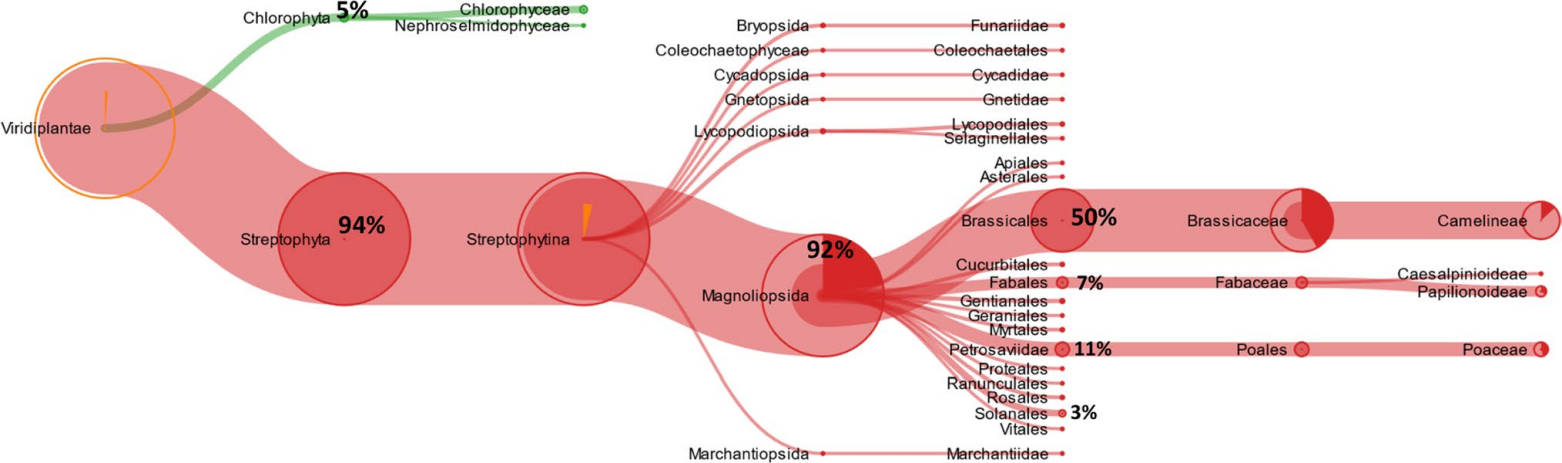
Meta-proteomic analysis: tree view of the identified peptides belonging to *Viridiplantae.* The percentage of peptides is calculated considering the 100% as the total number of peptides of the previous node

Among the 426 peptides identified by searching the *Viridiplantae* database, 265 sequences were classified, by Unipept analysis, specific for the clade *Viridiplantae*. Figure 3 shows the tree-graph results of Unipept investigation. Most of the peptides (249 out of 265 sequences; 94%) were related to the clade of *Streptophyta*, whereas about 5% (13 out of 265 sequences) to the clade of *Chlorophyta*. Moreover, this approach revealed that among the 249 *Streptophyta*-related sequences, 92% (corresponding to 230 sequences) belong to the class of *Magnoliopsida*, and are mainly specific (i.e.115 out of 230 peptides) of the *Brassicaceae* family. Figure 3 also shows that another group of peptides related to the class of *Magnoliopsida* appears specific to the orders of *Poales* (25 sequences), *Fabales* (15 of *Fabaceae*, of which 14 of the subfamily of *Papilionoideae*), and *Solanales* (7 sequences of *Solanaceae* and *Convolvulaceae*).

#### Peptides related to *Bacteria* and *Nematoda*

Among the peptides identified by searching the *Bacteria/Nematoda* database, 277 were specific to *Bacteria*, whereas only ten sequences resulted specifically in the *Nematoda* phylum. The meagre number of peptides related to *Nematoda* could be due to the limited number of entries (about 5000 proteins) of this phylum up to date reported in the Swiss-Prot database. However, all the peptides related to *Nematoda* were specific to *Rhabditida*, an order of phytoparasitic and zooparasitic microbivorous nematodes living in soil, and nine out of ten peptides were specific to the *Caenorhabditis* genus.

**Fig. 4:**
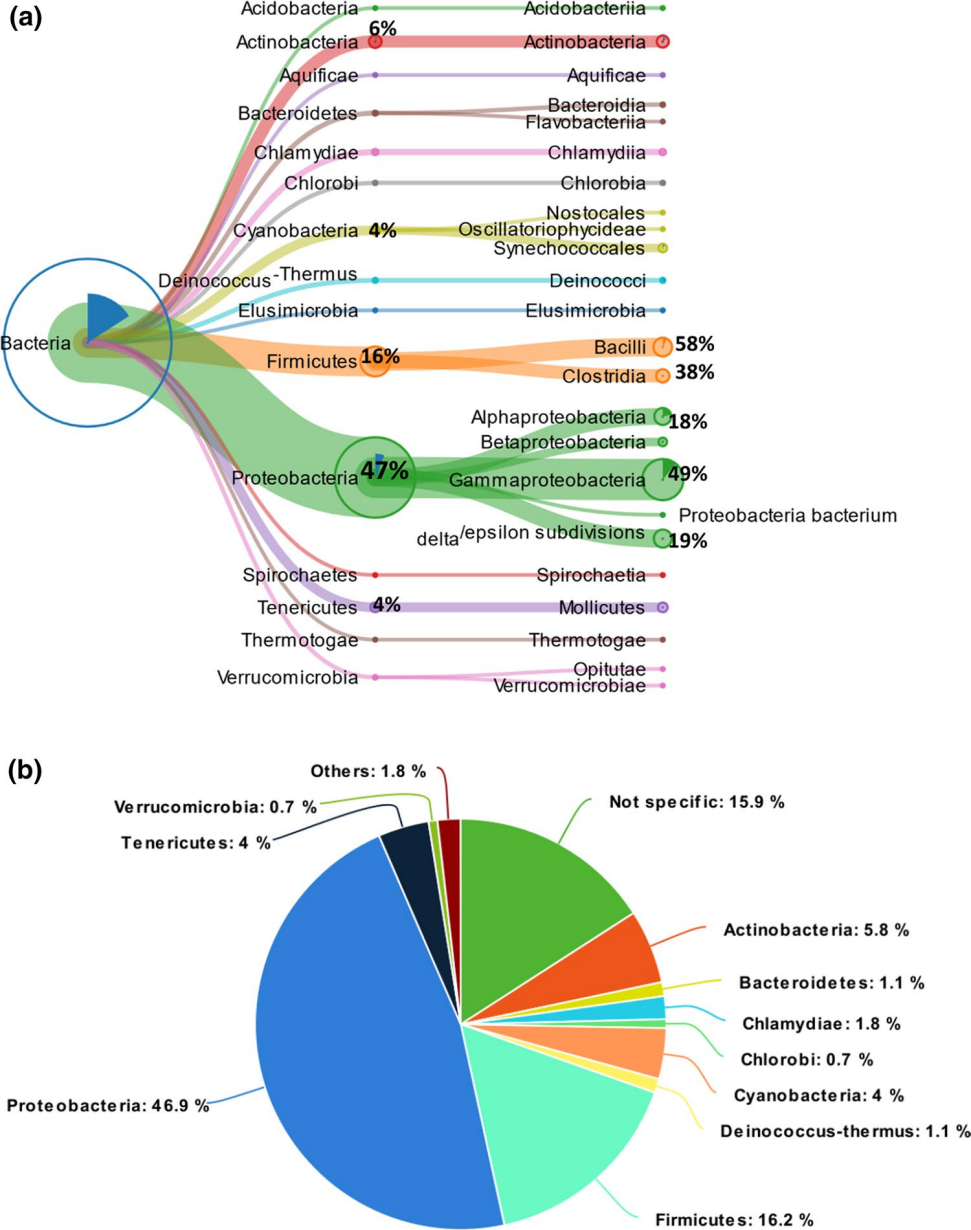
**a** Meta-proteomic analysis: tree view of bacteria peptides. The percentage of peptides is calculated considering the 100% as the total number of peptides of the previous node; **b** pie chart of the percentage of peptides for each phylum

Among the 277 sequences of *Bacteria*, about 63% can be related to two main phyla: *Proteobacteria* (46.9%; corresponding to 130 peptides), and *Firmicutes* (16.2%; corresponding to 45 peptides). The remaining peptides were related to other phyla including *Actinobacteria* (5.8%; 16 peptides), *Cyanobacteria* (4%; 11 peptides), *Tenericutes* (4%; 11 peptides), *Chlamydiae* (1.8%; 5 peptides), *Deinococcus–Thermus* (1.1%; 3 peptides), *Bacteroidetes* (1.1%; 3 peptides), and other bacteria phyla represented by a lower number of peptides (Fig. 4a, b).

### Level of deamidation and other chemical modifications

**Fig. 5:**
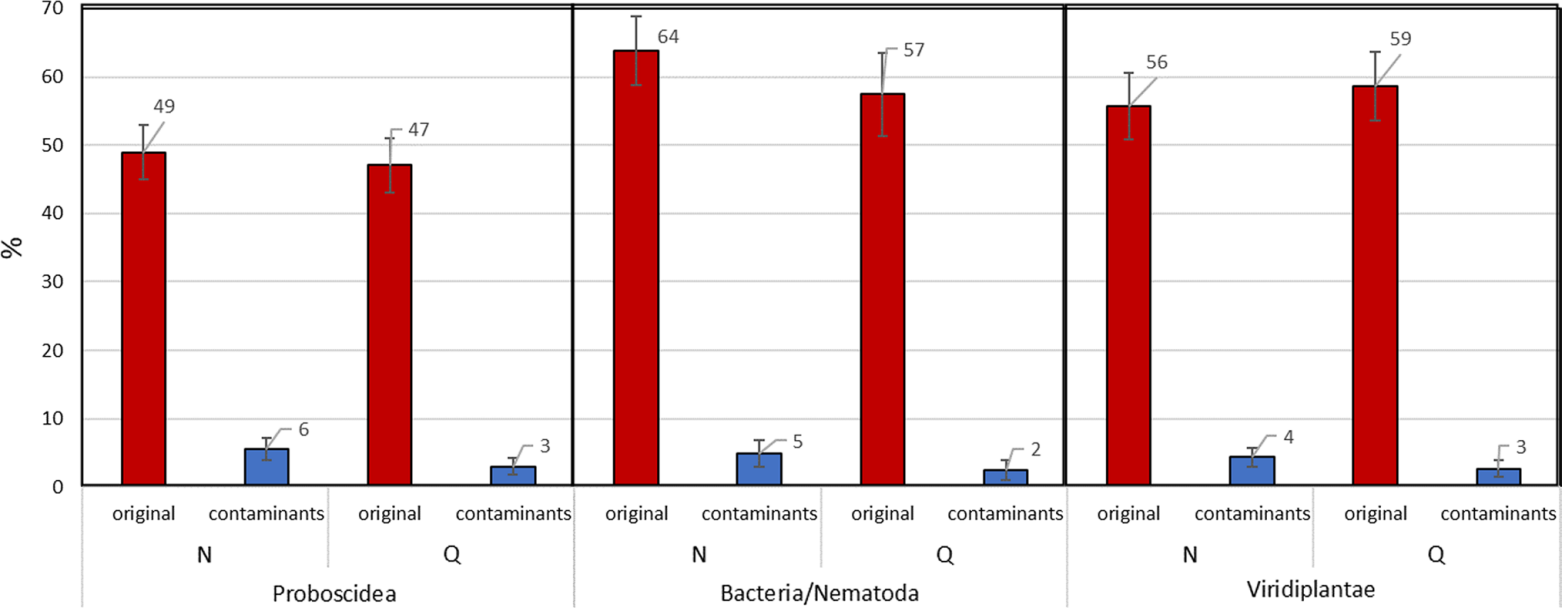
Deamidation level (reported as percentage) of asparagine (N) and glutamine (Q) residues in the *Proboscidea *(gut), *Viridiplantae* and *Bacteria*/*Nematoda* peptides. The level of deamidation of contaminant peptides identified in each database search is also reported. The error bars indicate the standard deviation calculated after 1000 bootstrap iterations

To discriminate the original endogenous components, present in the investigated gut, from components that are instead probably contaminants related to the post-excavation history of the sample, we investigated the deamidation (i.e. the removal of an amide group) of glutamine and asparagine residues, which are transformed into glutamic and aspartic acids, respectively, (Schroeter and Cleland 2016). As known, asparagine and glutamine residues naturally deamidate over time (Robinson and Robinson 2001; Robinson 2002). Even if different environmental factors and the inherent properties of proteins (i.e. primary sequence and three-dimensional structure) may affect the level of the deamidation process, and therefore the deamidation rate could display a high degree of variability, in almost all studies carried out up to date, it has been observed that it is generally much higher in ancient molecules than in modern ones. Therefore, the site-specific deamidation of asparagine and, mainly, of glutamine residues represents a useful tool for identifying modern contamination, and it is one proposed marker of age recently adopted in many archaeological and paleontological studies. In the meantime, it is important to highlight the need for multiple lines of evidence to authenticate ancient protein data (van Doorm et al. 2012; Wilson et al. 2012; Chowdhury et al. 2019; Ramsøe et al. 2020, 2021; Tanasi et al. 2021). In this study, the deamidation level of asparagine and glutamine residues was calculated for all the three types of peptides classified as “original”: i.e. peptides related to *Mammoth*, *Viridiplantae*, and *Bacteria/Nematode*. These results were compared with the deamidation level of those peptides classified as “contaminants” (Fig. 5), because belonging to the proteins of the c-RAP database. Figure 5 shows that the deamidation level of the “original peptides” ranges from 49 to 64% for the asparagine residues and from 47 to 59% for the glutamine residues. On the contrary, “contaminant peptides” present a deamidation level of 4–6% for the asparagine residues and 2–3% for glutamine residues. These results highlight two important aspects. Peptides from *Mammoth*, *Viridiplantae*, *Bacteria*, and *Nematode* show a similar deamidation level. This result supports the hypothesis that peptides from plants, bacteria, and nematodes here detected are reasonably ancient as much as the peptides of the mammoth. Furthermore, the deamidation level of the “original peptides” is from seven to twelve times higher for the asparagine residues and from fourteen to twenty-two times higher for the glutamine residues with respect to the corresponding deamidation level of “contaminant peptides”.

On the other hand, other DCMs have also been detected and studied concerning the degradation of ancient proteins. These forms of random, spontaneous, and non-enzymatic alterations are mainly related to oxidative stress and damage affecting chromophoric amino acids such as tryptophan (Trp), histidine (His), cysteine (Cys), methionine (Met), tyrosine (Tyr), and phenylalanine (Phe) (Stadtman et al. 2005; Pattison et al. 2012; Mikšík et al. 2016; Cannizzo et al. 2012; Pattinson et al. 2012; Davis 2016). Therefore, the level of the oxidation products at tryptophan, tyrosine, cysteine, and methionine residues was calculated for both the “original” and “contaminant” peptides.

Overall, the comparison of the level of the oxidation products of the above-reported amino acids in original and in contaminant peptides confirms the trend already observed for the deamidation, i.e. that ancient peptides have a much higher level of oxidation than the contaminant ones (see Supplementary Material and Fig. S1 for details).

## Discussion

The discovery of the Shandrin mammoth in the early summer of 1972 revealed that its internal organs were well preserved as frozen monoliths. A team of paleontologists, anatomists, microbiologists, parasitologists, and geologists examined the anatomy and the plants' macro-remains of the gastrointestinal tract. It was possible to weigh 291 kg of almost intact remains in the stomach, and 25 kg of decomposed parts in the intestine, and to observe extensive hemorrhages in muscular and submucosal layers (Yudichev and Averikhin 1982). In particular, the mammoth had died in the early spring of asphyxia due to acute meteorism of the stomach and the gut because of the consumption of poorly digestible forage such as old dry grass, turf, and bush branches. Paleobotanic investigation (Ukraintseva 1979) evidenced that remains of the gastrointestinal tract of the mammoth were mainly from moss spores (77%) and grass pollen (19%), together with low amounts (4%) of tree, shrub, and undershrub pollen. Studying the composition of plants that animals ate before they died, could help not only to reconstruct the flora of the area but also to understand the living conditions and probably the reasons for the extinction. During the Kargin interglacial time, climatic conditions in Siberia were milder, and probably the change in environment was harmful to animals such as mammoths, *ovibos,* and others who were more used to a “dry” cold climate (Ukraintseva 1979). Therefore, joining paleobotanical records of pollen, DNA, and macrofossils of plant remains of the gastrointestinal tract, it was supposed that the Shandrin region had vegetation highly similar to the Arctic or alpine heathland, taiga, bog, and tundra grassland, and with low similarities for desert, semi-desert, steppe or marsh. Interestingly, the macrofossils were also typical of a fen and swamp woodland. On the other hand, the pollen, representing the wide surroundings of the study site due to wind transport, was also similar to deciduous scrub vegetation. This evidence supports the hypothesis that most of the well-preserved animals probably died near water sites where they were looking for water or fresh vegetation (Axmanová et al. 2020). Furthermore, biochemical studies showed a proteins-rich environment in the mammoth intestine (Ukraintseva 2013a, b), but proteomics analyses were never performed. *Proboscidea* is a hindgut fermenter, using the cecum for fermentation of plant fiber (Fowler and Mikota 2006); hence, even if a wide amount of plants and pollen remains were identified in the stomach, which plays a storage function, little is known about the forage already or partially digested present in the intestine. In this respect, the proteomic analysis of the gut carried out by coupling the non-invasive EVA technique with the high-resolution Orbitrap Fusion Tribrid mass spectrometer allowed in-depth exploration of the meta-proteome composition.

First of all, some of the identified proteins (i.e. ATP synthase, tubulin, annexin, hemoglobin) related to the mammoth, and in general to *Proboscidea*, represent part of the most abundant components of the Mammalia intestine. Besides, keratins are reported among the most abundant gut proteins, but in our sample, it was not possible to distinguish between mammoth and human origin of these proteins (Rodríguez-Piñeiro et al. 2013).

Moreover, most of the results of our meta-proteomic analysis agree with the previous paleobotanical studies and with the reconstructed habitat of the Shandrin mammoth. In this respect, the hypothesis of the proximity with a water site is supported by the identification of freshwater green algae such as *Tetradesmus obliquus* and *Chaetosphaeridium globosum*, probably part of the dietary habit of the mammoth. On the other hand, the identification of many peptides of *Papilionoideae* in the intestine could be related to the *Oxytropis sordida*, a species of flowering plant belonging to the subfamily of *Papilionoideae*, whose presence is documented in the vicinity of the Shandrin mammoth’s burial site (Chuck et al. 1997; Solonevich et al. 1977). Moreover, the identification of a protein (i.e. Protein TIC 214) from *Huperzia*, a genus observed in other close Siberian sites (Ukraintseva 2013b) might confirm the presence of moss. Very interesting appears the identification of three peptides of a protein (i.e. Prunin 1 Pru, *Prunus* species), belonging to the family of *Rosaceae*. *Rosaceae* spores and pollens were observed in the gastrointestinal tract of the Shandrin mammoth in previous studies (Ukraintseva 2013b). The presence of *Prunus* plants during the Late-Pleistocene (126,000–11,700, Before the Present Era, BP) in Siberia was never proved through the identification of remains. However, a very recent investigation (Fletcher et al. 2021) reports the presence of *Prunus* plants during the late Miocene (from 23 million years ago, Ma, to 5 Ma BP) and early Pliocene (from 5 to 2.5 Ma BP). Moreover, in a recent study (Volkova et al. 2020) a phylogeographic analysis of contemporary populations was performed, which excluded the possibility of complete extinction of *Prunus padus* during the Late-Pleistocene. Furthermore, this work suggested the survival of this plant to the glaciation during the early Pleistocene in many periglacial microrefugia at high latitudes and its subsequent diffusion to new areas during interglacial periods. Therefore, our identification of a protein of *Prunus* would be the first molecular evidence of the hypothesis above reported. On the other hand, previous studies on the intestine and stomach of Shandrin mammoth relate the identification of traces of the larvae of nematodes, even if it was not possible to reliably determine the species-specific endoparasite (Serdyuk 2018). Despite the limited *Nematoda* protein database, our meta-proteomic analysis suggests the presence of *Rhabditida*, an order of phytoparasitic and zooparasitic microbivorous nematodes living in the soil. In particular, most of the peptides related to *Rhabditida*, belong to the *Caenorhabditis* genus, characterized for living in bacteria-rich environments, such as sites of plants and fruit decomposition (Frézal and Félix 2015). However, it should be noted that *Caenorhabidits* are the most studied nematodes, so that the presence of another species of nematodes, which up to date are not represented in current databases, cannot be excluded.

There is another interesting aspect of the present results worth elaborating upon and regards the analysis of the bacteria microbiota, which is the community of commensal, symbiotic, and pathogenic microorganisms playing an important role in gastrointestinal regulation (Feng et al. 2018). Characterization of gut bacteria may help to shed light not only on the last meal of the mammoth but also on its dietary habits and habitat. In this respect, the results of characterization of the gut microbiota of the Shandrin mammoth appear to agree with those reported in a very recent investigation (Budd et al. 2019) carried out on the fecal microbiota of wild African savanna elephant (*Loxodonta africana*) and the African forest elephant (*Loxodonta cyclotis*) (Fig. 4b). In particular, our results highlight that the microbiota of the Shandrin mammoth was dominated by *Proteobacteria* (47%) and showed a lower content of *Firmicutes* (16%), similarly to the microbiota composition observed for the African forest elephant (*Proteobacteria,* 52%; *Firmicutes,* 17%). On the contrary, the microbiota composition of the Shandrin mammoth appears very different from that reported for the African savanna elephant and the Asian elephant (*Elephas maximus Linnaeus*) (Zhang et al. 2019), whose microbiota composition was dominated by *Firmicutes* (~ 40%), *Bacteriodetes* (~ 20%), and *Proteobacteria* (~ 20%).

As hypothesized (Budd et al. 2019), the different gut microbiota compositions observed between *L. cyclotis* and *L. africana* may be a result of their dietary differences. In particular, *L. cyclotis* has a diet that is higher in fruit, and, therefore, has a higher proportion of simple carbohydrates and a lower fiber content (Moermond and Denslow 1985), whereas woody browse and grasses primarily compose the *L. africana* diet. Therefore, the very high similarity of the composition of gut microbiota of the Shandrin mammoth and the forest elephant is an indirect confirmation of the mammoth environment, which was a paludal forest. Moreover, identification of the *Prunus* protein, probably coming from the fruit, and the predominance of grasses and mosses in the intestine, perhaps at a digestive phase before the mammoth death, agree with the supposed reduced intake of woody materials.

## Conclusions

Shotgun proteomic analysis of the Shandrin mammoth’s gut carried out by coupling the EVA film technology and high-resolution mass spectrometry allowed in-depth exploration of the meta-proteome composition. The MS data interpretation aimed at the identification of the protein permitted the description of proteins specific to the tissue investigated, but also of some plant proteins which may be related to the last meal of the animal. On the other hand, data interpretation aimed to explore the taxonomic distribution of all the peptides identified (i.e. not considering also the proteins from which these peptides come) highlighted the predominance of some taxonomies among *Viridiplantae*, *Bacteria*, and *Nematoda* which are in agreement with previous studies and with the reconstructed habitat of the Shandrin mammoth. However, even if the overall results here reported agree with the previous investigations of the Shandrin mammoth, it should be noted that they represent an indirect picture of the proteins present in the gut and might not be fully representative of the investigated tissue. The EVA technology, while being minimally invasive, is a "surface" analysis that samples only the proteins of the most superficial layer, and might have led to miss some other components. On the other hand, although we cannot deny that, in principle, a more detailed picture could be obtained with a classical sampling, it should be highlighted that these last types of analyses usually require the destruction of part of a sample of tissue, a practice which is (obviously) discouraged by most museums.

Finally, if palaeproteomics and palaeogenomics of microbiota of dental calculus are becoming a masterpiece in the evolution studies (Warinner et al. 2015), proteomic analysis of the microbiota of the ancient gut is very limited, because of the difficult preservation of this kind of soft tissues. What is more, the lack of information about the microbiota does not concern only the mammoth, but also the modern elephant. Not before the last few years, a few works about Asian and African elephants’ microbiotas have been realized to identify the correlation among food, habitat, and bacteria composition (Budd et al. 2019; Jakeer et al. 2020; Zhang et al. 2019). In this respect, the results here reported represent the first attempt to describe in-depth the proteome and peptidome composition of a tissue sample some 40,000 years’ old by coupling the EVA technology and the high-resolution mass spectrometry.

## Supplementary Information

Below is the link to the electronic supplementary material.

Supplementary file1 (DOCX 393 kb)
