# Supplementary material for: Meta-proteomic analysis of the Shandrin mammoth by EVA technology and high-resolution mass spectrometry: what is its gut microbiota telling us?
Source: Amino Acids. 2021 Aug 28;53(10):1507–21. doi: 10.1007/s00726-021-03061-0 (PMC8519927; doi:10.1007/s00726-021-03061-0)
Supplement: Supplementary file 1 — Supplementary file1 (DOCX 393 kb) [file 726_2021_3061_MOESM1_ESM.docx]

***Proteins and Peptides Identification***

The results of the research with the different chemical modifications were combined and the complete list of peptides and proteins are reported in the Supplementary Tables S1, S2, S3, and S4. Also proteins identified with only one peptide are listed.

Table S1 shows the list of *Proboscidea* proteins with the corresponding identified peptides.

Table S2 reports the list of proteins and peptides of c-RAP contaminants. Some contaminant proteins were identified only by peptides shared with keratin 14, keratin 17, keratin 32, keratin 27, and albumin of *Loxodonta africana.*

Table S3 shows the complete list of peptides and proteins identified from *Viridiplantae* database.

Table S4 reports the complete list of peptides and proteins of *Bacteria/Nematoda*.

***Table S1****. List of peptides and proteins of Proboscidea: for each peptide all the features (same sequence and different modifications) with the highest intensity are reported. Proteins identified with at least two peptides are reported in bold.*

| **Accession Number** | **Description** | **Peptide Sequence** | **Modifications** | **Charge** | **Theoretical Mass** | **Mass error**  **[ppm]** | **MaxQuant**  **Intensity** |
| --- | --- | --- | --- | --- | --- | --- | --- |
| **G3TRF5** | **Desmoplakin** | AELIVQPELK | Unmodified | 2 | 1138.66 | 0.49 | 2819600 |
|  |  | ALLQALLQTEDMLK | Oxidation (M) | 2 | 1601.87 | 0.60 | 1289400 |
|  |  | ALTGFDDPFSGK | Unmodified | 2 | 1253.59 | 1.97 | 422720 |
|  |  | FGDSNTVMR | Unmodified | 2 | 1025.46 | 0.94 | 223070 |
|  |  | GIVDSITGQR | Unmodified | 2 | 1044.56 | -0.51 | 316990 |
|  |  | GLVGIEFK | Unmodified | 2 | 861.50 | 0.51 | 1866200 |
|  |  | IEVLEEELR | Unmodified | 2 | 1128.60 | 0.66 | 1985500 |
|  |  | IQSQFTDAQK | Unmodified | 2 | 1164.58 | 0.69 | 1053600 |
|  |  | ISTEEAIR | Unmodified | 2 | 917.48 | 0.67 | 285470 |
|  |  | LLQLQEQMR | Unmodified | 2 | 1157.62 | -0.06 | 684060 |
|  |  | QQIQNDLNQWK | Gln->pyro-Glu | 2 | 1396.67 | 1.31 | 398540 |
|  |  | QVQNLVNK | Gln->pyro-Glu | 1 | 924.50 | 0.13 | 212420 |
|  |  | SQLQISNNR | Unmodified | 2 | 1058.55 | 1.03 | 474450 |
|  |  | SVEEVASEIQPFLR | Unmodified | 2 | 1602.83 | 1.70 | 448250 |
|  |  | SVQNDSQAIAEVLNQLK | Unmodified | 2 | 1855.96 | -2.37 | 365890 |
|  |  | TLELQGLINDLQR | Unmodified | 2 | 1511.83 | -3.20 | 854650 |
|  |  | VTQLTDRWQR | 2 Deamidation (NQ),Oxolactone | 2 | 1317.63 | -2.15 | 1131600 |
| **G3SR99** | **Junction plakoglobin** | ALMGSPQLVAAVVR | Oxidation (M) | 2 | 1426.80 | 0.19 | 3166600 |
|  |  | ALMGSPQLVAAVVR | Unmodified | 2 | 1410.80 | -0.46 | 560700 |
|  |  | HVAAGTQQPYTDGVR | Unmodified | 3 | 1598.78 | 0.30 | 146910 |
|  |  | LIILANGGPQALVQIMR | Oxidation (M),Deamidation (NQ) | 2 | 1823.03 | 0.82 | 1269900 |
|  |  | LIILANGGPQALVQIMR | Deamidation (NQ) | 2 | 1807.04 | -0.66 | 484990 |
|  |  | LLNDEDPVVVTK | Unmodified | 2 | 1340.72 | 0.09 | 2482600 |
|  |  | LLNQPNQWPLVK | Unmodified | 2 | 1448.81 | 1.93 | 1409000 |
|  |  | LNYGIPAIVK | Unmodified | 2 | 1086.64 | 0.43 | 1227800 |
|  |  | NEGTATYAAAVLFR | Unmodified | 2 | 1482.75 | -0.99 | 3093300 |
|  |  | RALMGSPQLVAAVVR | Oxidation (M) | 3 | 1582.90 | 0.81 | 159960 |
|  |  | TMQNTSDLDTAR | Unmodified | 2 | 1351.60 | 0.92 | 168380 |
|  |  | VSVELTNSLFK | Unmodified | 2 | 1235.68 | 0.74 | 3109600 |
|  |  | VSVELTNSLFK | Deamidation (NQ) | 2 | 1236.66 | 0.58 | 296500 |
| **G3T7E7** | **Annexin** | DALNIETAIK | Unmodified | 2 | 1086.59 | 1.01 | 723120 |
|  |  | GVDEVTIVNILTNR | Unmodified | 2 | 1541.84 | 0.91 | 1002300 |
|  |  | QDIAFAYQR | Gln->pyro-Glu | 2 | 1093.52 | 0.81 | 612930 |
|  |  | QDIAFAYQR | Unmodified | 2 | 1110.55 | -0.99 | 358420 |
|  |  | SLYYYIQQDTK | Unmodified | 2 | 1420.69 | 0.18 | 143040 |
|  |  | TNQELQEINR | Unmodified | 2 | 1243.62 | 0.46 | 1544500 |
|  |  | TPAQYDASELK | Unmodified | 2 | 1221.59 | -0.84 | 875340 |
| **P02085** | **Hemoglobin subunit beta** | EFTPDVQAAYEK | Unmodified | 2 | 1396.65 | 0.15 | 653730 |
|  |  | ELGGEALSR | Unmodified | 2 | 930.48 | -0.80 | 6596500 |
|  |  | LLGNVLVIVLAR | Unmodified | 2 | 1278.84 | -0.09 | 741400 |
|  |  | LLVVYPWTR | Unmodified | 2 | 1145.66 | -0.13 | 11453000 |
|  |  | LLVVYPWTR | Oxidation (W) | 2 | 1161.65 | 0.14 | 283490 |
|  |  | LLVVYPWTR | Dioxidation (W) | 2 | 1177.65 | 1.31 | 267060 |
|  |  | LLVVYPWTR | Dioxidation (Y) | 2 | 1177.65 | -0.99 | 154540 |
|  |  | VLTSFGEGLK | Unmodified | 2 | 1049.58 | 1.08 | 2395500 |
|  |  | VVAGVANALAHK | Deamidation (NQ) | 2 | 1149.65 | 0.45 | 303300 |
|  |  | VVAGVANALAHK | Unmodified | 2 | 1148.67 | -0.11 | 248340 |
| **P01955** | **Hemoglobin subunit alpha** | FLSNVSTVLTSK | Deamidation (NQ) | 2 | 1295.70 | 0.44 | 3253300 |
|  |  | FLSNVSTVLTSK | Unmodified | 2 | 1294.71 | -0.75 | 1525000 |
|  |  | MFFSFPTTK | Oxidation (M) | 2 | 1120.53 | 0.46 | 2999200 |
|  |  | MFFSFPTTK | Unmodified | 2 | 1104.53 | -0.39 | 341530 |
| **G3SR49** | **Glyceraldehyde-3-phosphate dehydrogenase** | QASEGPLK | Gln->pyro-Glu | 1 | 811.41 | 0.85 | 1071300 |
|  |  | VIISAPSADAPMFVMGVNHEK | 2 Oxidation (M) | 3 | 2244.09 | 0.35 | 313570 |
| **G3T9Y7** | **Plakophilin 1** | AVQYLSSQDEK | Unmodified | 2 | 1266.61 | -0.28 | 479350 |
|  |  | LDAEVPTR | Unmodified | 2 | 899.47 | 0.00 | 746380 |
|  |  | SPNQNVQQAAAGALR | Unmodified | 2 | 1523.78 | 1.05 | 1792300 |
| **G3TJF5** | **Desmoglein 1** | DGSNVIVTER | Unmodified | 2 | 1088.55 | 1.82 | 678620 |
|  |  | IIRQEPSDSPMFIINR | Oxidation (M) | 3 | 1930.99 | -0.77 | 381620 |
|  |  | QEPSDSPMFIINR | Gln->pyro-Glu | 2 | 1515.70 | 1.22 | 501870 |
| **G3TY97** | **Arginase** | DIVYIGLR | Unmodified | 2 | 947.54 | -0.09 | 595610 |
|  |  | TGLLSGLDIMEVNPSLGK | Oxidation (M) | 2 | 1858.97 | 0.12 | 426740 |
|  |  | VMEETLSYLLGR | Oxidation (M) | 2 | 1425.72 | -0.94 | 345410 |
| **G3TGS0** | **Transglutaminase 1** | IYYGTEAQIGER | Unmodified | 2 | 1398.68 | 0.99 | 287140 |
|  |  | NPLPVTLTNVVFR | Unmodified | 2 | 1468.84 | -0.09 | 421770 |
| **G3TIT8** | **Peroxiredoxin-1** | ATAVMPDGQFK | Oxidation (M) | 2 | 1179.56 | -0.66 | 340780 |
|  |  | LVQAFQFTDK | Unmodified | 2 | 1195.62 | 0.11 | 480460 |
| **G3UE91** | **HECT-type E3 ubiquitin transferase** | EVLQNQLGIR | Unmodified | 2 | 1168.66 | 0.57 | 1670700 |
|  |  | LSGNRGVQYTRLAVQR | Dioxidation (Y) | 3 | 1848.99 | -2.10 | 160530 |
| **G3TQD9** | **Integrin subunit alpha E** | TCLSTFKEWSNGSRLCER | Acetyl (K),2 Trioxidation (C) | 2 | 2368.01 | -2.39 | 4526800 |
|  |  | VQNIIQVKNVTK | 3 Deamidation (NQ) | 2 | 1385.78 | -0.31 | 214770 |
| **G3TQI0** | **60S ribosomal protein L40** | TITLEVEPSDTIENVK | Unmodified | 2 | 1786.92 | 0.74 | 1514000 |
|  |  | TLSDYNIQK | Unmodified | 2 | 1080.55 | -0.36 | 1917100 |
| **G3TJB6** | **Gamma-tubulin complex component** | RIQEFQESIPK | 2 Deamidation (NQ) | 2 | 1375.70 | 0.88 | 458220 |
|  |  | VFNFLWR | Dioxidation (W) | 2 | 1012.51 | 3.70 | 160670 |
| **G3TWI9** | **Chromosome 12 open reading frame 60** | EDVNIKDFFEQMLK | Oxidation (M),Glu->pyro-Glu | 2 | 1752.84 | 3.22 | 578190 |
|  |  | LSDFYRK | Dioxidation (Y) | 2 | 959.47 | -3.44 | 541030 |
| **G3TYY5** | **DNA helicase** | IEEIEREIIK | Unmodified | 2 | 1270.71 | 1.02 | 1309900 |
|  |  | TIQTIVFLYSLYKEGHTK | Oxidation (Y),Dioxidation (Y) | 2 | 2188.14 | -0.64 | 208590 |
| **G3U4D5** | **Catalase** | LSQEDPDYGIR | Unmodified | 2 | 1291.60 | -1.17 | 238280 |
|  |  | NLSVEDAAR | Unmodified | 2 | 973.48 | -0.22 | 692910 |
| **G3TW77** | **Heat shock proteins** | IINEPTAAAIAYGLDK | Unmodified | 2 | 1658,89 | -0,33 | 612010 |
|  |  | IINEPTAAAIAYGLDKK | Unmodified | 3 | 1786,98 | -1,17 | 391370 |
|  |  | NQVAMNPTNTVFDAK | Oxidation (M) | 2 | 1664,78 | 0,10 | 257990 |
|  |  | TTPSYVAFTDTER | Unmodified | 2 | 1486,69 | 0,78 | 433730 |
|  |  | VEIIANDQGNR | Unmodified | 2 | 1227,62 | 0,54 | 489420 |
| **G3UJE6** | **Histone H4** | TVTAMDVVYALKR | Oxidation (M) | 3 | 1481,79 | -0,012 | 113450 |
|  |  | VFLENVIR | Unmodified | 2 | 988,57 | 0,11 | 416690 |
|  |  | VFLENVIR | Deamidation (NQ) | 2 | 989,55 | -0,04 | 541050 |
|  |  | DNIQGITKPAIR | Unmodified | 3 | 1324,74 | 1,23 | 766490 |
|  |  | ISGLIYEETR | 3-iodotyrosine | 2 | 1305,51 | 0,95 | 966030 |
|  |  | ISGLIYEETR | Unmodified | 2 | 1179,61 | 1,15 | 3903700 |
| **G3T386** | **Actin gamma 1** | AGFAGDDAPR | Unmodified | 2 | 975,44 | -0,08 | 1435300 |
|  |  | GYSFTTTAER | Unmodified | 2 | 1131,52 | 0,69 | 1342100 |
|  |  | SYELPDGQVITIGNER | Unmodified | 2 | 1789,88 | 0,92 | 752110 |
|  |  | VAPEEHPVLLTEAPLNPK | Unmodified | 3 | 1953,06 | 0,03 | 916470 |
| G3STC3 | Kinesin family member 1B | YLLASENQR | Unmodified | 2 | 1092.56 | -1.27 | 854240 |
|  |  | YLLASENQR | Deamidation (NQ) | 2 | 1093.54 | 0.43 | 431540 |
| G3THY8 | RAB40C, member RAS oncogene family | SFSMANGMNAVMMHGR | 2 Oxidation (M),Deamidation (NQ) | 2 | 1772.71 | 2.66 | 27810000 |
|  |  | SFSMANGMNAVMMHGR | 3 Oxidation (M),Deamidation (NQ) | 2 | 1788.71 | -3.64 | 673110 |
| G3UBX2 | 2-oxoglutarate and iron dependent oxygenase domain containing 3 | KMAPNKGQGWK | Deamidation (NQ),Dioxidation (W) | 2 | 1276.62 | 3.00 | 810730 |
| G3UFG7 | 39S ribosomal protein L59, mitochondrial | MPQMIEDWR | Oxidation (M),Oxolactone | 2 | 1234.51 | 0.96 | 350850 |
| G3TQU8 | 3-hydroxybutyrate dehydrogenase 1 | GRVINISSMLGRMANPAR | 2 Oxidation (M) | 2 | 1974.03 | 0.20 | 409150 |
| G3SQM2 | 5-aminoimidazole-4-carboxamide ribonucleotide formyltransferase, EC 2.1.2.3, EC 3.5.4.10 (AICAR transformylase) (AICAR transformylase/inosine monophosphate cyclohydrolase) (Bifunctional purine biosynthesis protein ATIC) (IMP synthase) (Inosinicase) (Phosphoribosylaminoimidazolecarboxamide formyltransferase) | YGMNPHQTPAQLYTLK | 2 Deamidation (NQ),di-oxidation (M) | 2 | 1894.88 | 1.95 | 191250 |
| G3T4W3 | 78 kDa glucose-regulated protein (Binding-immunoglobulin protein) | SQIFSTASDNQPTVTIK | Unmodified | 2 | 1835.93 | -0.16 | 755090 |
| G3TUG5 | ACB domain-containing protein | AKWEAWNENK | Deamidation (NQ),Dioxidation (W) | 2 | 1307.58 | 2.49 | 135400 |
| G3SSS2 | Activating transcription factor 6 | KKIQMNSK | Oxidation (M),Deamidation (NQ) | 2 | 992.53 | -3.43 | 255860 |
| G3T1U4 | Acyl-CoA dehydrogenase family member 9 | VAMNILNSGRFSMGSTVAGMLK | 2 Oxidation (M) | 3 | 2315.14 | 2.02 | 169970 |
| G3TDA6 | ADAM metallopeptidase domain 23 | TLAGQYSKQMK | Oxidation (M),Deamidation (NQ) | 2 | 1270.62 | -2.42 | 76090 |
| G3U7S3 | ADAM metallopeptidase domain 29 | EMNTQGDRFGHCGINR | Oxidation (M),Deamidation (NQ) | 2 | 1907.80 | -0.27 | 802930 |
| G3TCT9 | Adenylate cyclase type 6, EC 4.6.1.1 | EDMMFHKIYIQK | Oxidation (M),Glu->pyro-Glu | 2 | 1579.75 | -2.03 | 138920 |
| G3T888 | Adipocyte plasma membrane-associated protein | MSEVDGLRQRR | Oxidation (M) | 2 | 1361.68 | 2.89 | 148210 |
| G3T5M1 | ALMS1 centrosome and basal body associated protein | INVNLENKDVMHTTK | Oxidation (M),Deamidation (NQ) | 2 | 1771.88 | 1.68 | 1104600 |
| G3TQ25 | Anaphase-promoting complex subunit 1 | WVDSNVPQIIR | Deamidation (NQ),Oxolactone | 2 | 1340.67 | -2.86 | 4070700 |
| G3T0D0 | Anaphase-promoting complex subunit 5 (Cyclosome subunit 5) | VDCKEQIRDVVYYQAR | Deamidation (NQ),Oxidation (Y) | 2 | 2057.98 | -4.42 | 180320 |
| G3SNH7 | APC membrane recruitment protein 2 | RGLKGIFSSMR | di-oxidation (M) | 2 | 1282.68 | -3.70 | 495880 |
| G3SWN8 | Aprataxin, EC 3.6.1.71, EC 3.6.1.72 (Forkhead-associated domain histidine triad-like protein) | AECNKGYVKVK | Acetyl (K),Trioxidation (C) | 2 | 1384.67 | 2.97 | 525260 |
| G3SUC3 | ArfGAP with RhoGAP domain, ankyrin repeat and PH domain 1 | RTLYIQGER | Deamidation (NQ),Dioxidation (Y) | 2 | 1167.59 | -0.47 | 1431500 |
| G3SX90 | Armadillo repeat containing 2 | GAVEILMNLIK | Deamidation (NQ) | 2 | 1200.68 | 3.81 | 334850 |
| G3TL39 | Armadillo repeat containing X-linked 2 | GSLFFLCTASGVCVK | 2 Trioxidation (C) | 2 | 1740.77 | 0.98 | 565290 |
| G3T874 | ARV1 homolog, fatty acid homeostasis modulator | CIECNEEAK | 2 Trioxidation (C) | 2 | 1247.43 | 3.37 | 1946800 |
| G3SXV8 | ASXL transcriptional regulator 1 | SLGEQNSVGGQGK | 3 Deamidation (NQ) | 2 | 1262.56 | -1.75 | 160170 |
| G3TRW4 | ATP binding cassette subfamily C member 1 | VDENQKAYYPSIVANR | Deamidation (NQ) | 3 | 1866.91 | 1.97 | 162490 |
| G3UKQ2 | ATP binding cassette subfamily C member 12 | GTHTQLMQERGR | Oxidation (M),Deamidation (NQ) | 2 | 1429.67 | -1.47 | 87540 |
| G3TYG5 | ATPase 13A5 | EKGEFRAQSTEQHFFTR | Acetyl (K),Glu->pyro-Glu | 3 | 2121.00 | 0.08 | 99026 |
| G3U5X6 | ATPase family AAA domain containing 3A | MSWLFGINKGPK | Oxidation (M),Deamidation (NQ),Dioxidation (W) | 2 | 1425.70 | 0.83 | 208180 |
| G3SMP1 | ATP-dependent DNA helicase, EC 3.6.4.12 | LIDSWMGKGAAK | Oxidation (M),Kynurenine | 2 | 1295.65 | -1.52 | 418600 |
| G3TX50 | ATP-dependent RNA helicase DDX54 | YISSSYKR | Oxidation (Y) | 2 | 1018.51 | 1.12 | 800060 |
| G3TBN9 | Axin 2 | ANGQVSLPHFPRTHR | 2 Deamidation (NQ) | 3 | 1717.86 | -4.27 | 2864600 |
| G3SZM3 | BAI1 associated protein 3 | VSPAMCALKVSIMCTK | Acetyl (K),Oxidation (M),di-oxidation (M) | 2 | 1884.88 | 3.29 | 456230 |
| G3T4J1 | Basic leucine zipper and W2 domains 1 | KVSMDNRLMELFPANK | Oxidation (M),Deamidation (NQ) | 2 | 1908.94 | 0.18 | 195410 |
| G3SLW9 | Basonuclin 1 | NRHSANPNPRLHMPMNR | 2 di-oxidation (M) | 2 | 2104.98 | 3.04 | 145440 |
| G3SMM1 | Bestrophin | IRDSVDLQSLMTEMNRYR | Oxidation (M) | 2 | 2242.08 | -2.76 | 2183300 |
| G3UCX2 | BHLH domain-containing protein | ALTALTEQQHQKIIALQNGKR | 3 Deamidation (NQ) | 3 | 2363.28 | 1.01 | 483890 |
| G3T3T1 | Blood vessel epicardial substance | LSLCTQISMLEMR | Oxidation (M),Deamidation (NQ),di-oxidation (M) | 2 | 1629.74 | 0.07 | 1195100 |
| Q68J46 | Blue cone photoreceptor pigment | GCIMEMVCGK | 2 di-oxidation (M) | 2 | 1247.47 | 2.58 | 757820 |
| G3TD47 | Bromodomain adjacent to zinc finger domain 2B | QQAIMAAEEKR | Acetyl (K),di-oxidation (M) | 2 | 1347.65 | -0.13 | 183520 |
| G3T6C3 | Calcium binding protein 7 | DGNGFISK | Deamidation (NQ) | 1 | 837.39 | -0.83 | 955240 |
| G3U7J6 | Calcium voltage-gated channel subunit alpha1 H | PYYAEYSPTR | 2 Oxidation (Y) | 2 | 1277.56 | 0.37 | 275370 |
| G3UD24 | Calcyon neuron specific vesicular protein | ICTPLTLEMYYTEMDPDRHR | Trioxidation (C) | 3 | 2588.13 | 2.11 | 5931700 |
| G3TXT5 | Calcyphosine like | GLNDYAVVMEK | Deamidation (NQ),di-oxidation (M) | 2 | 1270.58 | 2.80 | 671540 |
| G3T480 | Calmodulin regulated spectrin associated protein family member 2 | NVLDSSCSSDFSSR | Deamidation (NQ),Trioxidation (C) | 2 | 1608.62 | 1.83 | 370180 |
| G3TYF4 | cAMP regulated phosphoprotein 19 | MKNKQLPTAAPDK | Oxidation (M),Deamidation (NQ) | 2 | 1457.75 | -1.04 | 839800 |
| G3TBU3 | CAP-Gly domain containing linker protein 3 | KVHQVTMTQPK | Oxidation (M) | 2 | 1311.70 | 4.00 | 199160 |
| G3UFY1 | Carbohydrate sulfotransferase | TQREEPFNEHWER | 2 Deamidation (NQ),Oxidation (W) | 2 | 1774.75 | -1.35 | 227610 |
| G3SQM5 | Carbohydrate sulfotransferase, EC 2.8.2.- | QRKNATQEALR | Acetyl (K),2 Deamidation (NQ) | 3 | 1357.69 | 0.36 | 240290 |
| G3SVY6 | Cathepsin G | WNDIMLLQLETR | Deamidation (NQ),Dioxidation (W) | 2 | 1563.76 | -3.28 | 3090800 |
| G3TKF5 | CD163 molecule like 1 | MSSPAEEAWITCEDK | Oxidation (M),Kynurenine | 2 | 1772.72 | -3.72 | 30126000 |
| G3TJE4 | Cell division cycle associated 8 | AQSIQGKGRSK | Deamidation (NQ) | 2 | 1159.63 | 0.30 | 8024400 |
| G3T0X5 | Centromere protein K | LIKECEEIWKDMEECQNK | Acetyl (K),2 Deamidation (NQ) | 3 | 2425.05 | -1.13 | 1668400 |
| G3SZA4 | Centrosomal protein of 162 kDa | QALEVDLEKMK | Acetyl (K),Deamidation (NQ) | 2 | 1345.68 | 2.94 | 503540 |
| G3TUW5 | Charged multivesicular body protein 2A | GVTKAMGTMNRQLK | 2 di-oxidation (M) | 2 | 1597.79 | 2.39 | 1974300 |
| G3UJL3 | Charged multivesicular body protein 2B | NVIKEQNRELR | Deamidation (NQ) | 2 | 1398.76 | 2.77 | 371560 |
| G3SYQ0 | Chromo domain-containing protein | KVADNKAK | Deamidation (NQ) | 1 | 873.49 | 0.14 | 25872000 |
| G3SVF8 | Cilia and flagella associated protein 221 | EAIKQQFSQEKK | Acetyl (K),Deamidation (NQ) | 2 | 1505.77 | 0.38 | 330270 |
| G3T0V2 | Claspin | ENMEELLNLCSGK | Oxidation (M),2 Deamidation (NQ) | 2 | 1553.66 | -2.23 | 2379200 |
| G3SY79 | Coiled-coil domain containing 160 | ESISQLNEKER | Glu->pyro-Glu,Deamidation (NQ) | 2 | 1314.64 | 1.76 | 122070 |
| G3T471 | Coiled-coil domain containing 18 | EQSLQKSQEECIKLK | Trioxidation (C) | 2 | 1894.93 | 0.21 | 1099700 |
| G3SPR0 | Component of oligomeric Golgi complex 7 (Conserved oligomeric Golgi complex subunit 7) | QATKSLPR | Acetyl (K),Deamidation (NQ) | 2 | 942.51 | 0.74 | 134480 |
| G3T8X6 | Copine 4 | LQLYGPTNIAPIIQK | Deamidation (NQ),3-iodotyrosine | 2 | 1794.84 | 3.46 | 100880 |
| G3SWS3 | Copine 8 | MWAPDKCTALPPK | Acetyl (K),Oxidation (M) | 2 | 1571.75 | 1.49 | 448060 |
| G3TF56 | Cor1 domain-containing protein | LTVMIQQQQR | 3 Deamidation (NQ) | 2 | 1246.62 | -2.62 | 1054000 |
| G3T2S8 | Coronin | GLEVSKCEIAR | Acetyl (K),Trioxidation (C) | 2 | 1350.64 | 0.07 | 1786600 |
| G5E730 | Cramped chromatin regulator homolog 1 | SPRCTRNQATLR | Trioxidation (C) | 2 | 1506.73 | 0.72 | 147800 |
| G3TDW1 | CWC22 spliceosome associated protein homolog | LRMMQEQITDK | Oxidation (M),Deamidation (NQ) | 2 | 1408.67 | -0.56 | 568950 |
| G3TSP9 | Cyclin-T1 | MEGERKNNNK | Deamidation (NQ),di-oxidation (M) | 2 | 1251.55 | 0.05 | 237170 |
| G3UL67 | Cysteine and serine rich nuclear protein 3 | EEKLNSLKLK | Acetyl (K),Glu->pyro-Glu | 2 | 1224.71 | -3.60 | 474660 |
| G3TEI3 | Cytochrome P450 family 20 subfamily A member 1 | IEQLSYCRQVLCETVR | 2 Deamidation (NQ),Trioxidation (C) | 2 | 2102.96 | -3.44 | 229350 |
| G3TQH8 | Cytochrome P450 family 26 subfamily A member 1 | IEENIRSKICR | Acetyl (K) | 3 | 1458.76 | -4.23 | 801580 |
| G3SLQ2 | DENN domain containing 2B | NTSITHGAGGTKASQGTLSR | Acetyl (K),Deamidation (NQ) | 3 | 1985.98 | -0.92 | 280270 |
| G3TU50 | DENN domain containing 4B | SPMDSLLR | Oxidation (M) | 1 | 933.46 | -3.37 | 81090 |
| G3T936 | DEP domain-containing protein | LMQGYQIIVQPK | 2 Deamidation (NQ) | 2 | 1418.75 | 3.33 | 93308 |
| G3SYN1 | Derlin | YCRMLEEGSFRGR | Oxidation (M),Trioxidation (C) | 2 | 1723.74 | 1.35 | 210320 |
| G3SZ07 | DIS3-like exonuclease 1 | KMEIKENLFSNK | Oxidation (M),Deamidation (NQ) | 2 | 1496.75 | 2.64 | 5740500 |
| G3SU95 | DNA helicase, EC 3.6.4.12 | MYSYKALDK | Oxidation (M),Dopaquinone (Y) | 2 | 1147.52 | 2.77 | 60209 |
| G3SSD0 | DNA polymerase epsilon subunit (DNA polymerase II subunit 2) | ERYTILHQRTHR | 3-iodotyrosine | 2 | 1734.76 | 1.39 | 512540 |
| G3T573 | DNA polymerase subunit gamma-1, EC 2.7.7.7 (Mitochondrial DNA polymerase catalytic subunit) | AQQMYAVTK | Oxidation (M),Dioxidation (Y) | 2 | 1086.50 | -4.27 | 1070700 |
| G3UII9 | DNA replication ATP-dependent helicase/nuclease | PDPEAGLLLYLKTGQMYPVPAK | Oxidation (Y) | 3 | 2416.27 | -3.79 | 559310 |
| G5E707 | DNA topoisomerase 2 | QTWMNNMMK | 2 di-oxidation (M) | 2 | 1246.48 | -3.52 | 4270400 |
| G3SPD8 | DNA-directed RNA polymerase, EC 2.7.7.6 | QREHQVQALQR | Deamidation (NQ) | 3 | 1392.72 | 0.65 | 425770 |
| G3SSQ7 | DnaJ heat shock protein family (Hsp40) member B1 | MGKDYYQILGLAR | Oxidation (Y),Dioxidation (Y) | 2 | 1574.78 | -4.33 | 211550 |
| G3TH44 | DnaJ heat shock protein family (Hsp40) member C11 | AIYDIYGK | 2 Dopaquinone (Y) | 2 | 969.44 | -3.04 | 202880 |
| G5E6W7 | DnaJ homolog subfamily C member 10 | GVNSYPSLLIFR | Deamidation (NQ),Dioxidation (Y) | 2 | 1397.72 | -1.26 | 2803100 |
| G3UDL0 | Dpy-19 like 4 | RSAEDIYKILTSYK | Oxidation (Y) | 2 | 1701.89 | 2.79 | 227720 |
| G3T869 | DUF4706 domain-containing protein | VVPACHRQEPALK | Deamidation (NQ),Trioxidation (C) | 2 | 1552.77 | 4.16 | 1937500 |
| G3T342 | DUF4709 domain-containing protein | QLLQLQFEDR | Deamidation (NQ) | 2 | 1289.66 | 1.13 | 340100 |
| G3T3T9 | Dynein axonemal heavy chain 6 | LQKYINNPDFVPEK | Dopaquinone (Y) | 3 | 1717.87 | -4.24 | 2561300 |
| G3SLK0 | Dynein axonemal intermediate chain 2 | GVNHVEGGWPK | Deamidation (NQ),Oxidation (W) | 2 | 1195.56 | -2.55 | 4895900 |
| G3T497 | Dystonin | QSSINAMDEKVKK | Oxidation (M),Deamidation (NQ) | 2 | 1493.74 | -4.47 | 111830 |
| G3UAA1 | Dystonin | CENFTTCWR | Dioxidation (W) | 2 | 1304.49 | -2.06 | 40438000 |
| G3UAH9 | Early growth response protein | ALNNTYQSQLIK | Deamidation (NQ) | 3 | 1392.72 | 0.27 | 212770 |
| G3TQJ4 | EBF transcription factor 2 | SLGAEMDSVRSWVR | Oxidation (M),Kynurenine | 2 | 1611.77 | 2.41 | 117320 |
| G3T5Y2 | EF-hand calcium binding domain 12 | DNFLVYLQCWK | 2 Deamidation (NQ),Oxidation (W) | 2 | 1502.68 | -2.32 | 10673000 |
| G3T3B9 | EF-hand calcium binding domain 6 | ESGNEMNQSR | Glu->pyro-Glu,2 Deamidation (NQ) | 2 | 1134.42 | -2.94 | 239730 |
| G3T8I3 | EF-hand domain-containing protein | NWLVTCIQEILK | Deamidation (NQ),Oxolactone | 2 | 1530.78 | 2.76 | 3175200 |
| G3T7W3 | EH domain containing 1 | LAKVHAYIISSLKK | Dopaquinone (Y) | 2 | 1583.94 | -3.25 | 89584 |
| Q7YSN2 | Elongation factor 1 alpha | LPLQDVYK | Unmodified | 2 | 974.54 | -0.40 | 577280 |
| G3UL43 | EPS8 like 2 | AWLEAKAFSPR | Dioxidation (W) | 3 | 1306.67 | 3.23 | 4080900 |
| G3TES0 | ER-alpha (Estradiol receptor) (Estrogen receptor) (Nuclear receptor subfamily 3 group A member 1) | AGLSLQQQHR | 3 Deamidation (NQ) | 2 | 1139.56 | -1.06 | 237290 |
| G3TYT1 | Establishment of sister chromatid cohesion N-acetyltransferase 1 | SRAVQGQVQALKQSLPPTR | 2 Deamidation (NQ) | 2 | 2065.13 | 0.42 | 502240 |
| G3U235 | Eukaryotic translation initiation factor 3 subunit A | LESLNIQR | Deamidation (NQ) | 2 | 972.52 | -0.69 | 266150000 |
| G3U1L5 | Eukaryotic translation initiation factor 4 gamma 1 | AEKAWKPSSK | Dioxidation (W) | 2 | 1162.60 | 0.63 | 556550 |
| G3UCT3 | Exonuclease 5 | SISLQKWKR | Deamidation (NQ),Oxolactone | 2 | 1159.63 | -2.73 | 8019200 |
| G3SQ03 | Extracellular matrix protein 2 | EDEAMQPQVRGK | Oxidation (M),Glu->pyro-Glu | 2 | 1384.64 | 3.89 | 544330 |
| G3SN12 | FA complementation group M | STCLMSLAAMNSQR | Oxidation (M),Deamidation (NQ) | 2 | 1585.69 | 3.30 | 113130 |
| G3T8F5 | Family with sequence similarity 111 member A | LVINSTTMKEHK | di-oxidation (M) | 2 | 1431.74 | -1.79 | 1408100 |
| G3STV7 | Family with sequence similarity 135 member B | IEMCKTALK | Acetyl (K),di-oxidation (M) | 2 | 1166.57 | -3.02 | 1558900 |
| G3TB35 | Family with sequence similarity 160 member B2 | AEYPPGMR | Oxidation (M),Oxidation (Y) | 2 | 951.41 | -0.52 | 228040 |
| G3SL62 | Family with sequence similarity 172 member A | EMMQLYIR | Oxidation (M),Deamidation (NQ),Dioxidation (Y) | 2 | 1131.49 | 0.80 | 3058500 |
| G3T8B1 | Family with sequence similarity 83 member B | TNNPPSNWK | 2 Deamidation (NQ) | 1 | 1058.47 | 0.00 | 755730 |
| G3SM28 | F-box protein 28 | LSLLNMTFMK | 2 Oxidation (M) | 2 | 1228.62 | 1.78 | 111770 |
| G3SUR4 | FCH and mu domain containing endocytic adaptor 1 | PDVTQNNTVEPSR | 2 Deamidation (NQ) | 2 | 1457.66 | -2.16 | 207520 |
| G3TUG3 | FH2 domain-containing protein | RTMTTVLDPK | Oxidation (M) | 2 | 1176.62 | -3.84 | 543950 |
| G3SWN2 | Fibroblast activation protein alpha | DDNLEHYKNSTVMAR | Deamidation (NQ),Oxidation (Y) | 2 | 1808.80 | 4.27 | 332760 |
| G3TIC3 | Fibroblast growth factor receptor substrate 3 | YGYDSNLFSFESGRR | Dioxidation (Y) | 2 | 1828.80 | 0.13 | 107600 |
| G3SXW9 | Filamin B | TGRYSIGVTYGGDDIPYSPYR | 2 Dopaquinone (Y),3-iodotyrosine,di-iodotyrosine | 4 | 2741.76 | -2.03 | 692080 |
| G3TCZ0 | FKBP prolyl isomerase like | NSQKNLDSTSQIR | Deamidation (NQ) | 2 | 1490.73 | 4.44 | 5220600 |
| G3ULV9 | Forkhead box O3 | VQNEGTGKSSWWIINPDGGKSGK | Acetyl (K),Deamidation (NQ) | 2 | 2487.20 | -1.52 | 366810 |
| G3T420 | Frataxin intermediate form, EC 1.16.3.1 (Frataxin mature form) (Frataxin, mitochondrial) | QSVHLMNLR | 2 Deamidation (NQ),di-oxidation (M) | 2 | 1130.54 | -3.50 | 294260 |
| G3TXI8 | Fucosyltransferase 9 | KDFTVNLPR | Deamidation (NQ) | 2 | 1089.58 | 2.41 | 906910 |
| G3U5I6 | FUS RNA binding protein | SKAGVCDMLKGQTR | Deamidation (NQ),di-oxidation (M) | 2 | 1582.74 | 4.27 | 101630 |
| G3TND4 | FYVE, RhoGEF and PH domain containing 2 | LQDLWEVYQR | Oxidation (W) | 2 | 1364.67 | 1.35 | 1404600 |
| G3SVW5 | G protein-coupled receptor kinase, EC 2.7.11.- | MYACKKLDK | Acetyl (K),Trioxidation (C),di-oxidation (M) | 2 | 1277.56 | 3.96 | 1480800 |
| G3U6B1 | G_PROTEIN_RECEP_F1_2 domain-containing protein | GLQSQAPPVINDQHPVQPDAL | Deamidation (NQ) | 3 | 2224.11 | -0.27 | 5108200 |
| G3TVW9 | G_PROTEIN_RECEP_F3_4 domain-containing protein (GLOD4) | SLNYWSNLLGMKIYEKDEQK | Oxidation (M) | 3 | 2474.22 | -0.24 | 1586000 |
| G3TV72 | G_PROTEIN_RECEP_F3_4 domain-containing protein (GRM4) | RGCSWWWAR | Oxolactone,Oxidation (W) | 2 | 1293.55 | 4.26 | 1134100 |
| G3U4M8 | Gametogenetin-binding protein 2 | AEPEFAGGYERR | 3-iodotyrosine | 2 | 1506.54 | 1.59 | 354520 |
| G3SUF9 | Gastric inhibitory polypeptide (Glucose-dependent insulinotropic polypeptide) | QQDFVNWLLAQK | 2 Deamidation (NQ),Oxolactone | 2 | 1504.72 | -1.47 | 1138800 |
| G3T3W8 | Gastrulation brain homeobox 2 | AGNANSKTGEPSR | 2 Deamidation (NQ) | 2 | 1289.58 | 3.66 | 351030 |
| G3TTE3 | Geranylgeranyl transferase type-2 subunit alpha | ETLQYFQTLK | 2 Deamidation (NQ) | 2 | 1271.63 | -1.33 | 468660 |
| G3TMK7 | GLI family zinc finger 1 | THSNEKPYVCK | Deamidation (NQ),Dioxidation (Y) | 2 | 1394.61 | -3.72 | 59008 |
| G3U0V7 | GLI family zinc finger 2 | TSPNSLVAYINNSR | Deamidation (NQ) | 2 | 1535.76 | 1.31 | 241730 |
| G3TJB1 | Glucosamine (UDP-N-acetyl)-2-epimerase/N-acetylmannosamine kinase | LIQEWNSVDLR | 2 Deamidation (NQ),Oxolactone | 2 | 1387.66 | 0.64 | 199420 |
| G3SW15 | Glutamate receptor | VGKWENQTLSLR | Kynurenine | 2 | 1433.76 | 3.82 | 78540 |
| G3T9K6 | Glutaredoxin-like protein | QLLKLEQQGAR | 3 Deamidation (NQ) | 2 | 1285.69 | 0.03 | 631830 |
| C6FWG3 | Glyceraldehyde dehydrogenase | IVHNASCTTNCLAPLAK | Deamidation (NQ) | 3 | 1869.91 | 2.03 | 155780 |
| G3T4Z0 | Glyco_tran_10_N domain-containing protein | LVAWVVSNWQPNSVR | 2 Deamidation (NQ),Oxidation (W) | 2 | 1771.89 | -4.31 | 371060 |
| G3TTQ7 | Glycogen synthase kinase-3 beta | GEPNVSYICSRYYR | Dioxidation (Y) | 2 | 1794.80 | -4.01 | 562110 |
| G3TD69 | Glycoprotein M6B | KPAMETAAEENTEQSQERK | Deamidation (NQ),di-oxidation (M) | 2 | 2208.98 | 2.68 | 275340 |
| G3T597 | Glycosyltransferase-like domain-containing protein 1 | GKMAPFSWAALHGK | Oxidation (M),Oxolactone | 2 | 1529.74 | 2.65 | 543300 |
| G3U1Y8 | Glycylpeptide N-tetradecanoyltransferase | NMTMQRTMK | 2 Oxidation (M),Deamidation (NQ) | 2 | 1172.50 | 2.25 | 514540 |
| G3TM14 | GOLD domain-containing protein | GTGAFKIQQWDMGR | Deamidation (NQ),Oxolactone | 2 | 1608.74 | 3.12 | 180710 |
| G3TE09 | Golgin B1 | TEGDPEDNGQKEMK | Acetyl (K),Deamidation (NQ),di-oxidation (M) | 2 | 1651.65 | -3.58 | 1331400 |
| G3UIM5 | Gonadoliberin | AQHWSHGWYPGGKR | Deamidation (NQ),Oxolactone | 2 | 1680.75 | 3.55 | 129150 |
| G3TZG3 | GPRIN family member 3 | ENPTPKRFEQEQLR | 2 Deamidation (NQ) | 2 | 1772.87 | -2.39 | 134850 |
| G3U6V0 | HABP4_PAI-RBP1 domain-containing protein | TLDEWKAIQDK | Kynurenine | 2 | 1349.68 | -1.24 | 128530 |
| G3SWT6 | HAUS augmin like complex subunit 7 | LEILEWMCVR | Trioxidation (C),di-oxidation (M) | 2 | 1427.64 | -0.22 | 98497 |
| G3SX38 | Hdc homolog, cell cycle regulator | KDTDWYQVK | Oxidation (Y) | 2 | 1197.57 | -2.98 | 255140 |
| G3SLN5 | Heat shock protein family A (Hsp70) member 4 like | LGTVYEK | Dioxidation (Y) | 2 | 840.42 | 0.65 | 239220 |
| G3TQA5 | Hepatocyte growth factor | VGYCSQIPKCDMSSGR | Trioxidation (C),di-oxidation (M) | 2 | 1923.78 | 3.69 | 1391400 |
| G3T9U1 | Hephaestin like 1 | PSWLGFLGPILR | Dioxidation (W) | 2 | 1386.77 | 0.67 | 175000 |
| G3TL50 | Histidine--tRNA ligase | TTETQVFVATPQKNFLQER | 2 Deamidation (NQ) | 3 | 2238.12 | 3.12 | 1033700 |
| G3ULK4 | Histone acetyltransferase (EP300) | QIISHWKNCTR | Acetyl (K),Trioxidation (C) | 2 | 1531.72 | 1.18 | 875620 |
| G3TEZ7 | Histone acetyltransferase (KAT6B) | QSLNGSYMR | di-oxidation (M) | 2 | 1086.48 | -2.58 | 140230 |
| G3SMA5 | Homeobox A13 | VINKLKTTS | 2 Acetyl (K),Deamidation (NQ) | 2 | 1087.61 | -0.71 | 1204500 |
| G3SQP7 | Homeobox protein cut-like | ISNSDLSGSARR | Deamidation (NQ) | 2 | 1262.62 | -2.60 | 314960 |
| G3TQI3 | Hook microtubule tethering protein 2 | MGQPEGEGAMGLTAKK | Oxidation (M) | 2 | 1619.76 | -1.35 | 652100 |
| G3TBF3 | IF rod domain-containing protein | EQIKNLNSR | 2 Deamidation (NQ) | 2 | 1102.56 | -0.13 | 2626900 |
| G3UHP5 | Immunoglobulin superfamily member 3 | QEDSGKYNCR | Deamidation (NQ),Trioxidation (C) | 2 | 1304.49 | -2.14 | 40446000 |
| G3TPV4 | Inositol 1,4,5-trisphosphate receptor type 3 | SKFEENEDIVVMETKLK | Acetyl (K),Oxidation (M),Deamidation (NQ) | 2 | 2097.02 | 0.73 | 102170 |
| G3T4Y6 | Integrator complex subunit 12 (PHD finger protein 22) | MAQKTQKPAQK | Acetyl (K),2 Deamidation (NQ) | 2 | 1301.66 | 2.55 | 128220 |
| G3SPI0 | Integrin alpha FG-GAP repeat containing 2 | MESTNLLKLLETEPEYRR | Oxidation (M),Deamidation (NQ) | 3 | 2238.12 | 3.64 | 2635000 |
| G3TRJ9 | Iroquois homeobox 3 | ENKMTWAPR | Glu->pyro-Glu,Oxolactone | 2 | 1127.52 | 2.08 | 16429000 |
| G3TAH7 | Isoleucyl-tRNA synthetase, EC 6.1.1.5 | FLIQNVLR | Deamidation (NQ) | 2 | 1002.59 | 0.34 | 1626200 |
| G3TSN2 | IZUMO1 receptor, JUNO | LLNVCMNTK | Oxidation (M),Deamidation (NQ),Trioxidation (C) | 2 | 1156.51 | -2.56 | 185990 |
| G3THE0 | Kelch like family member 20 | TNQWSPVVAMTSR | Deamidation (NQ),Oxidation (W) | 2 | 1492.70 | -4.42 | 833670 |
| G3SZ72 | Kelch-like protein 3 | WDQIAELPSRR | Deamidation (NQ),Oxolactone | 2 | 1384.67 | -2.34 | 12392000 |
| G3TX86 | Kinesin family member 13B | LSPEKQNYR | Deamidation (NQ),Dioxidation (Y) | 2 | 1166.56 | -0.56 | 854840 |
| G3TPU3 | Kinesin-like protein (KIF 19) | LLKDSLGGNSR | Deamidation (NQ) | 2 | 1159.62 | 0.74 | 710660 |
| G3SM45 | Kinesin-like protein (KIF 17) | FLVRASYLEIYNEDVR | Deamidation (NQ),Oxidation (Y) | 2 | 2003.00 | -0.75 | 309470 |
| G3T5J0 | Lactoylglutathione lyase, EC 4.4.1.5 (Glyoxalase I) | KSLDFYTR | Dioxidation (Y) | 2 | 1060.52 | -0.37 | 82095 |
| G3TZL3 | Large tumor suppressor kinase 1 | KKQLENEMMR | Oxidation (M),Deamidation (NQ) | 3 | 1322.63 | 3.53 | 399660 |
| C6FWG7 | Latency-associated peptide (Transforming growth factor beta-1) | LQVEIKGLNNNHR | 2 Deamidation (NQ) | 2 | 1535.81 | 1.44 | 271060 |
| G3TD01 | LDL receptor related protein 1B | LYWADENHIEFSNMDGSHR | Oxidation (M),Oxolactone | 2 | 2349.97 | -2.07 | 5953000 |
| G3TMC9 | Lecithin retinol acyltransferase | YGTRISPQADK | Deamidation (NQ),3-iodotyrosine | 2 | 1361.51 | 0.48 | 1388300 |
| G3U518 | Leukocyte receptor cluster member 8 | KKMAVLECEDPER | Oxidation (M) | 2 | 1619.76 | -1.69 | 595290 |
| G3SZP8 | LIM homeobox 9 | PALCAGCGGKISDR | 2 Trioxidation (C) | 2 | 1556.66 | 2.25 | 1179000 |
| G3SQF4 | Lin-9 DREAM MuvB core complex component | NTGSNVEMPFRNSKR | Oxidation (M),Deamidation (NQ) | 2 | 1752.82 | 4.02 | 825340 |
| G3UD48 | Lipocln_cytosolic_FA-bd_dom domain-containing protein | SLNNNEEAIKKFK | 2 Deamidation (NQ) | 2 | 1535.78 | -2.31 | 202590 |
| G3SL13 | L-serine-phosphatidylethanolamine phosphatidyltransferase, EC 2.7.8.29 | MINEQQVEDITIDFFYR | Oxidation (M),Deamidation (NQ),Dioxidation (Y) | 2 | 2208.99 | -0.63 | 292910 |
| G3SXQ8 | Lysozyme, EC 3.2.1.17 | STDYGIFQINSR | Unmodified | 2 | 1399.67 | 0.53 | 1127200 |
| G3TPC7 | Maestro heat like repeat family member 1 | NCSHAATVMINCLLK | Deamidation (NQ),Trioxidation (C) | 2 | 1779.80 | -1.55 | 520080 |
| D6C6M1 | Matrix extracellular phosphoglycoprotein | TKQGCVEEQKITYK | Deamidation (NQ),Dioxidation (Y) | 2 | 1743.83 | 1.92 | 217830 |
| G3U0T6 | Matrix remodeling associated 5 | DLCWTSPTTAQPQR | Deamidation (NQ),Oxolactone | 2 | 1674.73 | -2.26 | 591430 |
| G3THX8 | MCF.2 cell line derived transforming sequence | SLNDSMHQIAITGYIGNLNDLGK | Deamidation (NQ),Oxidation (Y) | 3 | 2490.21 | 0.19 | 259820 |
| G3UCM5 | Mediator complex subunit 25 | MLPCQAYVNQGENLR | 2 Deamidation (NQ),Trioxidation (C) | 2 | 1841.79 | -0.21 | 1581000 |
| G3TL40 | Mediator of RNA polymerase II transcription subunit 17 | SVTENQENKLQR | Deamidation (NQ) | 2 | 1445.71 | 0.98 | 337120 |
| G3UH75 | Methyl-CpG binding domain protein 1 | SRGCGVCRGCQTR | Deamidation (NQ) | 2 | 1553.66 | -1.94 | 2167000 |
| G3T749 | Methyltransferase like 7A | LLPYFLMR | di-oxidation (M) | 2 | 1083.58 | 3.55 | 132540 |
| G3UJ89 | MIA SH3 domain ER export factor 3 | AELEDECKTLKQK | Trioxidation (C) | 2 | 1638.78 | -0.86 | 110440 |
| G3SS28 | Microtubule-associated protein | LDFKDRVQSK | Deamidation (NQ) | 2 | 1235.65 | -1.32 | 158530 |
| G3SN14 | MIS18 binding protein 1 | NNGGIVWGNVR | Deamidation (NQ),Oxidation (W) | 2 | 1201.58 | -3.29 | 421380 |
| G3TXN0 | Mitogen-activated protein kinase kinase kinase kinase 1 | TTWVYSINNVLMSLSGK | Deamidation (NQ),Dioxidation (W) | 2 | 1944.95 | -2.25 | 1662400 |
| G3TC11 | Mitogen-activated protein kinase kinase kinase kinase 5, EC 2.7.11.1 | ETLQGLAYLHTK | Glu->pyro-Glu,Deamidation (NQ) | 2 | 1355.71 | 0.60 | 2085300 |
| G3SPQ0 | MLLT3 super elongation complex subunit | PDSNLLTITSGQQDKK | 2 Deamidation (NQ) | 2 | 1745.87 | 0.93 | 151750 |
| G3T8U6 | MORN repeat-containing protein 5 | YIGEYIDGRMEGK | Oxidation (Y),Dopaquinone (Y) | 2 | 1559.69 | 0.74 | 267520 |
| G3TCQ4 | Mov10 like RISC complex RNA helicase 1 | NVDLTDVK | Deamidation (NQ) | 1 | 903.45 | -0.65 | 313580 |
| R4TVZ7 | Multifunctional fusion protein | LQISQQHFNK | 3 Deamidation (NQ) | 2 | 1244.60 | 3.81 | 91842 |
| G3TA55 | Multimerin 2 | EEELQTALTDIR | Deamidation (NQ) | 3 | 1417.69 | -2.25 | 116110 |
| G5E6Y9 | MutS homolog 3 | ALENDGPVKKR | Deamidation (NQ) | 2 | 1226.66 | -0.32 | 1644800 |
| G3SV10 | MYB proto-oncogene like 1 | VEQEGYLQDGIKSERSSSK | Deamidation (NQ),Oxidation (Y) | 2 | 2156.02 | -1.26 | 185330 |
| G3U1B9 | Myb-like domain-containing protein | KEIGVQNHDK | Deamidation (NQ) | 2 | 1167.59 | 0.47 | 1434800 |
| G3T0H3 | Myoferlin | TMKFIVWRR | Kynurenine | 2 | 1239.69 | 2.15 | 1688600 |
| G3U4D0 | Myosin binding protein C, cardiac | VGQYLQLRDSYDRTSK | Deamidation (NQ),Oxidation (Y),3-iodotyrosine | 2 | 2070.85 | -3.47 | 155350 |
| G3U2K0 | Myosin heavy chain 10 | AMVNKDDIQK | Acetyl (K),Deamidation (NQ),di-oxidation (M) | 2 | 1235.57 | -1.83 | 42841 |
| G3SR79 | Myosin heavy chain 7B | QAMEAQAATR | Oxidation (M),Deamidation (NQ) | 2 | 1092.49 | 0.17 | 191890 |
| G3UC08 | Myosin light chain kinase | ASMSAELSIQGLDNTNR | 2 Deamidation (NQ) | 2 | 1807.83 | -1.77 | 532760 |
| G3U6C3 | N(alpha)-acetyltransferase 80, NatH catalytic subunit | AGSSPQSLLETQYR | Deamidation (NQ),Oxidation (Y) | 2 | 1552.74 | -0.11 | 338360 |
| G3TB42 | Na(+)-dependent phosphate cotransporter 2B | VCCLLCGCPK | 2 Trioxidation (C) | 2 | 1361.51 | 2.62 | 1064600 |
| G3UDE1 | NCCRP1, F-box associated domain containing | VTDSSVSVQLRE | Unmodified | 2 | 1318.67 | -0.59 | 1484000 |
| G3TDQ7 | NCK associated protein 5 like | VFDLERQNQMLSALFQQK | Oxidation (M),Deamidation (NQ) | 2 | 2211.10 | -2.75 | 420270 |
| G3T4G5 | Nebulin | LVWSMNVAK | Oxidation (M),Deamidation (NQ),Oxolactone | 2 | 1077.52 | -2.74 | 389990 |
| G3SZM2 | Negative regulator of ubiquitin like proteins 1 | ALMLAMGYHEKGR | di-oxidation (M) | 2 | 1507.73 | 3.39 | 264420 |
| G3T6H4 | Nei like DNA glycosylase 2 | EQCPVGHQIMK | Glu->pyro-Glu,Deamidation (NQ) | 2 | 1308.60 | -2.31 | 230940 |
| G3UMA4 | Netrin 5 | VLAIEAAGPTWWR | Oxidation (W) | 2 | 1484.78 | 3.53 | 990050 |
| G3TSK2 | Neuregulin 2 | QNMCPAHQNR | 2 Deamidation (NQ),Trioxidation (C) | 2 | 1304.49 | 0.85 | 39952000 |
| G3SLM4 | Neuronal vesicle trafficking associated 1 | MVKLGNSFAEKGTK | Oxidation (M),Deamidation (NQ) | 2 | 1525.78 | 4.26 | 195640 |
| G3UBA2 | Non-specific serine/threonine protein kinase (RPS6KA2) | QDVHLVKGAMAATYFALNR | 2 Deamidation (NQ),Dopaquinone (Y) | 2 | 2120.04 | -1.22 | 555350 |
| G3TL75 | Non-specific serine/threonine protein kinase (SMG1) | RWQQREAALQAQK | Deamidation (NQ),Oxolactone | 2 | 1626.82 | -0.64 | 661170 |
| G3SPL7 | Non-specific serine/threonine protein kinase (TRPM7) | NTSSSTPQLRK | Deamidation (NQ) | 2 | 1218.62 | 2.31 | 5951400 |
| G3SNM7 | NOP2/Sun RNA methyltransferase 6 | EVTSYQPLQRKLFSVAVQLLK | Deamidation (NQ),Dopaquinone (Y) | 3 | 2461.36 | -1.04 | 409470 |
| G3TE67 | Nuclear pore complex protein Nup93 | EFDMILGKLENDGSR | Acetyl (K),Deamidation (NQ),di-oxidation (M) | 2 | 1797.81 | -3.83 | 668480 |
| G3TCT7 | Nuclear receptor coactivator 7 | LIEYYLNK | 2 Dopaquinone (Y) | 2 | 1082.53 | -3.95 | 424840 |
| G3SZU0 | Nucleobindin 2 | VYDPKNEEDDMIEMEEERLR | Acetyl (K),di-oxidation (M) | 3 | 2613.12 | -4.26 | 637060 |
| G3TCW0 | Nucleolar and coiled-body phosphoprotein 1 | TLQKAAGTVTPSKPASK | Deamidation (NQ) | 2 | 1684.94 | 0.44 | 1485600 |
| G3T3X7 | Nucleolar protein 9 | GIDMEKR | Oxidation (M) | 2 | 863.42 | -3.65 | 148980 |
| G3T9C6 | Nucleoporin 43 | DRIEITSLLPNR | Deamidation (NQ) | 2 | 1426.78 | 0.39 | 7680800 |
| G3TA95 | O-acyltransferase | HCPLPQTTFWGLVTPR | Oxolactone | 3 | 1922.95 | 2.90 | 315310 |
| G3T2R5 | Olfactomedin like 2B | EDMEEVQTEMNK | Glu->pyro-Glu,2 Deamidation (NQ) | 2 | 1465.56 | -0.02 | 969680 |
| G3UAW0 | Olfactory receptor | SLYNMLNLIN | 2 Deamidation (NQ),Dopaquinone (Y) | 2 | 1209.56 | 3.53 | 2142400 |
| G3SNV5 | ORC ubiquitin ligase 1 | YIEELESQVAQLK | Dopaquinone (Y) | 2 | 1562.78 | -3.80 | 1677400 |
| G5E7C5 | Oxysterol-binding protein | KTTESGSDQSEPR | Acetyl (K),Deamidation (NQ) | 2 | 1463.64 | -2.59 | 3317000 |
| G3STK0 | P2X purinoceptor | SWTIGIINR | Deamidation (NQ) | 2 | 1059.57 | -3.00 | 777090 |
| G3TDU9 | Palmitoyltransferase, EC 2.3.1.225 (ZDHHC13) | CKELVEAGYDVR | Trioxidation (C) | 2 | 1485.68 | 2.27 | 629530 |
| G3SYR1 | Palmitoyltransferase, EC 2.3.1.225 (ZDHHC17) | NVWMINHLQEARQAK | Oxolactone | 3 | 1850.92 | 0.01 | 8180900 |
| G3UFA7 | PDZ and LIM domain 7 | QRLMENTEDWR | Oxidation (M),Oxolactone | 2 | 1506.65 | -1.38 | 376890 |
| G3UGQ9 | Penta-EF-hand domain containing 1 | FIQVCTQLQVLTEAFREK | 3 Deamidation (NQ) | 3 | 2212.11 | -2.37 | 1124600 |
| G3TU08 | Peptidylprolyl isomerase | LEQSTIVK | Unmodified | 2 | 916.52 | -0.97 | 216250 |
| G3UL63 | Pericentriolar material 1 | LRQMLNELMR | Oxidation (M) | 2 | 1318.68 | 3.68 | 77318 |
| G3TZP0 | Phosphoinositide 5-phosphatase | TGMGGATGNKGAVAIR | Deamidation (NQ),di-oxidation (M) | 2 | 1492.73 | -2.64 | 137690 |
| G3TV81 | Phosphoinositide phospholipase C | LTDPVPNPNPHESK | Deamidation (NQ) | 2 | 1544.75 | -3.97 | 1422800 |
| G3SQE7 | Phospholipase A2 group XV | RAPNENGPYFLALR | 3-iodotyrosine | 2 | 1742.74 | -3.60 | 227100 |
| G3UJ92 | Phospholipid-transporting ATPase (ATP8B1) | TEEERQIRTQSK | Deamidation (NQ) | 2 | 1504.75 | 3.77 | 3275300 |
| G3TAZ5 | Phospholipid-transporting ATPase (ATP10A) | CTVSGIEYSHDANARR | Oxidation (Y) | 2 | 1850.83 | -1.12 | 927910 |
| G3U978 | Phosphoseryl-tRNA kinase | AEFLEDLRQGSNK | Deamidation (NQ) | 2 | 1506.73 | 0.05 | 1495400 |
| G3T1S5 | Piwi like RNA-mediated gene silencing 4 | LSMYQIGR | Oxidation (M),Deamidation (NQ),Dioxidation (Y) | 2 | 1015.46 | -0.09 | 525690 |
| G3T547 | Plasminogen activator, EC 3.4.21.68 | QYKQPQFR | 2 Deamidation (NQ) | 2 | 1095.53 | -2.86 | 237390 |
| G3TS83 | Plexin C1 | ENEQLNCNFENITR | Glu->pyro-Glu,2 Deamidation (NQ) | 2 | 1763.74 | 0.38 | 237150 |
| G3SN10 | Plexin domain containing 2 | NLDSLKAVDTNR | Acetyl (K) | 2 | 1386.71 | -0.18 | 1709200 |
| G3TMI0 | Poly [ADP-ribose] polymerase | AILEKAGPELQEELNK | Deamidation (NQ) | 2 | 1781.94 | -3.68 | 453710 |
| G3SU39 | Poly [ADP-ribose] polymerase (PARP8) | GIYFMGMCSR | 2 di-oxidation (M) | 2 | 1284.49 | 1.70 | 199350 |
| G3TK42 | Potassium two pore domain channel subfamily K member 10 | HGQGASEDNIINKFGSSSK | 3 Deamidation (NQ) | 2 | 1977.89 | -0.40 | 265830 |
| G3SYH6 | Potassium voltage-gated channel subfamily B member 1 (Voltage-gated potassium channel subunit Kv2.1) | NGSIVSMNMK | 2 Oxidation (M),Deamidation (NQ) | 2 | 1112.48 | -3.21 | 268790 |
| G3T270 | PPR_long domain-containing protein | KMNITISQNIYRGIR | Oxidation (M),Deamidation (NQ),Oxidation (Y) | 2 | 1838.97 | -2.22 | 168990 |
| G3UKH3 | PR/SET domain 5 | PFQCEECKALFR | Acetyl (K),Trioxidation (C) | 2 | 1673.72 | 0.02 | 9965100 |
| G3TY10 | Pre-mRNA-splicing factor 38B | LTRKQVMGLITHTDSPYIR | Oxidation (M),Deamidation (NQ) | 3 | 2245.19 | 4.29 | 1054700 |
| G3TKX0 | Presequence translocase associated motor 16 | VNDKSVGGSFYLQSK | Deamidation (NQ),3-iodotyrosine | 2 | 1754.70 | 2.96 | 257210 |
| G3TF58 | Probable RNA-binding protein 46 (RNA-binding motif protein 46) | EVDEETMQRVK | Glu->pyro-Glu,Deamidation (NQ) | 2 | 1345.62 | 3.84 | 347730 |
| G3T352 | Progesterone immunomodulatory binding factor 1 | QNMELSVRCAHEEDRLER | Oxidation (M) | 4 | 2287.04 | -0.53 | 642960 |
| G3TIH2 | Proline rich coiled-coil 2C | KYATLSLFNTYKGK | Dioxidation (Y),Dopaquinone (Y) | 2 | 1678.86 | 4.33 | 1279100 |
| G3TA84 | Proteasome subunit beta | LAAIAESGVER | Unmodified | 2 | 1114.60 | 0.69 | 421730 |
| G3UNJ6 | Protein kinase domain-containing protein | TKWSEEDQESGQKR | Oxidation (W) | 3 | 1722.78 | -3.28 | 893030 |
| G3TSU2 | Protein phosphatase 2 regulatory subunit B''alpha | LKSFQQAQIQNK | Acetyl (K),2 Deamidation (NQ) | 2 | 1475.76 | -0.89 | 3471800 |
| G3UJY5 | Protein phosphatase 6 regulatory subunit 2 | QEVLHWLNEEK | Oxolactone | 3 | 1437.69 | 2.13 | 448610 |
| G3UFE4 | Protein phosphatase, Mg2+/Mn2+ dependent 1J | ELGQRMLYR | Dopaquinone (Y) | 2 | 1178.59 | 4.00 | 95634 |
| G3UB79 | Protein S100 | LQDAEIAR | Deamidation (NQ) | 2 | 915.47 | 0.13 | 336140 |
| G3SNZ5 | Pseudouridine synthase 7 like | SLMPEFKVRER | Oxidation (M) | 2 | 1406.73 | -1.39 | 104440 |
| G3SSZ8 | Purine nucleoside phosphorylase, EC 2.4.2.1 (Inosine-guanosine phosphorylase) | LGADAVGMSTVPEVIVAR | Oxidation (M) | 2 | 1799.95 | -0.82 | 237080 |
| G3TZN5 | RAB GTPase activating protein 1 like | IQELEHQRGALMNEIQAAK | Deamidation (NQ),di-oxidation (M) | 3 | 2211.10 | 2.22 | 481810 |
| G3SZA5 | RAB guanine nucleotide exchange factor 1 | DLIDWTDGIAKEVQDIVEKYPLDIK | Dioxidation (Y) | 3 | 2947.51 | -0.17 | 812320 |
| G3TQR7 | RAB43, member RAS oncogene family | TITQSYYR | 2 Dopaquinone (Y) | 2 | 1058.47 | -0.27 | 275560 |
| G3U030 | Rab-GAP TBC domain-containing protein | DEDMEKQLLYLR | Acetyl (K),Oxidation (M),Deamidation (NQ) | 2 | 1610.75 | 4.36 | 511840 |
| G3UK01 | RAD54 like | LTPLQMELYKR | Acetyl (K),di-oxidation (M) | 2 | 1464.76 | -1.99 | 196840 |
| G3T7U2 | Raf-1 proto-oncogene, serine/threonine kinase | LTDSSKTSNTIR | Deamidation (NQ) | 2 | 1322.67 | 1.39 | 1791900 |
| G3TBK3 | Ral guanine nucleotide dissociation stimulator like 1 | ISIEDNNGNMYK | 2 Deamidation (NQ),Oxidation (Y) | 2 | 1414.59 | -3.09 | 1338500 |
| G3TXF8 | Ras association domain family member 8 | VLNNCKTTADELK | 2 Deamidation (NQ),Trioxidation (C) | 2 | 1554.71 | -4.25 | 933760 |
| G3U9L7 | RCR-type E3 ubiquitin transferase | WMGKDGQQK | Acetyl (K),2 Deamidation (NQ),di-oxidation (M) | 2 | 1152.48 | -3.60 | 543680 |
| G3SNQ4 | Receptor expression-enhancing protein | VSWIISR | Kynurenine | 2 | 863.49 | -3.41 | 1834300 |
| G3SXZ6 | Receptor protein-tyrosine kinase, EC 2.7.10.1 | KILNSIQVMRAQMNQ | Oxidation (M),Deamidation (NQ) | 2 | 1789.92 | 2.01 | 558680 |
| G3UGN6 | Regulator of chromosome condensation 2 | IKKLPEYNPR | Dopaquinone (Y) | 2 | 1270.70 | -3.50 | 1593500 |
| G3TLF2 | Regulatory factor X7 | SQSVPLTVMMQTAFPNALQK | Oxidation (M),3 Deamidation (NQ) | 2 | 2209.06 | -1.15 | 696570 |
| G3TSA0 | Replication initiator 1 | LPLPCPECGRR | 2 Trioxidation (C) | 2 | 1449.63 | -0.44 | 140230 |
| G3SXH4 | REST corepressor 2 | KEVQVSQYR | Dioxidation (Y) | 2 | 1167.59 | 0.31 | 1523000 |
| G3TRX5 | Retinoic acid induced 14 | MIDELNKQVSELSQLYK | Acetyl (K),3 Deamidation (NQ) | 2 | 2082.01 | -3.37 | 182490 |
| G3TEJ9 | Rho GTPase activating protein 1 | RSANTQIVR | 2 Deamidation (NQ) | 2 | 1045.55 | -0.31 | 232300 |
| G3U810 | Ribonuclease P/MRP subunit p25 like | ATGKAVSCAEIVK | Acetyl (K),Trioxidation (C) | 2 | 1422.70 | -1.79 | 249280 |
| G3UN48 | Ribosomal protein S6 kinase | NLAQKPKTMDVER | Acetyl (K),Oxidation (M),Deamidation (NQ) | 2 | 1587.79 | -3.71 | 317870 |
| G3UGV3 | Ribosome biogenesis protein BOP1 | EVQLTDEQVALVRRLQR | Deamidation (NQ) | 2 | 2053.13 | -4.29 | 124050 |
| G3TKX6 | RING-type domain-containing protein | RAAIMRGAQQQR | 2 Deamidation (NQ) | 2 | 1386.72 | -2.22 | 1709200 |
| G3SUP7 | RING-type E3 ubiquitin transferase RAD18, EC 2.3.2.27 | NNRVLDELVK | Deamidation (NQ) | 3 | 1199.65 | 0.27 | 374550 |
| G3T4F2 | RNA helicase, EC 3.6.4.13 | EVRQLAEDFLR | Glu->pyro-Glu,Deamidation (NQ) | 2 | 1357.70 | -1.96 | 256610 |
| G3TA14 | RNA helicase, EC 3.6.4.13 | ELALQVEAECSKYSYK | Glu->pyro-Glu,Oxidation (Y),Dopaquinone (Y) | 2 | 1928.88 | -2.05 | 193720 |
| G3TF85 | RNA helicase, EC 3.6.4.13 | LIDHLENTKGFNLR | 2 Deamidation (NQ) | 2 | 1670.86 | -2.31 | 2110500 |
| G3T6Q3 | RNA-binding protein 8A | RGGRSHSTSPDWR | Kynurenine | 2 | 1501.71 | 2.15 | 86834 |
| G3TIE4 | Rubicon autophagy regulator | AGATHVNRCMLCQAK | Oxidation (M),Deamidation (NQ) | 2 | 1732.78 | 0.29 | 187530 |
| G3SNC1 | RUN and TBC1 domain-containing protein 3 | DLAESEGWAPRR | Dioxidation (W) | 2 | 1417.66 | 3.44 | 336540 |
| G3U2J9 | RUN domain containing 3B | LRENQLSESVSQNK | 3 Deamidation (NQ) | 2 | 1633.78 | 3.08 | 9748900 |
| G3SMR6 | SCP domain-containing protein | YEIINLYYFQWK | Dioxidation (Y),2 di-iodotyrosine | 3 | 2214.42 | -1.20 | 1960300 |
| G3SNA9 | SDS3 homolog, SIN3A corepressor complex component | PIMTRKLR | Oxidation (M) | 2 | 1029.61 | -3.59 | 368660 |
| G3UK56 | SEC24 homolog D, COPII coat complex component | MIMNIIQQKRPYSMK | 2 Oxidation (M),Deamidation (NQ),di-oxidation (M) | 2 | 1944.95 | 0.02 | 1682200 |
| G3TKN1 | Secretion regulating guanine nucleotide exchange factor | VTAVWSGWTHLVAQT | 2 Oxolactone | 2 | 1682.81 | 3.35 | 147150 |
| G3TC31 | Serine and arginine repetitive matrix 1 | MMQINLTGFLNGKNAR | 2 Oxidation (M) | 2 | 1838.91 | -1.31 | 183870 |
| G3T7P0 | Serine protease 37 | ECQQTEQGK | Glu->pyro-Glu,Deamidation (NQ) | 2 | 1089.44 | -4.02 | 581380 |
| G3T413 | Serine/threonine-protein kinase 40, EC 2.7.11.1 | KLLVLDPQQR | 2 Deamidation (NQ) | 2 | 1210.69 | 0.73 | 135650 |
| G3SLX2 | Serpin family B member 12 | TQTLLFYGR | Unmodified | 2 | 1097.59 | -0.31 | 705630 |
| G3TL68 | Serpin family B member 7 | NYEIKHYLR | Deamidation (NQ),Dopaquinone (Y) | 2 | 1249.61 | -2.05 | 270060 |
| G3TEY6 | Serpin family F member 1 | LDLQEINNWVQAQMK | Oxidation (M),2 Deamidation (NQ) | 2 | 1846.88 | 1.14 | 101300 |
| G3SQ86 | SFI1 centrin binding protein | AEEAAQYK | Deamidation (NQ) | 1 | 909.41 | 0.53 | 1095100 |
| G3TQW4 | Short stature homeobox 2 | MPFQQVTLNVSDGR | 3 Deamidation (NQ) | 2 | 1593.73 | -0.64 | 619510 |
| G3TR45 | Shugoshin 1 | NRNAQRR | 2 Deamidation (NQ) | 2 | 915.46 | 2.81 | 265400 |
| G3TA18 | Sideroflexin 3 | ICMAIPAMAIPPVIMDSLEK | 2 di-oxidation (M) | 2 | 2263.10 | -0.01 | 151650 |
| G3TQB1 | Sin3A associated protein 130 | KAMLQEIANQK | Acetyl (K),di-oxidation (M) | 2 | 1346.69 | -2.09 | 1039800 |
| G3TB94 | Small nuclear RNA activating complex polypeptide 1 | GDMQNGNKEAK | Acetyl (K),2 Deamidation (NQ) | 2 | 1234.51 | -2.44 | 354950 |
| G3TPB8 | Small nuclear RNA activating complex polypeptide 2 | LLLLNTPR | Deamidation (NQ) | 2 | 939.58 | -0.15 | 192660 |
| G3TM92 | SMCR8-C9orf72 complex subunit | TLRCPMYR | di-oxidation (M) | 2 | 1127.52 | -1.30 | 2163100 |
| G3SZS6 | Sodium/potassium-transporting ATPase subunit alpha | LNIAVEQVNEWDAK | Deamidation (NQ),Oxolactone | 2 | 1642.78 | -2.53 | 253650 |
| G3UC99 | Solute carrier family 25 member 39 | ELGTCVQAAVAQGGWR | 2 Deamidation (NQ),Oxolactone | 2 | 1717.77 | 1.98 | 556200 |
| G3UC24 | Solute carrier family 25 member 48 | LQMQTQPFR | Oxidation (M),3 Deamidation (NQ) | 2 | 1166.53 | 3.15 | 603950 |
| G3UL64 | Solute carrier family 9 member B2 | VTVTVSQFSY | Deamidation (NQ),Oxidation (Y) | 2 | 1146.54 | 0.67 | 245500 |
| G3T5X2 | Sorting nexin (SNX5) | EVDDFFEQEK | Glu->pyro-Glu,Deamidation (NQ) | 2 | 1267.52 | 3.22 | 80489 |
| G3T5V9 | Sorting nexin (SNX6) | LSDLLKYYLR | Dioxidation (Y) | 2 | 1314.72 | -1.57 | 376290 |
| G3UJN9 | Spectrin repeat containing nuclear envelope protein 1 | LSLDQALVK | Deamidation (NQ) | 2 | 986.56 | 0.84 | 1259500 |
| G3T3F4 | Sperm associated antigen 7 | DAAHMLQANK | Oxidation (M),2 Deamidation (NQ) | 2 | 1115.49 | -3.68 | 116670 |
| G3SU15 | Spermatogenesis associated serine rich 2 | YRNSSWYSSGSR | Deamidation (NQ) | 2 | 1449.63 | 0.85 | 8764700 |
| G3U2L0 | Spermatogenic leucine zipper 1 | NKKAHNNWFNSR | Deamidation (NQ),Oxidation (W) | 2 | 1531.73 | -4.39 | 873780 |
| G3T130 | Sphingosine-1-phosphate lyase 1 | ESVTQIMKNPK | Acetyl (K),Deamidation (NQ) | 2 | 1316.66 | -2.80 | 46812 |
| G3TPJ6 | ST6 N-acetylgalactosaminide alpha-2,6-sialyltransferase 2 | KFSDHYFDR | Oxidation (Y) | 2 | 1229.55 | 1.96 | 121730 |
| G3TL47 | Striatin | SKLQDMLANLR | 2 Deamidation (NQ) | 2 | 1289.66 | -3.42 | 443310 |
| G3SL67 | Succinyl-CoA:3-ketoacid-coenzyme A transferase, EC 2.8.3.5 | VVVTMEHSAKGNAHK | Oxidation (M),Deamidation (NQ) | 2 | 1623.80 | 2.06 | 211110 |
| G3U794 | SUMO specific peptidase 5 | TWIQDESLFAK | Deamidation (NQ),Oxolactone | 2 | 1351.63 | -2.92 | 143870 |
| G3TD30 | Survival motor neuron domain containing 1 | VKWQQFNNRAYSK | Oxidation (Y) | 2 | 1683.85 | -2.39 | 140660 |
| G3T1N0 | Switching B cell complex subunit SWAP70 | IMKEQALQEAMK | Acetyl (K),Oxidation (M) | 2 | 1476.73 | 2.95 | 416510 |
| G3SQN9 | Syncoilin, intermediate filament protein | EAGAKALQKTQAEIQEMK | 2 Deamidation (NQ) | 2 | 1974.99 | 0.34 | 219780 |
| G3TTS2 | Syntaxin binding protein 5 like | QNGRSKMDMMK | Oxidation (M),Deamidation (NQ) | 2 | 1341.58 | 2.39 | 416970 |
| G3SLV9 | Talin 2 | IASSKTANPVAKR | 2 Acetyl (K),Deamidation (NQ) | 2 | 1426.78 | -0.13 | 9019500 |
| G3TF90 | TAO kinase 3 | LQHQTELENQLEYNKRR | Dopaquinone (Y) | 3 | 2212.10 | -1.63 | 1670000 |
| G3T7E8 | TAR (HIV-1) RNA binding protein 1 | VGWNRGNPIWR | Kynurenine | 2 | 1357.70 | -2.20 | 278410 |
| G3UG62 | TBC1 domain family member 10A | WLDMLNNWDK | Oxolactone,Dioxidation (W) | 2 | 1379.58 | 3.15 | 747420 |
| G3UC74 | T-box transcription factor 2 | AKYILLMDIVAADDCR | Acetyl (K) | 3 | 1907.95 | -3.82 | 319390 |
| G3SS52 | Terpene cyclase/mutase family member, EC 5.4.99.- | QMNKGGFPFSTR | Acetyl (K),Oxidation (M),Deamidation (NQ) | 2 | 1427.65 | -3.86 | 2354700 |
| G3UNE6 | Testis expressed 15, meiosis and synapsis associated | INWDALLGSSNWETETLK | 2 Kynurenine | 2 | 2084.01 | 0.34 | 312220 |
| G3TX85 | Testis specific 10 | YQNTLQLEQEVK | 2 Deamidation (NQ) | 3 | 1493.72 | 3.57 | 100140 |
| G3TL41 | Tetratricopeptide repeat domain 19 | LASIYAAQNR | Deamidation (NQ),Dopaquinone (Y) | 2 | 1120.55 | 0.76 | 207930 |
| G3SRN0 | Tetratricopeptide repeat domain 8 | LNLTKYAQK | Deamidation (NQ),Dopaquinone (Y) | 2 | 1092.58 | 0.35 | 499450 |
| G3U1B2 | Tetratricopeptide repeat, ankyrin repeat and coiled-coil containing 2 | TLPVAQAYQDNLYR | Deamidation (NQ),2 Oxidation (Y) | 2 | 1683.81 | -2.77 | 135580 |
| G3UL46 | Toll-like receptor | SLNSIPSGLTAAVK | Deamidation (NQ) | 2 | 1357.75 | 1.40 | 1004700 |
| G3SXG7 | TOPBP1 interacting checkpoint and replication regulator | AEDAFSWGQFGLGSR | Oxolactone | 2 | 1640.72 | -1.98 | 314510 |
| G3STU1 | Tousled like kinase 1 | QQEWVNQQR | 3 Deamidation (NQ),Oxolactone | 2 | 1231.51 | -4.27 | 1867700 |
| G3UGS9 | Transcription factor 3 | GSAQYYPSYPGNPRR | 3-iodotyrosine | 2 | 1837.70 | -2.05 | 130150 |
| G3TIC7 | Transformer 2 beta homolog | GYDRGYDDRDYYSR | Oxidation (Y),Dopaquinone (Y) | 2 | 1829.72 | 3.33 | 440950 |
| G3TJE7 | Transmembrane and coiled-coil domain family 2 | LANNADKQQVSR | 3 Deamidation (NQ) | 2 | 1345.65 | 3.16 | 742690 |
| G3TKL4 | Transmembrane protein 242 | KKSPEWFSK | Kynurenine | 2 | 1139.60 | -0.15 | 108660 |
| G3SXG5 | Transporter | LEIMLEPK | Unmodified | 2 | 971.54 | 3.21 | 530460 |
| G3UB00 | Trehalase | QQGRSCSPGSLGTGK | Deamidation (NQ),Trioxidation (C) | 2 | 1567.69 | 0.58 | 1942500 |
| G3SU13 | Tripartite motif containing 47 | VLCPINYPESPTR | Deamidation (NQ),Trioxidation (C) | 2 | 1593.73 | -1.82 | 948140 |
| G3T118 | Tryptophan 2,3-dioxygenase, TDO, EC 1.13.11.11 | EIFQNGHVRDERNMLK | Deamidation (NQ) | 3 | 1985.97 | -0.21 | 316980 |
| G3UI44 | TSPY like 4 | GAVGVASQMMAGAK | Deamidation (NQ),2 di-oxidation (M) | 2 | 1341.59 | -2.97 | 403370 |
| G3TGJ7 | Tyrosinase | ACVSSKSMMEK | Acetyl (K),Oxidation (M),di-oxidation (M) | 2 | 1346.55 | -2.11 | 152690 |
| G3T029 | Tyrosine-protein kinase, EC 2.7.10.2 | NKNSINILWKLEVAK | Deamidation (NQ),Kynurenine | 2 | 1774.00 | -0.65 | 1763500 |
| G3T042 | U6 small nuclear RNA (adenine-(43)-N(6))-methyltransferase, EC 2.1.1.- | LLSTTREYSLYLFLPCVVPEETSAK | Oxidation (Y) | 3 | 2931.49 | 4.24 | 566580 |
| G3SMR1 | Ubiquitinyl hydrolase 1, EC 3.4.19.12 | DSVCSNSNSNNGKNAR | Deamidation (NQ),Trioxidation (C) | 2 | 1771.70 | 3.39 | 2846600 |
| G3TI23 | Uncharacterized protein | QSAELDNRLFK | Deamidation (NQ) | 2 | 1320.67 | -0.43 | 6203200 |
| G3SMZ1 | Uncharacterized protein | GAMLTHGNVVADFSGFLKVTEKVIFPR | Oxidation (M),Deamidation (NQ) | 3 | 2949.54 | 4.28 | 4160300 |
| G3SNW1 | Uncharacterized protein | SVNGAEIIM | Deamidation (NQ) | 1 | 933.45 | -3.73 | 699170 |
| G3SP29 | Uncharacterized protein | WEPWQDCNR | Deamidation (NQ),Oxolactone | 2 | 1304.49 | 1.48 | 32527000 |
| G3SQJ9 | Uncharacterized protein | MVELWAWQEGAR | Deamidation (NQ),Oxidation (W) | 2 | 1491.68 | 0.71 | 216640 |
| G3SS98 | Uncharacterized protein | NSILLLIQTMCEKSYIEAR | Oxidation (M),2 Deamidation (NQ) | 3 | 2299.14 | 0.74 | 477110 |
| G3SSW2 | Uncharacterized protein | MRLEQDLKK | Acetyl (K),Deamidation (NQ),di-oxidation (M) | 2 | 1234.62 | -2.59 | 6724200 |
| G3STN8 | Uncharacterized protein | KLEIAQNILADEQER | 3 Deamidation (NQ) | 2 | 1771.88 | -1.57 | 1198600 |
| G3SUM2 | Uncharacterized protein | QAWTTDISRLLWR | Deamidation (NQ),Oxolactone | 2 | 1659.84 | 2.58 | 111920 |
| G3SV32 | Uncharacterized protein | MMNKHPKMVSR | 2 Acetyl (K) | 2 | 1441.70 | 0.00 | 444620 |
| G3SWX5 | Uncharacterized protein | MEANQKAKK | Oxidation (M),2 Deamidation (NQ) | 2 | 1064.52 | -3.12 | 274120000 |
| G3SXI4 | Uncharacterized protein | LAREMLLMR | 2 Oxidation (M) | 2 | 1163.62 | 2.41 | 473690 |
| G3SZ50 | Uncharacterized protein | PYQTMSNPMSK | 2 di-oxidation (M) | 2 | 1346.55 | -0.17 | 96309 |
| G3T2J6 | Uncharacterized protein | GTISILTMYHR | Oxidation (M) | 3 | 1306.67 | -0.22 | 8755300 |
| G3T359 | Uncharacterized protein | ESKFQWR | Kynurenine | 2 | 983.48 | 0.55 | 83967 |
| G3T3E2 | Uncharacterized protein | QLIEWLNK | 2 Deamidation (NQ),Oxidation (W) | 2 | 1060.54 | -3.98 | 267220 |
| G3T4C7 | Uncharacterized protein | LSMENEELLWK | Oxolactone | 2 | 1404.66 | 3.72 | 2162600 |
| G3T6I4 | Uncharacterized protein | EEKVDFQELLVK | Glu->pyro-Glu,Deamidation (NQ) | 2 | 1458.76 | -2.25 | 191160 |
| G3T8C8 | Uncharacterized protein | LEQLQQAVAR | Deamidation (NQ) | 2 | 1155.62 | -0.41 | 276950 |
| G3T963 | Uncharacterized protein | PYQCPECEYCTNRADALR | 3-iodotyrosine,di-iodotyrosine | 4 | 2679.65 | 3.36 | 268060 |
| G3T9T5 | Uncharacterized protein | AELEKHGYKMETS | Acetyl (K),di-oxidation (M) | 2 | 1595.71 | 2.97 | 206290 |
| G3TAQ7 | Uncharacterized protein | EGKESKEVR | 2 Acetyl (K),Glu->pyro-Glu | 2 | 1126.56 | -0.86 | 496050 |
| G3TCG3 | Uncharacterized protein | ALECYCKGNAFMK | Oxidation (M),Dopaquinone (Y) | 2 | 1620.67 | -1.12 | 115630 |
| G3TDG7 | Uncharacterized protein | GEVTWDCVK | Trioxidation (C) | 2 | 1140.48 | -1.97 | 662120 |
| G3TFK2 | Uncharacterized protein | TLNLCKMIDK | Acetyl (K),Deamidation (NQ),di-oxidation (M) | 2 | 1309.63 | 0.05 | 222150 |
| G3TGT5 | Uncharacterized protein | AGIQIMAGKTLR | di-oxidation (M) | 2 | 1289.71 | -3.46 | 399950 |
| G3TIC5 | Uncharacterized protein | NMAEAPKGWAQRR | Deamidation (NQ),Dioxidation (W) | 2 | 1546.73 | -1.92 | 7407300 |
| G3TLL1 | Uncharacterized protein | QNLDYLNSYLEK | Deamidation (NQ),Oxidation (Y),Dioxidation (Y) | 2 | 1547.70 | 3.02 | 187080 |
| G3TLL6 | Uncharacterized protein | EVFAPYYAEILKNVI | Deamidation (NQ),Dioxidation (Y) | 2 | 1800.92 | -0.22 | 252430 |
| G3TMN4 | Uncharacterized protein | DPCTANPYRK | Trioxidation (C) | 2 | 1268.55 | -3.99 | 3710800 |
| G3TPW1 | Uncharacterized protein | KSGYQMEKDELLSYIK | Dopaquinone (Y),3-iodotyrosine | 2 | 2070.85 | -2.11 | 186850 |
| G3TRK1 | Uncharacterized protein | FGCTKLACSPTLIR | Acetyl (K),Trioxidation (C) | 2 | 1712.82 | 0.60 | 942570 |
| G3TRN6 | Uncharacterized protein | ENVNHVNDLAR | 2 Deamidation (NQ) | 2 | 1281.59 | 0.26 | 627170 |
| G3TVI9 | Uncharacterized protein | SNNKIQNAPSR | 3 Deamidation (NQ) | 2 | 1230.58 | -2.22 | 384780 |
| G3U2V9 | Uncharacterized protein | LAYYAYQK | 2 Oxidation (Y) | 2 | 1050.50 | 3.18 | 89463 |
| G3U307 | Uncharacterized protein | KQQLSIGPCK | 2 Deamidation (NQ) | 2 | 1159.59 | -0.89 | 733460 |
| G3U415 | Uncharacterized protein | KWPQQVVQK | 2 Deamidation (NQ) | 2 | 1141.61 | -3.07 | 354180 |
| G3U5E2 | Uncharacterized protein | TFNQSLLEAMFSK | Oxidation (M),2 Deamidation (NQ) | 2 | 1532.71 | 3.34 | 1102100 |
| G3U5I9 | Uncharacterized protein | LNFNEYFEVIYK | 2 Deamidation (NQ),Dopaquinone (Y) | 2 | 1593.72 | 4.36 | 617390 |
| G3U5V7 | Uncharacterized protein | IQEENVNLKNPLEK | 2 Deamidation (NQ) | 3 | 1668.86 | 2.58 | 463910 |
| G3UAD5 | Uncharacterized protein | IIQIDIWIH | Deamidation (NQ),Oxidation (W) | 2 | 1166.63 | 4.42 | 674810 |
| G3UAS4 | Uncharacterized protein | VSGSQLSLEMKEEDVLK | Deamidation (NQ),di-oxidation (M) | 3 | 1923.93 | -0.12 | 577580 |
| G3UAW9 | Uncharacterized protein | EARGGRPVASGYGR | Dopaquinone (Y) | 2 | 1445.71 | -1.05 | 335400 |
| G3UEL9 | Uncharacterized protein | VGMLALEMLGR | 2 Oxidation (M) | 2 | 1220.63 | 3.77 | 690070 |
| G3UFX7 | Uncharacterized protein | YMVIQAASLEWKNK | Acetyl (K),Deamidation (NQ),di-oxidation (M) | 2 | 1754.85 | -0.17 | 1882300 |
| G3UGR6 | Uncharacterized protein | SDYQLWVSSGKK | Deamidation (NQ),Oxolactone | 2 | 1411.66 | 2.36 | 315760 |
| G3UIR6 | Uncharacterized protein | VNKDDIQKMNPPK | 2 Acetyl (K),Deamidation (NQ) | 2 | 1610.80 | -2.36 | 84194 |
| G3UL45 | Uncharacterized protein | KIINWNNVK | 2 Deamidation (NQ) | 2 | 1129.61 | -3.09 | 306210 |
| G3X8I5 | Uncharacterized protein | QLQQEDLARR | Deamidation (NQ) | 2 | 1256.65 | 2.04 | 241170 |
| G3TCJ6 | USH1 protein network component harmonin binding protein 1 | EDELARTQASLQAMR | Glu->pyro-Glu,Deamidation (NQ) | 2 | 1700.82 | -1.78 | 223750 |
| G3TRU9 | Utrophin | EVSTKFQLFQKPANFEQR | Acetyl (K),Deamidation (NQ) | 3 | 2239.13 | -1.11 | 330350 |
| G3T3S2 | Vascular endothelial growth factor C | FHHQTCSCYR | 2 Trioxidation (C) | 2 | 1490.53 | -1.70 | 4939700 |
| G3SUS0 | Vascular endothelial growth factor D | VIDEEWQR | Deamidation (NQ),Oxolactone | 2 | 1088.48 | -3.65 | 689280 |
| G3UDW0 | VPS39 subunit of HOPS complex | IQQIHVVSQFK | 2 Deamidation (NQ) | 2 | 1327.71 | -1.66 | 2057600 |
| G3SZS3 | V-type proton ATPase subunit a | NLLELIEYTHMLR | 3-iodotyrosine | 2 | 1769.77 | 3.74 | 479540 |
| G3SX59 | V-type proton ATPase subunit H | QLQSEQPQTAAAR | 3 Deamidation (NQ) | 2 | 1429.67 | 0.57 | 520710 |
| G3U7H5 | WAP, follistatin/kazal, immunoglobulin, kunitz and netrin domain containing 1 | LEDAGLYTCTAR | Trioxidation (C) | 2 | 1416.62 | -4.19 | 334420 |
| G3TCR2 | WASH complex subunit 4 | MESILSKEQRMDK | Oxidation (M),Deamidation (NQ) | 2 | 1610.76 | -3.49 | 555860 |
| G3TAU4 | WD repeat and FYVE domain containing 3 | RWSDQLSLDEK | Dioxidation (W) | 2 | 1407.66 | 1.26 | 282540 |
| G3SN23 | WD repeat domain 20 | QGESFAVHTCKSKSTR | Acetyl (K),Trioxidation (C) | 2 | 1911.87 | 3.72 | 3205600 |
| G3UAJ6 | WD_REPEATS_REGION domain-containing protein | DLERTSYNAFLNMKNR | Oxidation (M),Deamidation (NQ) | 2 | 1987.94 | -0.66 | 425210 |
| G3SMX9 | WSC domain containing 2 | TISAYIKMVDAALKGR | Oxidation (M),Dioxidation (Y) | 2 | 1783.95 | -3.62 | 1567500 |
| G3TE29 | Zinc finger CCCH-type containing 12B | MTATAVVGTPKMK | 2 di-oxidation (M) | 2 | 1397.69 | -0.56 | 1295300 |
| G3UDK7 | Zinc finger CCCH-type containing 4 | TVLWNPEDLIPLPVPK | Deamidation (NQ),Kynurenine | 2 | 1835.01 | -2.26 | 1032900 |
| G3SQR5 | Zinc finger MYND-type containing 8 | FQTSSQKWHMQKMQR | Oxidation (M),Deamidation (NQ) | 2 | 1966.91 | -1.86 | 102890 |
| G3TG66 | Zinc finger protein 148 | NDEEQMETHER | Oxidation (M),2 Deamidation (NQ) | 2 | 1434.52 | 4.04 | 215440 |
| G3TH53 | Zinc finger protein 287 | LEESYGCDDR | Trioxidation (C) | 2 | 1290.47 | 4.17 | 5082600 |
| G3T0I2 | Zinc finger protein 292 | NWQAYMQYCVLCDKEFLGHR | 3 Deamidation (NQ) | 3 | 2620.12 | -1.59 | 3425300 |
| G3SN36 | Zinc finger protein 532 | EDTKAPSPK | Acetyl (K),Glu->pyro-Glu | 2 | 995.49 | -0.28 | 394320 |
| G3SML9 | Zinc finger SWIM-type containing 5 | LTLWRQQGTSMTDKCR | Oxidation (W) | 2 | 1995.96 | 0.45 | 200790 |

***Table S2.*** *List of peptides and proteins identified from the c-RAP database. For each peptide all the features (same sequence and different modifications) with the highest intensity are reported. C-RAP proteins identified with peptides in common with Proboscidea sequences are indicated with the letters a-h.*

| **Proteins** | **Peptide Sequence** | **Modifications** | **Theoretical Mass** | **Mass error**  **[ppm]** | **Charge** | **MaxQuant Intensity** |
| --- | --- | --- | --- | --- | --- | --- |
| CON__A2A4G1 | VLDELTLAR | Unmodified | 1028.59 | -0.35 | 2 | 38812000 |
|  | LASYLDKVR | Unmodified | 1063.60 | 0.57 | 2 | 11203000 |
|  | LAADDFR | Unmodified | 806.39 | 0.02 | 2 | 1.99E+08 |
|  | TRLEQEIATYR | Unmodified | 1378.72 | -0.36 | 2 | 3810900 |
|  | VTMQNLNDR | Unmodified | 1089.52 | -0.27 | 2 | 77324000 |
| CON__O43790 | LEAAVAQSEQQGEAALSDAR | Unmodified | 2042.99 | 0.27 | 2 | 283450 |
| CON__P00761 | IITHPNFNGNTLDNDIMLIK | Oxidation (M).Deamidation (NQ) | 2299.15 | -0.43 | 3 | 18272000 |
|  | LGEHNIDVLEGNEQFINAAK | Unmodified | 2210.10 | -0.31 | 3 | 52298000 |
|  | LSSPATLNSR | Unmodified | 1044.56 | -0.36 | 2 | 3.51E+08 |
| CON__P02533 a) | APSTYGGGLSVSSSR | Unmodified | 1424.69 | -0.21 | 2 | 39595000 |
|  | DAEEWFFTK | Unmodified | 1171.52 | 0.62 | 2 | 848200 |
|  | ILTATVDNANVLLQIDNAR | Unmodified | 2053.12 | 0.30 | 2 | 14676000 |
|  | GSCGIGGGIGGGSSR | Unmodified | 1277.58 | 0.81 | 2 | 762690 |
|  | ISSVLAGGSCR | Unmodified | 1105.55 | 0.07 | 2 | 1240600 |
|  | QFTSSSSMK | Oxidation (M).Gln->pyro-Glu | 1000.42 | 0.55 | 2 | 554860 |
|  | LLEGEDAHLSSSQFSSGSQSSR | Unmodified | 2308.06 | 1.16 | 3 | 10203000 |
|  | QRPAEIKDYSPYFK | Gln->pyro-Glu | 1723.86 | -0.49 | 3 | 894310 |
|  | MSVEADINGLR | Oxidation (M) | 1219.59 | -0.08 | 2 | 879610 |
|  | MSVEADINGLRR | Oxidation (M) | 1375.69 | 1.48 | 2 | 1452100 |
|  | TKYETELNLR | Unmodified | 1265.66 | 1.49 | 2 | 1968700 |
|  | ASLENSLEETK | Unmodified | 1219.59 | -0.23 | 2 | 8818800 |
|  | ASLENSLEETKGR | Unmodified | 1432.72 | -0.40 | 3 | 2585200 |
|  | ALEEANADLEVK | Unmodified | 1300.65 | 0.04 | 2 | 45156000 |
|  | ILNEMRDQYEK | Unmodified | 1437.69 | -0.14 | 3 | 596010 |
|  | TEELNREVATNSELVQSGK | Unmodified | 2103.04 | 0.87 | 3 | 1156800 |
| CON__P02535-1 | LENEIQTYR | Unmodified | 1164.58 | -0.16 | 2 | 56659000 |
|  | QSLEASLAETEGR | Gln->pyro-Glu | 1372.65 | 0.21 | 2 | 24545000 |
|  | QSVEADINGLRR | Gln->pyro-Glu | 1339.68 | 0.10 | 2 | 71641000 |
|  | QSVEADINGLR | Gln->pyro-Glu | 1183.58 | 0.99 | 2 | 15232000 |
| CON__P02538 | AIGGGLSSVGGGSSTIK | Unmodified | 1446.77 | -0.41 | 2 | 20727000 |
|  | AIGGGLSSVGGGSSTIKYTTTSSSSR | Unmodified | 2417.20 | -1.55 | 3 | 2611700 |
|  | SGFSSVSVSR | Unmodified | 1011.50 | -0.14 | 2 | 14388000 |
| CON__P02538 | DVDAAYMNK | Unmodified | 1025.45 | 0.34 | 2 | 307310 |
|  | FLEQQNKVLETK | Unmodified | 1475.80 | -0.35 | 3 | 373260 |
|  | AEAESWYQTK | Unmodified | 1211.55 | 0.53 | 2 | 702560 |
|  | ADTLTDEINFLR | Unmodified | 1406.70 | 0.64 | 2 | 7963400 |
|  | ISIGGGSCAISGGYGSR | Unmodified | 1597.75 | 0.55 | 2 | 936480 |
|  | NTKQEIAEINR | Unmodified | 1314.69 | 0.18 | 3 | 1160700 |
|  | QEIAEINR | Gln->pyro-Glu | 954.48 | 1.10 | 2 | 621180 |
|  | SLYGLGGSK | Unmodified | 880.47 | -1.42 | 2 | 5154600 |
|  | TAAENEFVTLK | Unmodified | 1221.62 | 1.03 | 2 | 3466800 |
|  | TAAENEFVTLKK | Unmodified | 1349.72 | -0.26 | 3 | 5001200 |
|  | QLDSIVGER | Unmodified | 1015.53 | 0.23 | 2 | 9725600 |
|  | QNLEPLFEQYINNLR | Gln->pyro-Glu | 1872.94 | -1.71 | 2 | 715920 |
|  | WTLLQEQGTK | Unmodified | 1202.63 | -0.24 | 2 | 6313900 |
| CON__P02666 | AVPYPQR | Unmodified | 829.44 | 0.24 | 2 | 1044300 |
|  | DMPIQAFLLYQEPVLGPVR | Oxidation (M) | 2201.16 | 0.14 | 2 | 542850 |
| CON__P02668 | SCQAQPTTMAR | Oxidation (M).Deamidation (NQ) | 1266.53 | -1.97 | 2 | 337060 |
| CON__P04259 | ATGGGLSSVGGGSSTIK | Unmodified | 1434.73 | -1.09 | 2 | 12459000 |
|  | ATGGGLSSVGGGSSTIKYTTTSSSSR | Unmodified | 2405.17 | 1.24 | 3 | 1937500 |
|  | SGFSSISVSR | Unmodified | 1025.51 | 0.53 | 2 | 23124000 |
|  | VLDTKWTLLQEQGTK | Unmodified | 1758.95 | -0.25 | 3 | 459180 |
| CON__P04264 | AEAESLYQSKYEELQITAGR | Unmodified | 2285.12 | 1.01 | 3 | 2395300 |
|  | FSSCGGGGGSFGAGGGFGSR | Unmodified | 1764.73 | -0.64 | 2 | 7216800 |
|  | GENALKDAK | Unmodified | 944.49 | 0.60 | 2 | 621330 |
|  | GGGGGGYGSGGSSYGSGGGSYGSGGGGGGGR | Unmodified | 2382.94 | 0.11 | 2 | 52279000 |
|  | GSGGGSSGGSIGGR | Unmodified | 1091.50 | -0.06 | 2 | 4877300 |
|  | GSYGSGGSSYGSGGGSYGSGGGGGGHGSYGSGSSSGGYR | Unmodified | 3311.30 | 0.07 | 3 | 546140 |
|  | KQISNLQQSISDAEQR | Unmodified | 1843.94 | -0.13 | 3 | 1123800 |
|  | LNDLEDALQQAKEDLAR | Unmodified | 1940.98 | -0.23 | 3 | 257240 |
|  | MSGECAPNVSVSVSTSHTTISGGGSR | Oxidation (M) | 2580.15 | -0.16 | 3 | 7286100 |
|  | NKLNDLEDALQQAK | Unmodified | 1598.83 | 0.73 | 2 | 3340200 |
|  | NMQDMVEDYR | 2 Oxidation (M) | 1331.51 | -0.01 | 2 | 4447400 |
|  | NSKIEISELNR | Unmodified | 1301.69 | -0.05 | 3 | 2594800 |
|  | QISNLQQSISDAEQR | Gln->pyro-Glu | 1698.82 | 0.31 | 2 | 1.27E+08 |
|  | QISNLQQSISDAEQRGENALK | Gln->pyro-Glu | 2311.14 | -0.51 | 2 | 9391100 |
|  | QISNLQQSISDAEQRGENALKDAK | Gln->pyro-Glu | 2625.30 | 0.46 | 3 | 753160 |
|  | SGGGFSSGSAGIINYQR | Unmodified | 1656.79 | 0.32 | 2 | 45522000 |
|  | SGYRSGGGFSSGSAGIINYQR | Deamidation (NQ) | 2120.99 | 0.00 | 2 | 389100 |
|  | SKAEAESLYQSK | Unmodified | 1339.66 | 0.63 | 2 | 30198000 |
|  | SLVNLGGSK | Unmodified | 873.49 | 0.02 | 2 | 514080 |
|  | SLVNLGGSKSISISVAR | Unmodified | 1686.96 | 0.85 | 3 | 1029500 |
|  | LLRDYQELMNTK | Oxidation (M) | 1538.78 | 0.09 | 3 | 7369900 |
|  | NKYEDEINKR | Unmodified | 1307.65 | 0.20 | 3 | 607920 |
|  | FLEQQNQVLQTK | Deamidation (NQ) | 1475.76 | 1.03 | 2 | 32333000 |
|  | LALDLEIATYR | Unmodified | 1276.70 | 0.58 | 2 | 8816900 |
|  | LRSEIDNVK | Unmodified | 1072.59 | -0.08 | 2 | 9608200 |
|  | LRSEIDNVKK | Unmodified | 1200.68 | 0.10 | 3 | 6876900 |
|  | SISISVAR | Unmodified | 831.48 | 0.02 | 2 | 309110 |
|  | SLDLDSIIAEVK | Unmodified | 1301.71 | -0.26 | 2 | 2.2E+08 |
|  | TNAENEFVTIK | Unmodified | 1264.63 | -0.52 | 2 | 1.61E+08 |
|  | TNAENEFVTIKK | Unmodified | 1392.72 | -0.68 | 3 | 1.73E+08 |
|  | YEELQITAGR | Unmodified | 1178.59 | -0.17 | 2 | 3.48E+08 |
|  | SLNNQFASFIDK | Unmodified | 1382.68 | 0.78 | 2 | 4.22E+08 |
|  | SLNNQFASFIDKVR | Unmodified | 1637.85 | 0.47 | 3 | 6536600 |
|  | TLLEGEESR | Unmodified | 1032.51 | 0.04 | 2 | 3.49E+08 |
| CON__P07477 | TLNNDIMLIK | Deamidation (NQ) | 1174.63 | 0.22 | 2 | 940630 |
| CON__P08779 | APSTYGGGLSVSSR | Unmodified | 1337.66 | 0.50 | 2 | 28728000 |
|  | EVASNSELVQSSR | Unmodified | 1404.68 | 0.71 | 2 | 2047800 |
|  | IIAATIENAQPILQIDNAR | Unmodified | 2063.14 | 0.47 | 2 | 15862000 |
|  | LLEGEDAHLSSQQASGQSYSSR | Unmodified | 2349.08 | 0.72 | 3 | 2277300 |
|  | NKIIAATIENAQPILQIDNAR | Unmodified | 2305.28 | 0.71 | 3 | 154480 |
|  | QTRPILKEQSSSSFSQGQSS | Gln->pyro-Glu | 2164.04 | -0.68 | 2 | 824830 |
|  | QTVEADVNGLR | Unmodified | 1200.61 | -0.42 | 2 | 308470 |
|  | QTVEADVNGLRR | Gln->pyro-Glu | 1339.68 | 2.28 | 2 | 4358600 |
|  | TEELNKEVASNSELVQSSR | Unmodified | 2119.04 | 0.30 | 3 | 4030300 |
|  | VLQGLEIELQSQLSMK | Oxidation (M) | 1830.98 | -0.10 | 3 | 1883100 |
|  | GQTGGDVNVEMDAAPGVDLSR | Oxidation (M) | 2102.95 | -0.42 | 2 | 542320 |
| CON__P13645 | ELTTEIDNNIEQISSYK | Unmodified | 1995.96 | -0.44 | 2 | 6972600 |
|  | GSLGGGFSSGGFSGGSFSR | Unmodified | 1706.76 | -0.24 | 2 | 2.51E+08 |
|  | GSSGGGCFGGSSGGYGGLGGFGGGSFR | Unmodified | 2341.98 | 0.02 | 2 | 1761400 |
|  | IRLENEIQTYR | Unmodified | 1433.76 | -2.19 | 3 | 16366000 |
|  | LKYENEVALR | Unmodified | 1233.67 | 0.23 | 3 | 42353000 |
|  | NQILNLTTDNANILLQIDNAR | Unmodified | 2366.26 | 1.14 | 3 | 800720 |
|  | NVQALEIELQSQLALK | Unmodified | 1796.00 | -0.15 | 2 | 22585000 |
|  | NVSTGDVNVEMNAAPGVDLTQLLNNMR | 2 Oxidation (M) | 2903.38 | 0.31 | 3 | 19185000 |
|  | RVLDELTLTK | Unmodified | 1186.69 | -0.25 | 2 | 5350400 |
|  | SGGGGGGGGCGGGGGVSSLR | Unmodified | 1548.67 | 1.53 | 2 | 534650 |
|  | SLLEGEGSSGGGGR | Unmodified | 1261.59 | 0.23 | 2 | 64362000 |
|  | SQYEQLAEQNR | Unmodified | 1364.63 | 0.18 | 2 | 43760000 |
|  | SQYEQLAEQNRK | Unmodified | 1492.73 | 0.85 | 3 | 61974000 |
|  | YENEVALR | Unmodified | 992.49 | 0.47 | 2 | 35799000 |
|  | DAEAWFNEK | Unmodified | 1108.48 | 0.41 | 2 | 7992800 |
| CON__P13646-1 | STSVTTTSSASVTTTSNASGR | Unmodified | 2001.95 | 0.36 | 2 | 142530 |
| CON__P13647 | AQYEEIANR | Unmodified | 1092.52 | 0.42 | 2 | 20700000 |
|  | EYQELMNTK | Oxidation (M) | 1170.52 | 0.63 | 2 | 1907700 |
|  | GLGVGFGSGGGSSSSVK | Unmodified | 1438.71 | 1.39 | 2 | 4500500 |
|  | ISISTSGGSFR | Unmodified | 1110.57 | -0.18 | 2 | 52754000 |
|  | LAELEEALQK | Unmodified | 1142.62 | 0.34 | 2 | 9196800 |
|  | NKLAELEEALQK | Unmodified | 1384.76 | 0.06 | 2 | 603480 |
|  | NMQDLVEDFK | Unmodified | 1237.56 | 0.10 | 2 | 295450 |
|  | QCANLQNAIADAEQR | Gln->pyro-Glu | 1683.76 | 0.33 | 2 | 332160 |
|  | SFSTASAITPSVSR | Unmodified | 1409.72 | 0.61 | 2 | 16231000 |
|  | SLYNLGGSKR | Unmodified | 1093.59 | 0.97 | 2 | 1241200 |
|  | TEAESWYQTK | Unmodified | 1241.56 | -0.17 | 2 | 1456400 |
|  | TEAESWYQTKYEELQQTAGR | Unmodified | 2417.11 | 1.81 | 3 | 237330 |
|  | TTAENEFVMLK | Oxidation (M) | 1297.62 | -0.24 | 2 | 5914500 |
|  | TTAENEFVMLKK | Oxidation (M) | 1425.72 | -0.29 | 3 | 3984300 |
|  | VDALMDEINFMK | 2 Oxidation (M) | 1456.66 | -0.63 | 2 | 2136600 |
|  | VSLAGACGVGGYGSR | Unmodified | 1409.67 | -0.50 | 2 | 796130 |
|  | YEELQQTAGR | Unmodified | 1193.57 | 0.33 | 2 | 15512000 |
| CON__P20930 | LAQAYYESTR | Unmodified | 1200.58 | -0.74 | 2 | 328150 |
|  | QSESSHGWTGPSTGVR | Gln->pyro-Glu | 1654.73 | 0.71 | 2 | 1073200 |
| CON__P35527 | DQIVDLTVGNNK | Unmodified | 1314.68 | 0.35 | 2 | 992050 |
|  | FSSSSGYGGGSSR | Unmodified | 1234.52 | 0.87 | 2 | 6065000 |
|  | HGVQELEIELQSQLSK | Unmodified | 1836.96 | -0.47 | 3 | 2681100 |
|  | HGVQELEIELQSQLSKK | Unmodified | 1965.05 | -0.29 | 3 | 252640 |
|  | IKFEMEQNLR | Oxidation (M) | 1322.67 | 0.18 | 3 | 22391000 |
|  | LASYLDKVQALEEANNDLENK | Unmodified | 2376.18 | 1.19 | 3 | 1543200 |
|  | MTLDDFR | Oxidation (M) | 912.40 | 0.33 | 2 | 2036800 |
|  | NYSPYYNTIDDLK | Unmodified | 1604.74 | -0.10 | 2 | 883790 |
|  | QEYEQLIAK | Gln->pyro-Glu | 1103.55 | 0.66 | 2 | 44402000 |
|  | QFSSSYLSR | Gln->pyro-Glu | 1056.49 | 0.16 | 2 | 14900000 |
|  | QGVDADINGLR | Unmodified | 1156.58 | 0.79 | 2 | 20588000 |
|  | QVLDNLTMEK | Oxidation (M).Gln->pyro-Glu | 1188.57 | -0.40 | 2 | 44564000 |
|  | SGGGGGGGLGSGGSIR | Unmodified | 1231.59 | 0.17 | 2 | 2.06E+08 |
|  | STMQELNSR | Oxidation (M) | 1080.49 | 0.16 | 2 | 53413000 |
|  | TLLDIDNTR | Unmodified | 1059.56 | -0.63 | 2 | 2.3E+08 |
|  | TLNDMRQEYEQLIAK | Oxidation (M) | 1866.91 | -0.17 | 3 | 17323000 |
|  | VQALEEANNDLENK | Unmodified | 1585.76 | 0.48 | 2 | 30429000 |
| CON__P35908 | GGGFGGGSGFGGGSGFGGGSGFSGGGFGGGGFGGGR | Unmodified | 2830.19 | -0.07 | 3 | 1212600 |
|  | GGGGGGFRGFSSGSAVVSGGSR | Unmodified | 1898.90 | 0.23 | 3 | 547230 |
|  | GGSGGGGSISGGGYGSGGGSGGR | Unmodified | 1740.74 | 0.11 | 2 | 23443000 |
|  | GGSSSGGGYGSGGGGSSSVK | Unmodified | 1587.68 | 1.33 | 2 | 553100 |
|  | GSSSGGGYSSGSSSYGSGGR | Unmodified | 1739.70 | 0.43 | 2 | 13246000 |
|  | LNDLEEALQQAK | Unmodified | 1370.70 | -0.13 | 2 | 8956300 |
|  | LQGEIAHVK | Unmodified | 993.56 | 0.43 | 2 | 5762700 |
|  | MSGDLSSNVTVSVTSSTISSNVASK | Oxidation (M) | 2473.19 | 1.04 | 3 | 13105000 |
|  | NVQDAIADAEQR | Unmodified | 1328.63 | 0.26 | 2 | 20905000 |
|  | NVQDAIADAEQRGEHALK | Unmodified | 1963.97 | -0.07 | 3 | 320390 |
|  | QSGSRGGSGGGGSISGGGYGSGGGSGGR | Deamidation (NQ) | 2256.97 | 0.56 | 2 | 1087800 |
|  | SISISVAGGGGGFGAAGGFGGR | Unmodified | 1837.91 | 0.71 | 2 | 36744000 |
|  | STSSFSCLSR | Unmodified | 1130.50 | 0.11 | 2 | 1357200 |
|  | TAAENDFVTLK | Unmodified | 1207.61 | -0.54 | 2 | 28442000 |
|  | TAAENDFVTLKK | Unmodified | 1335.70 | 0.15 | 3 | 28473000 |
|  | TSQNSELNNMQDLVEDYKK | Oxidation (M) | 2271.03 | 0.30 | 3 | 749460 |
|  | VDLLNQEIEFLK | Unmodified | 1459.79 | -3.36 | 2 | 30988000 |
|  | VLYDAEISQIHQSVTDTNVILSMDNSR | Oxidation (M) | 3063.48 | -0.23 | 3 | 3412400 |
|  | YEELQVTVGR | Unmodified | 1192.61 | -0.78 | 2 | 49464000 |
|  | YGSGGGSKGGSISGGGYGSGGGK | Unmodified | 1889.85 | 0.92 | 3 | 538770 |
|  | YLDGLTAER | Unmodified | 1036.52 | -0.15 | 2 | 48288000 |
|  | LALDVEIATYRK | Unmodified | 1390.78 | 0.43 | 3 | 218210 |
|  | NLDLDSIIAEVK | Unmodified | 1328.72 | 0.27 | 2 | 68922000 |
|  | GFSSGSAVVSGGSR | Unmodified | 1253.60 | 0.14 | 2 | 1.31E+08 |
|  | GFSSGSAVVSGGSRR | Unmodified | 1409.70 | 0.38 | 3 | 2728100 |
|  | VDPEIQNVK | Unmodified | 1040.55 | 0.18 | 2 | 1.09E+08 |
|  | LALDVEIATYR | Unmodified | 1262.69 | 0.33 | 2 | 2542100 |
|  | GGGFGGGSSFGGGSGFSGGGFGGGGFGGGR | Unmodified | 2398.01 | 0.08 | 2 | 6219000 |
| CON__Q04695 b) | ASLEGNLAETENR | Unmodified | 1402.67 | 0.27 | 2 | 600060 |
|  | GQVGGEINVEMDAAPGVDLSR | Oxidation (M) | 2129.01 | -0.24 | 2 | 354530 |
|  | QFTSSSSIKGSSGLGGGSSR | Gln->pyro-Glu | 1868.89 | -0.21 | 2 | 3449100 |
|  | TIVEEVQDGK | Unmodified | 1116.57 | 0.64 | 2 | 433410 |
|  | TMQALEIELQSQLSMK | 2 Oxidation (M) | 1880.92 | 1.13 | 3 | 559020 |
|  | ILTATVDNANILLQIDNAR | Unmodified | 2067.13 | 0.05 | 2 | 2188900 |
|  | TKFETEQALR | Unmodified | 1221.64 | 0.05 | 2 | 879660 |
| CON__Q2M2I5 c) | LANYLDK | Deamidation (NQ) | 836.43 | 1.11 | 1 | 445810 |
| CON__Q5D862 | FSNSSSSNEFSK | Unmodified | 1319.56 | -0.21 | 2 | 2495600 |
|  | QETTHGQTINTTR | Gln->pyro-Glu | 1468.69 | -0.30 | 2 | 666720 |
|  | QSSYGQHGSGSSQSSGYGQYGSR | Gln->pyro-Glu | 2333.95 | 0.22 | 2 | 386520 |
|  | SVVTVIDVFYK | Unmodified | 1268.70 | 0.94 | 2 | 2761000 |
| CON__Q6KB66-1 | AQYDAVAAR | Unmodified | 963.48 | -0.07 | 2 | 953720 |
|  | LAQLEAALQQAK | Unmodified | 1282.72 | 0.02 | 2 | 956490 |
|  | SAEYGSSLQSSR | Unmodified | 1270.58 | 0.57 | 2 | 794090 |
|  | SEIADLNVR | Unmodified | 1015.53 | 0.43 | 2 | 968160 |
| CON__Q7Z3Y8 d) | SASLQQQISDDAGATTSAR | Unmodified | 1905.90 | -1.73 | 2 | 253480 |
| CON__Q7Z794 | DVDAAYVSK | Unmodified | 966.47 | 0.05 | 2 | 453750 |
|  | QLLEGEESR | Deamidation (NQ) | 1060.50 | 1.14 | 2 | 1138000 |
|  | SLYNLGGSR | Unmodified | 965.49 | -0.53 | 2 | 612410 |
|  | TQYELIAQR | Unmodified | 1120.59 | -2.10 | 2 | 1879200 |
|  | VDTLTGEVNFLK | Unmodified | 1334.71 | 0.36 | 2 | 1885700 |
|  | YQELQITAGR | Deamidation (NQ) | 1178.59 | -0.40 | 2 | 4.23E+08 |
| CON__Q86YZ3 | GSGSGQSPSSGQHGTGFGR | Unmodified | 1746.77 | -0.16 | 3 | 440250 |
|  | QGSGSGQSPGHGQR | Gln->pyro-Glu | 1321.58 | 0.86 | 2 | 553830 |
|  | QSLGHGQHGSGSGQSPSPSR | Gln->pyro-Glu | 1929.87 | -2.13 | 3 | 5620300 |
| CON__Q8N1N4-2 | ATLENDFVVLK | Unmodified | 1247.68 | 0.83 | 2 | 566550 |
|  | IEIDPQFQVVR | Unmodified | 1342.72 | 2.19 | 2 | 1351800 |
|  | QLEQLQGER | Gln->pyro-Glu | 1082.54 | 0.18 | 2 | 507530 |
|  | QNASLQAAITDAEQR | Gln->pyro-Glu | 1597.77 | 0.56 | 2 | 757640 |
|  | TLNNQFASFIDK | Unmodified | 1396.70 | 1.12 | 2 | 1641500 |
|  | VDELEAALR | Unmodified | 1014.53 | 0.82 | 2 | 1391300 |
|  | VQISQLHQEIQR | Unmodified | 1477.80 | -0.19 | 3 | 1248900 |
|  | YLDFSSIITEVR | Unmodified | 1441.75 | -0.76 | 2 | 1035100 |
| CON__Q9C075 | MTNAQIILLIDNAR | Oxidation (M) | 1600.86 | 0.86 | 2 | 262050 |
|  | QNNEYQVLLGIK | Gln->pyro-Glu | 1400.73 | 0.36 | 2 | 226920 |
| CON__Q9NSB2 | QLEVLVSDQAR | Gln->pyro-Glu | 1239.65 | -3.68 | 2 | 191100 |
|  | SLLTPLNLEIDPNAQR | Unmodified | 1792.97 | 0.28 | 2 | 249440 |
|  | VAPATGDLLSTGTR | Unmodified | 1357.72 | 0.47 | 2 | 577680 |
|  | VGGVGVPAAPSITAVTVNK | Unmodified | 1735.98 | -0.18 | 2 | 693780 |
| CON__Q9R0H5 | AQYEEIALK | Unmodified | 1063.55 | 0.97 | 2 | 539240 |
|  | QASNLETAIADAEQR | Gln->pyro-Glu | 1598.75 | 0.22 | 2 | 326740 |
|  | FQELQLAAGR | Unmodified | 1131.60 | 1.23 | 2 | 1003800 |
| CON__Q7RTS7 e) | AEAEALYQTK | Unmodified | 1122.56 | -0.04 | 2 | 1031200 |
| CON__Q9NSB4 f) | FASFINKVR | Deamidation (NQ) | 1081.59 | -0.39 | 2 | 1538400 |
| CON__Q9UE12 g) | QNQEYQVLLDVR | Gln->pyro-Glu | 1486.74 | -2.75 | 2 | 680610 |
|  | LNVEVDAAPTVDLNR | Unmodified | 1624.84 | -0.06 | 2 | 271370 |
|  | LVVQIDNAK | Unmodified | 998.58 | -1.14 | 2 | 604230 |
| Q6B3Z0 h) | KVPQVSTPTLVEVSR | Unmodified | 1638.93 | -0.11 | 3 | 532190 |

1. Keratin 14 OS=Loxodonta Africana
2. Keratin 17/ Keratin 19 OS=Loxodonta Africana
3. Keratin 24 OS=Loxodonta Africana
4. Keratin 27 OS=Loxodonta Africana
5. Keratin 5 OS=Loxodonta Africana
6. Keratin 82 OS=Loxodonta Africana
7. Keratin 32 OS=Loxodonta Africana
8. Albumin OS=Loxodonta Africana

***Table S3****. List of peptides and proteins identified from Viridiplantae database investigation. For each peptide all the features (same sequence and different modifications) with the highest intensity are reported. Proteins identified with at least two peptides are reported in bold. The column Taxa-specie refers to the MaxQuant software assignment before the BLAST search.*

| **Accession Number** | **Description** | ***Specie*** | **Peptide Sequence** | **Modifications** | **Charge** | **Theoretical Mass** | **Mass error [ppm]** | **MaxQuant Intensity** |
| --- | --- | --- | --- | --- | --- | --- | --- | --- |
| **Q43607** | **Prunin 1 Pru du 6** | *Prunus dulcis* | ALPDEVLANAYQISR | Unmodified | 2 | 1658.86 | 1.12 | 1069500 |
|  |  |  | ISTLNSHNLPILR | Unmodified | 3 | 1476.84 | 0.30 | 149940 |
|  |  |  | TEENAFINTLAGR | Unmodified | 2 | 1434.71 | 0.19 | 398760 |
| **P08477** | **Glyceraldehyde-3-phosphate dehydrogenase 2** | *Hordeum vulgare* | AASFNIIPSSTGAAK | Unmodified | 2 | 1433.75 | 0.19 | 651860 |
|  |  |  | VLPELNGK | Unmodified | 2 | 868.50 | -0.11 | 592620 |
|  |  |  | VPTVDVSVVDLTVR | Unmodified | 2 | 1495.82 | -0.91 | 672250 |
| **P59259** | **Histone H4** | *Arabidopsis thaliana* | IFLENVIR | Unmodified | 2 | 1002.59 | -0.10 | 1627600 |
|  |  |  | TLYGFGG | Unmodified | 1 | 713.34 | 0.62 | 1626800 |
|  |  |  | TVTAMDVVYALK | Oxidation (M) | 2 | 1325.69 | -0.61 | 420880 |
|  |  |  | DNIQGITKPAIR | Unmodified | 3 | 1324,74 | 1,23 | 766490 |
|  |  |  | ISGLIYEETR | 3-iodotyrosine | 2 | 1305,51 | 0,95 | 966030 |
|  |  |  | ISGLIYEETR | Unmodified | 2 | 1179,61 | 1,15 | 3903700 |
| **P24459** | **ATP synthase subunit alpha, mitochondrial** | *Phaseolus vulgaris* | VVDALGVPIDGR | Unmodified | 2 | 1209.67 | 0.93 | 287400 |
|  |  |  | VVSVGDGIAR | Unmodified | 2 | 971.54 | 0.31 | 424260 |
| **F4J339** | **Probable disease resistance protein RPP1** | *Arabidopsis thaliana* | KLWEGTKQLR | Di-oxidation W | 2 | 1289.71 | 1.12 | 399950 |
|  |  |  | RTFLSHIMESFR | Unmodified | 3 | 1522.77 | 1.24 | 142690 |
| **P43295** | **Probable cysteine protease RD19B** | *Arabidopsis thaliana* | ANLLRAMRHQK | Oxidation (M),Deamidation (NQ) | 2 | 1353.73 | 3.62 | 765160 |
|  |  |  | QVVDETEPKVLSSEDHFTLFK | Acetyl (K),Deamidation (NQ) | 3 | 2490.22 | 0.16 | 10162000 |
| **Q10E64** | **Kinesin-like protein KIN-8B** | *Oryza sativa* | ILKDGLSGNSR | Deamidation (NQ) | 2 | 1159.62 | -0.04 | 710660 |
|  |  |  | MIDNLQVEVSQLK | Oxidation (M) | 2 | 1531.79 | 3.96 | 7373200 |
| **Q1KVQ9** | **Uncharacterized membrane protein ycf78** | *Tetradesmus obliquus* | DQMTNAIEQNNEK | 3 Deamidation (NQ) | 2 | 1536.62 | 3.03 | 2388000 |
|  |  |  | ENLNVSDLQENK | Glu->pyro-Glu,Deamidation (NQ) | 2 | 1384.65 | 0.50 | 563200 |
| **Q5SCY7** | **Protein TIC 214** | *Huperizia lucidula* | NNDIFEFEPDDKDK | Acetyl (K),2 Deamidation (NQ) | 2 | 1768.73 | 1.34 | 207670 |
|  |  |  | QIMLNISKRNNK | Oxidation (M),2 Deamidation (NQ) | 2 | 1475.78 | 2.07 | 765860000 |
| **Q7PCJ6** | **DNA-directed RNA polymerase subunit beta** | *Chlamydomonas reinhardtii* | NATINVLIKPQQEK | Deamidation (NQ) | 3 | 1595.89 | -1.10 | 468290 |
|  |  |  | NYLDLNPYQSLK | 2 Deamidation (NQ),2 dioxidation Y | 2 | 1532.69 | -2.40 | 444470 |
| **Q84JZ8** | **Protein tesmin/TSO1-like CXC 4** | *Arabidopsis thaliana* | EDKDLQPSGKQR | Glu->pyro-Glu,Deamidation (NQ) | 2 | 1382.68 | 0.34 | 4384400 |
|  |  |  | QLSQGLDELGSCKR | Deamidation (NQ) | 2 | 1590.77 | -1.35 | 613380 |
| **Q8MA10** | **DNA-directed RNA polymerase subunit beta** | *Chaetosphaeridium globosum* | DLSLDINTNLLK | Unmodified | 2 | 1357.75 | 0.53 | 1091800 |
|  |  |  | KLNFYQK | Oxidation Y | 2 | 955.51 | -2.98 | 325720 |
| **Q9MA55** | **Acyl-CoA-binding domain-containing protein 4** | *Arabidopsis thaliana* | ELQSVRGQLAAEQSR | Deamidation (NQ) | 3 | 1671.85 | 0.98 | 340590 |
|  |  |  | YNNEINLLK | Deamidation (NQ),dioxidation Y | 2 | 1152.57 | -0.25 | 1692500 |
| **Q9MTJ3** | **DNA-directed RNA polymerase subunit alpha** | *Oenothera elata subsp. hookeri* | SIFIDQLELPPR | Unmodified | 2 | 1426.78 | -2.26 | 7680800 |
|  |  |  | VDSKRLYYGR | Iodination,Oxidation Y | 2 | 1397.56 | 1.23 | 1089200 |
| **Q9SM50** | **Transcription factor HY5** | *Solanum lycopersicum* | KAYLIDLEAR | O-2H (Y) | 2 | 1204.65 | 3.86 | 79990 |
|  |  |  | VSAQQARERK | 2 Deamidation (NQ) | 2 | 1173.61 | 1.51 | 389590 |
| A0A075FBG7 | 9,13-epoxylabda-14-ene synthase, chloroplastic | *Marrubium vulgare* | EKVGGKADK | Unmodified | 2 | 930.51 | -0.33 | 13259000 |
|  |  |  | VGGKADK | Unmodified | 1 | 673.38 | -0.63 | 18161000 |
| **P11143** | **Heat shock proteins** | *Zea mays* | IINEPTAAAIAYGLDK | Unmodified | 2 | 1658,89 | -0,33 | 612010 |
|  |  |  | IINEPTAAAIAYGLDKK | Unmodified | 3 | 1786,98 | -1,17 | 391370 |
|  |  |  | NQVAMNPTNTVFDAK | Oxidation (M) | 2 | 1664,78 | 0,10 | 257990 |
|  |  |  | TTPSYVAFTDTER | Unmodified | 2 | 1486,69 | 0,78 | 433730 |
|  |  |  | VEIIANDQGNR | Unmodified | 2 | 1227,62 | 0,54 | 489420 |
| **G3T386** | **Actin** | *Mesostigma viridae* | AGFAGDDAPR | Unmodified | 2 | 975,44 | -0,08 | 1435300 |
|  |  |  | GYSFTTTAER | Unmodified | 2 | 1131,52 | 0,69 | 1342100 |
|  |  |  | SYELPDGQVITIGNER | Unmodified | 2 | 1789,88 | 0,92 | 752110 |
|  |  |  | VAPEEHPVLLTEAPLNPK | Unmodified | 3 | 1953,06 | 0,03 | 916470 |
| B3TN96 | NAD(P)H-quinone oxidoreductase subunit 5 | *Brachypodium distachyon* | SQNMALMGGLRK | Oxidation (M) | 2 | 1320.66 | 0.03 | 6203200 |
|  |  |  | SQNMALMGGLRK | Oxidation (M),Deamidation (NQ) | 2 | 1321.65 | 3.87 | 915900 |
| A3BDI8 | Zinc finger A20 and AN1 domain-containing stress-associated protein 8 | *Oryza sativa subsp. japonica* | CHKEMIMKQEQAK | Acetyl (K),Oxidation (M),Trioxidation (C) | 2 | 1765.78 | -0.31 | 3254100 |
| A6H5F7 | DNA-directed RNA polymerase subunit beta'', EC 2.7.7.6 (PEP) | *Cycas taitungensis* | ENYLGDQRAR | Deamidation (NQ),dioxidation Y | 2 | 1253.56 | -1.73 | 266350 |
| A7UL74 | Protein CHROMOSOME TRANSMISSION FIDELITY 7, EC 2.3.1. | *Arabidopsis thaliana* | DYMYGLPFK | Oxidation (M),Oxidation Y,O-2H (Y) | 2 | 1178.50 | -3.51 | 179140 |
| B2X1Z4 | DNA-directed RNA polymerase subunit beta'', EC 2.7.7.6 (PEP) | *Oedogonium cardiacum* | EIVVNFNYLKK | Oxidation Y | 2 | 1381.76 | -3.41 | 1714400 |
| B2Y1W2 | ATP synthase subunit alpha, chloroplastic, EC 7.1.2.2 | *Welwitschia mirabilis* | EQTELFVIQQKN | Glu->pyro-Glu | 2 | 1457.75 | -0.02 | 958330 |
| B6TVL4 | Calcium sensing receptor, chloroplastic (Sulfurtransferase 3) | *Zea mays* | GSNVIIMDSYSDVAKTVAK | Oxidation (M),dioxidation Y | 2 | 2045.00 | -3.64 | 500790 |
| B9F058 | Acyl-[acyl-carrier-protein] desaturase 3, chloroplastic, EC 1.14.19.- | *Oryza sativa subsp. japonica* | APPAAAAPFSWIHGR | Trp->Kynurenine | 2 | 1551.79 | -2.53 | 1323400 |
| C0LGQ7 | Probable LRR receptor-like serine/threonine-protein kinase At4g20450, EC 2.7.11.1 | *Arabidopsis thaliana* | QHLSGENSRSPLSWENR | 2 Deamidation (NQ),O-2H (W) | 2 | 2011.90 | -1.78 | 1471200 |
| F4HVY0 | Very-long-chain aldehyde decarbonylase CER1, EC 4.1.99.5 (Protein ECERIFERUM 1) | *Arabidopsis thaliana* | VLSLGLMNQGEELNR | Oxidation (M),3 Deamidation (NQ) | 2 | 1690.81 | -3.09 | 371920 |
| F4I9Q5 | DExH-box ATP-dependent RNA helicase DExH7, chloroplastic, EC 3.6.4.13 | *Arabidopsis thaliana* | SLIEKQSCDNTSR | Deamidation (NQ),Trioxidation (C) | 2 | 1585.69 | 3.91 | 113960 |
| F4J7T2 | Chromatin modification-related protein EAF1 B (ESA1-associated factor 1 B) | *Arabidopsis thaliana* | SPQSGTSGVNNQAGKQRQR | 3 Deamidation (NQ) | 2 | 2001.95 | -0.98 | 176380 |
| F4JLS1 | Lysine-specific histone demethylase 1 homolog 3, EC 1 | *Arabidopsis thaliana* | QANTTNTSRIR | 2 Deamidation (NQ) | 2 | 1262.62 | -3.83 | 314960 |
| F4JSG3 | Replication protein A 70 kDa DNA-binding subunit E, AtRPA70E | *Arabidopsis thaliana* | KVAFTKYNFK | Deamidation (NQ) | 2 | 1245.68 | -2.10 | 350200 |
| F4K4L7 | Transcription initiation factor TFIID subunit 4b (TBP-associated factor 4b, AtTAF4b) | *Arabidopsis thaliana* | LQKMSSQQAR | 3 Deamidation (NQ) | 2 | 1178.56 | -3.14 | 211200 |
| F4K5T4 | Probable transcription factor At5g28040 (Storekeeper-like protein At5g28040) | *Arabidopsis thaliana* | LELVQEQIR | 2 Deamidation (NQ) | 2 | 1128.60 | 3.46 | 1985500 |
| G3CCC0 | Copal-8-ol diphosphate hydratase, chloroplastic, EC 4.2.1.133 (8-hydroxy-copalyl diphosphate synthase) | *Nicotiana tabacum* | DDLDWQRLLK | Deamidation (NQ),O-2H (W) | 2 | 1315.64 | 1.16 | 156980 |
| G3ESU9 | Acyl-[acyl-carrier-protein] hydrolase FATB1, chloroplastic, CvFatB1, EC 3.1.2. | *Cuphea viscosissima* | NDLIWVLTK | O-2H (W) | 2 | 1114.60 | -3.24 | 3855800 |
| G7ZZZ3 | CLAVATA3/ESR (CLE)-related protein 12, MtCLE12 [Cleaved into: CLE12p] | *Medicago truncatula* | LEGGSNIDSQRLLHELMVDR | Deamidation (NQ) | 3 | 2282.13 | -2.07 | 367620 |
| H2DF88 | Acid beta-fructofuranosidase 2, vacuolar, EC 3.2.1.26 (Vacuolar invertase 2, RvVI2) | *Rosa hybrid cultivar* | TTITTRVYPTQAIYGAAR | Iodination,Oxidation Y | 2 | 2123.95 | 1.17 | 146060 |
| H3K2Y6 | Mediator of RNA polymerase II transcription subunit 12 | *Arabidopsis thaliana* | FEFLSFDYTISTIQR | Deamidation (NQ) | 3 | 1866.90 | 2.14 | 528850 |
| J9QS25 | (E)-2-epi-beta-caryophyllene synthase, EC 4.2.3.137 (Microbial Terpene synthase-like protein 26, SmMTPSL26) | *Selaginella moellendorffii* | MEDVLAEKLSR | Acetyl (K),Oxidation (M) | 2 | 1347.67 | -4.01 | 128240 |
| O03073 | ATP synthase subunit beta, chloroplastic, EC 7.1.2.2 (ATP synthase F1 sector subunit beta) (F-ATPase subunit beta) | *Lonchitis hirsuta* | FVQAGSEVSALLGR | Deamidation (NQ) | 2 | 1433.75 | 1.13 | 115640 |
| O04336 | Probable WRKY transcription factor 21 (WRKY DNA-binding protein 21) | *Arabidopsis thaliana* | KLQSHVSQSLLLDPCQQR | 2 Deamidation (NQ) | 3 | 2138.08 | -0.17 | 2270200 |
| O22437 | Magnesium-chelatase subunit ChlD, chloroplastic, Mg-chelatase subunit D, EC 6.6.1.1 | *Pisum sativum* | LDASNYGR | Oxidation Y | 1 | 910.41 | 0.13 | 241560 |
| O23342 | Preprotein translocase subunit SECE1 | *Arabidopsis thaliana* | EIEWPAFQK | Deamidation (NQ),Di-oxidation W | 2 | 1179.54 | -1.58 | 69413 |
| O49289 | Putative DEAD-box ATP-dependent RNA helicase 29, EC 3.6.4.13 | *Arabidopsis thaliana* | YIKLNNGDR | Deamidation (NQ) | 2 | 1092.56 | -1.16 | 854240 |
| O49840 | Probable serine/threonine-protein kinase PBL3, EC 2.7.11.1 (PBS1-like protein 3) (Protein kinase 2B) | *Arabidopsis thaliana* | GAYTAASLALQCLNPDAK | Deamidation (NQ),Trioxidation (C) | 2 | 1911.89 | -3.15 | 4320400 |
| O64790 | Probable disease resistance protein At1g61300 | *Arabidopsis thaliana* | LLIIHVLDCPKLRK | Acetyl (K),Trioxidation (C) | 2 | 1807.04 | 0.21 | 484990 |
| O65202 | Peroxisomal acyl-coenzyme A oxidase 1, AOX 1, EC 1.3.3.6 (Long-chain acyl-CoA oxidase, AtCX1) | *Arabidopsis thaliana* | LVASDPVFEKSNR | Acetyl (K) | 2 | 1502.77 | -1.49 | 4854600 |
| O65373 | BAG family molecular chaperone regulator 5, mitochondrial (Bcl-2-associated athanogene 5) | *Arabidopsis thaliana* | KISSINREANR | 2 Deamidation (NQ) | 2 | 1288.67 | -1.06 | 183460 |
| O65440 | Leucine-rich repeat receptor-like serine/threonine-protein kinase BAM3, EC 2.7.11.1 (Protein BARELY ANY MERISTEM 3) | *Arabidopsis thaliana* | GVMPNGEEVAVKK | Oxidation (M) | 2 | 1372.70 | 0.34 | 106840 |
| O80337 | Ethylene-responsive transcription factor 1A, AtERF1A (Ethylene-responsive element-binding factor 1A, EREBP-1A) | *Arabidopsis thaliana* | NPSFSKLYPCFTESWGDLPLK | Oxidation (W) | 3 | 2501.19 | -1.60 | 280710 |
| O80458 | Dehydrodolichyl diphosphate synthase 1, Dedol-PP synthase 1, EC 2.5.1.87 | *Arabidopsis thaliana* | LIGLIKIKAAR | 2 Acetyl (K) | 2 | 1278.84 | -0.84 | 556810 |
| O82150 | ATP-dependent zinc metalloprotease FTSH, chloroplastic, EC 3.4.24.- (DS9) | *Nicotiana tabacum* | KSLIPQSILNKK | 2 Acetyl (K) | 2 | 1451.87 | 1.28 | 266860 |
| P05493 | ATP synthase subunit alpha, mitochondrial | *Pisum sativum* | AAELTTLLESR | Unmodified | 2 | 1202.65 | 2.03 | 239770 |
| P0C5D6 | Serine/threonine-protein kinase SAPK3, EC 2.7.11.1 (Osmotic stress/abscisic acid-activated protein kinase 3) | *Oryza sativa subsp. japonica* | ILGVQYSIPDYVR | Deamidation (NQ),Oxidation Y,O-2H (Y) | 2 | 1552.78 | -2.99 | 307840 |
| P0CB22 | Histone-lysine N-methyltransferase ATX2, EC 2.1.1. | *Arabidopsis thaliana* | IYGSKMSQITTPSNILSMAEK | Deamidation (NQ) | 3 | 2299.14 | 1.40 | 2380100 |
| P12300 | Glucose-1-phosphate adenylyltransferase large subunit, chloroplastic/amyloplastic, EC 2.7.7.27 | *Triticum aestivum* | VPIGVGENTKISNCIIDMNAR | Deamidation (NQ) | 3 | 2301.15 | -1.39 | 1417700 |
| P14232 | TGACG-sequence-specific DNA-binding protein TGA-1A, ASF-1 protein, TGA1a | *Nicotiana tabacum* | VLSSQWATR | O-2H (W) | 2 | 1060.53 | 4.41 | 758900 |
| P19366 | ATP synthase subunit beta, chloroplastic, EC 7.1.2.2 | *Arabidopsis thaliana* | GIYPAVDPLDSTSTMLQPR | Oxidation (M),Deamidation (NQ) | 2 | 2077.00 | 2.10 | 417960 |
| P25413 | Ribulose bisphosphate carboxylase large chain, RuBisCO large subunit, EC 4.1.1.39 | *Aegilops crassa* | EMTLGFVDLLR | Oxidation (M) | 2 | 1308.67 | -3.21 | 401000 |
| P26846 | NADH-ubiquinone oxidoreductase chain 2, EC 7.1.1.2 (NADH dehydrogenase subunit 2) | *Marchantia polymorpha* | TWVLYKPMDR | Oxidation (M) | 2 | 1323.66 | 2.91 | 232450 |
| P31163 | 50S ribosomal protein L2, chloroplastic | *Pisum sativum* | LISGQHHCGKGRNPR | Deamidation (NQ) | 2 | 1716.86 | -3.27 | 1174600 |
| P32733 | Kunitz-type trypsin inhibitor alpha chain | *Prosopis juliflora* | NGGSYYILPAFRGK | Deamidation (NQ),dioxidation Y | 2 | 1574.77 | -1.11 | 81394 |
| P34924 | Glyceraldehyde-3-phosphate dehydrogenase, cytosolic, EC 1.2.1.12 | *Pinus sylvestris* | VPTPDVSVVDLTVR | Unmodified | 2 | 1495.82 | -1.38 | 672250 |
| P36495 | Uncharacterized membrane protein ycf78 (ORF-S) (ORF1995) (ORFA) | *Chlamydomonas reinhardtii* | KQLNNPANPSLETSTK | 2 Deamidation (NQ) | 2 | 1742.87 | -1.68 | 130650 |
| P38500 | Ferredoxin--nitrite reductase, chloroplastic, EC 1.7.7.1 | *Betula pendula* | PYNNLLSQFITANSRGNLAFTNLPRK | Deamidation (NQ) | 3 | 2949.55 | 3.02 | 3723800 |
| P49969 | Signal recognition particle 54 kDa protein 2, SRP54 | *Hordeum vulgare* | MVRDMQTNIRK | Oxidation (M),Deamidation (NQ) | 2 | 1407.70 | 3.86 | 161770 |
| P51567 | Serine/threonine-protein kinase AFC2, EC 2.7.12.1 | *Arabidopsis thaliana* | LDWPDGATSRDSLK | Oxidation (W) | 2 | 1575.75 | -0.43 | 476190 |
| P53780 | Cystathionine beta-lyase, chloroplastic, CBL, EC 4.4.1.13 (Beta-cystathionase) (Cysteine lyase) | *Arabidopsis thaliana* | VPTWEKKQISNR | 2 Deamidation (NQ),Trp->Kynurenine | 2 | 1490.77 | -0.15 | 524340 |
| P53991 | Ferredoxin--NADP reductase, chloroplastic, FNR, EC 1.18.1.2 | *Chlamydomonas reinhardtii* | INSKGKEVPTAR | Acetyl (K),Deamidation (NQ) | 2 | 1341.73 | 1.08 | 485460 |
| P82869 | Peptidyl-prolyl cis-trans isomerase CYP37, chloroplastic, PPIase CYP37, EC 5.2.1 | *Arabidopsis thaliana* | LSLPVQNNNINEST | 3 Deamidation (NQ) | 2 | 1544.72 | -2.39 | 233680 |
| P85190 | ATP-dependent zinc metalloprotease FTSH, chloroplastic, EC 3.4.24.- | *Helianthus annuus* | LLAGQPR | Deamidation (NQ) | 1 | 754.43 | 1.21 | 186130 |
| Q01909 | ATP synthase gamma chain 2, chloroplastic (F-ATPase gamma subunit 2) | *Arabidopsis thaliana* | KNLTMAYNRAR | Oxidation (M),dioxidation Y | 2 | 1384.69 | -2.85 | 254870 |
| Q04648 | Probable cytochrome c biosynthesis protein | *Oenothera berteroana* | AQPRPQLLWKN | 2 Deamidation (NQ),Trp->Kynurenine | 2 | 1355.72 | 0.68 | 4175700 |
| Q06215 | Polyphenol oxidase A1, chloroplastic, PPO, EC 1.10.3.1 (Catechol oxidase) | *Vicia faba* | LVEVEVNDGNLRK | Deamidation (NQ) | 2 | 1484.78 | -1.34 | 1119200 |
| Q06FM0 | 30S ribosomal protein S7, chloroplastic | *Pelargonium hortorum* | SLAYKILYR | 2 dioxidation Y | 2 | 1189.63 | -3.02 | 195190 |
| Q06SJ3 | 30S ribosomal protein S4, chloroplastic | *Stigeoclonium helveticum* | NLLNNQFQLLQKLK | 2 Deamidation (NQ) | 2 | 1714.96 | 1.14 | 446350 |
| Q09G44 | 30S ribosomal protein S4, chloroplastic | *Platanus occidentalis* | ALIQNYLDSSSHEELPK | 2 Deamidation (NQ),Oxidation Y | 2 | 1960.93 | -1.45 | 316770 |
| Q0D3S3 | Probable GTP-binding protein OBGC1, chloroplastic | *Oryza sativa subsp. japonica* | PYIVVYNK | Iodination,O-2H (Y) | 2 | 1134.42 | -2.70 | 239730 |
| Q0DJC5 | Dolichyl-diphosphooligosaccharide--protein glycosyltransferase subunit 1A (Ribophorin IA, RPN-IA) (Ribophorin-1A) | *Oryza sativa subsp. japonica* | TDMSISKNSPSYLAK | Deamidation (NQ),dioxidation Y | 2 | 1673.78 | 2.06 | 2915000 |
| Q0G9X2 | DNA-directed RNA polymerase subunit beta'', EC 2.7.7.6 (PEP) | *Daucus carota* | TCLVLNCTQDKK | Deamidation (NQ),Trioxidation (C) | 2 | 1527.69 | -1.12 | 1705100 |
| Q1KVT8 | 30S ribosomal protein S19, chloroplastic | *Tetradesmus obliquus* | KIEKFNAQGQK | 2 Deamidation (NQ) | 2 | 1291.68 | 0.37 | 327200 |
| Q1KVX8 | DNA-directed RNA polymerase subunit beta'', EC 2.7.7.6 (PEP) | *Tetradesmus obliquus* | KASEISTIQK | Acetyl (K) | 2 | 1145.63 | 0.63 | 1225400 |
| Q1PDV2 | Serine/arginine-rich SC35-like splicing factor SCL28, At-SCL28, AtSCL28 | *Arabidopsis thaliana* | FGPLKDIYLPRNYYTGEPR | Deamidation (NQ) | 3 | 2299.16 | -4.32 | 259580 |
| Q1PFQ9 | Pentatricopeptide repeat-containing protein At1g28690, mitochondrial | *Arabidopsis thaliana* | ILPSNHYSTFPLK | Deamidation (NQ),Oxidation Y | 2 | 1532.79 | -1.27 | 337180 |
| Q20EV9 | ATP synthase subunit alpha, chloroplastic, EC 7.1.2.2 (ATP synthase F1 sector subunit alpha) (F-ATPase subunit alpha) | *Oltmannsiellopsis viridis* | TAIALDTILNQK | Unmodified | 2 | 1299.74 | 1.09 | 224600 |
| Q2L8Z4 | DNA-directed RNA polymerase subunit beta', EC 2.7.7.6 (PEP) | *Gossypium hirsutum* | YNPWNRK | Trp->Kynurenine | 2 | 980.48 | -1.72 | 650290 |
| Q2R9D2 | B3 domain-containing protein Os11g0197600 | *Oryza sativa subsp. japonica* | ITLWDPQGK | Deamidation (NQ),Di-oxidation W | 2 | 1089.53 | -3.89 | 2086800 |
| Q32RY2 | DNA-directed RNA polymerase subunit beta'', EC 2.7.7.6 (PEP) | *Staurastrum punctulatum* | NYSIAEK | O-2H (Y) | 1 | 837.39 | 0.25 | 957130 |
| Q338B0 | Heat stress transcription factor A-2c | *Oryza sativa subsp. japonica* | NPEFFQQLAQQK | Deamidation (NQ) | 3 | 1477.72 | -2.58 | 724570 |
| Q338N2 | Zinc finger CCCH domain-containing protein 62, OsC3H62 | *Oryza sativa subsp. japonica* | QSNNWGSTDHDK | 2 Deamidation (NQ),O-2H (W) | 2 | 1403.52 | -0.57 | 215610 |
| Q38861 | General transcription and DNA repair factor IIH helicase subunit XPB1, TFIIH subunit XPB, EC 3.6.4.12 | *Arabidopsis thaliana* | LELKPDHGNR | Acetyl (K),Deamidation (NQ) | 2 | 1220.61 | 0.40 | 1006900 |
| Q41771 | Ent-copalyl diphosphate synthase AN1, chloroplastic, Ent-CPP synthase, Ent-CPS, EC 5.5.1.13 | *Zea mays* | MINTLACVVALTK | Oxidation (M),Trioxidation (C) | 2 | 1496.76 | 0.89 | 5362200 |
| Q42536 | Protochlorophyllide reductase A, chloroplastic, PCR A, EC 1.3.1.33 (NADPH-protochlorophyllide oxidoreductase A, POR A) | *Arabidopsis thaliana* | LNASASSSFKESSLFGVSLSEQSK | Deamidation (NQ) | 3 | 2490.21 | 0.71 | 10162000 |
| Q42662 | 5-methyltetrahydropteroyltriglutamate--homocysteine methyltransferase, EC 2.1.1.14 | *Plectranthus scutellarioides* | AGITVIQIDEAALR | Unmodified | 2 | 1468.82 | -0.90 | 192560 |
| Q42877 | DNA-directed RNA polymerase II subunit RPB2, RNA polymerase II subunit 2, RNA polymerase II subunit B2, EC 2.7.7.6 | *Solanum lycopersicum* | IKYAKEILQK | Deamidation (NQ) | 2 | 1233.73 | 3.16 | 96741 |
| Q43092 | Granule-bound starch synthase 1, chloroplastic/amyloplastic, EC 2.4.1.242 (Granule-bound starch synthase I, GBSS-I) | *Pisum sativum* | QIILNCMAQNFSWKKPAK | Trioxidation (C) | 3 | 2224.11 | 1.82 | 2127800 |
| Q4VZL1 | 30S ribosomal protein S15, chloroplastic | *Cucumis sativus* | ELINQLDIRESK | 2 Deamidation (NQ) | 3 | 1458.76 | 0.42 | 2530300 |
| Q4VZP3 | DNA-directed RNA polymerase subunit beta'', EC 2.7.7.6 (PEP) | *Cucumis sativus* | VPQSPSQNQGTIR | 3 Deamidation (NQ) | 2 | 1413.67 | 1.62 | 200570 |
| Q53LQ0 | Protein disulfide isomerase-like 1-1, OsPDIL1-1, EC 5.3.4.1 (Endosperm storage protein 2, Protein ESP2) | *Oryza sativa subsp. japonica* | NQGKNIQEYK | Deamidation (NQ) | 2 | 1221.60 | 1.82 | 661190 |
| Q570C0 | Protein TRANSPORT INHIBITOR RESPONSE 1 (Weak ethylene-insensitive protein 1) | *Arabidopsis thaliana* | LLGQKMPKLNVEVIDER | 2 Acetyl (K) | 2 | 2065.12 | 2.42 | 502240 |
| Q5XF04 | Probable xyloglucan galactosyltransferase GT15, EC 2.4.1.- (Glycosyltransferase 15, AtGT15) | *Arabidopsis thaliana* | PKDKISMCK | Acetyl (K),Trioxidation (C) | 2 | 1195.56 | -2.54 | 1087400 |
| Q5XF07 | DNA-(apurinic or apyrimidinic site) endonuclease, EC 3.1.-.- (APEX1-like protein) | *Arabidopsis thaliana* | NDWSQFSK | Deamidation (NQ),Trp->Kynurenine | 2 | 1015.42 | 3.13 | 785860 |
| Q652N5 | Probable anion transporter 4, chloroplastic (Phosphate transporter 4;4) | *Oryza sativa subsp. japonica* | LTTRTTIFQVSNYSR | Deamidation (NQ) | 2 | 1786.92 | 0.73 | 1186100 |
| Q6L3Z0 | Putative late blight resistance protein homolog R1B-13 | *Solanum demissum* | VEDAELMLRK | Oxidation (M) | 2 | 1218.63 | -2.04 | 5018400 |
| Q6YVX9 | Zinc finger CCCH domain-containing protein 16, OsC3H16 | *Oryza sativa subsp. japonica* | NPGGDYEWADWDNPPPRYWIR | 2 Deamidation (NQ),2 Trp->Kynurenine | 3 | 2613.12 | -3.80 | 637060 |
| Q6Z9U7 | mRNA cap guanine-N7 methyltransferase 1, EC 2.1.1.56 (mRNA (guanine-N(7)-)-methyltransferase 1) (mRNA cap methyltransferase 1) | *Oryza sativa subsp. japonica* | SSARRVADHYSAR | Oxidation Y | 2 | 1490.73 | 2.89 | 5220600 |
| Q75NZ0 | Sulfite reductase [ferredoxin], chloroplastic, PsSiR, EC 1.8.7.1 | *Pisum sativum* | NVVEEYYGKK | O-2H (Y) | 2 | 1241.59 | -4.13 | 200340 |
| Q7DMA9 | Peptidyl-prolyl cis-trans isomerase PASTICCINO1, EC 5.2.1.8 | *Arabidopsis thaliana* | KILNEGEGWESPR | Deamidation (NQ),Trp->Kynurenine | 2 | 1518.73 | 0.78 | 1654300 |
| Q7X659 | Vacuolar protein sorting-associated protein 35A (Protein ZIG SUPPRESSOR 3) (Vesicle protein sorting 35A) | *Arabidopsis thaliana* | LLKKPDQCR | Deamidation (NQ) | 2 | 1157.62 | 1.33 | 684060 |
| Q7X6P3 | Protein root UVB sensitive 1, chloroplastic (Protein WEAK AUXIN RESPONSE 3) | *Arabidopsis thaliana* | NEGYILTEHKGRFCVMLK | Oxidation (M) | 3 | 2210.10 | 0.67 | 8046400 |
| Q7XHR2 | Calmodulin-binding transcription activator CBT (CaM-binding transcription factor, OsCBT) | *Oryza sativa subsp. japonica* | AQQEYRR | Deamidation (NQ) | 2 | 950.46 | -0.39 | 954520 |
| Q7XKA8 | Serine/threonine-protein kinase SAPK5, EC 2.7.11.1 (Osmotic stress/abscisic acid-activated protein kinase 5) | *Oryza sativa subsp. japonica* | IVQEAQTVPK | Deamidation (NQ) | 2 | 1112.61 | 1.42 | 148240 |
| Q7XPZ4 | Very-long-chain aldehyde decarbonylase GL1-7, EC 4.1.99.5 (Protein GLOSSY 1-7) | *Oryza sativa subsp. japonica* | FSMQVWALPR | Oxidation (M),Deamidation (NQ),Trp->Kynurenine | 2 | 1254.61 | 1.43 | 355710 |
| Q7XQP4 | Serine/threonine-protein kinase SAPK7, EC 2.7.11.1 (Osmotic stress/abscisic acid-activated protein kinase 7) | *Oryza sativa subsp. japonica* | LMRNKETK | Acetyl (K),Deamidation (NQ),di-oxidation (M) | 2 | 1093.54 | -3.03 | 356750 |
| Q84JL5 | Probable alkaline/neutral invertase A, chloroplastic, A/N-INVA, EC 3.2.1.26 | *Arabidopsis thaliana* | YIRDTSFRSYPSR | Iodination | 2 | 1772.71 | 1.44 | 30132000 |
| Q84ND9 | DNA polymerase I B, chloroplastic/mitochondrial, EC 2.7.7.7 (DNA polymerase PolI-like B, AtPolI-like B) | *Arabidopsis thaliana* | DYLAQIEIVAKAEQEIAVSR | Deamidation (NQ) | 3 | 2246.18 | 1.53 | 271830 |
| Q84VG6 | Pentatricopeptide repeat-containing protein At2g17525, mitochondrial | *Arabidopsis thaliana* | CFSLGFVPDVVTVTK | Trioxidation (C) | 2 | 1715.84 | 0.90 | 164770 |
| Q84W55 | Type II inositol polyphosphate 5-phosphatase 15, At5PTase15, EC 3.1.3.36, EC 3.1.3.86 (Protein FRAGILE FIBER 3) | *Arabidopsis thaliana* | AQSSLGFFQRSR | 2 Deamidation (NQ) | 2 | 1384.67 | -0.70 | 525260 |
| Q84Y95 | Monothiol glutaredoxin-S14, chloroplastic, AtGRXcp, AtGrxS14 (CAX-interacting protein 1, CXIP1) | *Arabidopsis thaliana* | LVNSEKVVLFMKGTR | Oxidation (M) | 2 | 1735.97 | -1.75 | 133640 |
| Q8GY79 | Double-stranded RNA-binding protein 5 (dsRNA-binding protein 5, AtDRB5) | *Arabidopsis thaliana* | ETTNQWRRR | Glu->pyro-Glu,Deamidation (NQ) | 2 | 1228.61 | 3.01 | 260350 |
| Q8GZQ3 | Polyribonucleotide nucleotidyltransferase 1, chloroplastic, AtcpPNPase, EC 2.7.7.8 (Polynucleotide phosphorylase 1, PNPase 1) | *Arabidopsis thaliana* | QMAQRIDNLEGSDEYKR | Acetyl (K),2 Deamidation (NQ) | 3 | 2095.95 | 3.34 | 1113800 |
| Q8H038 | Xyloglucan galactosyltransferase KATAMARI1 homolog, EC 2.4.1.- | *Oryza sativa subsp. japonica* | LSLWTNMCK | Oxidation (M),Deamidation (NQ),Di-oxidation W | 2 | 1200.52 | -3.74 | 42492 |
| Q8H0B6 | Probable UDP-arabinose 4-epimerase 2, EC 5.1.3.5 (OsUEL-2) | *Oryza sativa subsp. japonica* | DNYRVTIVDNLSRGNMGAVR | Deamidation (NQ),Oxidation Y | 2 | 2266.11 | 4.05 | 411890 |
| Q8L4B0 | Probable galacturonosyltransferase 15, EC 2.4.1.- | *Arabidopsis thaliana* | SPQEILK | Deamidation (NQ) | 1 | 814.44 | 1.50 | 389810 |
| Q8L6J3 | DNA-directed RNA polymerase 2B, chloroplastic/mitochondrial, EC 2.7.7.6 | *Nicotiana tabacum* | YHNSPIMWRNIIK | Deamidation (NQ) | 3 | 1671.86 | 0.98 | 340590 |
| Q8L9T7 | Histidine-containing phosphotransfer protein 5 | *Arabidopsis thaliana* | QILQAGGTIPQVDIN | 3 Deamidation (NQ) | 2 | 1568.79 | 2.66 | 389760 |
| Q8LCS8 | Membrane-anchored ubiquitin-fold protein 2, AtMUB2, Membrane-anchored ub-fold protein 2 (NTGP5) | *Arabidopsis thaliana* | ILENNKTVGDCR | Acetyl (K),Trioxidation (C) | 2 | 1507.69 | 2.09 | 182180 |
| Q8RY24 | Probable sucrose-phosphate synthase 3, EC 2.4.1.14 | *Arabidopsis thaliana* | KQLEWEDSQR | Deamidation (NQ) | 2 | 1318.62 | -2.52 | 1947100 |
| Q8S1X8 | Probable glucuronosyltransferase Os01g0926600, EC 2.4.-.- | *Oryza sativa subsp. japonica* | VYVYELPTKYNK | dioxidation Y,O-2H (Y) | 2 | 1561.77 | 0.00 | 351030 |
| Q8VYR0 | Calcium uniporter protein 5, mitochondrial | *Arabidopsis thaliana* | KDMTVLEAK | Oxidation (M) | 2 | 1049.54 | -3.86 | 1310100 |
| Q8VZC9 | Vacuolar protein sorting-associated protein 25, AtVPS25 (ESCRT-II complex subunit VPS25) | *Arabidopsis thaliana* | AEWLDKGHR | O-2H (W) | 2 | 1124.54 | 0.44 | 227250 |
| Q8W3K0 | Probable disease resistance protein At1g58602 | *Arabidopsis thaliana* | LSEGGEDYYK | Oxidation Y,dioxidation Y | 2 | 1207.49 | 1.55 | 109740 |
| Q8W3M6 | Ubiquitin-like domain-containing CTD phosphatase, EC 3.1.3.16 | *Arabidopsis thaliana* | IDQYKINLR | 2 Deamidation (NQ) | 2 | 1163.62 | 0.36 | 482900 |
| Q93WD2 | Chlorophyll a-b binding protein CP29 (Lhcbm4) | *Chlamydomonas reinhardtii* | LAPYSEVFGLAR | dioxidation Y | 2 | 1353.69 | 4.21 | 290800 |
| Q943K1 | Photosystem II 22 kDa protein 1, chloroplastic, 22 kDa protein of photosystem II 1 | *Oryza sativa subsp. japonica* | ENELFVGR | Unmodified | 2 | 962.48 | 0.26 | 336570 |
| Q96542 | Ribulose bisphosphate carboxylase small subunit, chloroplastic, RuBisCO small subunit | *Betula pendula* | VQCMQVWPPLGLK | Oxidation (M),Deamidation (NQ),Trioxidation (C) | 2 | 1619.77 | -1.63 | 418020 |
| Q9C6B6 | ERAD-associated E3 ubiquitin-protein ligase component HRD3B (AtSel1B) | *Arabidopsis thaliana* | YLRQNMYDK | Oxidation Y,dioxidation Y | 2 | 1277.57 | -1.91 | 1449000 |
| Q9C6L8 | Aluminum-activated malate transporter 4, AtALMT4 | *Arabidopsis thaliana* | MADQTREAFLSR | Oxidation (M),Deamidation (NQ) | 2 | 1440.67 | -4.06 | 226730 |
| Q9FFF2 | Ubiquitin carboxyl-terminal hydrolase, EC 3.4.19.12 | *Arabidopsis thaliana* | IVMEEEKSKNWK | Oxidation (W) | 2 | 1535.77 | -4.12 | 492810 |
| Q9FGE9 | Protein trichome birefringence-like 12 | *Arabidopsis thaliana* | RDNMDVINSWRWEPNGCGLSR | Oxidation (M),Deamidation (NQ) | 3 | 2578.14 | -1.97 | 1208400 |
| Q9FHD5 | Cysteine-rich repeat secretory protein 57 | *Arabidopsis thaliana* | LSDKMGEVIIR | Acetyl (K),Oxidation (M) | 2 | 1317.70 | 3.58 | 127610 |
| Q9FIF7 | Putative pentatricopeptide repeat-containing protein At5g59200, chloroplastic | *Arabidopsis thaliana* | EAIYQRLQELNR | 2 Deamidation (NQ),O-2H (Y) | 2 | 1547.76 | 0.83 | 959950 |
| Q9FJC0 | Putative FBD-associated F-box protein At5g53640 | *Arabidopsis thaliana* | GLNQRLK | 2 Deamidation (NQ) | 2 | 829.47 | 0.30 | 511430 |
| Q9FJL0 | Structural maintenance of chromosomes protein 4, AtSMC4, SMC protein 4, SMC-4 | *Arabidopsis thaliana* | AKENNQIEGIYGR | Acetyl (K),2 Deamidation (NQ) | 2 | 1534.73 | 0.31 | 270200 |
| Q9FKQ2 | Putative clathrin assembly protein At5g65370 | *Arabidopsis thaliana* | FEELNVR | Deamidation (NQ) | 1 | 906.44 | 0.02 | 254020 |
| Q9FLK4 | Flavin-containing monooxygenase FMO GS-OX-like 8, EC 1.8 | *Arabidopsis thaliana* | RNTHDIADFNYSDK | Deamidation (NQ),Oxidation Y | 2 | 1711.74 | -2.69 | 91660 |
| Q9FLR5 | Structural maintenance of chromosomes protein 6A | *Arabidopsis thaliana* | ISSRKEELR | Acetyl (K) | 2 | 1158.64 | 0.87 | 179370 |
| Q9FMM3 | Protein ESSENTIAL FOR POTEXVIRUS ACCUMULATION 1 | *Arabidopsis thaliana* | SASSPSQAVSQSSSQSKSK | 3 Deamidation (NQ) | 2 | 1869.84 | -0.13 | 188750 |
| Q9FMN1 | Protein WEAK CHLOROPLAST MOVEMENT UNDER BLUE LIGHT-like 3, Protein WEL3 | *Arabidopsis thaliana* | LVEAKKEMEAAR | Acetyl (K),Oxidation (M) | 2 | 1431.74 | -1.49 | 1408100 |
| Q9FN92 | Probable receptor-like protein kinase At5g59700, EC 2.7.11.- | *Arabidopsis thaliana* | VTPSNDTLSR | Deamidation (NQ) | 2 | 1089.53 | -0.53 | 5176300 |
| Q9FX43 | Mitogen-activated protein kinase kinase 9, AtMKK9, MAP kinase kinase 9, EC 2.7.12.2 | *Arabidopsis thaliana* | QLMREMEILR | 2 Oxidation (M),Deamidation (NQ) | 2 | 1350.66 | -2.01 | 397230 |
| Q9FXA8 | Alkaline/neutral invertase A, mitochondrial, A/N-INVA, EC 3.2.1.26 | *Arabidopsis thaliana* | CSRVAAKTQILVR | Acetyl (K),Deamidation (NQ),Trioxidation (C) | 2 | 1591.84 | -2.26 | 95096 |
| Q9FZ42 | NADPH-dependent aldehyde reductase 1, chloroplastic, AtChlADR1, EC 1.1.1. | *Arabidopsis thaliana* | HMKEGSSIINTTSVNAYK | Oxidation (M),2 Deamidation (NQ) | 2 | 1996.94 | 3.66 | 179940 |
| Q9LE59 | Polygalacturonate 4-alpha-galacturonosyltransferase, EC 2.4.1.43 | *Arabidopsis thaliana* | LENAAIER | Deamidation (NQ) | 2 | 915.47 | -0.71 | 336210 |
| Q9LFA2 | Serine/threonine-protein kinase KIPK1, EC 2.7.11.1 (KCBP-interacting protein kinase) | *Arabidopsis thaliana* | IASMKKPGTPQSPR | 2 Acetyl (K),di-oxidation (M) | 2 | 1612.82 | 1.73 | 329750 |
| Q9LIH5 | Zinc finger CCCH domain-containing protein 38, AtC3H38 | *Arabidopsis thaliana* | SSDYNNTDYPEDNSR | 2 Deamidation (NQ),Oxidation Y | 2 | 1793.65 | -3.93 | 640340 |
| Q9LM46 | Agamous-like MADS-box protein AGL104 | *Arabidopsis thaliana* | LQQQLQMAEEELRR | 2 Deamidation (NQ) | 2 | 1772.87 | -3.95 | 1663000 |
| Q9LMI0 | Probable alpha,alpha-trehalose-phosphate synthase [UDP-forming] 7, EC 2.4.1.15 | *Arabidopsis thaliana* | NGSWSFSWDQDSLYLQLK | Trp->Kynurenine,Di-oxidation W | 2 | 2209.00 | -4.19 | 320750 |
| Q9LMT8 | Homeobox-leucine zipper protein HDG12 (HD-ZIP protein HDG12) | *Arabidopsis thaliana* | SLMTNIAVTAMEELLR | Oxidation (M),di-oxidation (M) | 2 | 1838.91 | -1.58 | 165470 |
| Q9LQ02 | DNA-directed RNA polymerase IV subunit 1 | *Arabidopsis thaliana* | QQLMVESWR | Oxidation (M),Deamidation (NQ),Trp->Kynurenine | 2 | 1196.55 | 0.06 | 311120 |
| Q9LS88 | Pentatricopeptide repeat-containing protein At3g23020 | *Arabidopsis thaliana* | TQYPDVYTSNCMINLYSER | Deamidation (NQ),O-2H (Y) | 2 | 2368.00 | 0.69 | 4526800 |
| Q9LUP5 | F-box/kelch-repeat protein At3g17530 | *Arabidopsis thaliana* | DMIVYSSGVSLKGNTYWVSGSKEK | Iodination | 3 | 2760.20 | -2.30 | 521370 |
| Q9LV52 | Heat stress transcription factor C-1, AtHsfC1 (AtHsf-08) | *Arabidopsis thaliana* | PEQMMAFLYK | Deamidation (NQ),O-2H (Y) | 2 | 1271.56 | 2.47 | 255600 |
| Q9LVW3 | Anthocyanidin 3-O-glucoside 2'''-O-xylosyltransferase, A3G2''XylT, EC 2.4.2.51 | *Arabidopsis thaliana* | KNHDKWR | Trp->Kynurenine | 2 | 986.50 | -2.98 | 198450 |
| Q9LXQ4 | Putative F-box/LRR-repeat protein At3g44080 | *Arabidopsis thaliana* | VTLDLHYLRYGYQK | Iodination,O-2H (Y) | 2 | 1907.81 | -4.20 | 802930 |
| Q9M096 | Putative F-box/LRR-repeat protein 19 | *Arabidopsis thaliana* | SCQNLKILK | 2 Deamidation (NQ),Trioxidation (C) | 2 | 1152.57 | -2.31 | 1692500 |
| Q9M1D8 | Pentatricopeptide repeat-containing protein At3g60050 | *Arabidopsis thaliana* | EACWLLKEMESR | Acetyl (K),Trioxidation (C) | 2 | 1640.72 | -0.78 | 315210 |
| Q9M1V5 | Heat stress transcription factor A-7b, AtHsfA7b (AtHsf-10) | *Arabidopsis thaliana* | AVENPSLLQQIFEQK | 3 Deamidation (NQ) | 2 | 1745.87 | -1.58 | 151750 |
| Q9M2E8 | Homeobox-leucine zipper protein HDG1 (HD-ZIP protein HDG1) | *Arabidopsis thaliana* | NGEIMESNVSRK | Oxidation (M),Deamidation (NQ) | 2 | 1379.64 | -2.65 | 280220 |
| Q9M5Q1 | Galactoside 2-alpha-L-fucosyltransferase, EC 2.4.1.- (PsFT1) (Xyloglucan alpha-(1,2)-fucosyltransferase) | *Pisum sativum* | PWILYKPENR | Trp->Kynurenine | 2 | 1318.70 | -3.87 | 235310 |
| Q9M891 | Pentatricopeptide repeat-containing protein At3g02490, mitochondrial | *Arabidopsis thaliana* | DVYKLFSELVK | Acetyl (K) | 3 | 1381.75 | 2.99 | 2186400 |
| Q9M8T5 | WEB family protein At3g02930, chloroplastic | *Arabidopsis thaliana* | ANELIASLENEKAK | 2 Deamidation (NQ) | 2 | 1530.78 | 1.89 | 3191200 |
| Q9M8Z5 | Guanine nucleotide-binding protein-like NSN1, Nucleolar GTP-binding protein NSN1 (DAR GTPase 4) | *Arabidopsis thaliana* | VEKDPGIPNDWPFKEQELK | Deamidation (NQ) | 3 | 2269.13 | -1.74 | 337540 |
| Q9MBF8 | Dynein-1-beta heavy chain, flagellar inner arm I1 complex (1-beta DHC) (Dynein-1, subspecies f) | *Chlamydomonas reinhardtii* | DIRQWTVWSSLK | Trp->Kynurenine,Di-oxidation W | 2 | 1553.78 | -4.00 | 369780 |
| Q9SAB3 | Polyadenylate-binding protein RBP45B, Poly(A)-binding protein RBP45B (RNA-binding protein 45B, AtRBP45B) | *Arabidopsis thaliana* | LSWGRSPSNK | Deamidation (NQ),Di-oxidation W | 2 | 1163.56 | 1.21 | 1729800 |
| Q9SAF0 | Oxysterol-binding protein-related protein 1D (OSBP-related protein 1D) | *Arabidopsis thaliana* | IKGGSNYSCRLK | Unmodified | 2 | 1381.71 | -3.29 | 1165400 |
| Q9SJQ1 | Leucine-rich repeat receptor-like protein kinase PXC1 | *Arabidopsis thaliana* | LLDLHDNRLNGTVSPLTNCK | 2 Deamidation (NQ) | 3 | 2281.14 | 0.35 | 1074300 |
| Q9SLH0 | ETHYLENE INSENSITIVE 3-like 1 protein | *Arabidopsis thaliana* | LAYGASK | Oxidation Y | 1 | 724.38 | 0.93 | 69637 |
| Q9SPL3 | Vicilin-like antimicrobial peptides 2-3 (MiAMP2) | *Macadamia integrifolia* | YGQAYEVKPEDYR | 2 Iodination,Di-iodination | 3 | 2120.33 | 2.86 | 2437700 |
| Q9SR82 | Putative pentatricopeptide repeat-containing protein At3g08820 | *Arabidopsis thaliana* | LICDMPMR | Oxidation (M),Trioxidation (C) | 2 | 1098.45 | 0.83 | 351530 |
| Q9STR4 | 1-aminocyclopropane-1-carboxylate synthase 7, ACC synthase 7, EC 4.4.1.14 | *Arabidopsis thaliana* | QAMASFMEQIRGGKAR | 2 Oxidation (M),Deamidation (NQ) | 3 | 1812.86 | 0.13 | 82046 |
| Q9STY4 | Riboflavin biosynthesis protein PYRR, chloroplastic | *Arabidopsis thaliana* | ISSSSPLICRATLSNGDNSR | Deamidation (NQ) | 3 | 2135.03 | 1.31 | 294270 |
| Q9SW96 | Asparagine--tRNA ligase, cytoplasmic 1, EC 6.1.1.22 (Asparaginyl-tRNA synthetase 1, AsnRS 1) | *Arabidopsis thaliana* | KDGNIDYSK | Deamidation (NQ),Oxidation Y | 2 | 1055.48 | 0.48 | 104470 |
| Q9SXA1 | Phosphatidylinositol 4-kinase alpha 1, PI4-kinase alpha 1, PtdIns-4-kinase alpha 1, EC 2.7.1.67 | *Arabidopsis thaliana* | QLLLMLCQHEADR | Oxidation (M),Deamidation (NQ) | 2 | 1642.78 | 0.05 | 521930 |
| Q9SY07 | Pentatricopeptide repeat-containing protein At4g02820, mitochondrial | *Arabidopsis thaliana* | AGEMALIVEERMAK | Oxidation (M) | 2 | 1562.78 | -0.96 | 2298200 |
| Q9SYI0 | Protein translocase subunit SECA1, chloroplastic, AtcpSecA, EC 7.4.2.4 | *Arabidopsis thaliana* | LFPCKLSNEKAK | Acetyl (K) | 2 | 1475.78 | -0.38 | 765860000 |
| Q9T0P4 | Ferredoxin-dependent glutamate synthase 2, chloroplastic, EC 1.4.7.1 (Fd-GOGAT 2) | *Arabidopsis thaliana* | WHLAQPMR | Oxidation (M),Deamidation (NQ),Trp->Kynurenine | 2 | 1058.50 | -3.52 | 3682700 |
| Q9TL30 | 50S ribosomal protein L12, chloroplastic | *Nephroselmis olivacea* | DMIESVPK | Oxidation (M) | 1 | 933.45 | -2.18 | 699170 |
| Q9XEA0 | Leucine--tRNA ligase, chloroplastic/mitochondrial, EC 6.1.1.4 | *Arabidopsis thaliana* | DSKTWNTSGIEGVHR | Deamidation (NQ),Trp->Kynurenine | 2 | 1690.79 | -0.69 | 677910 |
| Q9ZU29 | Pentatricopeptide repeat-containing protein At2g01390 | *Arabidopsis thaliana* | VEEATEVYKEMLRSR | Oxidation (M) | 2 | 1854.91 | -3.84 | 365700 |
| Q9ZWB7 | Glutathione gamma-glutamylcysteinyltransferase 2, EC 2.3.2.15 | *Arabidopsis thaliana* | RTEDVNQNLSSEEK | 2 Deamidation (NQ) | 2 | 1649.74 | 3.61 | 1638000 |
| A0A1P8ARG1 | Protein NO VEIN-LIKE | *Arabidopsis thaliana* | GEEIAYRYFVAK | Oxidation Y,O-2H (Y) | 2 | 1474.71 | -2.98 | 526410 |
| A0A1S3ZP85 | Triacylglycerol lipase OBL1 | *Nicotiana tabacum* | LLGIYTYGQPR | Deamidation (NQ),Oxidation Y,O-2H (Y) | 2 | 1310.65 | 0.53 | 280010 |
| A0A2H5AIZ1 | Hydroxycinnamoyltransferase | *Narcissus pseudonarcissus* | LTKQQLDLLK | 2 Deamidation (NQ) | 2 | 1200.70 | -2.81 | 100370 |
| A0A2I7G3B0 | Aldehyde dehydrogenase 1 | *Tanacetum cinerariifolium* | VLSYIELGK | dioxidation Y | 2 | 1052.58 | -2.47 | 459940 |
| A0A2R6S148 | Transcription factor MYB1 | *Actinidia chinensis var. chinensis* | PSPPSNNEILWWDNK | 2 Deamidation (NQ),2 O-2H (W) | 2 | 1825.78 | 0.40 | 4624800 |
| A0A2U1KZS6 | NADPH--cytochrome P450 reductase 2 | *Artemisia annua* | EYVQHKLTQK | Glu->pyro-Glu,O-2H (Y) | 2 | 1268.65 | 0.93 | 254340 |
| A0A2U1Q018 | Alcohol dehydrogenase 1 | *Artemisia annua* | TNLCHVYPPSFSGLMNDGTSR | 2 Deamidation (NQ),O-2H (Y) | 2 | 2368.01 | -3.87 | 3415900 |
| A0A393 | Protein TIC 214 | *Coffea arabica* | EQTYVDKNR | 2 Deamidation (NQ),Oxidation Y | 2 | 1169.52 | 0.92 | 884420 |
| A3BXL8 | ABC transporter G family member 53 | *Oryza sativa subsp. japonica* | ETVNFSAKCQGIGHR | Acetyl (K),Glu->pyro-Glu | 2 | 1726.82 | -3.20 | 240950 |
| A4GGF4 | Protein TIC 214 | *Phaseolus vulgaris* | NIWISSNYNTYK | Deamidation (NQ),Oxidation Y,O-2H (Y) | 2 | 1532.68 | 4.33 | 550510 |
| A4QKG5 | Protein TIC 214 | *Barbarea verna* | NNYDFFVPENILSPKR | 2 Deamidation (NQ) | 2 | 1953.95 | -0.92 | 337920 |
| A6MMQ1 | Protein Ycf2 | *Dioscorea elephantipes* | NETLEESFWSSNINR | Deamidation (NQ),O-2H (W) | 2 | 1839.79 | 1.08 | 149220 |
| A7Y3J6 | Protein Ycf2 | *Ipomoea purpurea* | NLIQIQYNR | 2 Deamidation (NQ),dioxidation Y | 2 | 1194.59 | 0.15 | 323840 |
| A8CDT3 | Lupeol synthase | *Bruguiera gymnorhiza* | AQVEEARENFWR | 2 Deamidation (NQ),Di-oxidation W | 2 | 1567.69 | 1.37 | 2253300 |
| A8IB25 | 40S ribosomal protein SA | *Chlamydomonas reinhardtii* | LLILTDPR | Unmodified | 2 | 939.58 | 0.32 | 192850 |
| A8IW99 | Mitochondrial cardiolipin hydrolase | *Chlamydomonas reinhardtii* | EVADLKAQVDQLK | 2 Deamidation (NQ) | 2 | 1457.76 | 0.45 | 864670 |
| A8JF70 | Outer dynein arm protein 1 | *Chlamydomonas reinhardtii* | DMADMIQQANGAFEAREK | 3 Deamidation (NQ) | 3 | 2026.86 | 2.35 | 61713 |
| A8MRP2 | AIG2-like protein D | *Arabidopsis thaliana* | QLHMEGFLKMTK | 2 Oxidation (M) | 3 | 1493.74 | -3.51 | 275480 |
| A8W3H9 | Plastid 30S ribosomal protein S2 | *Cuscuta obtusiflora* | MEQKKGGLNNLPK | 2 Deamidation (NQ) | 2 | 1457.75 | -2.11 | 1082900 |
| A9SVH7 | Retinoblastoma-related protein | *Physcomitrium patens* | MTLWINFQKTGGLESGDIIK | Deamidation (NQ),Di-oxidation W | 2 | 2283.15 | 4.09 | 215030 |
| B1A948 | Cytochrome f | *Carica papaya* | GRGQIYPDGRK | Deamidation (NQ),dioxidation Y | 2 | 1278.63 | 2.30 | 2448300 |
| B5UAQ8 | Cheilanthifoline synthase | *Eschscholzia californica* | SSSISTMEWPKGPK | Oxidation (W) | 2 | 1549.74 | -3.52 | 1035200 |
| B5X0N6 | K(+) efflux antiporter 6 | *Arabidopsis thaliana* | QRMILMSRQSHSS | Oxidation (M) | 2 | 1575.76 | -3.62 | 498090 |
| B7EJ91 | Kinesin-like protein KIN-5C | *Oryza sativa subsp. japonica* | DLLQDVDNMLQEARNSSSR | Oxidation (M),3 Deamidation (NQ) | 2 | 2208.98 | 2.87 | 293510 |
| B9FK36 | Acetyl-CoA carboxylase 2 | *Oryza sativa subsp. japonica* | RWGAMVIVK | O-2H (W) | 2 | 1072.59 | 0.45 | 8648400 |
| B9FUF9 | Kinesin-like protein KIN-12E | *Oryza sativa subsp. japonica* | LAQSDMLLSHAR | Oxidation (M),Deamidation (NQ) | 2 | 1357.67 | 2.15 | 606920 |
| D3UAG0 | UDP-glycosyltransferase 71K1 | *Malus domestica* | IIKWLDDQPQK | Deamidation (NQ),O-2H (W) | 2 | 1397.72 | -0.23 | 2069100 |
| F4I248 | CSC1-like protein At1g69450 | *Arabidopsis thaliana* | LANGTYKR | Deamidation (NQ),Oxidation Y | 2 | 938.48 | 1.61 | 334280 |
| F4I2H7 | Protein TPX2 | *Arabidopsis thaliana* | EKENQYKR | Deamidation (NQ) | 2 | 1094.54 | 3.42 | 70727 |
| F4IAT2 | THO complex subunit 2 | *Arabidopsis thaliana* | YHATSFGMMR | 2 Oxidation (M) | 3 | 1231.51 | -4.05 | 431910 |
| F4IVR7 | Myosin-10 | *Arabidopsis thaliana* | IQKQARTYICQTAFK | Deamidation (NQ) | 2 | 1855.96 | 1.58 | 634550 |
| F4J394 | Kinesin-like protein KIN-7G | *Arabidopsis thaliana* | EKDLQIEKLNK | 2 Deamidation (NQ) | 2 | 1358.73 | 0.24 | 2851500 |
| F4JMJ1 | Heat shock 70 kDa protein 17 | *Arabidopsis thaliana* | EIIKEWETNK | Unmodified | 2 | 1288.67 | -1.75 | 26998000 |
| F4JVN6 | Tripeptidyl-peptidase 2 | *Arabidopsis thaliana* | AEGIPVSPYSVRR | dioxidation Y | 2 | 1461.76 | 1.16 | 244290 |
| F4JW68 | FCS-Like Zinc finger 7 | *Arabidopsis thaliana* | EQQMEHDEGK | Oxidation (M),Deamidation (NQ) | 2 | 1246.48 | -1.93 | 4270400 |
| F4K0J3 | Kinesin-like protein KIN-4C | *Arabidopsis thaliana* | ARNIQNKAVINR | 3 Deamidation (NQ) | 2 | 1398.76 | 2.21 | 373180 |
| F4K3G5 | Protein ENHANCED DOWNY MILDEW 2 | *Arabidopsis thaliana* | EPGSEIPTLDNDSQR | Glu->pyro-Glu,2 Deamidation (NQ) | 2 | 1640.72 | 0.15 | 235510 |
| F4KEM0 | Protein STICHEL-like 4 | *Arabidopsis thaliana* | LTWLTAALLQLAPDK | Di-oxidation W | 2 | 1684.94 | -1.20 | 1485600 |
| F6M8I0 | Monoterpene synthase | *Santalum album* | EENVNPNLLK | Glu->pyro-Glu,2 Deamidation (NQ) | 2 | 1152.57 | 0.62 | 1622000 |
| G7J1L1 | GRAS family protein TF80 | *Medicago truncatula* | IEATNLLQR | Deamidation (NQ) | 2 | 1057.58 | -0.87 | 278270 |
| O22892 | Ribosome biogenesis protein NOP53 | *Arabidopsis thaliana* | ENPFVQLKPSSNTNLKK | 3 Deamidation (NQ) | 2 | 1946.00 | -4.49 | 281990 |
| O23181 | Patatin-like protein 3 | *Arabidopsis thaliana* | NPVMGDISPLDFTR | Deamidation (NQ),di-oxidation (M) | 2 | 1593.73 | -1.13 | 948140 |
| O48573 | Disease resistance protein LAZ5 | *Arabidopsis thaliana* | LPQEMENMKSLVFLNMRR | 2 Oxidation (M) | 3 | 2267.12 | -0.94 | 901070 |
| O50003 | 60S ribosomal protein L12 | *Prunus armeniaca* | VTGGEVGAASSLAPK | Unmodified | 2 | 1342.71 | 1.68 | 189890 |
| O65352 | 14-3-3-like protein | *Helianthus annuus* | EESRGNEGHVSTIRDYR | Deamidation (NQ),Oxidation Y | 3 | 2020.92 | 0.18 | 114840 |
| O65569 | 40S ribosomal protein S11-2 | *Arabidopsis thaliana* | DYLHFVKKYR | Oxidation Y | 2 | 1383.73 | -0.48 | 790540 |
| O65790 | Cytochrome P450 81F1 | *Arabidopsis thaliana* | GEEMDMSESTGLGMRK | 2 Oxidation (M) | 2 | 1788.73 | 0.93 | 4820500 |
| O80492 | Probable protein phosphatase 2C 5 | *Arabidopsis thaliana* | NHMDTNNK | Oxidation (M),3 Deamidation (NQ) | 2 | 991.36 | -3.51 | 534290 |
| O80689 | Beta-glucosidase 45 | *Arabidopsis thaliana* | FQSWLNPEMQK | Oxidation (M),Deamidation (NQ),Trp->Kynurenine | 2 | 1427.64 | 3.37 | 2088900 |
| O98634 | Maturase K | *Brasenia schreberi* | SQMVENSFMIDTAIKR | Oxidation (M),2 Deamidation (NQ) | 2 | 1886.88 | 2.26 | 377190 |
| P07374 | Urease | *Canavalia ensiformis* | VNVLYGLNKR | 2 Deamidation (NQ) | 2 | 1176.65 | 0.30 | 238230 |
| P0CB23 | Uncharacterized protein At4g38062 | *Arabidopsis thaliana* | AVNEKLR | Deamidation (NQ) | 2 | 829.47 | 0.23 | 675330 |
| P0DI20 | CASP-like protein 2U2 | *Osmunda lancea* | PMTAKTAQDI | Oxidation (M) | 2 | 1090.53 | 0.21 | 79551 |
| P0DN94 | S-protein homolog 17 | *Arabidopsis thaliana* | EDGIYLKKEGNK | Deamidation (NQ),O-2H (Y) | 2 | 1407.69 | 0.65 | 197440 |
| P0DO14 | Sarpagan bridge enzyme | *Gelsemium sempervirens* | PKTMVATIMLYNSSGVTFAPYGDYWK | Deamidation (NQ),Trp->Kynurenine | 3 | 2944.40 | -0.46 | 313540 |
| P15804 | Phosphoenolpyruvate carboxylase 3 | *Sorghum bicolor* | MEWLVSELK | Oxidation (M) | 2 | 1149.57 | -3.92 | 1384100 |
| P17569 | Nitrate reductase [NADH] | *Cucurbita maxima* | WADWTVEVCGLVKR | Unmodified | 3 | 1717.86 | -0.03 | 2562000 |
| P17571 | Nitrate reductase [NADH] | *Zea mays* | PYTLKGYAYSGGGK | dioxidation Y | 2 | 1492.72 | 4.29 | 194170 |
| P30567 | Catalase isozyme 2 | *Gossypium hirsutum* | EKCIIGKENNFK | 2 Acetyl (K) | 2 | 1562.78 | -0.51 | 1678700 |
| P31683 | Enolase | *Chlamydomonas reinhardtii* | EAGWGVMTSHR | Di-oxidation W | 2 | 1261.55 | -0.87 | 119070 |
| P36910 | Acidic endochitinase SE2 | *Beta vulgaris* | GSAKYGGVMLWSK | O-2H (Y) | 2 | 1396.68 | 1.57 | 695930 |
| P38662 | Lectin | *Lablab purpureus* | VTASWDWQNGK | 2 Deamidation (NQ),Oxidation (W) | 2 | 1308.56 | -1.86 | 496140 |
| P40621 | HMG1/2-like protein | *Triticum aestivum* | LKGEYNKAIAAYNK | Deamidation (NQ),Oxidation Y | 2 | 1598.83 | -2.30 | 74833 |
| P41653 | Protein Ycf2 | *Pinus thunbergii* | YLAYTYQK | 2 dioxidation Y | 2 | 1112.50 | -1.84 | 706010 |
| P49100 | Cytochrome b5 | *Oryza sativa subsp. japonica* | DDCWLIIGGKVYNVSK | Oxidation (W) | 3 | 1881.93 | -0.32 | 273950 |
| P49102 | Nitrate reductase [NADH] 3 | *Zea mays* | IYTMKGFAYSGGGK | Oxidation Y | 3 | 1494.72 | -3.06 | 174740 |
| P54968 | IAA-amino acid hydrolase ILR1 | *Arabidopsis thaliana* | EISEAQASVYRCK | Glu->pyro-Glu,O-2H (Y) | 2 | 1535.70 | 3.77 | 748880 |
| P55005 | Beta-amylase | *Zea mays* | YDATAYNTILRNARPQGINK | 3 Deamidation (NQ) | 3 | 2281.13 | 0.94 | 299510 |
| P58051 | Cytochrome P450 71B14 | *Arabidopsis thaliana* | AQAEVREVIKNK | Acetyl (K),Deamidation (NQ) | 2 | 1426.78 | 0.33 | 7680800 |
| P60040 | 60S ribosomal protein L7-2 | *Arabidopsis thaliana* | ELIYKRGYGK | 2 dioxidation Y | 2 | 1289.66 | 2.19 | 340100 |
| P61241 | Protein Ycf2 | *Amborella trichopoda* | NLCLNKCVEK | Deamidation (NQ),Trioxidation (C) | 2 | 1325.60 | -0.73 | 233450 |
| P69310 | Ubiquitin | *Avena sativa* | TLADYNIQK | Oxidation Y | 2 | 1080.55 | 0.12 | 1917100 |
| P82621 | Defensin-like protein 226 | *Arabidopsis thaliana* | EVESKTKWGCDMNR | Acetyl (K),Oxidation (M) | 2 | 1796.78 | -1.00 | 279940 |
| P93119 | DNA topoisomerase 1 | *Daucus carota* | TLKEEGQSR | Acetyl (K),Deamidation (NQ) | 2 | 1089.53 | -0.12 | 3895600 |
| P93354 | Histone H2B | *Nicotiana tabacum* | AEMSVETYK | dioxidation Y | 1 | 1088.47 | -0.46 | 137550 |
| P93703 | Cytochrome P450 71C3 | *Zea mays* | VLVNGWAIGR | Trp->Kynurenine | 2 | 1087.61 | -3.16 | 1204500 |
| Q00016 | Isoflavone reductase | *Cicer arietinum* | GAYVTEADVGTYTIR | 2 dioxidation Y | 2 | 1678.77 | -1.22 | 333660 |
| Q01781 | Adenosylhomocysteinase | *Petroselinum crispum* | LVGVSEETTTGVKR | Unmodified | 3 | 1474.80 | 1.52 | 164740 |
| Q01861 | Phenylalanine ammonia-lyase 1 | *Pisum sativum* | VISPGEECDKLFTAICQGK | Acetyl (K),Deamidation (NQ),Trioxidation (C) | 2 | 2242.01 | 2.91 | 196340 |
| Q06FP6 | Protein TIC 214 | *Pelargonium hortorum* | VAIDSWVTGQNR | 2 Deamidation (NQ),Trp->Kynurenine | 2 | 1350.64 | 0.37 | 1786600 |
| Q0DZ85 | Expansin-B16 | *Oryza sativa subsp. japonica* | LAVAGHGGQLQNR | 3 Deamidation (NQ) | 2 | 1322.66 | 4.44 | 127740 |
| Q0IQU1 | Laccase-22 | *Oryza sativa subsp. japonica* | LSVENGKTYMLR | Oxidation (M),O-2H (Y) | 2 | 1439.71 | -0.64 | 2326500 |
| Q0JCR9 | Sugar transport protein MST1 | *Oryza sativa subsp. japonica* | HWYWKRFAR | Trp->Kynurenine | 2 | 1352.69 | 2.91 | 703740 |
| Q0WN69 | Kinesin-like protein KIN-14P | *Arabidopsis thaliana* | IQELEQNLVMWK | Oxidation (M),2 Deamidation (NQ) | 2 | 1547.75 | 3.74 | 789250 |
| Q0ZIW0 | Protein TIC 214 | *Vitis vinifera* | TGSNNYQIIDK | Deamidation (NQ),Oxidation Y | 2 | 1268.59 | 2.72 | 134770 |
| Q0ZPV7 | Carboxylesterase 1 | *Actinidia eriantha* | LEDPEKAK | Acetyl (K) | 2 | 970.50 | 0.16 | 2346500 |
| Q14FA0 | Protein TIC 214 | *Populus alba* | NGNDINTEIINNNNIK | 2 Deamidation (NQ) | 3 | 1800.85 | 1.27 | 397290 |
| Q15KI9 | Protein PHYLLO, chloroplastic | *Arabidopsis thaliana* | VSSVQDALVMQEVRR | 2 Deamidation (NQ) | 3 | 1717.87 | -2.53 | 2864600 |
| Q1PFK0 | F-box/LRR-repeat protein At1g55660 | *Arabidopsis thaliana* | WKSLWMWLPK | Trp->Kynurenine,Di-oxidation W | 2 | 1409.72 | 4.32 | 163430 |
| Q27GK7 | Topless-related protein 4 | *Arabidopsis thaliana* | SKTWKLTEISER | O-2H (W) | 2 | 1490.77 | -1.14 | 412030 |
| Q2MJ21 | Cytochrome P450 716A67 | *Medicago truncatula* | VLNWIWLK | Deamidation (NQ),Oxidation (W) | 2 | 1087.61 | 3.47 | 1088600 |
| Q2WGD3 | Protein TIC 214 | *Selaginella uncinata* | FRGILDFIAYYEK | 2 dioxidation Y | 2 | 1697.83 | 2.42 | 226710 |
| Q38846 | Ethylene response sensor 1 | *Arabidopsis thaliana* | EMGLILTQEETGR | Oxidation (M),Glu->pyro-Glu,Deamidation (NQ) | 2 | 1474.70 | 4.48 | 526410 |
| Q38946 | Glutamate dehydrogenase 2 | *Arabidopsis thaliana* | ENAGDVKAK | Glu->pyro-Glu,Deamidation (NQ) | 2 | 913.45 | 3.53 | 704290 |
| Q3B724 | Callose synthase 5 | *Arabidopsis thaliana* | TVGMWGSVK | Oxidation (M),O-2H (W) | 2 | 993.46 | -2.64 | 237960 |
| Q42485 | Zinc finger protein 1 | *Arabidopsis thaliana* | MEPSIKGDQEMLK | Oxidation (M) | 2 | 1520.72 | 0.88 | 1276100 |
| Q52MZ2 | bZIP transcription factor TGA10 | *Nicotiana tabacum* | ALSSLWHARPR | O-2H (W) | 3 | 1306.69 | -1.78 | 121260 |
| Q5MGA9 | Nucleosome assembly protein 1;3 | *Oryza sativa subsp. indica* | YHKLYGPLYSKR | 2 O-2H (Y) | 2 | 1551.78 | 3.95 | 1745700 |
| Q5PP28 | NAC domain-containing protein 3 | *Arabidopsis thaliana* | SGFWKSTGRPK | Trp->Kynurenine | 2 | 1253.65 | -0.06 | 3175400 |
| Q5SBP7 | Selinene synthase | *Ocimum basilicum* | MSANCVSAAPTSPKNSDVEEIRK | Oxidation (M),Deamidation (NQ) | 3 | 2507.16 | -3.26 | 496330 |
| Q688U3 | Zinc-finger homeodomain protein 6 | *Oryza sativa subsp. japonica* | VWMHNNKSSIGSSSGGGSR | Acetyl (K),Deamidation (NQ),di-oxidation (M) | 2 | 2021.89 | -1.17 | 7295700 |
| Q68RU8 | Protein TIC 214 | *Panax ginseng* | VSCLLIGLK | Trioxidation (C) | 2 | 1049.58 | -1.96 | 2099300 |
| Q69Q02 | 4-alpha-glucanotransferase DPE2 | *Oryza sativa subsp. japonica* | QFLSENEEWLK | 2 Deamidation (NQ),Trp->Kynurenine | 2 | 1427.65 | -0.87 | 1881700 |
| Q6IMT1 | Protein SABRE | *Arabidopsis thaliana* | VSLDWGR | Di-oxidation W | 2 | 863.41 | -1.30 | 111190 |
| Q6K2M1 | BURP domain-containing protein 14 | *Oryza sativa subsp. japonica* | MEVSKIEAAAR | di-oxidation (M) | 2 | 1235.62 | -1.79 | 3040500 |
| Q6NQJ8 | Protein SET DOMAIN GROUP 40 | *Arabidopsis thaliana* | LWLIPQSQRDKSVMR | Deamidation (NQ),Oxidation (W) | 2 | 1872.99 | 1.93 | 95469 |
| Q6WNQ9 | Isoflavone 3'-hydroxylase | *Medicago truncatula* | HWNDALSFK | Trp->Kynurenine | 2 | 1120.53 | -2.47 | 2320100 |
| Q6YXP5 | Protein TIC 214 | *Physcomitrium patens* | ENIKINNNLK | Deamidation (NQ) | 3 | 1199.65 | 0.37 | 374550 |
| Q6ZJK7 | Tryptophan decarboxylase 1 | *Oryza sativa subsp. japonica* | ELMERLNKTGK | Deamidation (NQ) | 2 | 1318.69 | -2.19 | 77318 |
| Q75GE8 | Calcium-dependent protein kinase 8 | *Oryza sativa subsp. japonica* | AGTDWRKASR | O-2H (W) | 2 | 1160.57 | 2.44 | 651350 |
| Q7X7E9 | Putative DNA ligase 4 | *Oryza sativa subsp. japonica* | EKRLLHLQPK | Glu->pyro-Glu,Deamidation (NQ) | 2 | 1243.74 | 2.07 | 2641000 |
| Q7X9B9 | Protein NLP2 | *Arabidopsis thaliana* | ANEKGKGVSLSWEYQK | Acetyl (K),2 Deamidation (NQ) | 3 | 1866.90 | 3.04 | 2086400 |
| Q7XA73 | Protein TIFY 4A | *Arabidopsis thaliana* | DSGMEGQANRKVSLQR | 2 Deamidation (NQ) | 2 | 1776.84 | -0.49 | 409720 |
| Q7XH05 | Probable aldehyde oxidase 1 | *Oryza sativa subsp. japonica* | LASTPEYQR | Deamidation (NQ) | 2 | 1064.51 | 0.98 | 259890000 |
| Q84JL3 | E3 ubiquitin-protein ligase SINAT3 | *Arabidopsis thaliana* | NYNYSLEVGGYGRK | Deamidation (NQ),2 Di-iodination | 4 | 2123.34 | 3.20 | 165060 |
| Q84JS6 | Homeobox protein knotted-1-like 6 | *Arabidopsis thaliana* | EARQALLDWWNLHYK | Glu->pyro-Glu,Deamidation (NQ),Oxidation (W),Trp->Kynurenine | 2 | 1944.95 | -0.54 | 1682200 |
| Q84SL0 | Calcium-dependent protein kinase 20 | *Oryza sativa subsp. japonica* | DLVKGMLNPDPR | Acetyl (K),Deamidation (NQ) | 2 | 1396.70 | 1.86 | 1022500 |
| Q84WJ0 | Protein DA1-related 5 | *Arabidopsis thaliana* | NQLQYMR | Oxidation (M),2 Deamidation (NQ) | 2 | 969.42 | 0.52 | 959950 |
| Q8GRN0 | FCS-Like Zinc finger 13 | *Arabidopsis thaliana* | SVQIMNDERQEQCK | Oxidation (M),Deamidation (NQ),Trioxidation (C) | 2 | 1828.76 | -0.18 | 492440 |
| Q8H181 | Trihelix transcription factor GTL2 | *Arabidopsis thaliana* | TLKPKNQNPK | 2 Deamidation (NQ) | 2 | 1168.65 | -0.84 | 1578600 |
| Q8H1B3 | Heat shock 70 kDa protein BIP3 | *Arabidopsis thaliana* | QATKDAGAIAGLNVVR | Acetyl (K),Deamidation (NQ) | 2 | 1625.87 | 0.14 | 926360 |
| Q8L5Z1 | GDSL esterase/lipase At1g33811 | *Arabidopsis thaliana* | TYVDALAQILGFR | Oxidation Y | 3 | 1481.79 | 3.03 | 197130 |
| Q8L7L1 | Sphingosine kinase 1 | *Arabidopsis thaliana* | LALLSLMTK | di-oxidation (M) | 1 | 1020.59 | 0.45 | 300420 |
| Q8L805 | 60S ribosomal protein L35 | *Triticum aestivum* | SKDDLTKQLAELK | Acetyl (K),Deamidation (NQ) | 2 | 1530.81 | -0.67 | 35098000 |
| Q8LBL5 | E3 ubiquitin-protein ligase PRT1 | *Arabidopsis thaliana* | QISKDDLLCSACKELLVR | 2 Trioxidation (C) | 2 | 2243.08 | 4.09 | 99655 |
| Q8LJW1 | ACT domain-containing protein ACR7 | *Arabidopsis thaliana* | KAYISSDGK | Oxidation Y | 2 | 983.49 | -0.03 | 75411 |
| Q8LPL3 | L-ascorbate oxidase | *Arabidopsis thaliana* | KLIMDNYDIMK | Oxidation (M),Deamidation (NQ) | 2 | 1399.67 | 0.73 | 1058600 |
| Q8RWU5 | F-box/LRR-repeat protein 3 | *Arabidopsis thaliana* | GLSYIGMGCSNLR | Deamidation (NQ),Trioxidation (C),di-oxidation (M) | 2 | 1507.63 | 0.93 | 1153600 |
| Q8RXF8 | Mitochondrial Rho GTPase 1 | *Arabidopsis thaliana* | LGDFNNLFR | Deamidation (NQ) | 2 | 1095.53 | -4.14 | 249580 |
| Q8RXT9 | GDSL esterase/lipase At1g28590 | *Arabidopsis thaliana* | QLQEELNGLRK | Deamidation (NQ) | 2 | 1327.71 | -0.13 | 1934200 |
| Q8S2T0 | Protein GRIP | *Arabidopsis thaliana* | QAWEEDLR | Deamidation (NQ),Oxidation (W) | 2 | 1062.46 | -0.67 | 99545 |
| Q8S3Q9 | Zinc-finger homeodomain protein 7 | *Oryza sativa subsp. japonica* | VWMHNNKHLAK | Oxidation (M),2 Deamidation (NQ) | 2 | 1394.68 | -3.48 | 178570 |
| Q8S5N2 | Inositol-3-phosphate synthase 1 | *Oryza sativa subsp. japonica* | RAMDEYTSEIFMGGK | 2 Oxidation (M) | 2 | 1765.77 | 2.59 | 84494 |
| Q8VXQ2 | Aldehyde dehydrogenase | *Craterostigma plantagineum* | IEECYKIIASK | Acetyl (K) | 3 | 1394.71 | 0.75 | 5024400 |
| Q8WJ18 | Maturase K | *Syzygium australe* | SQMLENSFLINNAMKK | Deamidation (NQ) | 3 | 1867.92 | -1.44 | 338060 |
| Q93V56 | Soluble inorganic pyrophosphatase 1 | *Arabidopsis thaliana* | SEETKDNQRLQR | 2 Deamidation (NQ) | 2 | 1504.71 | 4.12 | 1655600 |
| Q93YV0 | (E,E)-geranyllinalool synthase | *Arabidopsis thaliana* | MNSVLLHMK | Oxidation (M),Deamidation (NQ) | 2 | 1088.54 | -3.21 | 139340 |
| Q93Z37 | Protein BIG GRAIN 1-like E | *Arabidopsis thaliana* | LKVMESLSENQR | Acetyl (K),Oxidation (M) | 2 | 1490.74 | -1.95 | 4596200 |
| Q93ZD7 | Magnesium transporter MRS2-4 | *Arabidopsis thaliana* | LKQQFPQR | 3 Deamidation (NQ) | 2 | 1046.54 | -3.50 | 61163 |
| Q93ZT5 | EID1-like F-box protein 3 | *Arabidopsis thaliana* | TSGRFFLPKNCR | Acetyl (K),Trioxidation (C) | 2 | 1571.75 | 1.98 | 472090 |
| Q940Q4 | RING-H2 finger protein ATL13 | *Arabidopsis thaliana* | RTMLWLAGRQNK | Oxidation (M),O-2H (W) | 2 | 1502.78 | -2.97 | 965320 |
| Q94A41 | Alpha-amylase 3, chloroplastic | *Arabidopsis thaliana* | DLYNLNSRYGTIDELK | dioxidation Y | 2 | 1944.94 | 2.00 | 1682200 |
| Q95AG0 | Cytochrome f | *Chlamydomonas subcaudata* | MIFKPQSFLK | Acetyl (K),Oxidation (M) | 2 | 1295.69 | 2.18 | 3064400 |
| Q96453 | 14-3-3-like protein D | *Glycine max* | VELELSNICNDVMR | Trioxidation (C),di-oxidation (M) | 2 | 1770.78 | 0.81 | 376720 |
| Q99285 | Histone H2B | *Pisum sativum* | QVHPDIGISSK | Gln->pyro-Glu | 2 | 1162.60 | -0.01 | 581200 |
| Q9ATN1 | Aquaporin NIP3-1 | *Zea mays* | DENGETPRTQR | Deamidation (NQ) | 3 | 1302.58 | -1.83 | 231980 |
| Q9C927 | Purple acid phosphatase 5 | *Arabidopsis thaliana* | VSNIQYNITDGMSTPVK | Deamidation (NQ) | 3 | 1866.90 | -0.14 | 2086400 |
| Q9C9D0 | Cytosolic sulfotransferase 16 | *Arabidopsis thaliana* | EDRPAVYANSAYFR | Glu->pyro-Glu,O-2H (Y) | 2 | 1653.75 | 2.69 | 207740 |
| Q9FE01 | L-ascorbate peroxidase 2, cytosolic | *Oryza sativa subsp. japonica* | QVFSAQMGLSDK | Deamidation (NQ) | 2 | 1310.62 | 2.06 | 4439200 |
| Q9FFP2 | Protein KOKOPELLI | *Arabidopsis thaliana* | NVMGRFKR | Acetyl (K),Deamidation (NQ),di-oxidation (M) | 2 | 1081.53 | 1.54 | 713400 |
| Q9FH99 | F-box protein At5g67140 | *Arabidopsis thaliana* | CHQLKTIGMWCCR | Deamidation (NQ),O-2H (W) | 2 | 1763.74 | 3.26 | 237150 |
| Q9FIB0 | Cytochrome P450 78A7 | *Arabidopsis thaliana* | YDPLAQK | dioxidation Y | 1 | 865.42 | 0.34 | 1153000 |
| Q9FIM4 | Transcription factor MYB119 | *Arabidopsis thaliana* | ENTTFGPTREKHLVLNGGNR | Deamidation (NQ) | 3 | 2240.13 | -3.47 | 177600 |
| Q9FIN7 | MAG2-interacting protein 2 | *Arabidopsis thaliana* | WSEYWNPK | Deamidation (NQ),Trp->Kynurenine | 2 | 1113.48 | 4.48 | 160760 |
| Q9FJ25 | GDSL esterase/lipase At5g41890 | *Arabidopsis thaliana* | VIGENGTKEMLK | Acetyl (K),Oxidation (M) | 2 | 1375.70 | -2.40 | 447090 |
| Q9FKE6 | Cyclin-T1-5 | *Arabidopsis thaliana* | GEVKNTEEGEMVNNNVSPMMHSR | Oxidation (M),Deamidation (NQ) | 3 | 2605.12 | 0.06 | 467500 |
| Q9FKN7 | Protein DA1-related 4 | *Arabidopsis thaliana* | TPQYSKL | Acetyl (K) | 2 | 877.45 | -2.70 | 6156600 |
| Q9FKQ1 | Protein DETOXIFICATION 27 | *Arabidopsis thaliana* | ASARINKWSNTIK | Acetyl (K),2 Deamidation (NQ) | 2 | 1531.80 | -1.04 | 7373200 |
| Q9FKS8 | Lysine histidine transporter 1 | *Arabidopsis thaliana* | QKEIEDWLPITSSR | Deamidation (NQ),Di-oxidation W | 2 | 1733.85 | -4.15 | 657870 |
| Q9FMB6 | Transcription factor BIM3 | *Arabidopsis thaliana* | VLNTLTEALK | Deamidation (NQ) | 2 | 1101.63 | 1.39 | 976540 |
| Q9FN69 | Transcription factor GLABRA 3 | *Arabidopsis thaliana* | EKLMLDSPEAR | Acetyl (K),Oxidation (M) | 2 | 1345.65 | 0.58 | 742690 |
| Q9FT73 | ATP-dependent DNA helicase Q-like 2 | *Arabidopsis thaliana* | FNVFGISKYR | Iodination | 2 | 1355.55 | -1.86 | 335810 |
| Q9FT92 | Uncharacterized protein At5g08430 | *Arabidopsis thaliana* | LIDRANEK | Deamidation (NQ) | 2 | 958.51 | 1.32 | 522330 |
| Q9FWW5 | WEB family protein At1g12150 | *Arabidopsis thaliana* | RMVESELQRWR | Oxidation (M) | 2 | 1504.76 | -2.64 | 3275300 |
| Q9FY48 | E3 ubiquitin-protein ligase KEG | *Arabidopsis thaliana* | SLLEAQNADGQSALHLACRR | Deamidation (NQ) | 2 | 2210.09 | 0.16 | 333860 |
| Q9GI85 | Maturase K | *Adesmia lanata* | QFISSLEDAETIKSFNNLR | Deamidation (NQ) | 3 | 2212.10 | 1.82 | 1124600 |
| Q9LEJ0 | Enolase 1 | *Hevea brasiliensis* | VNQIGSVTESIEAVK | Unmodified | 2 | 1572.84 | 0.96 | 336820 |
| Q9LFJ9 | UDP-glycosyltransferase 78D4 | *Arabidopsis thaliana* | VSWVAFWTSGTR | O-2H (W),Di-oxidation W | 2 | 1441.66 | 0.35 | 1350400 |
| Q9LHN9 | IQ domain-containing protein IQM2 | *Arabidopsis thaliana* | ETKSFQLGKQLSCK | Acetyl (K),Deamidation (NQ),Trioxidation (C) | 2 | 1743.83 | 2.78 | 268710 |
| Q9LIF8 | Jacalin-related lectin 36 | *Arabidopsis thaliana* | FVYDKDSK | dioxidation Y | 1 | 1032.48 | -4.10 | 2388000 |
| Q9LKR6 | Jacalin-related lectin 40 | *Arabidopsis thaliana* | ILVRGGREGIQYVK | Iodination | 2 | 1712.82 | -0.92 | 181030 |
| Q9LM64 | Putative F-box protein At1g21990 | *Arabidopsis thaliana* | GECCLLTCQVK | Deamidation (NQ),2 Trioxidation (C) | 2 | 1463.56 | -1.56 | 1615700 |
| Q9LQQ9 | Mitogen-activated protein kinase 13 | *Arabidopsis thaliana* | LLSHMDHDNVIK | Oxidation (M),Deamidation (NQ) | 3 | 1437.69 | -0.30 | 285270 |
| Q9LQV2 | RNA-dependent RNA polymerase 1 | *Arabidopsis thaliana* | LLNQKKYDR | Deamidation (NQ),Oxidation Y | 2 | 1193.64 | 3.82 | 194860 |
| Q9LTY6 | Uridine kinase-like protein 5 | *Arabidopsis thaliana* | MEKLSNGVR | Deamidation (NQ),di-oxidation (M) | 2 | 1065.51 | -2.28 | 258530 |
| Q9LX56 | F-box protein At3g59150 | *Arabidopsis thaliana* | VLEISGYKGTWQELNQMKR | 2 Deamidation (NQ) | 3 | 2281.14 | -0.66 | 923950 |
| Q9M039 | Pyruvate decarboxylase 3 | *Arabidopsis thaliana* | VSAANGRPPNPQ | Deamidation (NQ) | 2 | 1207.59 | 1.01 | 127320 |
| Q9M8J3 | Protein FAR1-RELATED SEQUENCE 7 | *Arabidopsis thaliana* | YEQGLEQRR | Deamidation (NQ) | 2 | 1178.57 | 0.11 | 191850 |
| Q9MAG3 | ABC transporter G family member 24 | *Arabidopsis thaliana* | NLTFSGIVKMATNSETR | Oxidation (M) | 2 | 1883.94 | 1.37 | 115150 |
| Q9SB48 | NADPH--cytochrome P450 reductase 1 | *Arabidopsis thaliana* | LQQLAYGVFALGNR | 3 Deamidation (NQ) | 2 | 1551.79 | -1.88 | 1361200 |
| Q9SCV9 | Beta-galactosidase 3 | *Arabidopsis thaliana* | KALLINGQR | Acetyl (K),Deamidation (NQ) | 2 | 1054.61 | 0.27 | 5145000 |
| Q9SE83 | Dynamin-2A | *Arabidopsis thaliana* | ERYQKQSSLLSK | Acetyl (K),Glu->pyro-Glu,Deamidation (NQ) | 2 | 1490.77 | 0.27 | 548260 |
| Q9SGA8 | UDP-glycosyltransferase 83A1 | *Arabidopsis thaliana* | AMKVKEIVMK | Oxidation (M) | 2 | 1191.67 | 2.19 | 141030 |
| Q9SJB3 | ATPase 5, plasma membrane-type | *Arabidopsis thaliana* | LGMGTNMYPSSALLGQVK | Oxidation (M),Deamidation (NQ) | 3 | 1882.92 | -3.18 | 493150 |
| Q9SJG8 | Protein MEI2-like 2 | *Arabidopsis thaliana* | NIPNKYTYK | Deamidation (NQ),Oxidation Y,O-2H (Y) | 2 | 1170.56 | 3.17 | 225430 |
| Q9SJP9 | Proline transporter 3 | *Arabidopsis thaliana* | NNKLNTLQK | Acetyl (K),Deamidation (NQ) | 2 | 1114.60 | -0.13 | 3855800 |
| Q9SK32 | Protein MAIN-LIKE 1 | *Arabidopsis thaliana* | ISITKLYR | Iodination | 2 | 1118.50 | 3.24 | 205260 |
| Q9SP32 | Endoribonuclease Dicer homolog 1 | *Arabidopsis thaliana* | ITKTTAWDNPLQRAR | O-2H (W) | 2 | 1783.93 | 4.09 | 1306700 |
| Q9SR40 | Laccase-7 | *Arabidopsis thaliana* | IINAAMNIQLFFK | 3 Deamidation (NQ) | 2 | 1524.79 | 0.66 | 67095 |
| Q9SS38 | DNA gyrase subunit B, chloroplastic | *Arabidopsis thaliana* | SLNAYKAALAAK | O-2H (Y) | 2 | 1233.67 | -0.18 | 286940 |
| Q9SU04 | F-box protein KIB2 | *Arabidopsis thaliana* | ENKLYVLSVTR | Acetyl (K),Glu->pyro-Glu,Deamidation (NQ) | 2 | 1345.72 | 1.59 | 214340 |
| Q9SUN6 | Subtilisin-like protease SBT2.2 | *Arabidopsis thaliana* | TATTYTPQFMGLPK | Oxidation (M),Deamidation (NQ) | 2 | 1571.75 | -2.39 | 448060 |
| Q9SV30 | GATA transcription factor 8 | *Arabidopsis thaliana* | CMHCEVTKTPQWR | Oxidation (M),Deamidation (NQ),Trioxidation (C) | 2 | 1796.73 | 3.55 | 108500 |
| Q9SX99 | Protein OSB1, mitochondrial | *Arabidopsis thaliana* | RLELFDQKNR | Deamidation (NQ) | 2 | 1318.70 | -0.63 | 235310 |
| Q9SYD0 | Putative F-box only protein 10 | *Arabidopsis thaliana* | IWLTKNSINNR | Acetyl (K),2 Deamidation (NQ) | 2 | 1401.73 | -0.93 | 598220 |
| Q9T0N8 | Cytokinin dehydrogenase 1 | *Zea mays* | PWPASLAALALDGK | Di-oxidation W | 2 | 1440.76 | -1.28 | 120210 |
| Q9XIE4 | TORTIFOLIA1-like protein 5 | *Arabidopsis thaliana* | NLNTPLSSK | 2 Deamidation (NQ) | 2 | 974.49 | 0.21 | 40013000 |
| Q9ZPP1 | Calreticulin | *Berberis stolonifera* | WNGDANDK | 2 Deamidation (NQ),O-2H (W) | 2 | 934.33 | -2.01 | 25632000 |
| Q9ZPV9 | Syntaxin-112 | *Arabidopsis thaliana* | MESNIVTISR | Deamidation (NQ) | 2 | 1149.57 | -1.58 | 1384100 |
| Q9ZR72 | ABC transporter B family member 1 | *Arabidopsis thaliana* | LAFKDQANSFWR | 2 Deamidation (NQ),Di-oxidation W | 2 | 1515.70 | 3.14 | 501870 |
| Q9ZU23 | Jacalin-related lectin 5 | *Arabidopsis thaliana* | ESNTGSESNTNSSPQK | Glu->pyro-Glu,Deamidation (NQ) | 2 | 1648.68 | -1.88 | 161010 |
| Q9ZU40 | Sulfhydryl oxidase 2 | *Arabidopsis thaliana* | NYKPHYEKVAR | Iodination | 2 | 1529.63 | 3.54 | 80707 |
| Q9ZVF3 | MLP-like protein 328 | *Arabidopsis thaliana* | MATSGTYVTEVPLK | Oxidation Y | 2 | 1511.75 | 2.17 | 705420 |
| Q9ZVU5 | KIN17-like protein | *Arabidopsis thaliana* | VDQKELETVLPQIGGMVK | Oxidation (M) | 2 | 1999.07 | -4.19 | 139430 |
| W8JDE2 | Alstonine synthase | *Catharanthus roseus* | DPKIWPNADK | O-2H (W) | 2 | 1196.58 | -4.33 | 355200 |
|  |  |  |  |  |  |  |  |  |

***Table S4****. List of peptides and proteins identified from Bacteria/Nematoda database. For each peptide all the features (same sequence and different modifications) with the highest intensity are reported. The column Taxa-specie refers to the MaxQuant software assignment before the BLAST search.*

| **Accession Number** | **Description** | ***Specie*** | **Peptide Sequence** | **Modifications** | **Charge** | **Theoretical Mass** | **Mass error [ppm]** | **MaxQuant Intensity** |
| --- | --- | --- | --- | --- | --- | --- | --- | --- |
| **P62784** | **Histone H4** | *Caenorhabditis elegans* | TVTAMDVVYALKR | Oxidation (M) | 3 | 1481,79 | -0,012 | 113450 |
|  |  |  | VFLENVIR | Unmodified | 2 | 988,57 | 0,11 | 416690 |
|  |  |  | VFLENVIR | Deamidation (NQ) | 2 | 989,55 | -0,04 | 541050 |
|  |  |  | DNIQGITKPAIR | Unmodified | 3 | 1324,74 | 1,23 | 766490 |
|  |  |  | ISGLIYEETR | 3-iodotyrosine | 2 | 1305,51 | 0,95 | 966030 |
|  |  |  | ISGLIYEETR | Unmodified | 2 | 1179,61 | 1,15 | 3903700 |
| **P90689** | **Actin** | *Brugia malayi* | GYSFTTTAER | Unmodified | 2 | 1131,52 | 0,69 | 1342100 |
|  |  |  | SYELPDGQVITIGNER | Unmodified | 2 | 1789,88 | 0,92 | 752110 |
|  |  |  | VAPEEHPVLLTEAPLNPK | Unmodified | 3 | 1953,06 | 0,03 | 916470 |
| A0K1I7 | tRNA (guanine-N(7)-)-methyltransferase, EC 2.1.1.33 | *Arthrobacter sp. (strain FB24)* | QQAWEEHSDR | 2 Deamidation (NQ),Di-oxidation W | 2 | 1318.51 | -0.07 | 4820800 |
| A0L6H3 | 1-deoxy-D-xylulose-5-phosphate synthase, EC 2.2.1.7 | *Magnetococcus marinus (strain ATCC BAA-1437 / JCM 17883 / MC-1)* | QLNEAKLPQVAQEMR | Oxidation (M),2 Deamidation (NQ) | 2 | 1771.88 | 2.40 | 1198600 |
| A0L8B0 | Chaperone protein HtpG (Heat shock protein HtpG) | *Magnetococcus marinus (strain ATCC BAA-1437 / JCM 17883 / MC-1)* | MLEELPPAPPAKEGEEPEPPK | Unmodified | 3 | 2284.13 | -1.04 | 225720 |
| A0LRL4 | DNA-directed RNA polymerase subunit beta', RNAP subunit beta', EC 2.7.7.6 | *Acidothermus cellulolyticus (strain ATCC 43068 / 11B)* | YPKVMVAACLDEMK | Oxidation (M),Trioxidation (C) | 2 | 1717.77 | 1.63 | 694440 |
| A0LSL0 | ATP synthase subunit a | *Acidothermus cellulolyticus (strain ATCC 43068 / 11B)* | MQLLFEMLVSWVNR | Oxidation (M),Deamidation (NQ),Di-oxidation W | 2 | 1813.87 | -0.29 | 319300 |
| A0R8J2 | 50S ribosomal protein L5 | *Bacillus thuringiensis (strain Al Hakam)* | LVSVSLPR | Unmodified | 1 | 869.53 | 1.37 | 3345500 |
| A1AT72 | ATP phosphoribosyltransferase, ATP-PRT, ATP-PRTase, EC 2.4.2.17 | *Pelobacter propionicus (strain DSM 2379 / NBRC 103807 / OttBd1)* | SYFPGIDDSEMTCK | Oxidation (M) | 3 | 1664.67 | 1.69 | 122360 |
| A1AVK6 | 30S ribosomal protein S3 | *Ruthia magnifica subsp. Calyptogena magnifica* | LTNASVSRIQIERLANNAK | 3 Deamidation (NQ) | 2 | 2100.12 | -4.18 | 390630 |
| A1B8C2 | Heme A synthase, HAS, EC 1.3.- | *Paracoccus denitrificans (strain Pd 1222)* | IPTGWTPR | O-2H (W) | 2 | 940.48 | 1.00 | 153690 |
| A1K9D3 | Probable GTP-binding protein EngB | *Azoarcus sp. (strain BH72)* | YGDQVSVQLFSSLK | Oxidation Y | 2 | 1585.80 | 0.76 | 131420 |
| A1S4Q1 | 3-hydroxyacyl-[acyl-carrier-protein] dehydratase FabZ, EC 4.2.1.59 ((3R)-hydroxymyristoyl-[acyl-carrier-protein] dehydratase, (3R)-hydroxymyristoyl-ACP dehydrase) | *Shewanella amazonensis (strain ATCC BAA-1098 / SB2B)* | MSNQMNTMDIK | 3 Oxidation (M),Deamidation (NQ) | 2 | 1360.53 | -2.48 | 159190 |
| A1SVC5 | Imidazole glycerol phosphate synthase subunit HisF, EC 4.3.2.10 | *Psychromonas ingrahamii (strain 37)* | GAGEIVLNVMNQDGVR | 3 Deamidation (NQ),di-oxidation (M) | 2 | 1705.78 | -0.29 | 715030 |
| A1TKA9 | Diaminopimelate epimerase, DAP epimerase, EC 5.1.1.7 | *Acidovorax citrulli (strain AAC00-1) (Acidovorax avenae subsp. citrulli)* | VNAGYLQIEDR | Deamidation (NQ),Iodination | 2 | 1403.52 | -3.27 | 215610 |
| A1VKP2 | Formamidopyrimidine-DNA glycosylase, Fapy-DNA glycosylase, EC 3.2.2.23 | *Polaromonas naphthalenivorans (strain CJ2)* | WPLGCETQQLQGQR | 2 Deamidation (NQ),O-2H (W) | 2 | 1715.76 | -0.48 | 404590 |
| A1WYD5 | Siroheme synthase 2 | *Halorhodospira halophila (strain DSM 244 / SL1)* | EAEQAMLELLRR | Deamidation (NQ),di-oxidation (M) | 2 | 1490.74 | -0.72 | 47133000 |
| A2BQ65 | 1,4-alpha-glucan branching enzyme GlgB, EC 2.4.1.18 | *Prochlorococcus marinus (strain AS9601)* | NLIDDLNVLYK | dioxidation Y | 2 | 1350.70 | -2.36 | 724600 |
| A3CQ51 | Acetyl-coenzyme A carboxylase carboxyl transferase subunit beta, ACCase subunit beta | *Streptococcus sanguinis (strain SK36)* | EKPQAKPEVPDELFSK | Acetyl (K),Glu->pyro-Glu | 3 | 1864.96 | 0.33 | 685250 |
| A3CRC5 | DNA replication and repair protein RecF | *Streptococcus sanguinis (strain SK36)* | ITKVNHLKQSK | Acetyl (K),2 Deamidation (NQ) | 2 | 1338.75 | 1.70 | 1241800 |
| A3DE44 | Translation initiation factor IF-2 | *Hungateiclostridium thermocellum (strain ATCC 27405 )* | QNSKAAADKNR | 2 Deamidation (NQ) | 2 | 1203.58 | -2.36 | 209210 |
| A3PDJ9 | tRNA uridine(34) hydroxylase, EC 1.14 | *Prochlorococcus marinus (strain MIT 9301)* | CEKATTLLK | Acetyl (K),Trioxidation (C) | 2 | 1152.57 | -3.56 | 1692500 |
| A4G9S1 | 30S ribosomal protein S5 | *Herminiimonas arsenicoxydans* | MQSKMQSEK | Acetyl (K),Deamidation (NQ),di-oxidation (M) | 2 | 1170.49 | -3.44 | 163760 |
| A4IM55 | 50S ribosomal protein L28 | *Geobacillus thermodenitrificans (strain NG80-2)* | SHAMNANRRTWK | Oxidation (M),2 Deamidation (NQ) | 2 | 1488.69 | -1.23 | 469190 |
| A4IRW4 | UPF0354 protein GTNG_2723 | *Geobacillus thermodenitrificans (strain NG80-2)* | NSRQMYETIK | 2 Deamidation (NQ),di-oxidation (M) | 2 | 1302.58 | 2.81 | 627930 |
| A4J3Q0 | Gamma-glutamyl phosphate reductase, GPR, EC 1.2.1.41 | *Desulfotomaculum reducens (strain MI-1)* | ETILQANGK | 2 Deamidation (NQ) | 1 | 974.49 | 0.38 | 3061700 |
| A4WB84 | Cardiolipin synthase A, CL synthase, EC 2.7.8 | *Enterobacter sp. (strain 638)* | MIMIDNYIAYTGSMNMVDPR | 2 Oxidation (M),2 Deamidation (NQ) | 2 | 2368.01 | -3.74 | 3862100 |
| A4XYB5 | Sugar fermentation stimulation protein homolog | *Pseudomonas mendocina (strain ymp)* | KLPGSWEISETPQGR | O-2H (W) | 2 | 1697.84 | -1.51 | 191760 |
| A5CVI8 | ATP synthase subunit alpha, EC 7.1.2.2 | *Vesicomyosocius okutanii subsp. Calyptogena okutanii (strain HA)* | QLNAHEISDLIK | 2 Deamidation (NQ) | 2 | 1381.71 | 1.99 | 1049800 |
| A5CXL8 | 30S ribosomal protein S8 | *Vesicomyosocius okutanii subsp. Calyptogena okutanii (strain HA)* | IKLKYYDNK | Deamidation (NQ),Oxidation Y | 2 | 1200.64 | -2.21 | 404310 |
| A5EHE7 | Cysteine--tRNA ligase, EC 6.1.1.16 | *Bradyrhizobium sp. (strain BTAi1 / ATCC BAA-1182)* | HLYGAEHVTYVR | Iodination,O-2H (Y) | 2 | 1583.60 | -2.98 | 61766 |
| A5EXA4 | 30S ribosomal protein S8 | *Dichelobacter nodosus (strain VCS1703A)* | LMDPVADMLTRVRNAQR | Oxidation (M),2 Deamidation (NQ) | 2 | 2002.99 | 2.47 | 427110 |
| A5FMG2 | Leucine--tRNA ligase, EC 6.1.1.4 | *Flavobacterium johnsoniae (strain ATCC 17061 / DSM 2064 / JCM 8514 / NBRC 14942 / NCIMB 11054 / UW101) (Cytophaga johnsonae)* | FKAWREDFNTAEFIFDENGK | 2 Deamidation (NQ) | 3 | 2465.12 | -2.62 | 144780 |
| A5N4T0 | tRNA pseudouridine synthase A, EC 5.4.99.12 | *Clostridium kluyveri (strain ATCC 8527 / DSM 555 / NCIMB 10680)* | QYNAITIQQR | 2 Deamidation (NQ) | 2 | 1235.61 | -0.15 | 3040500 |
| A5UI44 | Glutamate 5-kinase, EC 2.7.2.11 | *Haemophilus influenzae (strain PittGG)* | YNSDSLQLIKGR | Acetyl (K) | 3 | 1434.75 | -0.74 | 252210 |
| A5V5D1 | Serine hydroxymethyltransferase, SHMT, Serine methylase, EC 2.1.2.1 | *Sphingomonas wittichii (strain RW1 / DSM 6014 / JCM 10273)* | WYKAVQYGVR | dioxidation Y,O-2H (Y) | 2 | 1314.64 | 1.38 | 940520 |
| A6GYJ4 | Phosphoribosylaminoimidazole-succinocarboxamide synthase, EC 6.3.2.6 | *Flavobacterium psychrophilum (strain ATCC 49511 / DSM 21280 / CIP 103535 / JIP02/86)* | GQILNQIATK | 2 Deamidation (NQ) | 2 | 1086.59 | 0.75 | 723120 |
| A6L2M8 | Alpha-1,3-galactosidase B, EC 3.2.1.n1, EC 3.2.1.n2 | *Bacteroides vulgatus (strain ATCC 8482 / DSM 1447 / JCM 5826 / NBRC 14291 / NCTC 11154)* | SIDGLIFKNNVIR | Deamidation (NQ) | 2 | 1488.83 | -0.41 | 370410 |
| A6L7P5 | Lysine--tRNA ligase, EC 6.1.1.6 | *Bacteroides vulgatus (strain ATCC 8482 / DSM 1447 / JCM 5826 / NBRC 14291 / NCTC 11154)* | VSVAGRMMSR | Oxidation (M),di-oxidation (M) | 2 | 1140.54 | 1.91 | 3876600 |
| A6LA97 | Aspartate carbamoyltransferase, EC 2.1.3.2 | *Parabacteroides distasonis (strain ATCC 8503 / DSM 20701 / CIP 104284 / JCM 5825 / NCTC 11152)* | VKNVYILRNDMLR | Oxidation (M),2 Deamidation (NQ) | 2 | 1650.88 | 2.83 | 1785900 |
| A6M1Z4 | Argininosuccinate synthase, EC 6.3.4.5 | *Clostridium beijerinckii (strain ATCC 51743 / NCIMB 8052) (Clostridium acetobutylicum)* | IGGENAIGITDMVENR | Oxidation (M) | 3 | 1703.81 | -4.38 | 69175 |
| A6Q6I2 | DNA-directed RNA polymerase subunit beta, RNAP subunit beta, EC 2.7.7.6 | *Sulfurovum sp. (strain NBC37-1)* | LNGNIELNEAKR | Acetyl (K),2 Deamidation (NQ) | 2 | 1413.71 | -0.24 | 277880 |
| A6T3L2 | DNA-directed RNA polymerase subunit beta', RNAP subunit beta', EC 2.7.7.6 | *Janthinobacterium sp. (strain Marseille)* | AMMDQLKVEDVTKR | Acetyl (K),Deamidation (NQ),di-oxidation (M) | 2 | 1737.83 | 0.18 | 545590 |
| A6TCV9 | Alanine--tRNA ligase, EC 6.1.1.7 | *Klebsiella pneumoniae subsp. pneumoniae (strain ATCC 700721 / MGH 78578)* | YAWELLTGENWFALPKEK | Deamidation (NQ),O-2H (W) | 2 | 2209.07 | -3.68 | 696570 |
| A6TXC7 | Recombination protein RecR | *Alkaliphilus metalliredigens (strain QYMF)* | EVCNICSDPNR | Deamidation (NQ),Trioxidation (C) | 2 | 1411.53 | 3.86 | 84855 |
| A6VMR3 | Ribosomal RNA large subunit methyltransferase K/L | *Actinobacillus succinogenes (strain ATCC 55618 / DSM 22257 / 130Z)* | KPLFATCAR | Trioxidation (C) | 2 | 1110.55 | -1.59 | 276050 |
| A6VUQ2 | 23S rRNA (uracil(1939)-C(5))-methyltransferase RlmD, EC 2.1.1.190 | *Marinomonas sp. (strain MWYL1)* | NAWEDWAEK | Deamidation (NQ),Trp->Kynurenine | 2 | 1152.47 | 1.07 | 376320 |
| A6VYK1 | Phospho-N-acetylmuramoyl-pentapeptide-transferase, EC 2.7.8.13 | *Marinomonas sp. (strain MWYL1)* | MLLLLTAYLSK | Oxidation (M),O-2H (Y) | 2 | 1294.72 | -2.70 | 1436800 |
| A7GVW5 | Imidazole glycerol phosphate synthase subunit HisF, EC 4.3.2.10 | *Campylobacter curvus (strain 525.92)* | EVQERGAGEILLTSMDK | Oxidation (M),Glu->pyro-Glu | 2 | 1872.93 | 2.22 | 715920 |
| A7H186 | Ribonuclease 3, EC 3.1.26.3 (Ribonuclease III, RNase III) | *Campylobacter curvus (strain 525.92)* | EAQQMAAKIALEKMK | 2 di-oxidation (M) | 2 | 1752.88 | 3.04 | 347570 |
| A7H375 | Bifunctional purine biosynthesis protein PurH | *Campylobacter jejuni subsp. doylei (strain ATCC BAA-1458 / RM4099 / 269.97)* | KNQNDENFR | 2 Deamidation (NQ) | 2 | 1165.50 | 4.33 | 186710 |
| A7HLW9 | Glycine--tRNA ligase alpha subunit, EC 6.1.1.14 | *Fervidobacterium nodosum (strain ATCC 35602 / DSM 5306 / Rt17-B1)* | MYLQDVIK | Oxidation (M),Deamidation (NQ) | 2 | 1025.51 | 4.11 | 701910 |
| A7MKC0 | Glutamyl-tRNA reductase, GluTR, EC 1.2.1.70 | *Cronobacter sakazakii (strain ATCC BAA-894) (Enterobacter sakazakii)* | TELYLSVEEQDNLHDKLVR | Deamidation (NQ) | 3 | 2301.15 | -2.34 | 402340 |
| A7ZE62 | Alanine--tRNA ligase, EC 6.1.1.7 | *Campylobacter concisus (strain 13826)* | VENLVNEWIVNGANAK | Deamidation (NQ),Trp->Kynurenine | 2 | 1773.89 | 1.39 | 1045300 |
| A8EQW8 | Dual-specificity RNA methyltransferase RlmN, EC 2.1.1.192 (23S rRNA (adenine(2503)-C(2))-methyltransferase) | *Arcobacter butzleri (strain RM4018)* | ENYPIDIMQIVK | Oxidation (M),Glu->pyro-Glu,Deamidation (NQ) | 2 | 1460.72 | 1.68 | 1315600 |
| A8EQW9 | Glutamate--tRNA ligase 1, EC 6.1.1.17 | *Arcobacter butzleri (strain RM4018)* | EMLELFDPSNINK | Glu->pyro-Glu,Deamidation (NQ) | 2 | 1531.72 | -0.27 | 875620 |
| A8EZB8 | Glutamate--tRNA ligase 2, EC 6.1.1.17 | *Rickettsia canadensis (strain McKiel)* | DSWRIWTK | O-2H (W),Oxidation (W) | 2 | 1120.53 | -3.30 | 2564500 |
| A8F962 | Cysteine--tRNA ligase, EC 6.1.1.16 | *Bacillus pumilus (strain SAFR-032)* | TINIYNTLTRK | Iodination | 2 | 1461.65 | -0.08 | 9072100 |
| A8FDQ3 | UPF0154 protein BPUM_1692 | *Bacillus pumilus (strain SAFR-032)* | YMMSYLKK | Acetyl (K),Oxidation (M) | 2 | 1120.53 | -2.47 | 2564500 |
| A8FRR3 | Undecaprenyl-phosphate 4-deoxy-4-formamido-L-arabinose transferase, EC 2.4.2.53 | *Shewanella sediminis (strain HAW-EB3)* | YPSKLINKLVK | Iodination | 2 | 1427.70 | 0.02 | 415230 |
| A8G9Z9 | DNA mismatch repair protein MutS | *Serratia proteamaculans (strain 568)* | QDNLLAAIWQDAR | 2 Deamidation (NQ),Trp->Kynurenine | 2 | 1518.73 | -0.08 | 11834000 |
| A8GFE3 | UPF0229 protein Spro_2732 | *Serratia proteamaculans (strain 568)* | EYEDLQAK | Glu->pyro-Glu | 2 | 976.45 | -1.03 | 310910 |
| A8GZ14 | CinA-like protein | *Shewanella pealeana (strain ATCC 700345 / ANG-SQ1)* | ERLENWFTR | Glu->pyro-Glu,O-2H (W) | 2 | 1245.59 | -0.02 | 850730 |
| A8L6K4 | LexA repressor, EC 3.4.21.88 | *Frankia sp. (strain EAN1pec)* | MTSQERGTR | Oxidation (M),Deamidation (NQ) | 2 | 1081.48 | 4.33 | 4949700 |
| A8MHN7 | NH(3)-dependent NAD(+) synthetase, EC 6.3.1.5 | *Alkaliphilus oremlandii (strain OhILAs) (Clostridium oremlandii (strain OhILAs))* | TQNIQKNIDQVVEWLR | Oxidation (W) | 2 | 1999.05 | 4.16 | 290350 |
| A8Q2R5 | WD repeat-containing protein 48 homolog | *Brugia malayi (Filarial nematode worm)* | LQLIDSESR | Deamidation (NQ) | 2 | 1060.54 | 0.10 | 634310 |
| A9BD99 | Trigger factor, TF, EC 5.2.1.8 (PPIase) | *Prochlorococcus marinus (strain MIT 9211)* | VVFDPKK | Acetyl (K) | 1 | 873.50 | -1.80 | 25884000 |
| A9GI17 | Protein translocase subunit SecA | *Sorangium cellulosum (strain So ce56) (Polyangium cellulosum (strain So ce56))* | DNMKFSALEYAQR | Deamidation (NQ),Oxidation Y | 2 | 1588.72 | -2.24 | 1202000 |
| A9NFI4 | Acetate kinase, EC 2.7.2.1 | *Acholeplasma laidlawii (strain PG-8A)* | KYGFHGTSHQYVSELAHARLGK | Deamidation (NQ),Iodination | 3 | 2612.14 | 1.42 | 30217000 |
| B0S1F0 | UPF0365 protein FMG_0772 | *Finegoldia magna (strain ATCC 29328 / DSM 20472 / WAL 2508) (Peptostreptococcus magnus)* | LQTDQAEADKR | Deamidation (NQ) | 2 | 1274.61 | 1.67 | 54090 |
| B0SSV1 | Arginine--tRNA ligase, EC 6.1.1.19 (Arginyl-tRNA synthetase, ArgRS) | *Leptospira biflexa serovar Patoc (strain Patoc 1 / ATCC 23582 / Paris)* | NLVLTELESAVSSYLTK | dioxidation Y | 2 | 1897.99 | -0.63 | 741590 |
| B0T0B1 | D-mannonate dehydratase Caul1427, ManD, EC 4.2.1.8 | *Caulobacter sp. (strain K31)* | LEDGTMYNW | Oxidation (M),Oxidation (W) | 2 | 1159.45 | -1.56 | 250570 |
| B0UCS0 | 30S ribosomal protein S2 | *Methylobacterium sp. (strain 4-46)* | IARDGWINQAR | 2 Deamidation (NQ) | 2 | 1300.65 | 3.65 | 182200 |
| B0UT85 | tRNA 5-methylaminomethyl-2-thiouridine biosynthesis bifunctional protein MnmC, tRNA mnm(5)s(2)U biosynthesis bifunctional protein | *Histophilus somni (strain 2336) (Haemophilus somnus)* | RECLCGIKNAIPQQNTK | 3 Deamidation (NQ) | 2 | 2031.97 | 3.79 | 183340 |
| B1IN35 | GTP 3',8-cyclase, EC 4.1.99.22 | *Clostridium botulinum (strain Okra / Type B1)* | NYVDSSQALLSKINETIYNK | 2 Deamidation (NQ) | 3 | 2301.14 | 3.88 | 309440 |
| B1N017 | Phosphoglucosamine mutase, EC 5.4.2.10 | *Leuconostoc citreum (strain KM20)* | IMYITGKFMNEQGR | Acetyl (K),2 Deamidation (NQ) | 2 | 1730.80 | -1.79 | 1195400 |
| B1VA77 | Ribosome-recycling factor, RRF (Ribosome-releasing factor) | *Phytoplasma australiense* | IQILTDKNMKMIEK | Oxidation (M),Deamidation (NQ),di-oxidation (M) | 2 | 1752.90 | 2.99 | 12887000 |
| B1VDJ5 | Probable transcriptional regulatory protein cu0933 | *Corynebacterium urealyticum (strain ATCC 43042 / DSM 7109)* | TAMTKNGGNMADAGSVSYQFER | 2 Oxidation (M),2 Deamidation (NQ) | 2 | 2367.99 | 2.25 | 4526800 |
| B1WTI3 | Biotin synthase, EC 2.8.1.6 | *Crocosphaera subtropica (strain ATCC 51142 / BH68) (Cyanothece sp. (strain ATCC 51142))* | KAGIQACTGGIMGMGETWEDRVDLAISLR | di-oxidation (M) | 3 | 3166.52 | -2.02 | 151830 |
| B1YJV2 | Glutamyl-tRNA reductase, GluTR, EC 1.2.1.70 | *Exiguobacterium sibiricum (strain DSM 17290 / CIP 109462 / JCM 13490 / 255-15)* | EQVSFGEHEMKGAVVALR | Acetyl (K),Oxidation (M),Deamidation (NQ) | 2 | 2044.99 | 2.02 | 500790 |
| B1ZT51 | tRNA dimethylallyltransferase, EC 2.5.1.75 | *Opitutus terrae (strain DSM 11246 / JCM 15787 / PB90-1)* | EAMNIAGYVAAARR | Oxidation (M),Glu->pyro-Glu,Deamidation (NQ) | 2 | 1490.73 | 4.37 | 5220600 |
| B2IK78 | 50S ribosomal protein L18 | *Beijerinckia indica subsp. indica (strain ATCC 9039 / DSM 1715 / NCIB 8712)* | MAKDIEATERR | Acetyl (K),Oxidation (M) | 3 | 1376.67 | -0.73 | 286180 |
| B2J5Z9 | Nitrogenase-stabilizing/protective protein NifW | *Nostoc punctiforme (strain ATCC 29133 / PCC 73102)* | MTGTIDEFKK | Acetyl (K),Oxidation (M) | 2 | 1226.59 | -1.34 | 988810 |
| B2JKI4 | Isocitrate dehydrogenase kinase/phosphatase, IDH kinase/phosphatase, IDHK/P, EC 2.7.11.5, EC 3.1.3.- | *Paraburkholderia phymatum (strain DSM 17167 / CIP 108236 / LMG 21445 / STM815) (Burkholderia phymatum)* | PAISTEYIENDEPAAKPTYRAYYPGK | Deamidation (NQ) | 3 | 2944.41 | -4.05 | 4049300 |
| B2KCB2 | Proline--tRNA ligase, EC 6.1.1.15 (Prolyl-tRNA synthetase, ProRS) | *Elusimicrobium minutum (strain Pei191)* | KLTQYYLPTLKEAPK | Acetyl (K),Deamidation (NQ) | 2 | 1835.01 | -1.23 | 2091000 |
| B2KE31 | DNA ligase, EC 6.5.1.2 (Polydeoxyribonucleotide synthase [NAD(+)]) | *Elusimicrobium minutum (strain Pei191)* | YEDEVAIR | Unmodified | 2 | 993.48 | 1.36 | 917830 |
| B2UAK3 | Phosphatidylglycerol--prolipoprotein diacylglyceryl transferase, EC 2.5.1.145 | *Ralstonia pickettii (strain 12J)* | PMGAVSGAFLLGYGVFR | Oxidation (M),Iodination | 2 | 1882.79 | 4.35 | 350910 |
| B2UB00 | DNA ligase, EC 6.5.1.2 (Polydeoxyribonucleotide synthase [NAD(+)]) | *Ralstonia pickettii (strain 12J)* | AAWLRAELNR | Trp->Kynurenine | 2 | 1202.65 | -1.10 | 197730 |
| B2UMT4 | 50S ribosomal protein L22 | *Akkermansia muciniphila (strain ATCC BAA-835 / Muc)* | TLKSALANAENNAELSVDTLVVK | Deamidation (NQ) | 3 | 2400.27 | -2.44 | 413810 |
| B2UTK2 | UDP-N-acetylmuramate--L-alanine ligase, EC 6.3.2.8 | *Helicobacter pylori (strain Shi470)* | ALGVEINIPHDPKAIK | Deamidation (NQ) | 2 | 1714.96 | 0.97 | 446350 |
| B2UV86 | Ribonuclease HII, RNase HII, EC 3.1.26.4 | *Helicobacter pylori (strain Shi470)* | SLANQIKIDGNTAFGLNK | Acetyl (K) | 2 | 1945.03 | -2.40 | 552610 |
| B2UWX8 | S-ribosylhomocysteine lyase, EC 4.4.1.21 | *Clostridium botulinum (strain Alaska E43 / Type E3)* | AKLEGIIDLSPMGCR | Acetyl (K),Oxidation (M) | 2 | 1716.85 | -2.19 | 1249400 |
| B2UXS9 | Peptidyl-tRNA hydrolase, PTH, EC 3.1.1.29 | *Clostridium botulinum (strain Alaska E43 / Type E3)* | QDINSAMNKFNGFKANK | Oxidation (M),2 Deamidation (NQ) | 2 | 1943.90 | 0.59 | 221110 |
| B2UYT8 | Ketol-acid reductoisomerase (NADP(+)), KARI, EC 1.1.1.86 | *Clostridium botulinum (strain Alaska E43 / Type E3)* | LYCWNNEDK | 2 Deamidation (NQ),Trioxidation (C) | 2 | 1290.47 | 4.23 | 4080500 |
| B3PB55 | Aspartate--tRNA(Asp/Asn) ligase, EC 6.1.1.23 | *Cellvibrio japonicus (strain Ueda107) (Pseudomonas fluorescens subsp. cellulosa)* | RTEMQNNLR | Deamidation (NQ) | 2 | 1161.56 | 2.87 | 121840 |
| B3PIS9 | ATP synthase subunit alpha, EC 7.1.2.2 ( | *Cellvibrio japonicus (strain Ueda107) (Pseudomonas fluorescens subsp. cellulosa)* | LKASRTW | Trp->Kynurenine | 2 | 864.48 | -0.09 | 432230 |
| B4S5G5 | Polyribonucleotide nucleotidyltransferase, EC 2.7.7.8 | *Prosthecochloris aestuarii (strain DSM 271 / SK 413)* | DAQMIDTLTDDADKR | Oxidation (M) | 3 | 1722.77 | -0.60 | 1047800 |
| B4SJT3 | Glycerol kinase, EC 2.7.1.30 | *Stenotrophomonas maltophilia (strain R551-3)* | EQIAAQWGLDR | Deamidation (NQ),Di-oxidation W | 2 | 1318.62 | -2.33 | 1732700 |
| B4U1E8 | Translation initiation factor IF-2 | *Streptococcus equi subsp. zooepidemicus (strain MGCS10565)* | QVAEQKAK | Deamidation (NQ) | 2 | 901.49 | -0.10 | 917080 |
| B5XT16 | Probable intracellular septation protein A | *Klebsiella pneumoniae (strain 342)* | ELALPQQVWSR | 2 Deamidation (NQ),Di-oxidation W | 2 | 1359.67 | -1.56 | 2391800 |
| B5XTR4 | Thiosulfate sulfurtransferase GlpE, EC 2.8.1.1 | *Klebsiella pneumoniae (strain 342)* | MEQFECINVEEAHQK | Oxidation (M),Deamidation (NQ) | 2 | 1907.80 | 0.28 | 885410 |
| B5YIL6 | Methionyl-tRNA formyltransferase, EC 2.1.2.9 | *Thermodesulfovibrio yellowstonii (strain ATCC 51303 / DSM 11347 / YP87)* | GKNLQAPEIKK | 2 Acetyl (K),2 Deamidation (NQ) | 2 | 1310.71 | 2.39 | 1410600 |
| B5ZAS4 | Recombination protein RecR | *Ureaplasma urealyticum serovar 10 (strain ATCC 33699 / Western)* | TNIIKCKYCSNFGNK | 2 Acetyl (K),Trioxidation (C) | 2 | 1977.89 | -0.48 | 265830 |
| B5ZBT4 | DNA polymerase IV, Pol IV, EC 2.7.7.7 | *Ureaplasma urealyticum serovar 10 (strain ATCC 33699 / Western)* | IQNEVKNK | Deamidation (NQ) | 2 | 972.52 | 0.13 | 111950 |
| B6ELD2 | Cell division protein ZapC | *Aliivibrio salmonicida (strain LFI1238) (Vibrio salmonicida (strain LFI1238))* | MAKMQVVNSSYYALVG | Oxidation (M),Deamidation (NQ) | 2 | 1776.84 | 0.45 | 409720 |
| B6IRS7 | Adenylate kinase, AK, EC 2.7.4.3 (ATP-AMP transphosphorylase) | *Rhodospirillum centenum (strain ATCC 51521 / SW)* | QIEAIIAG | Deamidation (NQ) | 1 | 814.44 | 2.55 | 414470 |
| B6JMR9 | Ferrochelatase, EC 4.99.1.1 ( | *Helicobacter pylori (strain P12)* | LNNLENNAAKSPK | Deamidation (NQ) | 2 | 1412.73 | 1.41 | 766370 |
| B7GM51 | ATP-dependent helicase/nuclease subunit A | *Anoxybacillus flavithermus (strain DSM 21510 / WK1)* | VSWMNDPK | Deamidation (NQ),Trp->Kynurenine | 2 | 980.43 | 2.46 | 411260 |
| B7VGZ3 | UDP-2,3-diacylglucosamine hydrolase, EC 3.6.1.54 | *Vibrio atlanticus (strain LGP32) (Vibrio splendidus (strain Mel32))* | SLDIMDVTQQEVENVMK | 2 Deamidation (NQ),di-oxidation (M) | 2 | 2011.90 | -2.77 | 1431000 |
| B7VJ13 | 4-hydroxy-tetrahydrodipicolinate reductase, HTPA reductase, EC 1.17.1.8 | *Vibrio atlanticus (strain LGP32) (Vibrio splendidus (strain Mel32))* | MTFANGAIKAAVWLNDK | Oxidation (W) | 3 | 1864.95 | 4.08 | 667990 |
| B8DNL3 | Trigger factor, TF, EC 5.2.1.8 (PPIase) | *Desulfovibrio vulgaris (strain Miyazaki F / DSM 19637)* | EAVTSSYMQSRTQLVK | Deamidation (NQ),di-oxidation (M) | 3 | 1859.89 | -2.42 | 277550 |
| B8E1E7 | 30S ribosomal protein S8 | *Dictyoglomus turgidum (strain Z-1310 / DSM 6724)* | IKNANMR | Oxidation (M),2 Deamidation (NQ) | 2 | 863.42 | -3.72 | 4703300 |
| B8FGS9 | Tyrosine--tRNA ligase, EC 6.1.1.1 | *Desulfatibacillum aliphaticivorans* | VKDLDGAELNLAK | Deamidation (NQ) | 2 | 1385.74 | 2.07 | 652070 |
| B8FXD8 | ATP-dependent helicase/deoxyribonuclease subunit B, EC 3.1.-.-, EC 3.6.4.12 | *Desulfitobacterium hafniense (strain DCB-2 / DSM 10664)* | EETAGLNETR | Glu->pyro-Glu,Deamidation (NQ) | 2 | 1101.49 | 3.85 | 463000 |
| B8I813 | Energy-coupling factor transporter transmembrane protein EcfT, ECF transporter T component EcfT | *Ruminiclostridium cellulolyticum (strain ATCC 35319 / DSM 5812 / JCM 6584 / H10) (Clostridium cellulolyticum)* | AQSSRGADFDSGNMIER | 2 Deamidation (NQ) | 2 | 1841.79 | 2.05 | 1581000 |
| B9DIJ2 | N-acetylneuraminate lyase, NAL, Neu5Ac lyase, EC 4.1.3.3 | *Staphylococcus carnosus (strain TM300)* | ILELGLYPTLK | Iodination | 2 | 1384.65 | -2.24 | 545360 |
| B9DIS2 | ATP-dependent helicase/nuclease subunit A, EC 3.1 | *Staphylococcus carnosus (strain TM300)* | ANLYGLYNK | Deamidation (NQ),dioxidation Y,O-2H (Y) | 2 | 1101.50 | 2.19 | 659410 |
| B9DJR3 | Putative hydro-lyase Sca_2211, EC 4.2.1.- | *Staphylococcus carnosus (strain TM300)* | MTDLQNIEPQALRK | Oxidation (M) | 3 | 1671.86 | -2.52 | 367650 |
| B9DKF4 | 3-methyl-2-oxobutanoate hydroxymethyltransferase, EC 2.1.2.11 | *Staphylococcus carnosus (strain TM300)* | QLSNLFDMKKNSEK | Oxidation (M),Deamidation (NQ) | 2 | 1697.83 | 3.71 | 191680 |
| B9DMM2 | 60 kDa chaperonin (GroEL protein) (Protein Cpn60) | *Staphylococcus carnosus (strain TM300)* | QIAENAGLEGSIIVEK | 2 Deamidation (NQ) | 3 | 1671.86 | 0.47 | 320510 |
| B9DMT3 | DNA polymerase IV, Pol IV, EC 2.7.7.7 | *Staphylococcus carnosus (strain TM300)* | EVMHTNGIYNGQDLYNK | Oxidation (M),Deamidation (NQ) | 2 | 2011.89 | -1.94 | 1431000 |
| B9DYC1 | 50S ribosomal protein L5 | *Clostridium kluyveri (strain NBRC 12016)* | IVINMGVGEAKDNPK | Oxidation (M),Deamidation (NQ) | 2 | 1600.81 | -3.78 | 116280 |
| B9E0C6 | ATP-dependent helicase/deoxyribonuclease subunit B, EC 3.1.-.-, EC 3.6.4.12 | *Clostridium kluyveri (strain NBRC 12016)* | NILLYKIIEENKNK | Deamidation (NQ),Oxidation Y | 2 | 1747.97 | 0.60 | 1961100 |
| B9KFG7 | DNA-directed RNA polymerase subunit beta, RNAP subunit beta, EC 2.7.7.6 | *Campylobacter lari (strain RM2100 / D67 / ATCC BAA-1060)* | SFDYFLNIDAK | O-2H (Y) | 2 | 1345.62 | 3.57 | 347730 |
| B9L7R9 | CTP synthase, EC 6.3.4.2 (Cytidine 5'-triphosphate synthase) | *Nautilia profundicola (strain ATCC BAA-1463 / DSM 18972 / AmH)* | SPNKVIMSFVENAYKCQR | Oxidation (M),O-2H (Y) | 2 | 2200.04 | -2.37 | 770350 |
| B9M5C0 | 1-deoxy-D-xylulose 5-phosphate reductoisomerase, DXP reductoisomerase, EC 1.1.1.267 | *Geobacter daltonii (strain DSM 22248 / JCM 15807 / FRC-32)* | SLSSIEDVIEIDQWGR | O-2H (W) | 3 | 1859.89 | 0.17 | 214960 |
| C0QQ02 | Catalase-peroxidase, CP, EC 1.11.1.21 (Peroxidase/catalase) | *Persephonella marina (strain DSM 14350 / EX-H1)* | SPAGAWQWVAVNPDR | Oxidation (W) | 3 | 1668.80 | 2.69 | 111830 |
| C0R573 | Leucine--tRNA ligase, EC 6.1.1.4 (Leucyl-tRNA synthetase, LeuRS) | *Wolbachia sp. subsp. Drosophila simulans (strain wRi)* | LWRMVMQLK | Di-oxidation W | 2 | 1235.65 | -2.28 | 158530 |
| C0Z9F1 | Endonuclease MutS2, EC 3.1.-.- | *Brevibacillus brevis (strain 47 / JCM 6285 / NBRC 100599)* | AQLAWSMK | Deamidation (NQ),Di-oxidation W | 2 | 966.45 | -1.88 | 1899400 |
| C0ZL89 | Lysylphosphatidylglycerol biosynthesis bifunctional protein LysX | *Rhodococcus erythropolis (strain PR4 / NBRC 100887)* | MESPPENPRQAR | Oxidation (M),Deamidation (NQ) | 2 | 1427.65 | -3.28 | 2088900 |
| C4K799 | 50S ribosomal protein L15 | *Hamiltonella defensa subsp. Acyrthosiphon pisum (strain 5AT)* | IIPYQSQYAK | Deamidation (NQ),dioxidation Y,O-2H (Y) | 2 | 1256.59 | -3.07 | 407320 |
| C4L0I9 | Peptidase T, EC 3.4.11.4 (Aminotripeptidase, Tripeptidase) | *Exiguobacterium sp. (strain ATCC BAA-1283 / AT1b)* | MVNSMKLAMAFQNR | 2 Oxidation (M),2 Deamidation (NQ) | 2 | 1673.76 | 2.56 | 222740 |
| C4LL52 | 50S ribosomal protein L3 | *Corynebacterium kroppenstedtii (strain DSM 44385 / JCM 11950 / CIP 105744 / CCUG 35717)* | VDADSNLLLIK | Deamidation (NQ) | 3 | 1200.66 | -0.16 | 24975 |
| C4XIN9 | DNA-directed RNA polymerase subunit beta, RNAP subunit beta, EC 2.7.7.6 | *Desulfovibrio magneticus (strain ATCC 700980 / DSM 13731 / RS-1)* | ETNEAVAEVLDEYDR | Glu->pyro-Glu,Deamidation (NQ) | 2 | 1734.76 | 1.45 | 553450 |
| C6CU08 | UPF0756 membrane protein Pjdr2_2290 | *Paenibacillus sp. (strain JDR-2)* | MTGELILVGLIVIGLIGR | Oxidation (M) | 2 | 1882.13 | -0.41 | 67405 |
| C6DF90 | Flagellar brake protein YcgR (Cyclic di-GMP binding protein YcgR) | *Pectobacterium carotovorum subsp. carotovorum (strain PC1)* | LQNEKKK | 2 Acetyl (K),Deamidation (NQ) | 2 | 971.53 | 0.11 | 120300 |
| C6E6M9 | Phosphoribosylformylglycinamidine cyclo-ligase, EC 6.3.3.1 (AIR synthase) (AIRS) | *Geobacter sp. (strain M21)* | ITYKDAGVDIDAGNTFVQMIK | Deamidation (NQ) | 3 | 2299.14 | 4.12 | 680850 |
| D9XF45 | Phosphonoacetaldehyde reductase, EC 1.1.1.309 | *Streptomyces viridochromogenes (strain DSM 40736 / JCM 4977 / BCRC 1201 / Tue 494)* | LLRYNHAVPAGDCADPR | Oxidation Y | 2 | 1939.93 | -0.14 | 235230 |
| G5ECQ3 | Serine/threonine-protein kinase sel-5, EC 2.7.11.1 (Suppressor/enhancer of lin-12 protein 5) | *Caenorhabditis elegans* | KEPKQLSENK | Deamidation (NQ) | 2 | 1200.64 | -0.27 | 435220 |
| I3VRU1 | Alpha-galacturonidase, EC 3.2.1.67 | *Thermoanaerobacterium saccharolyticum (strain DSM 8691 / JW/SL-YS485)* | INVLGINHFTWIDNAR | Oxidation (W) | 2 | 1897.98 | 2.15 | 741590 |
| I6XD69 | Mycoketide-CoA synthase, EC 2.3.1.295 (Polyketide synthase Pks12) | *Mycobacterium tuberculosis (strain ATCC 25618 / H37Rv)* | RQVDDSVAAAK | Deamidation (NQ) | 2 | 1159.58 | 2.30 | 733460 |
| O02305 | Nuclear hormone receptor family member nhr-217 | *Caenorhabditis elegans* | YFVFSNVWIESTWLYSLAK | Deamidation (NQ),Oxidation Y | 2 | 2369.16 | 2.70 | 108720 |
| O30409 | Tyrocidine synthase 3 (Tyrocidine synthase III | *Brevibacillus parabrevis* | NVTRLVMHTNYVQVR | Deamidation (NQ),O-2H (Y) | 2 | 1843.94 | 2.44 | 164830 |
| O32483 | Phosphoenolpyruvate carboxylase, PEPC, PEPCase, EC 4.1.1.31 | *Rhodopseudomonas palustris (strain ATCC BAA-98 / CGA009)* | YEPYRLAVSGIYAR | 2 Di-iodination,O-2H (Y) | 4 | 2174.43 | -1.03 | 2947400 |
| O34171 | Flagellum-specific ATP synthase, EC 7.1.2.2 | *Agrobacterium fabrum (strain C58 / ATCC 33970) (Agrobacterium tumefaciens (strain C58))* | MTMPESMLSESK | 3 Oxidation (M) | 2 | 1417.58 | 2.95 | 79808 |
| O44518 | Cytoplasmic FMR1-interacting protein homolog | *Caenorhabditis elegans* | QLASCMLLDKR | Acetyl (K) | 3 | 1375.70 | -3.92 | 479960 |
| O44568 | Probable peptide chain release factor 1, mitochondrial, MRF-1, MtRF-1 | *Caenorhabditis elegans* | SELSQLR | Deamidation (NQ) | 2 | 832.43 | 0.31 | 3807700 |
| O51149 | 30S ribosomal protein S2 | *Borrelia burgdorferi (strain ATCC 35210 / B31 / CIP 102532 / DSM 4680)* | KEISQLNR | Acetyl (K),Deamidation (NQ) | 2 | 1029.55 | 0.36 | 6200000 |
| O51889 | ATP-dependent DNA helicase Rep, EC 3.6.4.12 | *Buchnera aphidicola subsp. Schizaphis graminum (strain Sg)* | PMNLLQIVK | Oxidation (M),2 Deamidation (NQ) | 2 | 1072.58 | 2.85 | 8095900 |
| O66503 | Ribonucleoside-diphosphate reductase subunit alpha, EC 1.17.4.1 | *Aquifex aeolicus (strain VF5)* | ILYDRYLVR | Iodination,O-2H (Y) | 2 | 1349.56 | 0.23 | 196370 |
| O67383 | Argininosuccinate lyase, ASAL, EC 4.3.2.1 (Arginosuccinase) | *Aquifex aeolicus (strain VF5)* | NLKNMIK | Oxidation (M),Deamidation (NQ) | 2 | 876.47 | -3.17 | 835520 |
| O83085 | Putative adenosine/adenine deaminase, EC 3.5.4.- | *Treponema pallidum (strain Nichols)* | KSLEYYRSQPK | Oxidation Y,dioxidation Y | 2 | 1445.72 | -2.23 | 313420 |
| O85140 | Malonyl CoA-acyl carrier protein transacylase, MCT, EC 2.3.1.39 | *Salmonella typhimurium (strain LT2 / SGSC1412 / ATCC 700720)* | AGAACKAAGAK | Acetyl (K),Trioxidation (C) | 2 | 1064.49 | -0.50 | 37214000 |
| O86028 | Methylmalonyl-CoA mutase, MCM, EC 5.4.99.2 | *Rhizobium meliloti (strain 1021) (Ensifer meliloti) (Sinorhizobium meliloti)* | DWEALAEKELR | Di-oxidation W | 3 | 1390.67 | 0.01 | 240160 |
| P00145 | Cytochrome c' | *Pararhodospirillum photometricum (Rhodospirillum photometricum)* | ADAVKSADAAK | Acetyl (K) | 2 | 1087.55 | 0.58 | 120140 |
| P08007 | Oligopeptide transport ATP-binding protein OppF | *Salmonella typhimurium (strain LT2 / SGSC1412 / ATCC 700720)* | SDIQMIFQDPLASLNPR | Oxidation (M),2 Deamidation (NQ) | 2 | 1961.94 | 4.16 | 136400 |
| P08310 | Muconate cycloisomerase 1, EC 5.5.1.1 | *Pseudomonas putida (Arthrobacter siderocapsulatus)* | VDVNQYWDESQAIR | Deamidation (NQ) | 3 | 1722.78 | -1.75 | 1532200 |
| P08655 | Uncharacterized 19.7 kDa protein in mercuric resistance operon | *Staphylococcus aureus* | MASVGMSEEEAKNSGR | Acetyl (K),Oxidation (M),Deamidation (NQ),di-oxidation (M) | 2 | 1772.72 | -0.93 | 27810000 |
| P0A211 | Protein FliZ | *Salmonella typhi* | IALRKYQQYK | dioxidation Y,O-2H (Y) | 2 | 1355.72 | 2.25 | 4175700 |
| P0A4N2 | Maltodextrin transport system permease protein MalC | *Streptococcus pneumoniae (strain ATCC BAA-255 / R6)* | KLHAFDMEDV | Acetyl (K),di-oxidation (M) | 2 | 1277.56 | -0.15 | 378610 |
| P0ACE2 | Hydrogenase-2 large chain, HYD2, EC 1.12.99.6 | *Shigella flexneri* | AWASGTMWR | Oxidation (M),O-2H (W),Trp->Kynurenine | 2 | 1098.46 | -3.06 | 304230 |
| P0C0A4 | tRNA(Met) cytidine acetate ligase, EC 6.3.4.- | *Bacillus cereus (strain ATCC 14579 / DSM 31 / JCM 2152 / NBRC 15305 / NCIMB 9373 / NRRL B-3711)* | AILSQNSSIQAQTIK | Deamidation (NQ) | 2 | 1601.86 | 3.07 | 1289400 |
| P0C0F2 | DNA polymerase III subunit alpha, EC 2.7.7.7 | *Streptococcus pyogenes* | AMSKKNLQEMQK | 2 Acetyl (K),2 Deamidation (NQ) | 2 | 1520.72 | 2.58 | 1276100 |
| P12746 | Transcriptional activator protein LuxR | *Aliivibrio fischeri (Vibrio fischeri)* | KNINADDTYR | 2 Deamidation (NQ) | 2 | 1210.55 | 1.64 | 1915600 |
| P12990 | ATP synthase gamma chain | *Vibrio alginolyticus* | AVTDMQTWR | Oxidation (M),Oxidation (W) | 2 | 1138.51 | 3.98 | 64338 |
| P17155 | Photosystem I P700 chlorophyll a apoprotein A2, EC 1.97.1.12 (PsaB) | *Synechococcus sp. (strain ATCC 27264 / PCC 7002 / PR-6) (Agmenellum quadruplicatum)* | EILNAHQPPSGK | Glu->pyro-Glu,Deamidation (NQ) | 2 | 1272.65 | 2.68 | 743460 |
| P17452 | Dermonecrotic toxin, DNT (Mitogenic toxin) (PMT) | *Pasteurella multocida* | MLEDSDVQIRSNSEATR | Oxidation (M) | 2 | 1965.91 | -0.97 | 171710 |
| P19424 | Endoglucanase, EC 3.2.1.4 (Alkaline cellulase) | *Bacillus sp. (strain KSM-635)* | GDNMILVGNPNWSQR | Oxidation (M),2 Deamidation (NQ) | 2 | 1717.77 | 3.36 | 556360 |
| P19580 | Capsule biosynthesis protein CapB | *Bacillus anthracis* | MIYWFTDEEQPIK | Oxidation (M),Deamidation (NQ),Trp->Kynurenine | 2 | 1719.77 | -3.18 | 179410 |
| P20588 | Type-2 restriction enzyme HinfI, R.HinfI, EC 3.1.21.4 | *Haemophilus influenzae* | GNIYVPENTNIRR | 2 Deamidation (NQ) | 2 | 1546.77 | 1.19 | 326320 |
| P21458 | DNA translocase SpoIIIE (Stage III sporulation protein E) | *Bacillus subtilis (strain 168)* | SNMQSSKK | Acetyl (K),di-oxidation (M) | 2 | 982.44 | -3.73 | 186750 |
| P21880 | Dihydrolipoyl dehydrogenase, EC 1.8.1.4 | *Bacillus subtilis (strain 168)* | VLNSTGALALK | Deamidation (NQ) | 2 | 1086.63 | 0.81 | 2776800 |
| P24242 | HTH-type transcriptional regulator AscG | *Escherichia coli (strain K12)* | LIANGKWTPASGAEGVEMLLER | O-2H (W) | 3 | 2355.19 | -4.34 | 322370 |
| P27350 | Alpha-amylase, EC 3.2.1.1 (1,4-alpha-D-glucan glucanohydrolase) | *Streptomyces thermoviolaceus* | LSNPNAYWK | Deamidation (NQ),Trp->Kynurenine | 2 | 1096.52 | -0.25 | 2954700 |
| P27509 | Uncharacterized protein in pqqA 5'region (ORF X) | *Klebsiella pneumoniae* | QVNALKMQIDVSHMNEK | 2 Deamidation (NQ),di-oxidation (M) | 2 | 2017.94 | -1.07 | 107360 |
| P29190 | Phosphoenolpyruvate carboxykinase [GTP], PEPCK, EC 4.1.1.32 | *Haemonchus contortus (Barber pole worm)* | VIDWIVRR | Di-oxidation W | 2 | 1087.61 | -0.70 | 1439300 |
| P30435 | Xylose isomerase, EC 5.3.1.5 | *Thermoanaerobacterium saccharolyticum* | ENYVFWGGR | Deamidation (NQ),Trp->Kynurenine | 2 | 1131.50 | -1.76 | 2772300 |
| P31776 | Penicillin-binding protein 1A, PBP-1a, PBP1a (Penicillin-binding protein A) | *Haemophilus influenzae (strain ATCC 51907 / DSM 11121 / KW20 / Rd)* | PGQKMWQPK | Di-oxidation W | 2 | 1130.55 | 2.51 | 252560 |
| P37627 | Uncharacterized protein YhiJ | *Escherichia coli (strain K12)* | IDDWQIER | O-2H (W) | 2 | 1087.49 | 4.20 | 117250 |
| P41843 | Uncharacterized protein T20B12.3 | *Caenorhabditis elegans* | LLQCHWNQSVR | O-2H (W) | 3 | 1453.69 | -2.06 | 1749900 |
| P42375 | 60 kDa chaperonin (GroEL protein) (Protein Cpn60) | *Porphyromonas gingivalis (strain ATCC BAA-308 / W83)* | VTVDKDNTTIVNGAGNK | Deamidation (NQ) | 2 | 1745.88 | -4.42 | 151750 |
| P44399 | L-fuculokinase, EC 2.7.1.51 (L-fuculose kinase) | *Haemophilus influenzae (strain ATCC 51907 / DSM 11121 / KW20 / Rd)* | NVLWNQIR | 2 Deamidation (NQ),Oxidation (W) | 2 | 1059.53 | 3.89 | 154790 |
| P44421 | Methionine aminopeptidase, MAP, MetAP, EC 3.4.11.18 (Peptidase M) | *Haemophilus influenzae (strain ATCC 51907 / DSM 11121 / KW20 / Rd)* | ICHEYMVNEQK | 2 Deamidation (NQ),Trioxidation (C) | 2 | 1499.59 | 4.11 | 261370 |
| P45081 | ATP-binding/permease protein CydC | *Haemophilus influenzae (strain ATCC 51907 / DSM 11121 / KW20 / Rd)* | ATYRTQFLEFIQAQAELLLFNAEDK | Deamidation (NQ),Oxidation Y | 3 | 2975.49 | 2.00 | 529200 |
| P45273 | Riboflavin synthase, RS, EC 2.5.1.9 | *Haemophilus influenzae (strain ATCC 51907 / DSM 11121 / KW20 / Rd)* | VGDYVNIER | Deamidation (NQ) | 2 | 1064.51 | 0.68 | 362680 |
| P45743 | Isochorismatase, EC 3.3.2.1 (2,3 dihydro-2,3 dihydroxybenzoate synthase) | *Bacillus subtilis (strain 168)* | NVFTCENIRK | 2 Deamidation (NQ) | 2 | 1281.60 | -4.01 | 576960 |
| P47306 | Uncharacterized glycosyltransferase MG060, EC 2.4.-.- | *Mycoplasma genitalium (strain ATCC 33530 / G-37 / NCTC 10195)* | VGNSMSIPWHSSR | Oxidation (M),Di-oxidation W | 2 | 1504.67 | -1.34 | 192190 |
| P47311 | Putative ABC transporter ATP-binding protein MG065 | *Mycoplasma genitalium (strain ATCC 33530 / G-37 / NCTC 10195)* | QEILLNNWFNSNER | 4 Deamidation (NQ) | 2 | 1779.80 | -0.21 | 139310 |
| P47615 | Threonine--tRNA ligase, EC 6.1.1.3 (Threonyl-tRNA synthetase, ThrRS) | *Mycoplasma genitalium (strain ATCC 33530 / G-37 / NCTC 10195)* | VNLDDNQDR | 2 Deamidation (NQ) | 2 | 1089.46 | 0.87 | 495180 |
| P50193 | Modification methylase HphIB, M.HphIB, EC 2.1.1.72 | *Haemophilus parahaemolyticus* | LNNTYKQALAFALMR | Deamidation (NQ),Oxidation Y | 2 | 1769.91 | -4.01 | 220890 |
| P52980 | 1,4-alpha-glucan branching enzyme GlgB, EC 2.4.1.18 | *Kitasatospora aureofaciens (Streptomyces aureofaciens)* | MPGDWWQQR | Oxidation (M),Oxidation (W) | 2 | 1234.52 | -3.95 | 351540 |
| P54298 | Acyl-homoserine-lactone synthase LuxM, AHL synthase LuxM, EC 2.3.1.184 | *Vibrio harveyi (Beneckea harveyi)* | AYFHSGYCDLNDDGK | Deamidation (NQ),O-2H (Y) | 2 | 1775.67 | 0.94 | 39318 |
| P55725 | Uncharacterized protein y4yQ | *Sinorhizobium fredii (strain NBRC 101917 / NGR234)* | VQQWFDHRTR | Oxidation (W) | 2 | 1387.67 | -3.56 | 423140 |
| P57304 | S-adenosylmethionine decarboxylase proenzyme, AdoMetDC, SAMDC, EC 4.1.1.50 | *Buchnera aphidicola subsp. Acyrthosiphon pisum (strain APS) (Acyrthosiphon pisum symbiotic bacterium)* | EFNLKNYLFNINLENLEKEER | Glu->pyro-Glu,2 Deamidation (NQ),Iodination | 3 | 2778.20 | -0.34 | 836100 |
| P57400 | Translation initiation factor IF-1 | *Buchnera aphidicola subsp. Acyrthosiphon pisum (strain APS) (Acyrthosiphon pisum symbiotic bacterium)* | EENIEMQGVVIDTLPNTMFR | Oxidation (M),Glu->pyro-Glu,Deamidation (NQ) | 2 | 2334.09 | 0.55 | 1072900 |
| P60549 | Guanylate kinase, EC 2.7.4.8 (GMP kinase) | *Bdellovibrio bacteriovorus (strain ATCC 15356 / DSM 50701 / NCIB 9529 / HD100)* | MANAEKEIR | Oxidation (M),Deamidation (NQ) | 2 | 1077.51 | 1.20 | 402630 |
| P61343 | Aspartyl/glutamyl-tRNA(Asn/Gln) amidotransferase subunit B, Asp/Glu-ADT subunit B, EC 6.3.5.- | *Geobacter sulfurreducens (strain ATCC 51573 / DSM 12127 / PCA)* | TVFDEMWQSDK | Oxidation (M),O-2H (W) | 2 | 1414.57 | -2.81 | 1233800 |
| P62351 | Histidine--tRNA ligase, EC 6.1.1.21 | *Wolbachia pipientis wMel* | EALILYFTKYQNDLSEDSKNR | Deamidation (NQ) | 3 | 2547.25 | 3.09 | 137250 |
| P71362 | L-2,4-diaminobutyrate decarboxylase, DABA decarboxylase, DABA-DC, EC 4.1.1.86 | *Haemophilus influenzae (strain ATCC 51907 / DSM 11121 / KW20 / Rd)* | ADYLNSEYDEAHGVPNLVAKSLQTTRR | Deamidation (NQ),Oxidation Y | 3 | 3063.49 | -2.08 | 5312000 |
| P72831 | Uncharacterized protein slr1298 | *Synechocystis sp. (strain PCC 6803 / Kazusa)* | QEPALTMTNYLAR | 2 Deamidation (NQ),dioxidation Y | 2 | 1540.71 | 0.45 | 321030 |
| P75262 | Probable ABC transporter permease protein MG189 homolog | *Mycoplasma pneumoniae (strain ATCC 29342 / M129)* | VVVVANWNQPEK | 2 Deamidation (NQ),O-2H (W) | 2 | 1397.68 | 4.10 | 1295300 |
| P75453 | Uncharacterized protein MPN_109 | *Mycoplasma pneumoniae (strain ATCC 29342 / M129)* | FKLNFHEKINQK | Acetyl (K),2 Deamidation (NQ) | 2 | 1588.82 | -1.34 | 250260 |
| P75551 | Oligopeptide transport ATP-binding protein OppF | *Mycoplasma pneumoniae (strain ATCC 29342 / M129)* | AQMAEELQNKPR | Acetyl (K),Deamidation (NQ),di-oxidation (M) | 2 | 1488.69 | -2.84 | 585800 |
| P75810 | Inner membrane protein YbjJ | *Escherichia coli (strain K12)* | TVNSSRNALKR | Acetyl (K),2 Deamidation (NQ) | 2 | 1288.67 | 2.53 | 183460 |
| P85097 | Respiratory nitrate reductase alpha chain, EC 1.7.5.1 | *Bradyrhizobium sp.* | ELIIQIAREFADNADKTHGR | Acetyl (K),2 Deamidation (NQ) | 3 | 2340.17 | 4.16 | 324920 |
| P91406 | Glutamate carboxypeptidase 2 homolog, EC 3.4.17.21 | *Caenorhabditis elegans* | MPYVGVGAQTVSTSLTGAPMVK | Deamidation (NQ),Oxidation Y | 3 | 2210.10 | -2.62 | 564810 |
| P94559 | Putative metallophosphoesterase YsnB, EC 3.1.4.- | *Bacillus subtilis (strain 168)* | MNVLIISDSHGLEEELQTIAK | Deamidation (NQ) | 3 | 2340.19 | -0.65 | 1028700 |
| P9WFX5 | Anthranilate phosphoribosyltransferase, EC 2.4.2.18 | *Mycobacterium tuberculosis (strain ATCC 25618 / H37Rv)* | WVRFGRQI | Deamidation (NQ),Oxidation (W) | 2 | 1077.57 | 0.02 | 489090 |
| P9WGB9 | Glycolipid sulfotransferase Rv1373, EC 2.8.2.- | *Mycobacterium tuberculosis (strain ATCC 25618 / H37Rv)* | DPRDAAVSMLYQSANMNEDRMR | Oxidation (M),2 Deamidation (NQ) | 2 | 2587.11 | -3.00 | 1726800 |
| P9WHJ3 | Protein RecA (Recombinase A) [Cleaved into: Endonuclease PI-MtuI, EC 3.1.-.- (Mtu RecA intein) ] | *Mycobacterium tuberculosis (strain ATCC 25618 / H37Rv)* | ALELAVAQIEKSYGK | Deamidation (NQ),O-2H (Y) | 2 | 1633.86 | -2.32 | 309730 |
| P9WQ27 | Probable 1,4-alpha-glucan branching enzyme Rv3031, EC 2.4.1.18 | *Mycobacterium tuberculosis (strain ATCC 25618 / H37Rv)* | DWQVWSGAK | Deamidation (NQ),O-2H (W),Di-oxidation W | 2 | 1122.46 | 2.06 | 336920 |
| Q02HI0 | Alkaline phosphatase L, L-AP, EC 3.1.3.1 | *Pseudomonas aeruginosa (strain UCBPP-PA14)* | SESSGTTELFTR | Unmodified | 2 | 1313.61 | 0.96 | 2042600 |
| Q02NN7 | Phenylalanine--tRNA ligase alpha subunit, EC 6.1.1.20 | *Pseudomonas aeruginosa (strain UCBPP-PA14)* | EKVQDALNAR | Glu->pyro-Glu,2 Deamidation (NQ) | 2 | 1126.56 | 2.34 | 522200 |
| Q03GG8 | S-adenosylmethionine synthase, AdoMet synthase, EC 2.5.1.6 (MAT) (Methionine adenosyltransferase) | *Pediococcus pentosaceus (strain ATCC 25745 / CCUG 21536 / LMG 10740 / 183-1w)* | IADQISDSILDELLKNDPDAR | Unmodified | 4 | 2340.18 | -2.72 | 636650 |
| Q07736 | Type I restriction enzyme EcoAI R protein, R.EcoAI, EC 3.1.21.3 | *Escherichia coli* | RALVNLNPEQVK | 2 Deamidation (NQ) | 2 | 1381.76 | 3.09 | 1598900 |
| Q08A11 | Adenosine deaminase, EC 3.5.4.4 | *Shewanella frigidimarina (strain NCIMB 400)* | ENNLVDFLKK | Deamidation (NQ) | 3 | 1219.64 | 2.91 | 67991 |
| Q09423 | Osmotic avoidance abnormal protein 8 | *Caenorhabditis elegans* | SLWEMFQR | Oxidation (M),Oxidation (W) | 2 | 1127.51 | -3.76 | 115680 |
| Q09647 | Tubulin polyglutamylase ttll-4, EC 6 | *Caenorhabditis elegans* | SIGINRLEQLCR | Deamidation (NQ),Trioxidation (C) | 2 | 1506.75 | -1.84 | 862350 |
| Q0A7E2 | Protein GrpE (HSP-70 cofactor) | *Alkalilimnicola ehrlichii (strain ATCC BAA-1101 / DSM 17681 / MLHE-1)* | AEMQNIQRR | Deamidation (NQ) | 2 | 1145.56 | 1.46 | 571200 |
|  |  |  | AEMQNIQRR | 2 Deamidation (NQ) | 2 | 1146.55 | 0.13 | 274700 |
|  |  |  | AEMQNIQRR | Oxidation (M),Deamidation (NQ) | 2 | 1161.56 | 0.69 | 232080 |
| Q0ACS7 | Chromosomal replication initiator protein DnaA | *Alkalilimnicola ehrlichii (strain ATCC BAA-1101 / DSM 17681 / MLHE-1)* | ENSLWKQCLRR | 2 Deamidation (NQ) | 2 | 1490.73 | 4.16 | 39173000 |
| Q0AGX5 | Tryptophan synthase beta chain, EC 4.2.1.20 | *Nitrosomonas eutropha (strain DSM 101675 / C91 / Nm57)* | MKMYDLPDK | 2 Oxidation (M) | 2 | 1171.53 | 0.64 | 227130 |
| Q0AQ76 | Thiazole synthase, EC 2.8.1.10 | *Maricaulis maris (strain MCS10)* | MEDAVTMDTAFEDK | Oxidation (M),di-oxidation (M) | 2 | 1649.64 | 3.75 | 215260 |
| Q0AYK9 | Probable chemoreceptor glutamine deamidase CheD, EC 3.5.1.44 | *Syntrophomonas wolfei subsp. wolfei (strain DSM 2245B / Goettingen)* | IGERNSVAVKENLQLHR | Deamidation (NQ) | 2 | 1963.06 | 0.13 | 525930 |
| Q0BIM8 | Indole-3-glycerol phosphate synthase, IGPS, EC 4.1.1.48 | *Burkholderia ambifaria (strain ATCC BAA-244 / AMMD) (Burkholderia cepacia (strain AMMD))* | QAAGHAAVIAEVKK | Deamidation (NQ) | 3 | 1392.77 | 0.73 | 1954000 |
| Q0I3A1 | Deoxyguanosinetriphosphate triphosphohydrolase-like protein | *Haemophilus somnus (strain 129Pt) (Histophilus somni)* | LEPYTPNAGMNLTR | Oxidation (M),2 Deamidation (NQ) | 2 | 1593.73 | -1.08 | 619510 |
| Q0SF99 | 50S ribosomal protein L10 | *Rhodococcus jostii (strain RHA1)* | ALKNFAKDNK | 2 Acetyl (K),Deamidation (NQ) | 2 | 1232.64 | 1.09 | 698830 |
| Q0VPG1 | Chaperone protein HtpG (Heat shock protein HtpG) | *Alcanivorax borkumensis (strain ATCC 700651 / DSM 11573 / NCIMB 13689 / SK2)* | ELLQDYGPVQK | Unmodified | 2 | 1288.67 | -1.44 | 26998000 |
| Q115Y7 | Triosephosphate isomerase, TIM, TPI, EC 5.3.1.1 | *Trichodesmium erythraeum (strain IMS101)* | RQYFGETDETVNMR | Oxidation (M),2 Deamidation (NQ) | 2 | 1762.75 | -4.47 | 308200 |
| Q136E5 | Enolase, EC 4.2.1.11 | *Rhodopseudomonas palustris (strain BisB5)* | IEQELGAQAHYAGK | Deamidation (NQ),dioxidation Y | 2 | 1546.73 | 2.77 | 6726000 |
| Q136Q9 | Heme A synthase, HAS, EC 1.3 | *Rhodopseudomonas palustris (strain BisB5)* | PVTGTLPPLNDAQWRDAFEGYK | Oxidation (W) | 3 | 2490.22 | -3.19 | 287560 |
| Q168B1 | Probable transaldolase, EC 2.2.1.2 | *Roseobacter denitrificans (strain ATCC 33942 / OCh 114) (Erythrobacter sp. (strain OCh 114)) (Roseobacter denitrificans)* | GLQQFMSDWEK | Oxidation (M),Deamidation (NQ),Di-oxidation W | 2 | 1416.59 | 1.57 | 260100 |
| Q16CP0 | 1-deoxy-D-xylulose-5-phosphate synthase 2, EC 2.2.1.7 DXPS 2) | *Roseobacter denitrificans (strain ATCC 33942 / OCh 114) (Erythrobacter sp. (strain OCh 114)) (Roseobacter denitrificans)* | LAEVQKAGEALAAR | Deamidation (NQ) | 2 | 1426.78 | 1.96 | 7680800 |
| Q17WJ2 | Lipid-A-disaccharide synthase, EC 2.4.1.182 | *Helicobacter acinonychis (strain Sheeba)* | IKKQDSHK | Acetyl (K),Deamidation (NQ) | 2 | 1025.55 | 0.74 | 239120 |
| Q18286 | Exocyst complex component 6 (Exocyst complex component Sec15) | *Caenorhabditis elegans* | ISHYDKNIQK | Deamidation (NQ),Oxidation Y | 2 | 1261.63 | 2.40 | 165220 |
| Q182K8 | Leucine--tRNA ligase, EC 6.1.1.4 (Leucyl-tRNA synthetase, LeuRS) | *Clostridioides difficile (strain 630) (Peptoclostridium difficile)* | SVYNFKEVESKWQK | 2 Deamidation (NQ) | 2 | 1772.86 | 2.32 | 470720 |
| Q1AXU6 | 60 kDa chaperonin (GroEL protein) (Protein Cpn60) | *Rubrobacter xylanophilus (strain DSM 9941 / NBRC 16129 / PRD-1)* | NVAAGANPVILRNGIEK | Deamidation (NQ) | 2 | 1735.96 | 4.20 | 133640 |
| Q1GKQ4 | Xylose isomerase, EC 5.3.1.5 | *Ruegeria sp. (strain TM1040) (Silicibacter sp.)* | MLNMVVEYKHK | Oxidation (M),Deamidation (NQ) | 2 | 1407.69 | -0.77 | 197440 |
| Q1II25 | Probable DNA ligase, EC 6.5.1.1 (Polydeoxyribonucleotide synthase [ATP]) | *Koribacter versatilis (strain Ellin345)* | EIAEMDEWFR | Oxidation (M),O-2H (W) | 2 | 1354.55 | -1.14 | 520820 |
| Q1LSW6 | UDP-N-acetylmuramate--L-alanine ligase, EC 6.3.2.8 | *Baumannia cicadellinicola subsp. Homalodisca coagulata* | SLCCTIRNTSK | Deamidation (NQ),2 Trioxidation (C) | 2 | 1435.59 | 2.51 | 844040 |
| Q1LTM9 | Leucine--tRNA ligase, EC 6.1.1.4 | *Baumannia cicadellinicola subsp. Homalodisca coagulata* | VIKLDLAAMSNDNK | Acetyl (K),Oxidation (M) | 2 | 1588.81 | 4.20 | 238270 |
| Q1MRS8 | DNA ligase, EC 6.5.1.2 | *Lawsonia intracellularis (strain PHE/MN1-00)* | LQAQRYSLR | Deamidation (NQ),dioxidation Y | 2 | 1166.60 | -0.14 | 555070 |
| Q1WUQ3 | UPF0342 protein LSL_0473 | *Lactobacillus salivarius (strain UCC118)* | TLQEQQMQGTLDEK | 2 Deamidation (NQ) | 2 | 1649.75 | -1.90 | 1616200 |
| Q1WV55 | Bifunctional protein GlmU | *Lactobacillus salivarius (strain UCC118)* | NSNIGPYSHLR | dioxidation Y | 2 | 1288.62 | -1.80 | 7127100 |
| Q21281 | Transmembrane matrix receptor MUP-4 | *Caenorhabditis elegans* | SLNQVCQQKNGK | 2 Deamidation (NQ) | 2 | 1404.67 | -1.97 | 2840700 |
| Q21HH5 | Ribosome-recycling factor, RRF | *Saccharophagus degradans (strain 2-40 / ATCC 43961 / DSM 17024)* | RAADEIQQITNK | Deamidation (NQ) | 2 | 1386.71 | 1.23 | 1709200 |
| Q27355 | Transcription factor lin-26 | *Caenorhabditis elegans* | LSNNKFNQMLSK | Oxidation (M) | 2 | 1438.72 | -3.28 | 4676400 |
| Q2G1N0 | Staphyloferrin B transporter | *Staphylococcus aureus (strain NCTC 8325 / PS 47)* | SFQCLLCTQQTCR | 2 Trioxidation (C) | 2 | 1796.71 | 3.39 | 23830000 |
| Q2GE84 | Lipoyl synthase, EC 2.8.1.8 (Lip-syn, LS) | *Neorickettsia sennetsu (strain ATCC VR-367 / Miyayama) (Ehrlichia sennetsu)* | YATDEEFQYYKEAAYAR | Iodination,Di-iodination,dioxidation Y,O-2H (Y) | 4 | 2540.60 | 3.30 | 4656600 |
| Q2J537 | DNA integrity scanning protein DisA | *Frankia casuarinae (strain DSM 45818 / CECT 9043 / CcI3)* | RYVLDGSAAILSR | O-2H (Y) | 2 | 1433.76 | 1.98 | 130030 |
| Q2J750 | Ribosomal protein S12 methylthiotransferase RimO, S12 MTTase, S12 methylthiotransferase, EC 2.8.4.4 | *Frankia casuarinae (strain DSM 45818 / CECT 9043 / CcI3)* | VTDLVEQLTAAR | Unmodified | 2 | 1314.71 | 0.96 | 417570 |
| Q2K711 | Probable potassium transport system protein kup 2 | *Rhizobium etli (strain CFN 42 / ATCC 51251)* | LDINFGFMDDPDVTR | Oxidation (M),Deamidation (NQ) | 2 | 1770.78 | -0.86 | 341520 |
| Q2NCF2 | Protoheme IX farnesyltransferase, EC 2.5.1. | *Erythrobacter litoralis (strain HTCC2594)* | TTAASPAALPADWRDFFALTK | Di-oxidation W | 3 | 2281.14 | -0.79 | 1139000 |
| Q2NQL6 | Elongation factor G, EF-G | *Sodalis glossinidius (strain morsitans)* | IAGSMAFKEGFMK | Acetyl (K),Oxidation (M),di-oxidation (M) | 2 | 1505.69 | -1.28 | 1238700 |
| Q2NU80 | Ribosomal RNA large subunit methyltransferase K/L | *Sodalis glossinidius (strain morsitans)* | YSSWVVIQEYVAPK | Oxidation Y,O-2H (Y) | 2 | 1697.83 | 1.90 | 119750 |
| Q2NUJ7 | Biotin synthase, EC 2.8.1.6 | *Sodalis glossinidius (strain morsitans)* | DMPLLEQMVQGVK | 2 Oxidation (M) | 2 | 1518.74 | -3.75 | 587470 |
| Q2RV14 | RNA pyrophosphohydrolase, EC 3.6.1.- ((Di)nucleoside polyphosphate hydrolase) | *Rhodospirillum rubrum (strain ATCC 11170 / ATH 1.1.1 / DSM 467 / LMG 4362 / NCIB 8255 / S1)* | QGVGIMLINAR | Oxidation (M),Gln->pyro-Glu | 2 | 1169.62 | -0.76 | 69690 |
| Q2S6P3 | Bifunctional protein GlmU | *Hahella chejuensis (strain KCTC 2396)* | NIDGWTRPVKK | Oxidation (W) | 2 | 1328.72 | 1.12 | 23493000 |
| Q2Y6B3 | GTP cyclohydrolase 1, EC 3.5.4.16 | *Nitrosospira multiformis (strain ATCC 25196 / NCIMB 11849 / C 71)* | MDSNDSEFGIGDWQR | Oxidation (M),Deamidation (NQ),Di-oxidation W | 2 | 1804.68 | 2.91 | 2316200 |
| Q30RJ0 | Bifunctional protein FolD | *Sulfurimonas denitrificans (strain ATCC 33889 / DSM 1251) (Thiomicrospira denitrificans (strain ATCC 33889 / DSM 1251))* | EDMVKGGAIIVDIGINR | Deamidation (NQ) | 2 | 1799.95 | -0.23 | 250870 |
| Q313K1 | Ribonuclease HII, RNase HII, EC 3.1.26.4 | *Desulfovibrio alaskensis (strain G20) (Desulfovibrio desulfuricans (strain G20))* | INILQATYR | 2 Deamidation (NQ) | 2 | 1092.58 | 0.07 | 582750 |
| Q31CU1 | Phosphoglucosamine mutase, EC 5.4.2.10 | *Prochlorococcus marinus (strain MIT 9312)* | VDSLSSEIANKLILEINKIMN | Acetyl (K),Deamidation (NQ),di-oxidation (M) | 3 | 2418.26 | -1.81 | 205240 |
| Q39CJ6 | Taurine import ATP-binding protein TauB, EC 7.6.2.7 | *Burkholderia lata (strain ATCC 17760 / DSM 23089 / LMG 22485 / NCIMB 9086 / R18194 / 383)* | MSTLEVRQVSVAYPGER | Deamidation (NQ),di-oxidation (M) | 2 | 1953.95 | -1.11 | 337920 |
| Q39D34 | LPS-assembly protein LptD | *Burkholderia lata (strain ATCC 17760 / DSM 23089 / LMG 22485 / NCIMB 9086 / R18194 / 383)* | VINVGYRYTR | Deamidation (NQ),Oxidation Y | 2 | 1256.65 | 1.87 | 49872000 |
| Q39Z73 | Ornithine carbamoyltransferase, OTCase, EC 2.1.3.3 | *Geobacter metallireducens (strain ATCC 53774 / DSM 7210 / GS-15)* | TVWDWAQK | Di-oxidation W | 2 | 1064.49 | -0.96 | 58879 |
| Q3A091 | GTP cyclohydrolase FolE2, EC 3.5.4.16 | *Pelobacter carbinolicus (strain DSM 2380 / NBRC 103641 / GraBd1)* | ARSLMEYR | Oxidation (M),Oxidation Y | 2 | 1056.50 | -1.98 | 3378600 |
| Q3AVR4 | Urease accessory protein UreD | *Synechococcus sp. (strain CC9902)* | LGRTAAGEDLGQGCWR | O-2H (W) | 3 | 1759.81 | 2.46 | 148100 |
| Q3AZ50 | Octanoyltransferase, EC 2.3.1.181 | *Synechococcus sp. (strain CC9902)* | LCDWMPGLR | Oxidation (W) | 2 | 1162.53 | -3.98 | 426410 |
| Q3IIX4 | 30S ribosomal protein S2 | *Pseudoalteromonas translucida (strain TAC 125)* | DLEIQSQDGTFEKLTK | Deamidation (NQ) | 3 | 1851.91 | -4.24 | 327170 |
| Q3KL70 | Probable endonuclease 4, EC 3.1.21.2 | *Chlamydia trachomatis serovar A (strain ATCC VR-571B / DSM 19440 / HAR-13)* | DIGATTVQLFTANQRQWK | 3 Deamidation (NQ) | 2 | 2079.03 | 1.19 | 164800 |
| Q3M7N8 | DNA replication and repair protein RecF | *Trichormus variabilis (strain ATCC 29413 / PCC 7937) (Anabaena variabilis)* | ISTSNSIR | Deamidation (NQ) | 2 | 877.45 | 0.46 | 6156600 |
| Q3SWQ3 | tRNA (guanine-N(7)-)-methyltransferase, EC 2.1.1.33 | *Nitrobacter winogradskyi (strain ATCC 25391 / DSM 10237 / CIP 104748 / NCIMB 11846 / Nb-255)* | SADWQDPWSGYTMTR | O-2H (W),Oxidation (W) | 2 | 1829.73 | 0.06 | 498570 |
| Q3YRF1 | Aspartate--tRNA(Asp/Asn) ligase, EC 6.1.1.23 | *Ehrlichia canis (strain Jake)* | AFSIAGYTQEMVDK | Oxidation (M),O-2H (Y) | 2 | 1588.71 | 2.34 | 122760 |
| Q3Z0T2 | 2-dehydro-3-deoxyphosphooctonate aldolase, EC 2.5.1.55 | *Shigella sonnei (strain Ss046)* | PQFVSPGQMGNIVDKFK | Oxidation (M),Deamidation (NQ) | 3 | 1907.95 | 0.49 | 109420 |
| Q44264 | Heat shock protein 15 homolog, HSP15 | *Aeromonas salmonicida* | FYKTRSLAR | Iodination | 2 | 1266.54 | -3.47 | 337060 |
| Q46VM0 | tRNA modification GTPase MnmE, EC 3.6.- | *Cupriavidus pinatubonensis (strain JMP 134 / LMG 1197) (Cupriavidus necator (strain JMP 134))* | IDVAPTVGGMMFSGNR | Oxidation (M) | 2 | 1666.78 | 1.04 | 144030 |
| Q470L8 | Formate-dependent phosphoribosylglycinamide formyltransferase | *Cupriavidus pinatubonensis (strain JMP 134 / LMG 1197) (Cupriavidus necator (strain JMP 134))* | TIAMSDPEQLK | Oxidation (M),Deamidation (NQ) | 2 | 1248.59 | -2.35 | 145300 |
| Q474D4 | Homoserine kinase, HK, HSK, EC 2.7.1.39 | *Cupriavidus pinatubonensis (strain JMP 134 / LMG 1197) (Cupriavidus necator (strain JMP 134))* | PLSATEAAHWRDMLR | Oxidation (M),Trp->Kynurenine | 2 | 1772.86 | 2.48 | 396420 |
| Q475Y4 | Putative pterin-4-alpha-carbinolamine dehydratase 1, PHS 1, EC 4.2.1.96 | *Cupriavidus pinatubonensis (strain JMP 134 / LMG 1197) (Cupriavidus necator (strain JMP 134))* | LESQTCTPCR | Deamidation (NQ),2 Trioxidation (C) | 2 | 1347.49 | 2.73 | 150260 |
| Q48A24 | DNA mismatch repair protein MutL | *Colwellia psychrerythraea (strain 34H / ATCC BAA-681) (Vibrio psychroerythus)* | LVEQSWQNK | Deamidation (NQ),Oxidation (W) | 2 | 1147.55 | 2.37 | 106860 |
| Q48LX4 | Membrane-bound lytic murein transglycosylase F, EC 4.2.2.n1 (Murein lyase F) | *Pseudomonas savastanoi pv. phaseolicola (strain 1448A / Race 6) (Pseudomonas syringae pv. phaseolicola (strain 1448A / Race 6))* | YEKHFQTSAKK | Acetyl (K),Deamidation (NQ) | 2 | 1408.70 | 2.91 | 112680 |
| Q48PB0 | 3-oxo-tetronate kinase, EC 2.7.1.217 (3-dehydrotetronate 4-kinase) | *Pseudomonas savastanoi pv. phaseolicola (strain 1448A / Race 6) (Pseudomonas syringae pv. phaseolicola (strain 1448A / Race 6))* | VLQAQTRHK | 2 Deamidation (NQ) | 2 | 1081.59 | 4.26 | 1638400 |
| Q493C8 | 1-deoxy-D-xylulose 5-phosphate reductoisomerase, DXP reductoisomerase, EC 1.1.1.267 | *Blochmannia pennsylvanicus (strain BPEN)* | ILLANKETLVTGGKLFMK | 2 Acetyl (K),di-oxidation (M) | 2 | 2091.16 | 1.66 | 641370 |
| Q49405 | DNA polymerase III subunit alpha, EC 2.7.7.7 | *Mycoplasma genitalium (strain ATCC 33530 / G-37 / NCTC 10195)* | QFLDKNQDLNKK | Deamidation (NQ) | 2 | 1490.77 | 1.85 | 548260 |
| Q49WR1 | Endonuclease MutS2, EC 3.1.-.- | *Staphylococcus saprophyticus subsp. saprophyticus (strain ATCC 15305 / DSM 20229 / NCIMB 8711 / NCTC 7292 / S-41)* | QSIKMELDLR | Acetyl (K),Oxidation (M) | 2 | 1289.66 | -1.78 | 443310 |
| Q4FUL7 | NADPH-dependent 7-cyano-7-deazaguanine reductase, EC 1.7.1.13 | *Psychrobacter arcticus (strain DSM 17307 / VKM B-2377 / 273-4)* | DVIGWQNDK | 2 Deamidation (NQ),O-2H (W) | 2 | 1089.46 | -2.57 | 407340 |
| Q4L6D0 | Probable CtpA-like serine protease, EC 3.4.21.- | *Staphylococcus haemolyticus (strain JCSC1435)* | IEYAYKTLNNDYYK | 2 Oxidation Y | 2 | 1828.85 | -2.80 | 107680 |
| Q4UK40 | DNA polymerase III subunit alpha, EC 2.7.7.7 | *Rickettsia felis (strain ATCC VR-1525 / URRWXCal2) (Rickettsia azadi)* | TVLNHESMKMDSR | Acetyl (K),Oxidation (M),Deamidation (NQ),di-oxidation (M) | 2 | 1637.70 | -3.86 | 112480 |
| Q4UM86 | DNA mismatch repair protein MutS | *Rickettsia felis (strain ATCC VR-1525 / URRWXCal2) (Rickettsia azadi)* | LKDQYCK | Deamidation (NQ),Oxidation Y | 2 | 970.44 | -2.10 | 578160 |
| Q5F5P1 | Aspartate carbamoyltransferase regulatory chain | *Neisseria gonorrhoeae (strain ATCC 700825 / FA 1090)* | MEAQKLSVEAIEK | Acetyl (K),Oxidation (M),Deamidation (NQ) | 2 | 1533.76 | -1.68 | 1028100 |
| Q5F651 | LPS-assembly protein LptD | *Neisseria gonorrhoeae (strain ATCC 700825 / FA 1090)* | MEGQSKVKVR | Acetyl (K),Deamidation (NQ),di-oxidation (M) | 2 | 1235.62 | -1.24 | 3040500 |
| Q5FSH9 | tRNA uridine(34) hydroxylase, EC 1.14.-.- (tRNA hydroxylation protein O) | *Gluconobacter oxydans (strain 621H) (Gluconobacter suboxydans)* | LKKEIVTMGEPDIDPR | Oxidation (M) | 2 | 1855.97 | -2.39 | 634550 |
| Q5L512 | Alanine--tRNA ligase, EC 6.1.1.7 | *Chlamydia abortus (strain DSM 27085 / S26/3) (Chlamydophila abortus)* | DQILNKLQNILDEKK | Acetyl (K) | 2 | 1853.03 | 1.69 | 986110 |
| Q5ZUJ9 | Alanine--tRNA ligase, EC 6.1.1.7 | *Legionella pneumophila subsp. pneumophila (strain Philadelphia 1 / ATCC 33152 / DSM 7513)* | VSQLLLDNKNQEK | 3 Deamidation (NQ) | 2 | 1530.78 | 0.34 | 3191200 |
| Q5ZW88 | Histidinol-phosphate aminotransferase 1, EC 2.6.1.9 | *Legionella pneumophila subsp. pneumophila (strain Philadelphia 1 / ATCC 33152 / DSM 7513)* | TNFRLTLDQIENSWK | Deamidation (NQ),O-2H (W) | 2 | 1878.91 | 3.59 | 133110 |
| Q64U07 | Isoleucine--tRNA ligase, EC 6.1.1.5 | *Bacteroides fragilis (strain YCH46)* | QRVNIDLYKEYAGR | Deamidation (NQ),Oxidation Y,dioxidation Y | 2 | 1772.87 | -0.57 | 396420 |
| Q64XW5 | Peptide chain release factor 1, RF-1 | *Bacteroides fragilis (strain YCH46)* | EEMDNSQERLPVLEEEIK | 2 Deamidation (NQ) | 2 | 2189.00 | 2.75 | 260940 |
| Q65Q30 | HTH-type transcriptional activator RhaR | *Mannheimia succiniciproducens (strain MBEL55E)* | IFKQQTNMTIAQYLQK | Oxidation (M),Deamidation (NQ),dioxidation Y | 2 | 2003.00 | -1.42 | 753200 |
| Q65R84 | 4-hydroxy-3-methylbut-2-en-1-yl diphosphate synthase (flavodoxin), EC 1.17.7.3 | *Mannheimia succiniciproducens (strain MBEL55E)* | QKERFDNEYIVDQLEAK | 2 Deamidation (NQ) | 2 | 2126.02 | 1.90 | 864530 |
| Q65T53 | Sulfite reductase [NADPH] flavoprotein alpha-component, SiR-FP, EC 1.8.1.2 | *Mannheimia succiniciproducens (strain MBEL55E)* | DQAEKIYVQDK | dioxidation Y | 2 | 1367.66 | 1.38 | 2264200 |
| Q65ZG8 | Period protein homolog lin-42 | *Caenorhabditis elegans* | NYQITYTPLDDLTDQK | Oxidation Y,dioxidation Y | 2 | 1974.91 | -1.33 | 2014700 |
| Q6AS16 | Lon protease 1, EC 3.4.21.53 | *Desulfotalea psychrophila (strain LSv54 / DSM 12343)* | DDTQTEMEKYENR | Oxidation (M),2 Deamidation (NQ) | 2 | 1675.65 | -2.96 | 173130 |
| Q6DAM0 | Phosphomethylpyrimidine synthase, EC 4.1.99.17 | *Pectobacterium atrosepticum (strain SCRI 1043 / ATCC BAA-672) (Erwinia carotovora subsp. atroseptica)* | QGMITPEMEFIAIR | Oxidation (M),Gln->pyro-Glu | 2 | 1633.78 | 1.87 | 9258800 |
| Q6F1N6 | Phosphate acyltransferase, EC 2.3.1.274 | *Mesoplasma florum (strain ATCC 33453 / NBRC 100688 / NCTC 11704 / L1) (Acholeplasma florum)* | SKGTLLQKEIYK | Iodination | 2 | 1532.71 | -0.31 | 1192700 |
| Q6GFX3 | Acetoin utilization protein AcuC | *Staphylococcus aureus (strain MRSA252)* | IIMFGGGGYNIWR | Oxidation (M),Deamidation (NQ),Di-oxidation W | 2 | 1531.71 | 3.75 | 794150 |
| Q6GGK4 | Segregation and condensation protein B | *Staphylococcus aureus (strain MRSA252)* | EASMYIEQLIEQK | Glu->pyro-Glu,Deamidation (NQ) | 2 | 1563.75 | 1.87 | 1018200 |
| Q6KHG5 | Holo-[acyl-carrier-protein] synthase, Holo-ACP synthase, EC 2.7.8.7 | *Mycoplasma mobile (strain ATCC 43663 / 163K / NCTC 11711)* | MIGVDLTK | Oxidation (M) | 1 | 891.47 | -2.44 | 1324400 |
| Q6KHS4 | 30S ribosomal protein S7 | *Mycoplasma mobile (strain ATCC 43663 / 163K / NCTC 11711)* | LINTIMLDGKK | Deamidation (NQ) | 2 | 1245.70 | -1.26 | 273960 |
| Q6MFA6 | Crossover junction endodeoxyribonuclease RuvC, EC 3.1.22.4 | *Protochlamydia amoebophila (strain UWE25)* | YQVQGMVQR | Oxidation (M),Deamidation (NQ),O-2H (Y) | 2 | 1138.51 | 1.51 | 107640 |
| Q6MQW5 | UPF0234 protein Bd0338 | *Bdellovibrio bacteriovorus (strain ATCC 15356 / DSM 50701 / NCIB 9529 / HD100)* | GSKAELQWDKK | Deamidation (NQ),Di-oxidation W | 2 | 1321.65 | 2.56 | 3594900 |
| Q6N5Q2 | 30S ribosomal protein S2 (RRP-S2) | *Rhodopseudomonas palustris (strain ATCC BAA-98 / CGA009)* | MSLPEFSMR | 2 Oxidation (M) | 2 | 1128.49 | 3.35 | 192860 |
| Q74CA7 | Exodeoxyribonuclease 7 large subunit, EC 3.1.11.6 | *Geobacter sulfurreducens (strain ATCC 51573 / DSM 12127 / PCA)* | EGLFADEHKKPIPPLPQK | Deamidation (NQ) | 2 | 2044.10 | 2.01 | 3382900 |
| Q74FU5 | NADPH-Fe(3+) oxidoreductase subunit beta, EC 1. | *Geobacter sulfurreducens (strain ATCC 51573 / DSM 12127 / PCA)* | VVVVGGGNTAIDCVR | Trioxidation (C) | 2 | 1562.77 | 1.45 | 1678700 |
| Q74K75 | SsrA-binding protein (Small protein B) | *Lactobacillus johnsonii (strain CNCM I-12250 / La1 / NCC 533)* | MKQKADNLIAQNK | 2 Deamidation (NQ) | 2 | 1502.78 | -2.09 | 768320 |
| Q7MWS5 | Protein translocase subunit SecA | *Porphyromonas gingivalis (strain ATCC BAA-308 / W83)* | IQDYVKDER | Deamidation (NQ),Iodination | 2 | 1291.46 | 2.17 | 268260 |
| Q7N8V1 | RNA polymerase-associated protein RapA, EC 3.6.4.- | *Photorhabdus laumondii subsp. laumondii (strain DSM 15139 / CIP 105565 / TT01)* | KYQSEQFK | 2 Deamidation (NQ) | 2 | 1058.49 | 1.66 | 3358600 |
| Q7NAM1 | 30S ribosomal protein S6 | *Mycoplasma gallisepticum (strain R(low / passage 15 / clone 2))* | QVQENGYFQIKGSK | Deamidation (NQ),Oxidation Y | 2 | 1641.80 | -1.42 | 522650 |
| Q7NAT4 | Cytadherence high molecular weight protein 1 | *Mycoplasma gallisepticum (strain R(low / passage 15 / clone 2))* | RNYEQLYRNQLQQNVNANYGNFR | 2 Deamidation (NQ) | 3 | 2903.37 | 0.37 | 1380700 |
| Q7NRN3 | 30S ribosomal protein S20 | *Chromobacterium violaceum (strain ATCC 12472 / DSM 30191 / JCM 1249 / NBRC 12614 / NCIMB 9131 / NCTC 9757)* | ANSAQARKR | Acetyl (K),2 Deamidation (NQ) | 2 | 1044.53 | 1.74 | 597140 |
| Q7NSS4 | Tetraacyldisaccharide 4'-kinase, EC 2.7.1.130 (Lipid A 4'-kinase) | *Chromobacterium violaceum (strain ATCC 12472 / DSM 30191 / JCM 1249 / NBRC 12614 / NCIMB 9131 / NCTC 9757)* | RLIEQHWYR | Deamidation (NQ),Oxidation (W) | 2 | 1316.66 | -2.59 | 1922300 |
| Q7SIB1 | CCA-adding enzyme, EC 2.7.7.72 | *Geobacillus stearothermophilus (Bacillus stearothermophilus)* | IYAWLMER | O-2H (W) | 2 | 1094.52 | 2.51 | 184180 |
| Q7UZW3 | Adenylate kinase, AK, EC 2.7.4.3 | *Prochlorococcus marinus subsp. pastoris (strain CCMP1986 / NIES-2087 / MED4)* | EIDLDTDLGKQVKDIMNR | Deamidation (NQ) | 3 | 2103.05 | -3.30 | 435380 |
| Q7V0U2 | Cobyric acid synthase | *Prochlorococcus marinus subsp. pastoris (strain CCMP1986 / NIES-2087 / MED4)* | MKRENIIDNLANEFK | Acetyl (K),di-oxidation (M) | 3 | 1907.94 | -1.27 | 1289000 |
| Q7V5N8 | Ribose-5-phosphate isomerase A, EC 5.3.1.6 | *Prochlorococcus marinus (strain MIT 9313)* | MADLQTQMKQAVAAAAVEQIK | Oxidation (M),2 Deamidation (NQ) | 2 | 2262.12 | -3.77 | 2433300 |
| Q7V9L7 | Trigger factor, TF, EC 5.2.1.8 (PPIase) | *Prochlorococcus marinus (strain SARG / CCMP1375 / SS120)* | MSNSQLK | 2 Deamidation (NQ) | 1 | 808.36 | -2.31 | 213890 |
| Q7VF28 | Thiazole synthase, EC 2.8.1.10 | *Helicobacter hepaticus (strain ATCC 51449 / 3B1)* | DTNIQFLPNSAGCVNAK | 3 Deamidation (NQ) | 2 | 1850.84 | -3.43 | 53274 |
| Q7VPM2 | Membrane protein insertase YidC (Foldase YidC) | *Haemophilus ducreyi (strain 35000HP / ATCC 700724)* | QQMSQEMMKLYKQEK | Oxidation (M),Deamidation (NQ),di-oxidation (M) | 2 | 1977.88 | 3.00 | 161590 |
| Q7VQM0 | Polyribonucleotide nucleotidyltransferase, EC 2.7.7.8 | *Blochmannia floridanus* | FQYGQHTVVLNTGAIAR | Deamidation (NQ),dioxidation Y | 2 | 1906.95 | 0.56 | 99759 |
| Q7VQV2 | Putative membrane protein insertion efficiency factor | *Blochmannia floridanus* | FSITCSQYGINSIR | Deamidation (NQ),Iodination | 2 | 1771.67 | -2.52 | 5635300 |
| Q7VR53 | Pyridoxine/pyridoxamine 5'-phosphate oxidase, EC 1.4.3.5 | *Blochmannia floridanus* | AIHINNNPKISLCFPWNIINR | 3 Deamidation (NQ) | 3 | 2536.29 | -0.41 | 938140 |
| Q7VRN9 | Elongation factor G, EF-G | *Blochmannia floridanus* | GIISNLENSMNIGR | Oxidation (M),3 Deamidation (NQ) | 2 | 1535.71 | 0.41 | 497340 |
| Q7VWK6 | Valine--tRNA ligase, EC 6.1.1.9 | *Bordetella pertussis (strain Tohama I / ATCC BAA-589 / NCTC 13251)* | RTFGLMHPKQAGAIEK | Deamidation (NQ) | 2 | 1783.94 | 1.54 | 1306700 |
| Q7ZAK9 | Segregation and condensation protein B | *Streptococcus mutans serotype c (strain ATCC 700610 / UA159)* | KDYADLLRQYSK | dioxidation Y,O-2H (Y) | 2 | 1544.75 | -3.27 | 1422900 |
| Q81BX6 | Putative adenine deaminase BC_3012, Adenase, Adenine aminase, EC 3.5.4.2 | *Bacillus cereus (strain ATCC 14579 / DSM 31 / JCM 2152 / NBRC 15305 / NCIMB 9373 / NRRL B-3711)* | DGSWQVNIAVK | Deamidation (NQ),O-2H (W) | 2 | 1230.59 | -4.47 | 384780 |
| Q81WK9 | Elongation factor Ts, EF-Ts | *Bacillus anthracis* | EDNFAEEVMNQVK | Oxidation (M),Glu->pyro-Glu,Deamidation (NQ) | 2 | 1550.66 | -2.41 | 124110 |
| Q82X77 | 30S ribosomal protein S8 | *Nitrosomonas europaea (strain ATCC 19718 / CIP 103999 / KCTC 2705 / NBRC 14298)* | CMTDPIADMLTRIR | Trioxidation (C),di-oxidation (M) | 2 | 1771.79 | 3.02 | 217200 |
| Q83D91 | Glucose-6-phosphate isomerase, GPI, EC 5.3.1.9 | *Coxiella burnetii (strain RSA 493 / Nine Mile phase I)* | SKYQELSSLHMR | Oxidation (M) | 3 | 1493.73 | 2.43 | 275480 |
| Q83PD9 | HTH-type transcriptional activator RhaR | *Shigella flexneri* | ITRGDLFYIHADDK | Iodination | 2 | 1788.73 | 1.70 | 4406000 |
| Q86GV3 | Receptor-type guanylate cyclase gcy-28, EC 4.6.1.2 | *Caenorhabditis elegans* | KEYPFLTRMQGSYR | dioxidation Y,O-2H (Y) | 2 | 1820.85 | 2.22 | 1052200 |
| Q88CS3 | Biosynthetic peptidoglycan transglycosylase, EC 2.4.1.129 | *Pseudomonas putida (strain ATCC 47054 / DSM 6125 / NCIMB 11950 / KT2440)* | PSAYVASR | O-2H (Y) | 2 | 863.41 | -0.30 | 1416500 |
| Q88DF3 | UPF0313 protein PP_4872 | *Pseudomonas putida (strain ATCC 47054 / DSM 6125 / NCIMB 11950 / KT2440)* | VGIIAQPNWQSK | Oxidation (W) | 2 | 1355.72 | 1.25 | 3472200 |
| Q88U41 | ATP-dependent helicase/nuclease subunit A, EC 3.1.-.-, EC 3.6.4.12 | *Lactobacillus plantarum (strain ATCC BAA-793 / NCIMB 8826 / WCFS1)* | LESQINKR | Acetyl (K) | 2 | 1028.56 | -0.65 | 3645900 |
| Q88Z86 | Bifunctional protein GlmU [Includes: UDP-N-acetylglucosamine pyrophosphorylase, EC 2.7.7.23 | *Lactobacillus plantarum (strain ATCC BAA-793 / NCIMB 8826 / WCFS1)* | TTKNAIIMAAGKGTR | Oxidation (M),Deamidation (NQ) | 3 | 1548.83 | -1.35 | 99303 |
| Q891R5 | Valine--tRNA ligase, EC 6.1.1.9 (Valyl-tRNA synthetase, ValRS) | *Clostridium tetani (strain Massachusetts / E88)* | VDKKLSNENFVK | 2 Acetyl (K),Deamidation (NQ) | 2 | 1504.78 | 2.91 | 390160 |
| Q892M0 | dTTP/UTP pyrophosphatase, dTTPase/UTPase, EC 3.6.1.9 | *Clostridium tetani (strain Massachusetts / E88)* | IKADYSCSKVK | Oxidation Y | 2 | 1313.66 | -2.49 | 342240 |
| Q89AK1 | Glyceraldehyde-3-phosphate dehydrogenase, GAPDH, EC 1.2.1.12 | *Buchnera aphidicola subsp. Baizongia pistaciae (strain Bp)* | LVSWYDNECGYSSK | Deamidation (NQ),Trioxidation (C) | 2 | 1755.69 | -0.97 | 265360 |
| Q89AK2 | Transcription-repair-coupling factor, TRCF, EC 3.6.4.- | *Buchnera aphidicola subsp. Baizongia pistaciae (strain Bp)* | NIKNGYYKPLNDIINTYPK | 2 Deamidation (NQ),dioxidation Y | 3 | 2301.15 | -1.81 | 402340 |
| Q8A7M7 | Single-stranded DNA-binding protein, SSB | *Bacteroides thetaiotaomicron (strain ATCC 29148 / DSM 2079 / NCTC 10582 / E50 / VPI-5482)* | YITEIFVDNMEMLSPK | Oxidation (M),Oxidation Y | 2 | 1960.92 | 1.94 | 242590 |
| Q8CRA8 | Heme sensor protein HssS, EC 2.7.13.3 | *Staphylococcus epidermidis (strain ATCC 12228 / FDA PCI 1200)* | EYERTQKPK | Deamidation (NQ) | 1 | 1178.59 | -0.27 | 3753500 |
| Q8DF81 | Phosphoribosylformylglycinamidine synthase, FGAM synthase, FGAMS, EC 6.3.5.3 | *Vibrio vulnificus (strain CMCP6)* | IFNADWTIDGVEQPKSLFKMIK | Oxidation (W) | 3 | 2595.34 | -1.45 | 225230 |
| Q8DI26 | RuBisCO accumulation factor 1 | *Thermosynechococcus elongatus (strain BP-1)* | GLAEWDRDR | Trp->Kynurenine | 2 | 1120.53 | 3.56 | 477760 |
| Q8DJM9 | UPF0182 protein tll1193 | *Thermosynechococcus elongatus (strain BP-1)* | QLQQLRSYYR | 3 Deamidation (NQ) | 2 | 1356.67 | -1.84 | 292760 |
| Q8E1H3 | Translation initiation factor IF-2 | *Streptococcus agalactiae serotype V (strain ATCC BAA-611 / 2603 V/R)* | NQRNSNWNHNKK | 2 Deamidation (NQ) | 3 | 1540.71 | -1.62 | 131170 |
| Q8E4M7 | 50S ribosomal protein L7/L12 | *Streptococcus agalactiae serotype III (strain NEM316)* | EITGEGLKEAK | Unmodified | 2 | 1173.62 | -0.52 | 618660 |
| Q8EQA8 | GTPase Der (GTP-binding protein EngA) | *Oceanobacillus iheyensis (strain DSM 14371 / CIP 107618 / JCM 11309 / KCTC 3954 / HTE831)* | AMKEFEKNIR | Acetyl (K),Oxidation (M) | 3 | 1322.67 | -0.10 | 16915000 |
| Q8EWX1 | DNA-directed RNA polymerase subunit beta, RNAP subunit beta, EC 2.7.7.6 (RNA polymerase subunit beta) (Transcriptase subunit beta) | *Mycoplasma penetrans (strain HF-2)* | VLELYYDKK | O-2H (Y) | 2 | 1183.61 | -3.49 | 26886000 |
| Q8FS35 | 30S ribosomal protein S13 | *Corynebacterium efficiens (strain DSM 44549 / YS-314 / AJ 12310 / JCM 11189 / NBRC 100395)* | RMEIALTYIYGIGPAR | O-2H (Y) | 2 | 1836.96 | 3.28 | 164400 |
| Q8G769 | Aspartyl/glutamyl-tRNA(Asn/Gln) amidotransferase subunit B, Asp/Glu-ADT subunit B, EC 6.3.5.- | *Bifidobacterium longum (strain NCC 2705)* | DYQISQYDK | O-2H (Y) | 2 | 1172.50 | 2.28 | 463180 |
| Q8K9F8 | Bifunctional chorismate mutase/prephenate dehydratase | *Buchnera aphidicola subsp. Schizaphis graminum (strain Sg)* | KNNLNTNYITR | Deamidation (NQ),O-2H (Y) | 2 | 1364.67 | 1.76 | 1405500 |
| Q8KAY6 | Glycogen synthase, EC 2.4.1.21 | *Chlorobaculum tepidum (strain ATCC 49652 / DSM 12025 / NBRC 103806 / TLS) (Chlorobium tepidum)* | ETAEENEEK | Glu->pyro-Glu,Deamidation (NQ) | 2 | 1060.42 | -2.84 | 345160 |
| Q8KCJ0 | Delta-aminolevulinic acid dehydratase, ALAD, ALADH, EC 4.2.1.24 | *Chlorobaculum tepidum (strain ATCC 49652 / DSM 12025 / NBRC 103806 / TLS) (Chlorobium tepidum)* | AGADIIFTYYAK | O-2H (Y) | 2 | 1345.66 | -2.46 | 528080 |
| Q8KCM7 | Lysine--tRNA ligase, EC 6.1.1.6 | *Chlorobaculum tepidum (strain ATCC 49652 / DSM 12025 / NBRC 103806 / TLS) (Chlorobium tepidum)* | GKSEAQLR | Acetyl (K) | 2 | 929.49 | 0.60 | 7298100 |
| Q8KIX3 | 60 kDa chaperonin (GroEL protein) | *Buchnera aphidicola subsp. Tetraneura caerulescens* | SDLSVPPQGGMGGMGGMM | 3 Oxidation (M) | 2 | 1755.69 | -2.12 | 247980 |
| Q8NUV0 | Putative surface protein MW2416 | *Staphylococcus aureus (strain MW2)* | SVANQEKKR | Acetyl (K),2 Deamidation (NQ) | 2 | 1102.56 | 2.40 | 2626900 |
| Q8PFH4 | UPF0337 protein XAC4007 | *Xanthomonas axonopodis pv. citri (strain 306)* | LQERYGWDR | Oxidation (W) | 2 | 1237.58 | 0.81 | 71006 |
| Q8PPP2 | Ubiquinone/menaquinone biosynthesis C-methyltransferase UbiE, EC 2.1.1.163, EC 2.1.1.201 | *Xanthomonas axonopodis pv. citri (strain 306)* | ERVGNEGAVVLGDINAGMLSVGR | Oxidation (M),Deamidation (NQ) | 2 | 2329.17 | 1.09 | 157050 |
| Q8R7C0 | Acetylglutamate kinase, EC 2.7.2.8 | *Caldanaerobacter subterraneus subsp. tengcongensis (strain DSM 15242 / JCM 11007 / NBRC 100824 / MB4) (Thermoanaerobacter tengcongensis)* | GYIPVIAPVSFGDDGK | O-2H (Y) | 2 | 1647.81 | 2.92 | 286090 |
| Q8R7V5 | 50S ribosomal protein L4 | *Caldanaerobacter subterraneus subsp. tengcongensis (strain DSM 15242 / JCM 11007 / NBRC 100824 / MB4) (Thermoanaerobacter tengcongensis)* | NMPNAKALYANLLNTYDVLK | Deamidation (NQ),2 Oxidation Y | 2 | 2298.16 | 1.80 | 247860 |
| Q8R9N0 | Tryptophan synthase alpha chain, EC 4.2.1.20 | *Caldanaerobacter subterraneus subsp. tengcongensis (strain DSM 15242 / JCM 11007 / NBRC 100824 / MB4) (Thermoanaerobacter tengcongensis)* | GYNRCEMLKEVK | Oxidation (M),O-2H (Y) | 2 | 1555.71 | 1.79 | 111610 |
| Q8R9T0 | Peptide deformylase, PDF, EC 3.5.1.88 | *Caldanaerobacter subterraneus subsp. tengcongensis (strain DSM 15242 / JCM 11007 / NBRC 100824 / MB4) (Thermoanaerobacter tengcongensis)* | YLDREGNVK | Deamidation (NQ) | 2 | 1093.54 | 1.59 | 426360 |
| Q8RC52 | Glutamate racemase 2, EC 5.1.1.3 | *Caldanaerobacter subterraneus subsp. tengcongensis (strain DSM 15242 / JCM 11007 / NBRC 100824 / MB4) (Thermoanaerobacter tengcongensis)* | AIMGDGVK | Oxidation (M) | 1 | 805.40 | -4.42 | 88465 |
| Q8UFV9 | Proline--tRNA ligase, EC 6.1.1.15 | *Agrobacterium fabrum (strain C58 / ATCC 33970) (Agrobacterium tumefaciens (strain C58))* | EDAIHSYNK | Glu->pyro-Glu,Deamidation (NQ) | 2 | 1058.47 | 0.21 | 947570 |
| Q8VUS5 | [LysW]-lysine hydrolase, EC 3.5.1.130 | *Thermus thermophilus (strain HB27 / ATCC BAA-163 / DSM 7039)* | QVAEMFFDLR | Oxidation (M),Gln->pyro-Glu | 2 | 1253.58 | -1.85 | 796540 |
| Q8X693 | 2-methylcitrate dehydratase, 2-MC dehydratase, EC 4.2.1.79 | *Escherichia coli O157:H7* | AHEIQGCIALENSFNR | 2 Deamidation (NQ),Trioxidation (C) | 2 | 1907.83 | 1.25 | 56559 |
| Q8XKU4 | Enolase, EC 4.2.1.11 (2-phospho-D-glycerate hydro-lyase) | *Clostridium perfringens (strain 13 / Type A)* | AAVPSGASTGIYEAVELR | Unmodified | 2 | 1789.92 | -0.05 | 542040 |
| Q8Y5Z8 | CCA-adding enzyme, EC 2.7.7.72 | *Listeria monocytogenes serovar 1/2a (strain ATCC BAA-679 / EGD-e)* | SLEEDLQRR | Deamidation (NQ) | 2 | 1145.57 | -3.99 | 672210 |
| Q8Y9Y1 | 3D-(3,5/4)-trihydroxycyclohexane-1,2-dione hydrolase, THcHDO hydrolase, EC 3.7.1.22 | *Listeria monocytogenes serovar 1/2a (strain ATCC BAA-679 / EGD-e)* | IAEGYGAKVYRANTK | Iodination,dioxidation Y | 2 | 1797.75 | -1.96 | 1225300 |
| Q8Z1U3 | Maltose/maltodextrin transport system permease protein MalG | *Salmonella typhi* | MAMVQPKSQK | 2 Oxidation (M),Deamidation (NQ) | 2 | 1179.56 | -1.61 | 186890 |
| Q8ZDW5 | Threonine--tRNA ligase, EC 6.1.1.3 | *Yersinia pestis* | LQDAGIRAK | Deamidation (NQ) | 2 | 971.54 | 0.78 | 435530 |
| Q8ZFQ6 | High frequency lysogenization protein HflD homolog | *Yersinia pestis* | QGEAAELTRYTLSLMVLER | Deamidation (NQ),di-oxidation (M) | 3 | 2212.10 | -0.19 | 1124600 |
| Q92ES1 | Uncharacterized Nudix hydrolase lin0387, EC 3.6.- | *Listeria innocua serovar 6a (strain ATCC BAA-680 / CLIP 11262)* | EGWPNYWDLSAAGSALK | Deamidation (NQ),2 O-2H (W) | 2 | 1892.82 | -2.02 | 806130 |
| Q92GR9 | Valine--tRNA ligase, EC 6.1.1.9 (Valyl-tRNA synthetase, ValRS) | *Rickettsia conorii (strain ATCC VR-613 / Malish 7)* | WQQIWQEK | O-2H (W),Oxidation (W) | 2 | 1174.54 | -0.59 | 89069 |
| Q92IQ1 | Elongation factor 4, EF-4, EC 3.6.5.n1 (Ribosomal back-translocase LepA) | *Rickettsia conorii (strain ATCC VR-613 / Malish 7)* | IESMEEPWIK | Oxidation (M),Di-oxidation W | 2 | 1308.59 | 2.93 | 247840 |
| Q92IT8 | UDP-N-acetylenolpyruvoylglucosamine reductase, EC 1.3.1.98 | *Rickettsia conorii (strain ATCC VR-613 / Malish 7)* | LEWEIKRIGR | O-2H (W) | 2 | 1312.73 | -1.11 | 393410 |
| Q939N5 | Platelet binding protein GspB | *Streptococcus gordonii* | ISKGQLPR | Deamidation (NQ) | 1 | 898.52 | 3.15 | 1005700 |
| Q95X91 | Nuclear hormone receptor family member nhr-96 | *Caenorhabditis elegans* | LEDWMKLDILK | Oxidation (M) | 2 | 1418.75 | 2.31 | 136680 |
| Q97FK1 | Nuclease SbcCD subunit C | *Clostridium acetobutylicum (strain ATCC 824 / DSM 792 / JCM 1419 / LMG 5710 / VKM B-1787)* | ENTMKAISLEKEDMEK | Oxidation (M),Deamidation (NQ) | 3 | 1911.88 | 2.08 | 1042300 |
| Q97JA9 | Cobalt-precorrin-5B C(1)-methyltransferase, EC 2.1.1.195 | *Clostridium acetobutylicum (strain ATCC 824 / DSM 792 / JCM 1419 / LMG 5710 / VKM B-1787)* | LEMNQKIK | Oxidation (M),Deamidation (NQ) | 2 | 1019.53 | -2.58 | 342240 |
| Q97QW6 | UDP-N-acetylglucosamine 1-carboxyvinyltransferase 2, EC 2.5.1.7 | *Streptococcus pneumoniae serotype 4 (strain ATCC BAA-334 / TIGR4)* | INNVLYEHLEGFIAK | Deamidation (NQ),O-2H (Y) | 2 | 1773.89 | -1.31 | 1045300 |
| Q985G2 | Cytochrome c-type biogenesis protein CcmE | *Mesorhizobium japonicum (strain LMG 29417 / CECT 9101 / MAFF 303099) (Mesorhizobium loti (strain MAFF 303099))* | AKGVWQESK | Deamidation (NQ),Di-oxidation W | 2 | 1064.51 | 0.96 | 2,60E+08 |
| Q98PZ7 | 50S ribosomal protein L6 | *Mycoplasma pulmonis (strain UAB CTIP)* | GIQYKDEKLR | Acetyl (K) | 2 | 1290.69 | 3.68 | 1599500 |
| Q98QQ2 | tRNA(Met) cytidine acetate ligase, EC 6.3.4.- | *Mycoplasma pulmonis (strain UAB CTIP)* | YTPMIFEKK | Oxidation (M) | 2 | 1171.59 | -2.03 | 38702 |
| Q99W46 | Serine-aspartate repeat-containing protein E | *Staphylococcus aureus (strain N315)* | LGNGSTIIDQNTEIK | 3 Deamidation (NQ) | 2 | 1604.78 | 2.04 | 1244000 |
| Q9AHD4 | Tyrosine-protein phosphatase CpsB, EC 3.1.3.48 | *Streptococcus pneumoniae serotype 4 (strain ATCC BAA-334 / TIGR4)* | KGMFETPEEKIAENFLQVR | 2 Deamidation (NQ) | 3 | 2267.11 | 2.39 | 901070 |
| Q9AKJ0 | Arp2/3 complex-activating protein rickA (Actin polymerization protein rickA) | *Rickettsia rickettsii* | INNRNSQK | Deamidation (NQ) | 1 | 973.49 | 3.76 | 121550 |
| Q9CK97 | Methionine import ATP-binding protein MetN, EC 7.4.2.11 | *Pasteurella multocida (strain Pm70)* | DAYPSNLSGGQKQR | Deamidation (NQ) | 2 | 1520.72 | 2.67 | 1116700 |
| Q9CMZ1 | Alpha-2-macroglobulin | *Pasteurella multocida (strain Pm70)* | AIKAYSASEGQIK | Deamidation (NQ),Oxidation Y | 2 | 1381.71 | 1.55 | 1049800 |
| Q9GTD4 | Nuclear hormone receptor family member nhr-71 | *Caenorhabditis elegans* | ISTDYSNSPDRYR | Iodination,Oxidation Y | 2 | 1714.61 | 2.50 | 193150 |
| Q9HYF0 | tRNA 5-methylaminomethyl-2-thiouridine biosynthesis bifunctional protein MnmC, tRNA mnm(5)s(2)U biosynthesis bifunctional protein | *Pseudomonas aeruginosa (strain ATCC 15692 / DSM 22644 / CIP 104116 / JCM 14847 / LMG 12228 / 1C / PRS 101 / PAO1)* | TVVCAEGYVAPPR | O-2H (Y) | 2 | 1431.68 | 0.91 | 334610 |
| Q9I4U3 | DNA recombination protein RmuC homolog | *Pseudomonas aeruginosa (strain ATCC 15692 / DSM 22644 / CIP 104116 / JCM 14847 / LMG 12228 / 1C / PRS 101 / PAO1)* | EAYQQLGER | 2 Deamidation (NQ) | 2 | 1094.49 | 3.11 | 57877 |
| Q9I702 | Putative 3-oxopropanoate dehydrogenase, EC 1.2.1.- | *Pseudomonas aeruginosa (strain ATCC 15692 / DSM 22644 / CIP 104116 / JCM 14847 / LMG 12228 / 1C / PRS 101 / PAO1)* | NTPPAKRAQVLYR | 2 Deamidation (NQ),Iodination | 2 | 1640.72 | -0.55 | 272940 |
| Q9JYJ6 | tRNA-specific 2-thiouridylase MnmA, EC 2.8.1.13 | *Neisseria meningitidis serogroup B (strain MC58)* | AGRYTCKTR | O-2H (Y) | 2 | 1125.53 | 0.44 | 1050000 |
| Q9JYM0 | Thiol:disulfide interchange protein DsbD, EC 1.8.1.8 | *Neisseria meningitidis serogroup B (strain MC58)* | FLQPSSQNGSGALPPPK | 2 Deamidation (NQ) | 3 | 1725.86 | 3.57 | 424100 |
| Q9JZZ5 | ATP-dependent Clp protease adapter protein ClpS | *Neisseria meningitidis serogroup B (strain MC58)* | DIAQTKQQQVMQR | Oxidation (M),2 Deamidation (NQ) | 2 | 1590.77 | -1.23 | 419260 |
| Q9K6A7 | Octanoyl-[GcvH]:protein N-octanoyltransferase, EC 2.3.1.204 | *Bacillus halodurans (strain ATCC BAA-125 / DSM 18197 / FERM 7344 / JCM 9153 / C-125)* | SLLLQQHLSQPWR | 2 Deamidation (NQ),Di-oxidation W | 2 | 1638.84 | 1.72 | 217280 |
| Q9KMW9 | GMP reductase, EC 1.7.1.7 (Guanosine 5'-monophosphate oxidoreductase | *Vibrio cholerae serotype O1 (strain ATCC 39315 / El Tor Inaba N16961)* | IIGDGGCTCPGDVAK | 2 Trioxidation (C) | 2 | 1614.65 | -0.50 | 3673600 |
| Q9KVX3 | DNA gyrase subunit B, EC 5.6.2.2 | *Vibrio cholerae serotype O1 (strain ATCC 39315 / El Tor Inaba N16961)* | TLNNYMDK | Deamidation (NQ),Iodination | 2 | 1124.33 | 0.97 | 1241900 |
| Q9PLL2 | Glycerol-3-phosphate dehydrogenase [NAD(P)+], EC 1.1.1.94 | *Chlamydia muridarum (strain MoPn / Nigg)* | MLAEGLTPEQAKTK | Oxidation (M) | 2 | 1531.79 | 3.56 | 7373200 |
| Q9PML1 | UPF0306 protein Cj1449c | *Campylobacter jejuni subsp. jejuni serotype O:2 (strain ATCC 700819 / NCTC 11168)* | IYALELFWLK | Iodination | 3 | 1420.63 | -0.83 | 870350 |
| Q9PQE9 | Cytidylate kinase, CK, EC 2.7.4.25 | *Ureaplasma parvum serovar 3 (strain ATCC 700970)* | DIGSIVLTNADLK | Deamidation (NQ) | 2 | 1358.73 | 1.96 | 2337700 |
| Q9PQX0 | Uncharacterized protein UU173 | *Ureaplasma parvum serovar 3 (strain ATCC 700970)* | YITKYDYIGYYTK | 2 Di-iodination,Oxidation Y | 3 | 2209.41 | 2.15 | 148000 |
| Q9R9H8 | Intracellular maltogenic amylase, EC 3.2.1.- | *Bacillus subtilis* | IMLDAVFNHIGSASPQWQDVVKNGDQSR | Acetyl (K),Oxidation (M),3 Deamidation (NQ) | 3 | 3172.48 | 1.77 | 334470 |
| Q9RJ50 | Tyrosine--tRNA ligase, EC 6.1.1.1 | *Streptomyces coelicolor (strain ATCC BAA-471 / A3(2) / M145)* | MLTKDSVAR | Acetyl (K),di-oxidation (M) | 2 | 1093.54 | -1.75 | 431540 |
| Q9RTY5 | 3-isopropylmalate dehydratase small subunit 1, EC 4.2.1.33 | *Deinococcus radiodurans (strain ATCC 13939 / DSM 20539 / JCM 16871 / LMG 4051 / NBRC 15346 / NCIMB 9279 / R1 / VKM B-1422)* | IWKFGDSVNTDDILPGK | Deamidation (NQ),Oxidation (W) | 3 | 1920.95 | -1.34 | 187780 |
| Q9RXB5 | Uncharacterized FtsK-like protein DR_0400 | *Deinococcus radiodurans (strain ATCC 13939 / DSM 20539 / JCM 16871 / LMG 4051 / NBRC 15346 / NCIMB 9279 / R1 / VKM B-1422)* | SQLDFSDPLLR | Unmodified | 2 | 1289.66 | 0.01 | 370230 |
| Q9RXK6 | 30S ribosomal protein S7 | *Deinococcus radiodurans (strain ATCC 13939 / DSM 20539 / JCM 16871 / LMG 4051 / NBRC 15346 / NCIMB 9279 / R1 / VKM B-1422)* | MAEANRAYAHYRW | Deamidation (NQ),O-2H (W) | 2 | 1652.72 | 4.16 | 281190 |
| Q9TYN3 | Transcription initiation factor TFIID subunit 2 | *Caenorhabditis elegans* | VTLNGVECEYSRR | Trioxidation (C) | 2 | 1629.74 | 0.56 | 1366500 |
| Q9X105 | Endonuclease MutS2, EC 3.1.-.- | *Thermotoga maritima (strain ATCC 43589 / MSB8 / DSM 3109 / JCM 10099)* | LSSEIKR | Unmodified | 2 | 831.48 | 2.33 | 3,87E+08 |
| Q9X220 | Probable tRNA sulfurtransferase, EC 2.8.1.4 | *Thermotoga maritima (strain ATCC 43589 / MSB8 / DSM 3109 / JCM 10099)* | EKLEKGNYR | dioxidation Y | 2 | 1167.59 | -1.49 | 1434800 |
| Q9XW70 | Bardet-Biedl syndrome 7 protein homolog | *Caenorhabditis elegans* | VEVACELNQDSVNHCLK | 3 Deamidation (NQ) | 3 | 2016.88 | 0.09 | 193950 |
| Q9Z507 | UvrABC system protein A, UvrA protein | *Streptomyces coelicolor (strain ATCC BAA-471 / A3(2) / M145)* | CENCAGDGTIK | 2 Trioxidation (C) | 2 | 1319.46 | 4.37 | 1561100 |
| Q9Z6W6 | UvrABC system protein C, Protein UvrC | *Chlamydia pneumoniae (Chlamydophila pneumoniae)* | TGYGKELLDLAYR | 2 dioxidation Y | 2 | 1561.76 | 3.48 | 351030 |
| Q9Z6Z6 | tRNA dimethylallyltransferase, EC 2.5.1.75 | *Chlamydia pneumoniae (Chlamydophila pneumoniae)* | KVSDHEWDIVPKASR | Trp->Kynurenine | 2 | 1769.91 | -1.94 | 231810 |
| Q9Z812 | Probable outer membrane protein pmp20 | *Chlamydia pneumoniae (Chlamydophila pneumoniae)* | KGYLGTWNLDPNSSGSK | Deamidation (NQ),Di-oxidation W | 2 | 1855.86 | -0.14 | 896950 |
| Q9ZDF2 | ADP,ATP carrier protein 2 (ADP/ATP translocase 2) | *Rickettsia prowazekii (strain Madrid E)* | EMVYIPLDNEIK | Oxidation (M),Glu->pyro-Glu | 2 | 1460.72 | 1.50 | 939450 |
| Q9ZKB3 | Phosphogluconate dehydratase, EC 4.2.1.12 | *Helicobacter pylori (strain J99 / ATCC 700824) (Campylobacter pylori J99)* | TRELYLENTFNPKNQPK | 3 Deamidation (NQ) | 2 | 2094.03 | 0.56 | 284490 |

***Control blank sample: Analysis of an empty EVA diskette***

An empty EVA diskette was processed in the same way of the sample. The raw data were analyzed against the three databases described in the main manuscript and all the chemical modifications observed in the sample were searched. It was possible to identify a meagre number of peptides. BLAST search revealed that most of these peptides were unspecific as taxonomy. Moreover, none of the peptides identified was in common with the peptides in the sample. The results of control blank sample are showed in Table S5.

***Table S5****. Peptides identified in the control blank sample (empty EVA diskette) by searching MS data in the three databases of Proboscidea, Viridiplantae, and Bacteria/Nematode.*

| **Database Investigated** | **Sequence** | **Modifications** | **Charge** | **Theoretical Mass** | **Mass error [ppm]** | **MaxQuant Intensity** | **BLAST Result** |
| --- | --- | --- | --- | --- | --- | --- | --- |
| *Proboscidea* | EAALIALR | Unmodified | 2 | 855.52 | -0.16 | 20586000 | Not specific |
|  | KMKSCLEFSLR | 2 Acetyl (K), trioxidation (C),  di-oxidation (M) | 2 | 1561.71 | -3.83 | 286190 | Not specific |
|  | QIAWEQKQR | Acetyl (K),deamidation (NQ) | 2 | 1228.62 | 0.59 | 659220 | Not specific |
|  | VKELSPR | Acetyl (K) | 2 | 869.50 | 0.24 | 336790 | Not specific |
|  | LQEELNKAMGIK | Oxidation (M),deamidation (NQ) | 2 | 1389.717 | -4.49 | 199060 | Not specific |
| *Bacteria/*  *Nematoda* | ISVAIDPR | Unmodified | 2 | 869.50 | 0.24 | 336790 | Not specific |
|  | VVADLTPR | Unmodified | 2 | 869.50 | 0.18 | 271040 | Not specific |
|  | ALRQGKR | Unmodified | 1 | 827.51 | 1.45 | 713610 | Not specific |
|  | IAIPKER | Unmodified | 2 | 825.51 | -0.19 | 132160 | Not specific |
| *Viridiplantae* | EVGDGGEKKK | Glu->pyro-Glu | 2 | 1027.53 | 1.67 | 30136 | *Arabidopsis thaliana* |
|  | LAPIQRK | Deamidation (NQ) | 2 | 825.51 | -0.19 | 132160 | Not specific |
|  | LLDEIIVK | Unmodified | 2 | 941.58 | -2.20 | 102930 | Not specific |
|  | QGKNELWWSK | Deamidation (NQ),2 oxidation (W) | 3 | 1307.61 | 0.17 | 68529 | *Arabidopsis thaliana* |

***Calculation of the level of chemical modififcations***

Taking into account that common protein background comprised human keratins together with several skin and saliva proteins introduced during sample handling, both database search and calculation of deamidation level were carried out using the common Repository of Adventitious Proteins (c-RAP) database (URL at <ftp://ftp.thegpm.org/fasta/cRAP> ), a predefined contaminants database for proteomics as background. Then, we calculated the deamidation level of asparagine and glutamine residues of potential contaminants peptides and compared it with that of the potential endogenous peptides identified in the gut of the Shandrin mammoth (results are reported in the main text and in Figure 4).

The code for the method of the calculation is freely available to the scientific community on GitHub (<https://github.com/dblyon/deamidation>). The script calculation is described as follow.

MaxQuant’s “evidence.txt” file was used to calculate separate deamidation rates for Asparagine (N) and Glutamine (Q). The fraction of num_N (number of Asparagines) to num_N-2-D (number of deamidated Asparagines) and the fraction num_Q (number of Glutamines) to num_N-2-D (number of deamidated Glutamines) were calculated for each peptide-to-spectrum match (PSM). The values obtained were termed respectively ratio_N-2-D and ratio_Q-2-E. The ratio_N-2-D or ratio_Q-2-E was multiplied for the “Intensity” of the PTM, the values were summed and the result divided by the total sum of all intensity values of the respective unmodified peptide sequence, obtaining a deamidation rate between 0 and 1 for each unique peptide sequence and charge state. For each peptide was calculated an average deamidation rate for Asparagine and Glutamine. The deamidation rates were averaged per sample. The latter set of values was sampled with replacement (bootstrapped) 1000 times. The mean, the standard deviation, and the 95% confidence intervals were calculated in order to achieve an estimate of the error of the calculation.

The program generates four delimited text files as output:

- Deamidation.txt (Raw Files, deamidation for N and Q, as mean, standard deviation, 95% confidence lower and upper limit)
- Number_of_Peptides_per_RawFile.txt
- Bootstrapped_values.txt (all the deamidation percentages calculated by e.g. 1000 bootstrap iterations, which are subsequently used to calculate the mean, std, and CI for shown in "Deamidation.txt")
- Protein_deamidation.txt (deamidation on the protein level, to be used with restraint since there usually are few data to acquire meaningful results, therefore no bootstrapping is applied)

Moreover, proteins are subject to damage because of the exposure to light or oxidative environmental factors. Many amino acids residues undergo modifications, such as oxidation, which could be index of photo-oxidative or aging damage. Therefore, other chemical modifications investigated for potential contaminants peptides and compared with those of the potential endogenous peptides (see Supplementary Figure S1).

Estimation of the percentage of the other diagenetic chemical modifications (DCM) investigated was obtained applying the same model of the deamidation script, separately for potentially original and potentially contaminant peptides. In detail, for each peptide-sequence containing the residue of interest, the ratio between the number of residues in the modified form multiplied for the intensities of their peptides and the total number of the residues multiplied for the intensities of their peptides was calculated. The values obtained for each peptide (0-1) were averaged per group (potentially original and potentially contaminant peptides) and multiplied by 100 as follows:

$$DCM_{\%}= \frac{1}{n}\sum_{i=0}^{n} \frac{x_{mod}\cdot f_{mod}\cdot I_{mod}}{x_{tot}\cdot f_{tot}\cdot I_{tot}}\cdot100$$

*x = number of residues per peptide*

*f = number of MS scan per peptide*

*I = Intensity of a peptide*

***Results of the oxidation level in ancient and contaminants peptides***

Figure S1 reports the results about the calculated oxidation level in ancient and contaminants peptides. If mono-oxidation of methionine (Met) can be an artifact of the analytical method (it is observed in the contaminant peptides with higher values), the observed values of Met di-oxidation for potential endogenous peptides, ranging from 20 to 37% for original (endogenous) peptides, are higher respect to those of contaminant peptides (about 4%). There is little evidence for direct UV/visible damage to Met residues; however, this oxidative damage of the peptides could be linked to a spontaneous aging process of the sample (Stadtman et al. 2005). Regarding the oxidation of cysteine (Cys) residues, for all the three groups of endogenous peptides, the observed level of tri-oxidation was very high, ranging from 62 to 74%. On the contrary, the level of Cys tri-oxidation in contaminant peptides is remarkably lower, as it ranges from 10 to 15%.

The same trend observed for Met residues, is reported for tryptophan (Trp); indeed, if mono-oxidation level in contaminant and original peptides presents comparable values, di-oxidation of Trp for original peptides is between 16 and 27%, whereas ranges from 5 to 11% for contaminant peptides. Similarly, kynurenine level ranges from 13 and 27% in original peptides and from 4 to 13% for contaminant peptides. Important differences can be observed in the percentage of formation of oxolactone, because it ranges from 25 to 37% in original peptides, whereas is always below 0.5% in contaminant peptides. Finally, all the observed modification of tyrosine (Tyr) residues (i.e. mono- and di-oxidation, and formation of dopaquinone) are significantly higher in original peptides respect to contaminants. In detail, mono-oxidation ranges from 19 to 26% in original peptides, whereas it is about 1.5% in contaminants; di-oxidation of Tyr ranges from 16 to 23%, and from 2.5 to 3.5% in original and contaminant peptides, respectively; level of dopaquinone formation is in the range 17-22% for original peptides, whereas is about 1-4% in contaminants.

Overall, these results show that original endogenous peptides present a higher level of oxidation/damage respect to the contaminant ones.


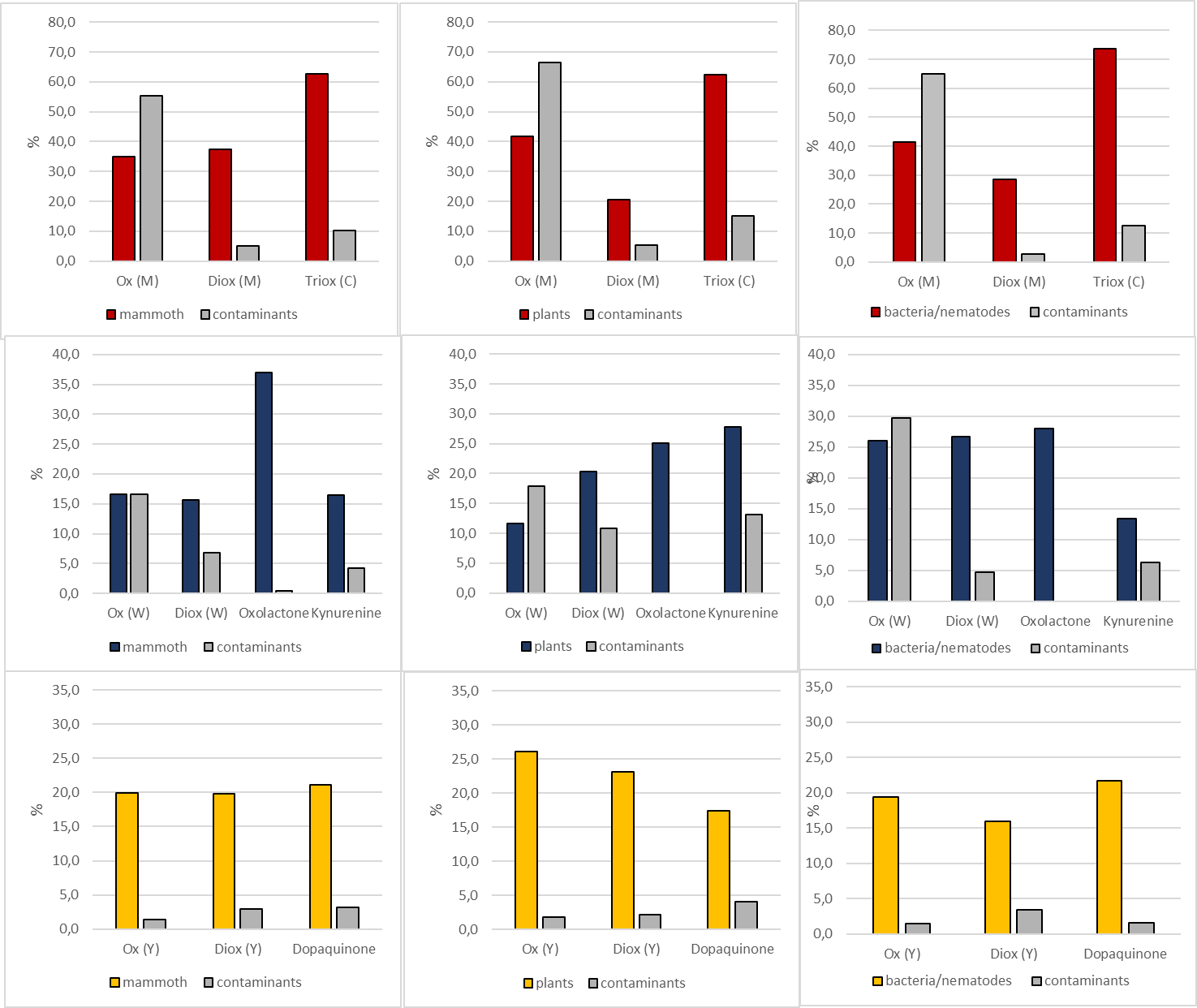


***Figure S1****. Level of DCMs for endogenous (Proboscidea, Viridiplantae, and Bacteria/Nematode) and C-Rap peptides. a) oxidation and di-oxidation of methionine residues; tri-oxidation of cysteine residues; b) oxidation, di-oxidation, and formation of kynerunine of tryptophan residues; c) oxidation, di-oxidation, and formation of dopaquinone of tyrpsine residues.*
